# Alkyl halides as both hydride and alkyl sources in catalytic regioselective reductive olefin hydroalkylation

**DOI:** 10.1038/s41467-020-19717-6

**Published:** 2020-11-17

**Authors:** Xianxiao Chen, Weidong Rao, Tao Yang, Ming Joo Koh

**Affiliations:** 1 Jiangsu Provincial Key Lab for the Chemistry and Utilization of Agro-Forest Biomass, College of Chemical Engineering, Nanjing Forestry University, Nanjing, 210037 China; 2 Department of Chemistry, National University of Singapore, 12 Science Drive 2, Singapore, 117549 Republic of Singapore

**Keywords:** Homogeneous catalysis, Reaction mechanisms, Synthetic chemistry methodology

## Abstract

Among the plethora of catalytic methods developed for hydrocarbofunctionalization of olefins to date, reactions that regioselectively install a functionalized alkyl unit at the 2-position of a terminal unactivated C=C bond to afford branched products are scarce. Here, we show that a Ni-based catalyst in conjunction with a stoichiometric reducing agent promote Markovnikov-selective hydroalkylation of unactivated alkenes tethered to a recyclable 8-aminoquinaldine directing auxiliary. These mild reductive processes employ readily available primary and secondary haloalkanes as both the hydride and alkyl donor. Reactions of alkenyl amides with ≥ five-carbon chain length regioselectively afforded β-alkylated products through remote hydroalkylation, underscoring the fidelity of the catalytic process and the directing group’s capability in stabilizing five-membered nickelacycle intermediates. The operationally simple protocol exhibits exceptional functional group tolerance and is amenable to the synthesis of bioactive molecules as well as regioconvergent transformations.

## Abstract (web summary)

Methods that regioselectively install a functionalized alkyl unit at the 2-position of a terminal unactivated C=C bond are scarce. Here, the authors report a Markovnikov-selective hydroalkylation of unactivated amide-tethered alkenes catalyzed by nickel in conjunction with a stoichiometric reductant.

## Introduction

**Fig. 1:**
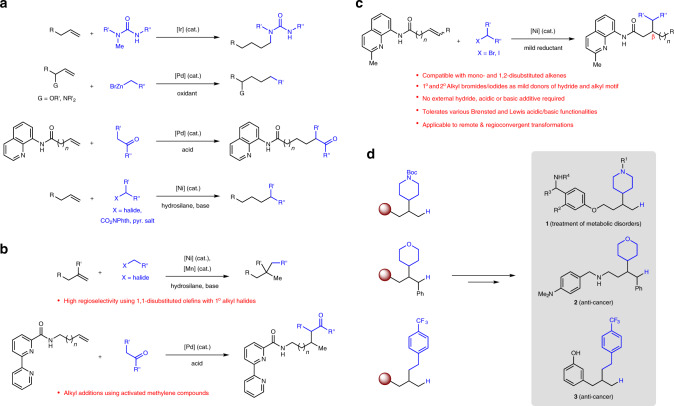
The significance of developing branched- and β-selective hydroalkylation of unactivated alkenes. **a** Reported methods that involve anti-Markovnikov-selective hydroalkylation of aliphatic olefins. **b** Reported methods that involve Markovnikov-selective hydroalkylation of aliphatic olefins. **c** An attractive catalytic approach for Markovnikov- and β-selective olefin hydroalkylation takes advantage of haloalkanes to transfer the hydride and alkyl motif under mild reductive conditions without additional hydrosilane, acidic or basic additives. **d** The resulting hydroalkylation products are versatile building blocks that may be used to access biologically active molecules. R, functional group; Phth, phthaloyl; pyr., pyridinium; cat., catalyst.

The abundance, low cost and distinct reactivity profiles of alkenes have enabled these feedstock molecules to be widely utilized in olefin functionalization reactions for various chemical synthesis applications^1–3^. In this respect, the installation of a hydrogen and carbon-based moiety across π-systems represents an effective strategy for C–C bond construction^4–6^. Numerous hydrocarbofunctionalization protocols rely on conjugation (i.e., 1,3-dienes^7–11^, olefins such as styrenes^12–14^, alkenyl boronates^15,16^, and Michael acceptors^17^) to deliver high regioselectivity. In contrast, reactions with unactivated aliphatic C=C bonds are typically plagued by lower substrate reactivity and/or poorer regiochemical differentiation leading to unsatisfactory levels of site selectivity. Notwithstanding these difficulties, remarkable advances for both anti-Markovnikov^18–25^ (linear)- and Markovnikov^26–30^ (branched)-selective hydroarylations of alkyl-substituted alkenes have been made. A single report on Pd-catalyzed olefin hydroalkynylation/hydroalkenylation to afford linear products was also recently disclosed^31^. On the other hand, there is growing demand for methods that furnish C–C bonds between two sp^3^-hybridized motifs, which are crucial for assembling the skeletal backbone of organic entities en route to bioactive compounds^32,33^. Accordingly, hydroalkylations across aliphatic olefins have been devised, although the vast majority involved linear-selective additions (Fig. 1a). Of these cases, either a limited range of ureas^34^, organometallic reagents^35,36^ or carbonyl compounds^37–39^ were employed as nucleophiles, or an exogenous protic source^40^ or hydrosilane/base reagent^41–45^ is needed to (i) promote protodemetallation or (ii) generate the requisite metal-hydride species.

To date, highly Markovnikov-selective hydroalkylations of aliphatic π-systems have been largely achieved with 1,1-disubstituted/trisubstituted alkenes and primary haloalkanes through a Mn/Ni dual catalytic metal-hydride hydrogen atom transfer approach^46^, or with olefins linked to a tridendate directing group and 1,3-dicarbonyl nucleophiles using a Pd-based catalyst^40^ (Fig. 1b). Therefore, there is compelling motivation to develop a complementary catalytic regime that accomplishes efficient and branched-selective hydroalkylation of unactivated acyclic olefins with exceptional control of regioselectivity in the presence of commonly occurring functionalities. For operational simplicity, we speculated that aliphatic halides could serve as mild donors of both the hydride (by facile in situ β-H elimination^47,48^) and alkyl component without external acidic or basic additives, which might otherwise compromise functional group compatibility (Fig. 1c). The products resulting from successful implementation of this strategy can be readily elaborated to a variety of important biologically active compounds (e.g., **1**–**3**, Fig. 1d). Herein, we report a directed Ni-catalyzed reductive protocol that achieves these goals.

## Results

### Reaction design and optimization

A hallmark of catalytic reductive transformations^49–57^ is the use of stoichiometric amounts of an inexpensive reducing agent to drive single-electron transfer processes mediated by an appropriate (e.g., Ni-based) catalyst. This led us to conceive a reductive strategy for alkene hydroalkylation that takes advantage of the characteristic mild reaction conditions. Specifically, we aimed to avoid the use of hydrosilanes, acidic and basic reagents that could engender undesired side reactions with certain sensitive functional units (see below for further discussion). However, the question remains how we could effectively deliver hydride to the C=C bond in this scenario.

**Fig. 2:**
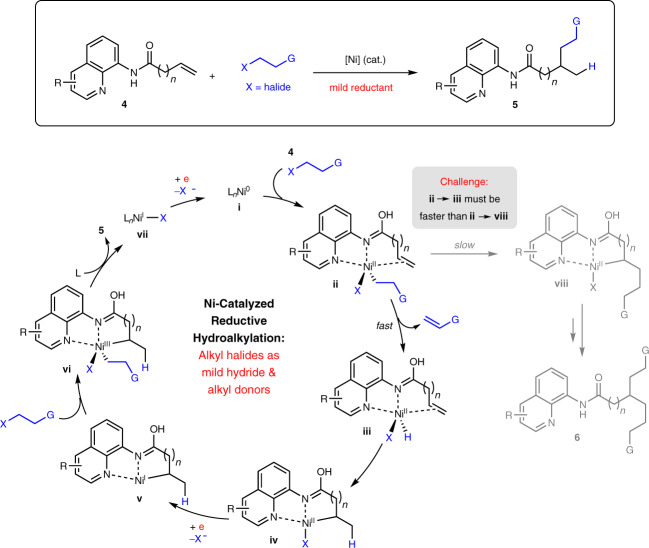
The challenges involved in developing site-selective reductive hydroalkylation reactions. As shown in the Ni catalytic cycle, the key to efficient transformation of **4** to **5** requires faster conversion of intermediate **ii** to **iv** in order to suppress adventitious formation of the undesired dialkylation adduct **6**. Under the reductive conditions, both the hydrogen and alkyl unit are derived from the haloalkane reagent. R, G, functional group; X, halide; L, ligand; cat., catalyst.

To this end, we envisioned a catalytic approach that merges aliphatic alkenes tethered to a suitable directing unit **4**^58^ with alkyl halides directly in the presence of a Ni-based catalyst and a reductant (Fig. 2). This strategy relies on the propensity of haloalkanes to undergo facile β-H elimination, an observation that was previously exploited in Ni catalysis to generate catalytic amounts of nickel-hydride species^47,48^. As illustrated in the putative catalytic pathway, a Ni(0) species **i** could first associate with **4** and react with an equivalent of the alkyl halide through a halogen atom abstraction/radical recombination^59,60^ process to give **ii**. At this juncture, a directing auxiliary with appropriate steric and/or electronic properties could induce alkene dissociation from the metal center in **ii**, providing an opportunity for the Ni–alkyl moiety to preferentially undergo β-H elimination^47,48^ leading to nickel-hydride **iii** with concomitant discharge of an alkene by-product (vs. olefin alkylnickelation to form **viii**). Regioselective β-hydride insertion across the associated C=C bond in **iii** then affords quinoline-chelated Ni(II) species **iv**. Following single-electron reduction with a reducing agent, Ni(I) complex **v** would be generated that could subsequently react with a second equivalent of alkyl halide to furnish **vi**. Reductive elimination of **vi** furnishes the desired hydroalkylation product **5** and **vii**, which eventually gets reduced back to **i** to turn over the catalytic cycle. The key to efficient transformation of **4** to **5** entails faster conversion of intermediate **ii** to **iv** via **iii** (vs. **ii** to **viii**) in order to suppress adventitious formation of the undesired dialkylation adduct **6**.

We first examined conditions that facilitate hydroalkylation of β,γ-unsaturated amides with 1-iodobutane **8** (Table 1). After an extensive survey, we found that the reaction between 8-aminoquinaldine-tethered alkene **7a** and **8** (2 equiv.) using NiCl_2_(PPh_3_)_2_ (15 mol%), manganese (1.5 equiv.) as reductant and NMP as solvent gave the best results, affording **9a** in 90% GC (88% isolated) yield and complete Markovnikov selectivity at ambient temperature (Table 1, entry 1). There was no appreciable diminution in yield or selectivity when the reaction was carried out on larger scale (2 mmol). Intriguingly, changing the directing group to other variants gave poorer yields and site selectivities, demonstrating the unexpected beneficial role of the *ortho*-methyl appendage. We reasoned that the effect might arise from an elevated steric strain inherent within complex **ii** (cf. Fig. 2), forcing Ni-olefin dissociation and allowing the sterically less demanding C_β_–H bond to coordinate and trigger β-H elimination^47,48^ prior to the ensuing β-H insertion to **iv**. Further studies to rationalize this effect will be reported in due course.

**Table 1:** Evaluation of reaction conditions for reductive hydroalkylation.


Entry	Deviation from standard conditions	Yield (%)	9a:10a^a^
1	None	90(88)(81^b^)	>95:5
2	NiCl_2_, NiCl_2_·DME, NiCl_2_(Py)_4_, NiI_2_ or Ni(COD)_2_ instead of NiCl_2_(PPh_3_)_2_	50–74	92:8–>95:5
3	DMF, DMA, DMPU or MeCN instead of NMP	70–92	92:8–94:6
4	DMSO, THF or toluene instead of NMP	Trace–10	ND
5	No Mn	Trace	ND
6	Mn (1 equiv.) instead of Mn (1.5 equiv.)	70	>95:5
7	Mn (2 equiv.) instead of Mn (1.5 equiv.)	90	>95:5
8	Zn instead of Mn	44	80:20
9	8 (1.5 equiv.) instead of 8 (2 equiv.)	70	>95:5
10	8 (2.5 equiv.) instead of 8 (2 equiv.)	91	>95:5
11	40 °C instead of RT	90	>95:5
12	10 mol % NiCl_2_(PPh_3_)_2_ instead of 15 mol %	92	92:8

Reactions were carried out on 0.1 mmol scale.

*DMA* *N,N*-dimethylacetamide, *DMF N,N*-dimethylformamide, NMP *N*-methyl-2-pyrrolidone, DMPU *N,N*′-dimethylpropyleneurea, *DMSO* dimethyl sulfoxide, *THF* tetrahydrofuran, *DME* 1,2-dimethoxyethane, *Py* pyridine, *COD* 1,5-cyclooctadiene, *RT* room temperature, *ND* not determined.

^a^Yields and regioisomeric ratios were determined by GC analysis with *n*-tridecane as internal standard. Values in parentheses denote yields for isolated and purified products.

^b^The reaction was conducted on 2 mmol scale.

Changing the Ni-based catalyst afforded lower yields across the board (Table 1 entry 2), while replacing NMP with other solvents did not improve results (Table 1, entries 3–4). Unsurprisingly, the reaction did not proceed in the absence of Mn, with 1.5 equiv. of Mn being optimal (Table 1, entries 5–7). Switching Mn to Zn as the reductant led to a drastic reduction in yield and regioselectivity (Table 1 entry 8). Decreasing the equivalents of **8** resulted in diminished reaction efficiency, whereas no appreciable improvement was detected at higher loadings (Table 1 entries 9–10). Carrying out the reaction at 40 °C gave similar results as RT (Table 1, entry 11). A slight drop in site selectivity was observed with 10 mol% loading of NiCl_2_(PPh_3_)_2_ (Table 1, entry 12). Performing the hydroalkylation by replacing Mn with hydrosilane/base as the hydride source^41–45^ led to lower yields of **9a** (see Supplementary Methods 2.6 for details), highlighting the importance of the reductive conditions.

### Substrate scope

**Fig. 3:**
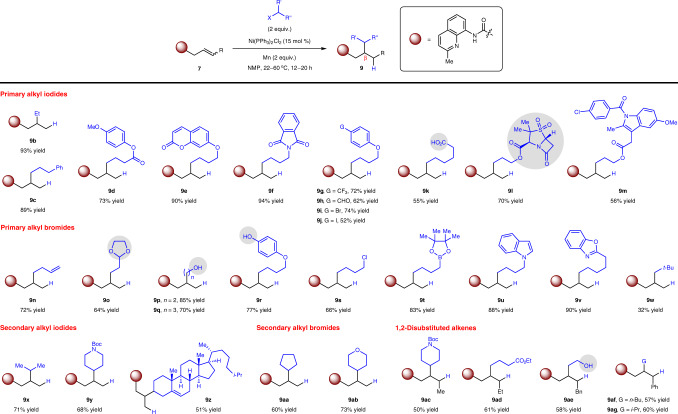
The range of products accessible by reductive olefin hydroalkylation. The protocol is compatible with both primary and secondary alkyl halides bearing Brønsted/Lewis acidic and basic functionalities, including those derived from complex bioactive molecules. Both mono- and 1,2-disubstituted alkenyl amides are tolerated in the catalytic system. For **9w**, the reaction was conducted with neopentyl bromide (3 equiv.) and isopropyl bromide (1.2 equiv.) using NiI_2_ as the catalyst. For **9ac** and **9af**, reactions were conducted using iodides (X = I). For **9ad**, **9ae**, and **9ag**, reactions were conducted using bromides (X = Br). For **9j**, ~20% of an inseparable hydrodeiodination side product was detected. For **9m**, ~3% of an inseparable self-coupling side product of iodide substrate was detected. **9g**, **9i**, **9u**, **9ab**, and **9af** were obtained as 88:12, 93:7, 92:8, 90:10, and 91:9 regioisomeric mixtures, respectively. **9l** and **9z** were obtained as 5:1 and 1:1 diastereomeric mixtures, respectively. Regioisomeric and diastereomeric ratios were determined by ^1^H NMR analysis. Yields are for isolated and purified products. R, functional group; X, halide; NMP, *N*-methyl-2-pyrrolidone; Boc, *tert*-butoxycarbonyl.

With the established conditions in hand, we proceeded to evaluate the scope by examining various functionalized aliphatic halides and alkenyl amides (Fig. 3). Primary alkyl iodides and bromides containing different functional groups underwent efficient hydroalkyl additions to **7a**, furnishing the desired branched compounds **9b**–**w** in 32–94% yield and high regioselectivities. Transformations with less activated organobromides were performed at 60 °C for optimal efficiency. These include products that contain an ester (**9d**), an enoate (**9e**), an aldehyde (**9h**), an alkene (**9n**) as well as unprotected protic units (problematic with basic organometallic reagents)^57,61^ such as carboxylic acid (**9k**), alcohol (**9p**–**q**) and phenol (**9r**). It merits mention that previous methods which rely on hydrosilane/base to generate the hydride source may cause undesired silylation side reactions with hydroxyl groups^46^. Both acid-labile (acetal **9o**) and base-labile (**9k**, **9r** and boronate **9t**) functionalities, which could be vulnerable under conditions that require acid/base additives^40–46^, as well as Lewis basic heterocyclic motifs (**9f**, **9u**–**v**) are tolerated. Among the products bearing a derivatizable halogen appendage (**9i**–**j**, **9s**), the reaction that afforded **9j** highlights the inherent chemoselectivity of an iodoalkane over an aryl iodide, although arene hydrodeiodination was detected as a side reaction. The reductive protocol is also compatible with substrates derived from complex multifunctional bioactive molecules such as base-sensitive sulbactam^62^ (**9l**) and indometacin (**9m**).

Secondary alkyl iodides and bromides also served as effective reagents under the standard conditions, delivering the expected products **9x**–**ab** in 51–73% yield and offering complementary scope to a previous report in which secondary haloalkanes were low-yielding^46^. However, organohalides that lack a C_β_–H bond and therefore incapable of generating the requisite nickel-hydride species by β-H elimination pose a challenge in our system. Attempts to carry out reductive hydroalkylation using neopentyl bromide (3 equiv.) as alkyl donor and isopropyl bromide (1.2 equiv.) as hydride donor^47,48^ gave the desired hydroalkylation adduct **9w** in 32% yield, along with side products derived from dialkylation with neopentyl bromide as well as reductive hydroalkylation with isopropyl bromide (see Supplementary Methods 2.7 for details). β,γ-Unsaturated amides with 1,2-disubstitiuted C=C bonds underwent reaction to form the corresponding β-alkylated products (**9ac**–**ag**), but those with 1,1-disubstituted and trisubstituted olefins were ineffective substrates (<5% conv. to desired product).

### Synthetic applications

**Fig. 4:**
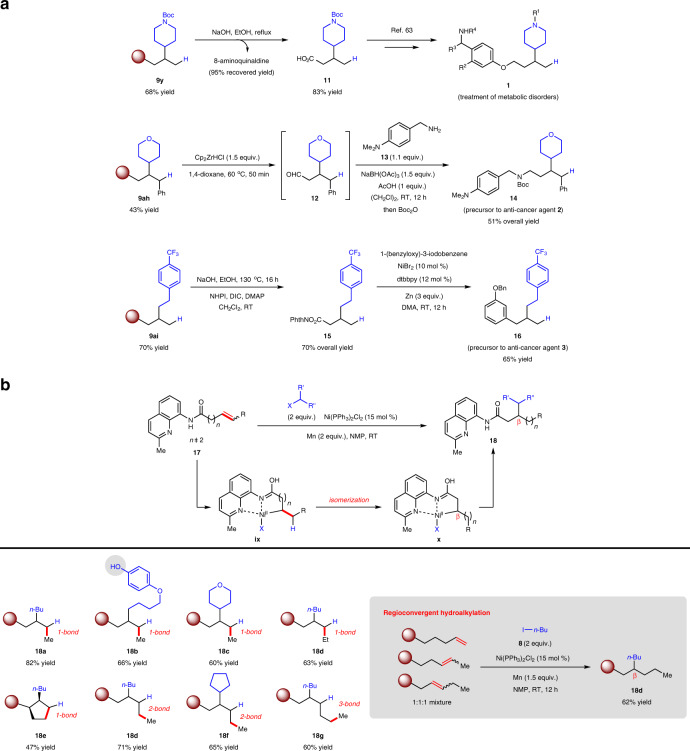
Application to synthesis of biologically active molecules and remote hydroalkylation. **a** The products resulting from reductive hydroalkylation can be conveniently transformed to a variety of medicinal compounds of interest. **b** Alkenyl amides bearing ≥ five-carbon chain length undergo remote hydroalkylation through in situ isomerization of alkylnickel intermediates, providing reliable access to β-alkylated molecules. Regioisomeric olefin mixtures can be regioconvergently converted to a single value-added product. Regioisomeric ratios were determined by ^1^H NMR analysis. Yields are for isolated and purified products. **9ah** was obtained as a 96:4 regioisomeric mixture. For **18a**, **18d**, **18e**, and **18g**, reactions were conducted using iodides (X = I). For **18b**, **18c**, and **18f**, reactions were conducted using bromides (X = Br). For **18g**, 5 equiv. of 1-iodobutane was used. R, functional group; X, halide; NMP, *N*-methyl-2-pyrrolidone; DMA, *N*,*N*-dimethylacetamide; NHPI, *N*-hydroxyphthalimide; DIC, *N*,*N*′-diisopropylcarbodiimide; DMAP, 4-dimethylaminopyridine; dtbbpy, 4,4′-di-*tert*-butyl-2,2′-bipyridine; Boc, *tert*-butoxycarbonyl; Phth, phthaloyl; Cp, cyclopentadienyl; RT, room temperature.

The first application that demonstrates utility of our Markovnikov-selective reductive hydroalkylation protocol involves the synthesis of a family of therapeutic compounds **1** for the treatment of metabolic disorders^63^ (Fig. 4a). Chemoselective removal of the amide directing group in **9y** (recovered 8-aminoquinaldine in 95% yield) afforded acid **11**, a key intermediate employed in the preparation of **1**, in 83% yield.

In a second instance, chemoselective reduction of the 8-aminoquinaldine amide in **9ah** to aldehyde **12** followed by reductive amination with benzylamine **13** and *N*-Boc protection delivered **14**, a precursor for the synthesis of an anti-cancer compound **2**^64^, in 51% overall yield. The entire sequence is more concise compared to a previous report^64^. The preparation of **16** highlights yet another compelling example of the versatility of the branched hydroalkylation products. Facile conversion of **9ai** to its redox-active ester derivative **15** (70% overall yield) set the stage for a catalytic decarboxylative cross-coupling^53^ with 1-(benzyloxy)-3-iodobenzene to furnish **16**, which has been further elaborated to another anti-cancer agent **3**^65^.

A corollary to the present reductive approach is the implementation of remote olefin hydroalkylation^66–70^, specifically with alkenyl amides **17** containing an extended hydrocarbon backbone (≥ five-carbon chain length). As showcased in Fig.4b, we postulated that the organonickel intermediate **ix** generated from nickel-hydride addition (cf. Fig. 2) could potentially isomerize to the relatively more stabilized five-membered nickelacycle **x**^66,67,71^ through consecutive β-H elimination/olefin insertion steps. Following a similar mechanism in Fig. 2, **x** could be converted to **18** with net remote alkylation at the β position. Although nickel-hydride-promoted remote hydrocarbonfunctionalizations have been disclosed, most instances involve functionalizations at either the sterically exposed terminus or the α-carbon site adjacent to an electron-stabilizing moiety^68,69^.

Gratifyingly, γ,δ-unsaturated amides bearing terminal and internal C=C bonds were found to participate in remote hydroalkylation with primary and secondary haloalkanes, affording products **18a**–**e** in 47–82% yield as single β regioisomers through single double-bond migrations (Fig. 4b). Cyclic olefins also underwent reaction as exemplified by **18e**, which was obtained as a single *syn* diastereomer^71^. Using the regioisomeric β,γ-unsaturated amide substrate leading to **18e** would be, however, less practical since the corresponding β,γ-unsaturated carboxylic acid is much more expensive. Transformations with δ,ε-unsaturated amides (involving two C=C bond isomerizations) were similarly efficient and site-selective (**18d** and **18f**). Remarkably, subjecting the 6-heptenoic acid-derived alkenyl amide to established conditions gave β-alkylated **18g** in 60% yield, underscoring the fidelity of the catalytic process that features an alkene transposition over three positions^67,68^. Furthermore, a streamlined synthesis of β-alkylated **18d** could be attained in 62% yield through regioconvergent hydroalkylation of an isomeric mixture of olefin substrates (Fig. 4b, gray inset).

### Mechanistic studies

**Fig. 5:**
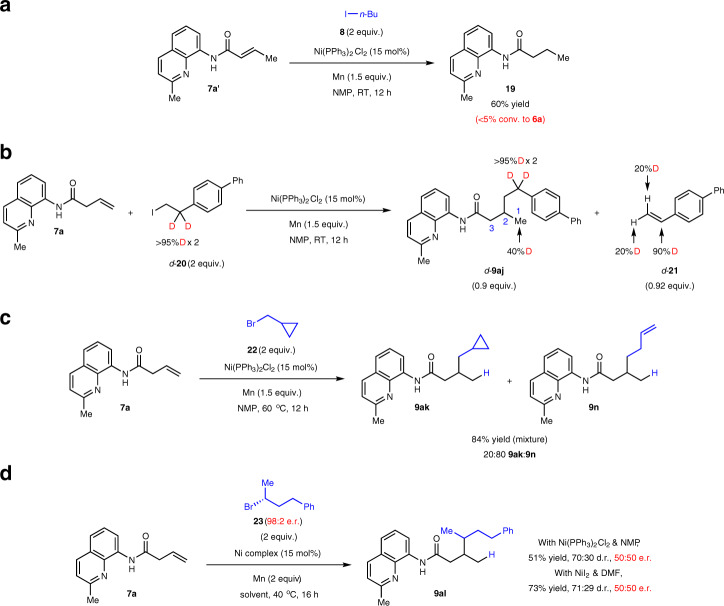
Mechanistic studies. **a** Control experiment ruling out the intermediacy of **7a′**. **b** Deuterium labeling experiment. **c** Radical clock experiment. **d** Complete stereochemical erosion with an enantioenriched alkyl halide. Diastereomeric ratios were determined by ^1^H NMR analysis. Enantiomeric ratios were determined by chiral HPLC analysis. Yields are for isolated and purified products. NMP, *N*-methyl-2-pyrrolidone; RT, room temperature.

Subjecting the α,β-unsaturated isomer of **7a** to the standard conditions only gave the fully hydrogenated product **19**, thereby ruling out the likelihood of olefin isomerization prior to the hydroalkylation event (Fig. 5a). To shed light on the importance of the alkyl halide partner in our catalytic system, we carried out deuterium labeling studies with **7a** in the presence of two equivalents of *d*-**20** under the standard reaction conditions (Fig. 5b). Accordingly, the hydroalkylation product *d*-**9aj** and olefin by-product *d*-**21** were isolated. Deuterium incorporation on C1 of *d*-**9aj** and deuterium scrambling in *d*-**21** suggest that a nickel-deuteride species was likely generated from reaction with *d*-**20** (i.e., **ii**→**iii** in Fig. 2). The reversible nature of β-H(D) elimination and re-insertion means that adventitious deuterium scrambling in *d*-**21** and formation of Ni–H cannot be avoided. Competitive addition of Ni–H and Ni–D across the olefin in **7a** eventually gave rise to *d*-**9aj** with 40% D incorporation at C1. Overall, these results support the haloalkane’s key role as a donor of both the hydride and alkyl group.

The radical nature of the reductive hydroalkylation reaction was substantiated through a radical clock experiment using (bromomethyl)cyclopropane **22** as an electrophile (Fig. 5c). A 20:80 mixture of **9ak** and the ring-ruptured product **9n** in 84% yield was detected, intimating that **22** likely reacts with the catalytic organonickel species (cf. **i**→**ii** and **v**→**vi** in Fig. 2) through a bromine abstraction/radical recombination process^59,60^ via a cyclopropylmethyl radical that is prone to ring opening. Further support for the intermediacy of radicals could be obtained from the corresponding reactions using enantioenriched **23**, in which the desired product **9al** was generated in 51–73% yield and ~70:30 d.r. as a racemic mixture (Fig. 5d).

In summary, we demonstrated that Ni-catalyzed branched-selective hydroalkyl additions to unactivated alkenyl amides can be achieved by using haloalkanes and manganese as reductant. With long-chain olefins, remote and regioconvergent hydroalkylations proceed to furnish products with reliable β selectivities. Mechanistic experiments corroborate the aliphatic halide’s dual role as a hydride and alkyl donor. Equally crucial is the 8-aminoquinaldine tether that effectively suppresses any adventitious dialkylation side reaction. The robust conditions are compatible with diverse functional groups, including those that are sensitive to hydrosilane, acidic or basic additives. In conjunction with existing methods, we expect our catalytic strategy to find significant utility in chemical synthesis.

## Methods

### General reductive hydroalkylation procedure

In a N_2_-filled glovebox, to an oven-dried 5 mL vial equipped with a magnetic stir bar were added alkene substrate (0.1 mmol), alkyl iodide or bromide (if solid, added at this time) (0.2 mmol), Ni(PPh_3_)_2_Cl_2_ (9.8 mg, 0.015 mmol) and Mn powder (0.15 mmol). The mixture was then dissolved in 0.3 mL dry NMP. The vial was tightly capped and removed from the glovebox. The alkyl iodide or bromide (if liquid, added at this time) was added by a micro-syringe. The mixture was allowed to vigorously stir at ambient temperature (for alkyl iodide) or 60 °C (for alkyl bromide) for 12–20 h. When alkene was almost fully consumed (monitored by TLC), the mixture was directly subjected to flash silica gel column chromatography to afford the pure product.

## Supplementary information

Supplementary Information

## Data Availability

All data are available from the corresponding authors upon reasonable request.
