# Supplementary material for: Alkyl halides as both hydride and alkyl sources in catalytic regioselective reductive olefin hydroalkylation
Source: Nat Commun. 2020 Nov 17;11:5857. doi: 10.1038/s41467-020-19717-6 (PMC7673021; doi:10.1038/s41467-020-19717-6)
Supplement: Supplementary file 1 — Supplementary Information [file 41467_2020_19717_MOESM1_ESM.pdf]

Supplementary Information
for
Alkyl Halides as Both Hydride and Alkyl Sources in Catalytic Regioselective Reductive Olefin Hydroalkylation

Xianxiao Chen[1,2], Weidong Rao[1], Tao Yang[2]* & Ming Joo Koh[2]*

[1]Jiangsu Provincial Key Lab for the Chemistry and Utilization of Ago-Forest Biomass, College of Chemical Engineering, Nanjing Forestry University, Nanjing, 210037, China.
[2]Department of Chemistry, National University of Singapore, 12 Science Drive 2, Republic of Singapore, 117549.
*Corresponding authors. Emails: chmkmj@nus.edu.sg; chmyt@nus.edu.sg.

Table of Contents

1. Supplementary Note 1 3

2. Supplementary Methods, Tables and Figures 4

2.1 Optimization of Reaction Conditions 4

2.2 Synthesis of Alkene Substrates 6

2.3 Synthesis and Characterization of Products 9

2.4 Application to Synthesis of Biologically Active Molecules 25

2.5 Mechanistic Studies 29

2.6 Attempted Hydroalkylation in the Presence of Hydrosilane/Base 35

2.7 Attempted Hydroalkylation Using Substrates Without C_β-H Bonds 36

2.8. NMR Spectra 38

3. Supplementary References 109

1. Supplementary Note 1

All commercial chemicals were used without additional purification, unless otherwise stated. Anhydrous solvent was purchased from commercial sources and transferred under argon atmosphere. NMR spectra were recorded on Bruker DPX 400 or Bruker DPX 500 spectrometer. Chemical shifts are reported in ppm from tetramethylsilane with the solvent resonance resulting from incomplete deuterium incorporation as the internal standard ($CDCl_3$: *δ* 7.26 ppm). Data are reported as follows: chemical shift, integration, multiplicity (s = singlet, d = doublet, t = triplet, q = quartet, m = multiplet) and coupling constants (Hz). ^{13}C NMR spectra were recorded on a Bruker DPX 400 or Bruker DPX 500spectrometer with complete proton decoupling. Chemical shifts are reported in ppm from tetramethylsilane with the solvent resonance as the internal standard ($CDCl_3$: *δ* 77.16 ppm). High-resolution mass spectrometric data were obtained using Bruker Apex IV RTMS. GC-MS analysis was performed on Shimadzu GC-2010 gas chromatography coupled to a Shimadzu QP2010 mass selective detector. Column conditions are reported in the experimental section below. Values for regioisomeric ratio of products were determined by GC and ^{1}H NMR. Solvents (acetonitrile, CH_2Cl_2, diethyl ether, tetrahydrofuran and toluene) were purified under a positive pressure of dry nitrogen gas by a modified innovative technologies purification system. *N,N*-Dimethylacetamide (anhydrous), *N*,*N*-dimethylformamide (anhydrous), *N*-methyl-2-pyrrolidone (anhydrous), *N*,*N'*-dimethylpropyleneurea (anhydrous) and dimethyl sulfoxide (anhydrous) were used as received. Flash chromatography was performed using Merck silica gel 60 (0.040-0.063 mm) or SiliCycle silica gel F60 (0.040-0.063 mm). All purification procedures of products were carried out with reagent grade solvents.

2. Supplementary Methods, Tables and Figures

2.1 Optimization of Reaction Conditions

General procedure for condition optimization: In a N_2-filled glovebox, to an oven-dried 5 mL vial equipped with a magnetic stir bar were added alkene substrate **7a** (22.6 mg, 0.1 mmol), $Ni(PPh_3)_2Cl_2$ (9.8 mg, 0.015 mmol), Mn powder (8.3 mg, 0.15 mmol) and dry NMP (0.3 mL) sequentially. The vial was tightly capped and removed out of the glovebox followed by the addition of 1-iodobutane **8** (36.8 mg, 0.2 mmol) via a micro-syringe. The mixture was allowed to vigorously stir at ambient temperature for 12 h. After that, 20 mg *n*-tridecane was added as an internal standard. 3 µL of the reaction mixture was taken out and subjected to GC analysis to determine the conversion of alkene **7a** as well as the calibrated GC yields of products **9a** and **10a**.

Supplementary Table 1 Effect of Nickel Catalyst[a]

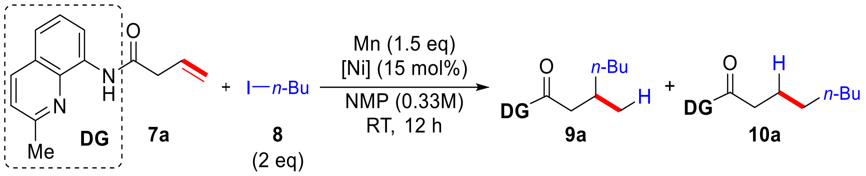

Entry	[Ni]	Conversion of **7a** (%)	Yield (%)	Ratio of **9a**:**10a**
1	$NiCl_2$	77	56	93:7
2	$NiCl_2DME$	>99	74	>95:5
3	$NiCl_2(Py)_4$	70	50	92:8
4	***$NiCl_2(PPh_3)_2$***	***>99***	***90***	***>95:5***
5	NiI_2	>99	63	>95:5
6	$Ni(COD)_2$	80	68	>95:5

[a]Conversion, yields and regioisomeric ratios (**9a**:**10a**) were determined by GC analysis with *n*-tridecane as internal standard.

Supplementary Table 2 Effect of Solvents[a]

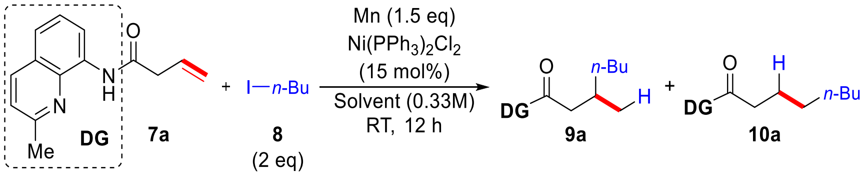

Entry	Solvents	Conversion of **7a** (%)	Yield (%)	Ratio of **9a**:**10a**
1	***NMP***	***>99***	***90***	***>95:5***
2	DMF	>99	86	92:8
3	DMA	>99	88	93:7
4	DMSO	<2	trace	ND
5	DMPU	>99	92	94:6
6	ACN	88	70	94:6
7	THF	40	10	>95:5
8	PhMe	<2	trace	ND

[a]Conversion, yields and regioisomeric ratios (**9a**:**10a**) were determined by GC analysis with *n*-tridecane as internal standard. ND, not determined.

Supplementary Table 3 Effect of Directing Groups[a]

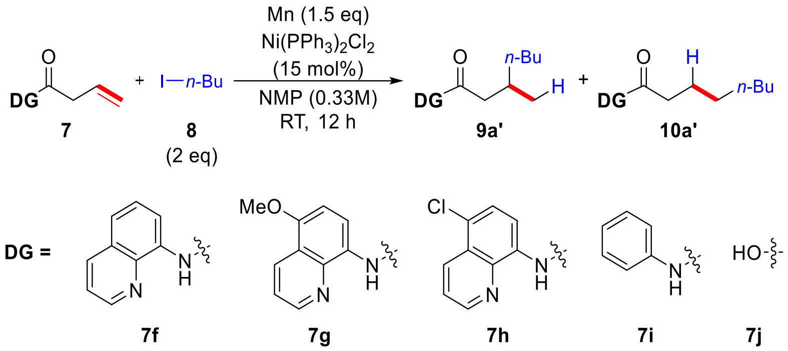

Entry	DG	Conversion of **7** (%)	Yield (%)	Ratio of **9a’**:**10a’**
1	***7a***	***>99***	***90***	***>95:5***
2	**7f**	60	40	75:25
3	**7g**	90	60	70:30
4	**7h**	90	65	45:55
5	**7i**	<2	trace	ND
6	**7j**	<2	trace	ND

[a]Conversion, yields and regioisomeric ratios (**9a’**:**10a’**) were determined by GC analysis with *n*-tridecane as internal standard. ND, not determined.

Supplementary Table 4 Effect of Other Conditions[a]

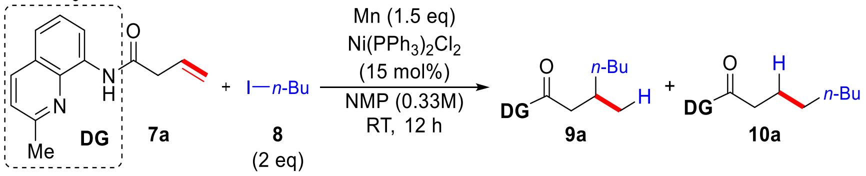

Entry	Deviation from Standard Conditions	Conversion of **7a** (%)	Yield (%)	Ratio of **9a**:**10a**
1	***None***	***>99***	***90***	***>95:5***
2	No Mn	<2	trace	ND
3	Mn (1 equiv.) instead of Mn (1.5 equiv.)	>99	70	>95:5
4	Mn (2 equiv.) instead of Mn (1.5 equiv.)	>99	90	>95:5
5	Zn instead of Mn	66	44	80:20
6	**8** (1.5 equiv.) instead of **8** (2 equiv.)	80	70	>95:5
7	**8** (2.5 equiv.) instead of **8** (2 equiv.)	>99	91	>95:5
8	40 °C instead of RT	>99	90	>95:5
9	10 mol% Nickel catalyst instead of 15 mol%	<2	92	92:8

[a]Conversion, yields and regioisomeric ratios (**9a**:**10a**) were determined by GC analysis with *n*-tridecane as internal standard.

Supplementary Table 5 Optimization of Reaction with Alkyl Bromide[a]

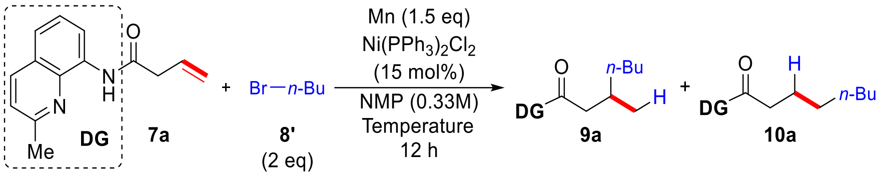

Entry	Temperature	Conversion of **7a** (%)	Yield (%)	Ratio of **9a**:**10a**
1	RT	>99	84	86:14
2	40 °C	>99	83	90:10
3	***60 °C***	***>99***	***83***	***>95:5***
4	80 °C	>99	81	>95:5

[a]Conversion, yields and regioisomeric ratios (**9a**:**10a**) were determined by GC analysis with *n*-tridecane as internal standard.

2.2 Synthesis of Alkene Substrates

General procedure for the synthesis of alkene substrates: According to the corresponding literature procedures[1,2], aryl amine (1.44 g, 10 mmol), EDCI (2.30 g, 12 mmol) and DMAP (122 mg, 1 mmol) were added sequentially in a 100 mL round-bottomed flask containing 30 mL DCM. Then enoic acid (11 mmol) was added dropwise and the reaction mixture was allowed to stir at ambient temperature for 16 h. The resulting mixture was washed with saturated aqueous $NaHCO_3$ solution, extracted with DCM and the combined organic layers were washed with brine, dried over $MgSO_4$ and concentrated under reduced pressure. The residue was purified by flash column chromatography (eluent: 10:1 hexanes:EtOAc) to afford alkene substrates.

***N*-(2-Methylquinolin-8-yl)but-3-enamide (7a):**

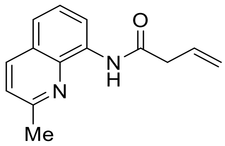

A colorless solid, 85% yield. **^{1}H NMR** (500 MHz, $CDCl_3$) δ 10.07 (s, 1H), 8.72 (dd, J = 5.6, 3.4 Hz, 1H), 8.01 (d, J = 8.3 Hz, 1H), 7.47 – 7.43 (m, 2H), 7.30 (d, J = 8.4 Hz, 1H), 6.16 (ddt, J = 17.3, 10.2, 7.1 Hz, 1H), 5.42 (dq, J = 10.1, 1.4 Hz, 1H), 5.40 – 5.38 (m, 1H),3.35 (d, J = 7.2 Hz, 2H), 2.72 (s, 3H). **^{13}C NMR** (100 MHz, $CDCl_3$) δ 169.1, 157.2, 137.9, 136.4, 133.8, 131.3, 126.3, 126.1, 122.4, 121.4, 120.1, 116.4, 43.2, 25.3. **HRMS (ESI)**: Calcd. 249.0998 for $C_{14}H_{14}N_2NaO$ $(M+Na)^+$, found 249.1003.

(*E*)-*N*-(2-Methylquinolin-8-yl)pent-3-enamide (7b):

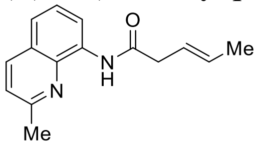

A colorless solid, 78% yield. **^{1}H NMR** (500 MHz, $CDCl_3$) δ 10.14 (s, 1H), 8.71 (dd, J = 7.2, 1.8 Hz, 1H), 7.94 (d, J = 8.4 Hz, 1H), 7.42 – 7.37 (m, 2H), 7.24 (d, J = 8.4 Hz, 1H), 5.87 – 5.80 (m, 1H), 5.78 – 5.72 (m, 1H), 3.24 (d, J = 6.9 Hz, 2H), 2.68 (s, 3H), 1.85 (dd, J = 6.2, 1.3 Hz, 3H). **^{13}C NMR** (125 MHz, $CDCl_3$) δ 169.9, 157.0, 137.8, 136.3, 133.8, 131.8, 126.3, 126.0, 123.7, 122.3, 121.2, 116.2, 42.1, 25.2, 18.2. **HRMS (ESI)**: Calcd. 263.1157 for $C_{15}H_{16}N_2NaO$ $(M+Na)^+$, found 263.1155.

(*E*)-*N*-(2-Methylquinolin-8-yl)hex-3-enamide (7c):

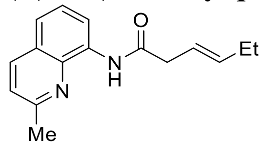

A colorless solid, 70% yield. **^{1}H NMR** (400 MHz, $CDCl_3$) δ 10.14 (s, 1H), 8.74 (dd, *J* = 5.8, 3.2 Hz, 1H), 8.03 (d, *J* = 8.4 Hz, 1H), 7.48 - 7.43 (m, 2H), 7.31 (d, *J* = 8.4 Hz, 1H), 5.94 – 5.86 (m, 1H), 5.76 (dtt, *J* = 15.5, 7.1, 1.5 Hz, 1H), 3.28 (d,*J* = 7.0 Hz, 2H), 2.72 (s, 3H), 2.22 (qdd, *J* = 7.4, 6.2, 1.3 Hz, 2H), 1.12 (t, *J* = 7.5 Hz, 3H). **^{13}C NMR** (100 MHz, $CDCl_3$) δ 170.2, 157.2, 138.9, 138.0, 136.5, 134.0, 126.5, 126.2, 122.4, 121.4, 121.4, 116.4, 42.3, 25.8, 25.4, 13.6. **HRMS** (**ESI**): Calcd. 277.1311 for $C_{16}H_{18}N_2NaO$ $(M+Na)^+$, found 277.1312.

(*E*)-*N*-(2-Methylquinolin-8-yl)-5-phenylpent-3-enamide (7d):

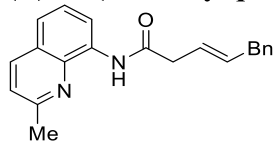

A colorless oil, 73% yield. **^{1}H NMR** (400 MHz, $CDCl_3$) δ 10.15 (s, 1H), 8.77 (td, *J* = 4.2, 3.8, 1.9 Hz, 1H), 8.05 (d, *J* = 8.3 Hz, 1H), 7.51 – 7.48 (m, 2H), 7.35 – 7.21 (m, 6H), 6.03 (ddd, *J* = 14.7, 7.1, 5.8 Hz, 1H), 5.93 – 5.85 (m, 1H), 3.57 (d, *J* = 6.5 Hz, 2H), 3.36 (d, *J* = 7.0 Hz, 2H), 2.73 (s, 3H). **^{13}C NMR** (100 MHz, $CDCl_3$) δ 169.7, 157.2, 140.0, 138.0, 136.5, 135.3, 133.9, 128.7, 128.6, 126.4, 126.3, 126.2, 124.0, 122.5, 121.4, 116.5, 42.1, 39.2, 25.4. **HRMS** (**ESI**): Calcd. 339.1468 for$C_{21}H_{20}N_2NaO$ $(M+Na)^+$, found 339.1474.

(*E*)-*N*-(2-Methylquinolin-8-yl)-4-phenylbut-3-enamide (7e):

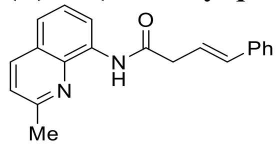

A colorless solid, 77% yield. **^{1}H NMR** (500 MHz, $CDCl_3$) δ 10.27 (s, 1H), 8.76 (d, *J* = 6.9 Hz, 1H), 8.00 (d, *J* = 9.0 Hz, 1H), 7.53 – 7.44 (m, 4H), 7.38 (dd, *J* = 8.5, 6.8 Hz, 2H), 7.32 – 7.25 (m, 2H), 6.77 (d, *J* = 15.8 Hz, 1H), 6.55 (dt, *J* = 15.6, 7.4 Hz, 1H), 3.52 (d, *J* = 7.5 Hz, 2H), 2.49 (s, 3H). **^{13}C NMR** (125 MHz, $CDCl_3$) δ 169.2, 157.4, 137.9, 136.9, 136.4, 135.7, 133.8, 128.7, 127.9, 126.6, 126.4, 126.1, 122.5, 122.2, 121.5, 116.3, 42.4, 25.0. **HRMS** (**ESI**): Calcd. 325.1311 for $C_{20}H_{18}N_2NaO$ $(M+Na)^+$, found 325.1315.

***N*-(2-Methylquinolin-8-yl)pent-4-enamide (17a):**

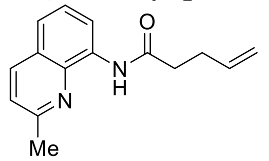

A colorless oil, 81% yield. **^{1}H NMR** (400 MHz, $CDCl_3$) δ 9.89 (s, 1H), 8.77 (dd, *J* = 6.7, 2.4 Hz, 1H), 8.02 – 7.99 (m, 1H), 7.48 – 7.42 (m, 2H), 7.31 – 7.28 (m, 1H), 5.97 (ddt, *J* = 16.6, 10.1, 6.3 Hz, 1H), 5.19 (dq, *J* = 17.1, 1.7 Hz, 1H), 5.08 (dq, *J* = 10.2, 1.4 Hz, 1H), 2.74 (s, 3H), 2.68 (dd, *J* = 7.9, 5.9 Hz, 2H), 2.60 (dtd, *J* = 8.1, 6.4, 1.5 Hz, 2H). **^{13}C NMR** (100 MHz, $CDCl_3$) δ 170.9, 157.2, 137.7, 137.0, 136.4, 133.9, 126.3, 126.1, 122.4, 121.3, 116.4, 115.8, 37.3, 29.5, 25.3. **HRMS** (**ESI**): Calcd. 263.1155 for

$C_{15}H_{16}N_2NaO$ (M+Na)$^+$, found 263.1155.

(*E*)-*N*-(2-Methylquinolin-8-yl)hex-4-enamide (17b):

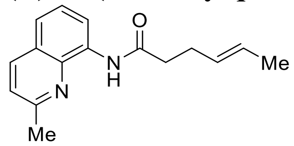

A colorless oil, 69% yield. **^{1}H NMR** (500 MHz, $CDCl_3$) δ 9.85 (s, 1H), 8.74 (dd, *J* = 6.9, 2.1 Hz, 1H), 7.99 (d, *J* = 8.4 Hz, 1H), 7.45 – 7.40 (m, 2H), 7.28 (d, *J* = 8.4 Hz, 1H), 5.62 – 5.51 (m, 2H), 2.72 (s, 3H), 2.61 (t, *J* = 7.2 Hz, 2H), 2.52 – 2.48 (m, 2H), 1.65 (d, *J* = 4.5 Hz, 3H). **^{13}C NMR** (125 MHz, $CDCl_3$) δ 171.2, 157.2, 137.8, 136.5, 134.0, 129.5, 126.4, 126.4, 126.1, 122.4, 121.2, 116.5, 38.1, 28.5, 25.3, 18.0. **HRMS** (**ESI**): Calcd. 277.1311 for $C_{16}H_{18}N_2NaO$ (M+Na)$^+$, found 277.1312.

***N*-(2-Methylquinolin-8-yl)cyclopent-3-ene-1-carboxamide (17c):**

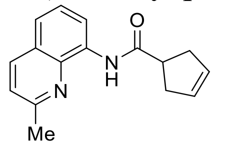

A colorless solid, 79% yield. **^{1}H NMR** (400 MHz, $CDCl_3$) δ9.99 (s, 1H), 8.75 (dd, *J* = 6.7, 2.3 Hz, 1H), 8.01 (d, *J* = 8.4 Hz, 1H), 7.47 – 7.41 (m, 2H), 7.30 (d, *J* = 8.4 Hz, 1H), 5.78 (s, 2H), 3.40 – 3.32 (m, 1H), 2.90 – 2.79 (m, 4H), 2.73 (s, 3H). **^{13}C NMR** (100 MHz, $CDCl_3$) *δ* 174.7, 157.2, 137.9, 136.5, 134.2, 129.4, 126.5, 126.1, 122.4, 121.2, 116.5, 45.2, 37.2, 25.4. **HRMS** (**ESI**): Calcd. 275.1155 for $C_{16}H_{16}N_2NaO$ (M+Na)$^+$, found 275.1165.

***N*-(2-Methylquinolin-8-yl)hex-5-enamide (17d):**

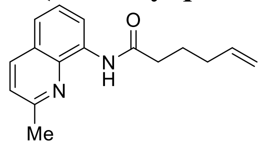

A colorless oil, 75% yield. **^{1}H NMR** (500 MHz, $CDCl_3$) δ 9.87 (s, 1H), 8.77 (dd, *J* = 7.0, 2.0 Hz, 1H), 8.00 (d, *J* = 8.4 Hz, 1H), 7.47 – 7.42 (m, 2H), 7.29 (d, *J* = 8.4 Hz, 1H), 5.87 (ddt, *J* = 17.0, 10.2, 6.7 Hz, 1H), 5.12 (ddd, *J* = 17.1, 3.5, 1.6 Hz, 1H), 5.05 (ddt, *J* = 10.2, 2.1, 1.2 Hz, 1H), 2.74 (s, 3H), 2.60 – 2.57 (m, 2H), 2.25 – 2.21 (m, 2H), 1.99 – 1.92 (m, 2H). **^{13}C NMR** (125 MHz, $CDCl_3$) *δ*171.5, 157.2, 137.9, 137.7, 136.5, 133.9, 126.4, 126.1, 122.4, 121.2, 116.4, 115.5, 37.3, 33.2, 25.3, 24.7. **HRMS** (**ESI**): Calcd. 277.1311 for$C_{16}H_{18}N_2NaO$ (M+Na)$^+$, found 277.1320.

N-(2-Methylquinolin-8-yl)hept-6-enamide (17e):

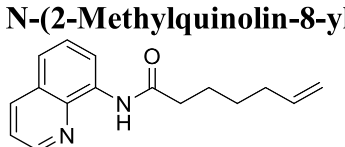

A colorless oil, 84% yield. **^{1}H NMR** (400 MHz, $CDCl_3$) δ 9.86 (s, 1H), 8.74 (dd, *J* = 6.7, 2.3 Hz, 1H), 8.00 (d, *J* = 8.4 Hz, 1H), 7.46 – 7.40 (m, 2H), 7.28 (d, *J* = 8.4 Hz, 1H), 5.83 (ddt, *J* = 16.9, 10.2, 6.7 Hz, 1H), 5.04 (dq, *J* = 17.1, 1.6 Hz, 1H), 4.97 (ddt, *J* = 10.2, 2.2, 1.2 Hz, 1H), 2.73 (s, 3H), 2.56 (t, *J* = 7.5 Hz, 2H), 2.17 – 2.11 (m, 2H), 1.84 (tt, *J* = 8.3, 6.5 Hz, 2H), 1.55 (tt, *J* = 9.8, 6.5 Hz, 2H). **^{13}C NMR** (100 MHz, $CDCl_3$) δ 171.6, 157.2, 138.6, 137.7, 136.5, 134.0, 126.4, 126.1, 122.4, 121.2, 116.4, 114.8, 38.1,

33.6, 28.6, 25.3, 25.2. **HRMS** (**ESI**): Calcd. 291.1468 for $C_{17}H_{20}N_2NaO$ (M+Na)$^+$, found 291.1470.

***N*-(5-Methoxyquinolin-8-yl)but-3-enamide (7g)[3]:**

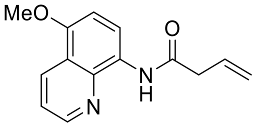

A colorless solid, 81% yield. **^{1}H NMR** (400 MHz, $CDCl_3$) δ 9.71 (s, 1H), 8.80 (dd, *J* = 4.3, 1.8 Hz, 1H), 8.68 (d, *J* = 8.5 Hz, 1H), 8.56 (dd, *J* = 8.4, 1.7 Hz, 1H), 7.42 (dd, *J* = 8.4, 4.2 Hz, 1H), 6.82 (d, *J* = 8.5 Hz, 1H), 6.14 (ddt, *J* = 17.2, 10.1, 7.1 Hz, 1H), 5.38 (dq, *J* = 10.4, 1.4 Hz, 1H), 5.37 – 5.34 (m, 1H), 3.98 (s, 3H), 3.33 (dt, *J* = 7.1, 1.3 Hz, 2H). **^{13}C NMR** (100 MHz, $CDCl_3$) δ 169.0, 150.4, 148.8, 139.3, 131.4, 131.3, 127.9, 120.8, 120.5, 120.0, 116.8, 104.5, 55.9, 43.2.

***N*-(5-Chloroquinolin-8-yl)but-3-enamide (7h):**

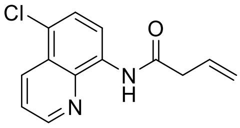

A colorless solid, 75% yield. **^{1}H NMR** (400 MHz, $CDCl_3$) δ 9.92 (s, 1H), 8.84 (dd, *J* = 4.2, 1.6 Hz, 1H), 8.71 (d, *J* = 8.4 Hz, 1H), 8.56 (dd, *J* = 8.4, 1.5 Hz, 1H), 7.59 – 7.55 (m, 2H), 6.20 – 6.06 (m, 1H), 5.42 – 5.39 (m, 1H), 5.37 (t, *J* = 1.3 Hz, 1H), 3.35 (dt, *J* = 7.1, 1.3 Hz, 2H). **^{13}C NMR** (100 MHz, $CDCl_3$) δ 169.4, 148.8, 139.1, 133.8, 133.6, 130.9, 127.4, 126.1, 124.5, 122.4, 120.4, 116.6, 43.3. **HRMS** (**ESI**): Calcd. 269.0452 for $C_{13}H_{11}ClN_2ONa$ (M+Na)$^+$, found 269.0456.

2.3 Synthesis and Characterization of Products

General procedure for nickel-catalyzed reductive hydroalkylation of alkenes

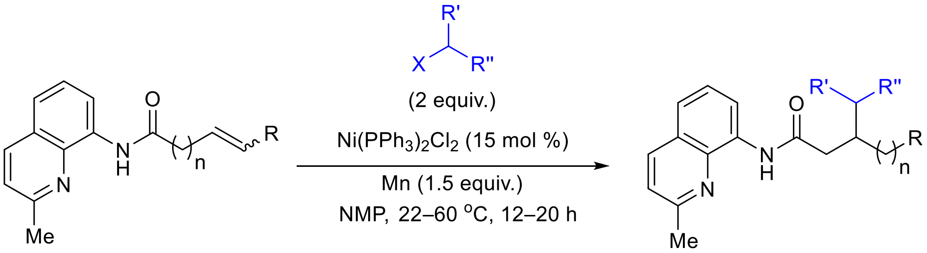

In a N_2-filled glovebox, to an oven-dried 5 mL vial equipped with a magnetic stir bar were added alkene substrate (0.1 mmol), alkyl iodide or bromide (if solid, added at this time) (0.2 mmol), $Ni(PPh_3)_2Cl_2$ (9.8 mg, 0.015 mmol) and Mn powder (0.15 mmol). The mixture was then dissolved in dry NMP (0.3 mL). The vial was tightly capped and removed from the glovebox. The alkyl iodide or bromide (if liquid, added at this time) was added by a micro-syringe. The mixture was allowed to vigorously stir at ambient temperature (for alkyl iodide) or 60 °C (for alkyl bromide) for 12 – 20 h. When alkene was almost fully consumed (monitored by TLC), the mixture was directly subjected to flash silica gel column chromatography to afford the pure product.

3-Methyl-*N*-(2-methylquinolin-8-yl)heptanamide (9a):

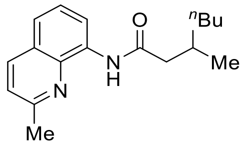

The title compound was prepared from **7a** (23 mg, 0.1 mmol) and 1-iodobutane (37 mg, 0.2 mmol) according to the general procedure. Purification using flash silica gel column chromatography (eluent: 15:1 hexanes:EtOAc) gave the pure product as acolorless oil (25 mg, 88% yield). **^{1}H NMR** (500 MHz, $CDCl_3$) δ 9.84 (s, 1H), 8.76 (dd, *J* = 7.2, 1.8 Hz, 1H), 7.97 (d, *J* = 8.2 Hz, 1H), 7.45 – 7.38 (m, 2H), 7.26 (d, *J* = 8.3 Hz, 1H), 2.72 (s, 3H), 2.55 (dd, *J* = 14.2, 6.1 Hz, 1H), 2.33 (dd, *J* = 14.2, 8.1 Hz, 1H), 2.21 – 2.11 (m, 1H), 1.49 – 1.24 (m, 6H), 1.05 (d, *J* = 6.7 Hz, 3H), 0.89 (t, *J* = 7.0 Hz, 3H). **^{13}C NMR** (125 MHz, $CDCl_3$) δ 171.4, 157.1, 137.7, 136.4, 134.0, 126.4, 126.1, 122.4, 121.1, 116.4, 46.0, 36.6, 30.9, 29.3, 25.3, 22.9, 19.9, 14.2. **HRMS** (**ESI**): Calcd. 307.1781 for $C_{18}H_{24}N_2NaO$ $(M+Na)^+$, found 307.1789.

General procedure for reaction on 2 mmol scale: In a N_2-filled glovebox, to an oven-dried 5 mL vial equipped with a magnetic stir bar were added alkene **7a** (2.0 mmol), $Ni(PPh_3)_2Cl_2$ (198 mg, 0.3 mmol) and Mn powder (4.0 mmol). The mixture was then dissolved in dry NMP (6.0 mL). The vial was tightly capped and removed from the glovebox. 1-Iodobutane (4.0 mmol) was then added by a micro-syringe. The mixture was allowed to vigorously stir at ambient temperature for 16 h, after which the mixture was diluted with EtOAc (20 mL) and washed with H_2O (15 mL). The mixture was filtered over Celite and rinsed with EtOAc. The organic phase was collected, dried over anhydrous Na_2SO_4, evaporated and purified by flash silica gel chromatography to afford the pure product **9a** (81% yield, 460 mg).

3-Methyl-*N*-(2-methylquinolin-8-yl)pentanamide (9b):

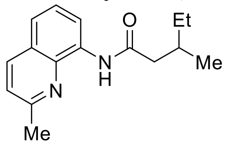

The title compound was prepared from **7a** (23 mg, 0.1 mmol) and iodoethane (31 mg, 0.2 mmol) according to the general procedure. Purification using flash silica gel column chromatography (eluent: 15:1 hexanes:EtOAc) gave the pure product as a colorless oil (24 mg, 93% yield). **^{1}H NMR** (300 MHz, $CDCl_3$) δ 9.86 (s, 1H), 8.75 (dd, *J* = 6.0, 3.1 Hz, 1H), 8.03 (d, *J* = 8.4 Hz, 1H), 7.52 – 7.38 (m, 2H), 7.31 (d, *J* = 8.4 Hz, 1H), 2.75 (s, 3H), 2.57 (dd, *J* = 14.2, 6.3 Hz, 1H), 2.35 (dd, *J* = 14.2, 8.0 Hz, 1H), 2.10 (dq, *J* = 13.7, 6.8 Hz, 1H), 1.51 (tq, *J* = 13.0, 7.4, 6.5 Hz, 1H), 1.33 (dt, *J* = 13.8, 7.2 Hz, 1H), 1.05 (d, *J* = 6.7 Hz, 3H), 0.97 (t, *J* = 7.4 Hz, 3H). **^{13}C NMR** (75 MHz, $CDCl_3$) δ 171.6, 157.3, 137.8, 136.6, 134.1, 126.5, 126.2, 122.5, 121.3, 116.5, 45.7, 32.6, 29.6, 25.4, 19.5, 11.6. **HRMS** (**ESI**): Calcd. 279.1468 for $C_{16}H_{20}N_2NaO$ $(M+Na)^+$, found 279.1475.

3-Methyl-*N*-(2-methylquinolin-8-yl)-6-phenylhexanamide (9c):

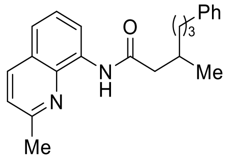

The title compound was prepared from **7a** (23 mg, 0.1 mmol) and (3-iodopropyl)benzene (49 mg, 0.2 mmol) according to the general procedure. Purification using flash silica gel column chromatography (eluent: 15:1 hexanes:EtOAc) gave the

pure product as a colorless oil (31 mg, 89% yield). ^{1}**H NMR** (500 MHz, $CDCl_3$) δ 9.88 (s, 1H), 8.80 (dd, *J* = 6.6, 2.4 Hz, 1H), 8.06 (d, *J* = 8.4 Hz, 1H), 7.51 – 7.46 (m, 2H), 7.34 (d, *J* = 8.4 Hz, 1H), 7.30 – 7.27 (m, 2H), 7.22 – 7.17 (m, 3H), 2.77 (s, 3H), 2.70 – 2.57 (m, 3H), 2.39 (dd, *J* = 14.2, 8.1 Hz, 1H), 2.26 (ddt, *J* = 14.4, 8.1, 6.5 Hz, 1H), 1.82 – 1.69 (m, 2H), 1.57 (ddt, *J* = 13.3, 10.7, 5.5 Hz, 1H), 1.39 (dddd, *J* = 13.3, 10.4, 7.9, 5.4 Hz, 1H), 1.10 (d, *J* = 6.7 Hz, 3H). ^{13}**C NMR** (100 MHz, $CDCl_3$) δ 171.3, 157.3, 142.7, 137.8, 136.6, 134.0, 128.5, 128.4, 126.5, 126.2, 125.8, 122.5, 121.3, 116.5, 46.0 36.6, 36.2, 30.9, 29.1, 25.4, 19.9. **HRMS** (**ESI**): Calcd. 369.1942 for $C_{23}H_{26}N_2NaO$ $(M+Na)^+$, found 369.1937.

4-Methoxyphenyl 5-methyl-7-((2-methylquinolin-8-yl)amino)-7-oxoheptanoate (9d):

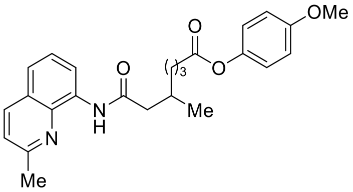

The title compound was prepared from **7a** (23 mg, 0.1 mmol) and 4-methoxyphenyl 4-iodobutanoate (64 mg, 0.2 mmol) according to the general procedure. Purification using flash silica gel column chromatography (eluent: 10:1 hexanes:EtOAc) gave the pure product as a colorless oil (31 mg, 73% yield). ^{1}**H NMR** (400 MHz, $CDCl_3$) δ 9.86 (s, 1H), 8.76 (dd, *J* = 6.0, 3.0 Hz, 1H), 8.03 (d, *J* = 8.4 Hz, 1H), 7.46 – 7.45 (m, 2H), 7.31 (d, *J* = 8.4 Hz, 1H), 6.97 (d, *J* = 9.1 Hz, 2H), 6.85 (d, *J* = 9.1 Hz, 2H), 3.78 (s, 3H), 2.74 (s, 3H), 2.61 – 2.54 (m, 3H), 2.41 (dd, *J* = 14.3, 7.8 Hz, 1H), 2.31 – 2.18 (m, 1H), 1.93 – 1.76 (m, 2H), 1.64 – 1.56 (m, *J* = 13.4, 10.9, 5.5 Hz, 1H), 1.47 – 1.39 (m, 1H), 1.11 (d, *J* = 6.7 Hz, 3H). ^{13}**C NMR** (100 MHz, $CDCl_3$) δ 172.6, 171.1, 157.3, 157.3, 144.3, 137.8, 136.5, 134.0, 126.5, 126.2, 122.5, 122.4, 121.3, 116.5, 114.5, 55.7, 45.8, 36.2, 34.5, 30.7, 25.4, 22.6, 19.9. **HRMS** (**ESI**): Calcd. 443.1941 for $C_{25}H_{28}N_2NaO_4$ $(M+Na)^+$, found 443.1943.

3-Methyl-*N*-(2-methylquinolin-8-yl)-7-((2-oxo-2*H*-chromen-7-yl)oxy)heptanam ide (9e):

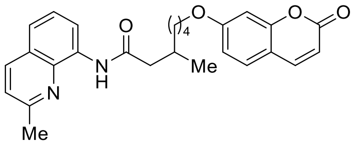

The title compound was prepared from **7a** (23 mg, 0.1 mmol) and 7-(4-iodobutoxy)-2H-chromen-2-one (69 mg, 0.2 mmol) according to the general procedure. Purification using flash silica gel column chromatography (eluent: 10:1 hexanes:EtOAc) gave the pure product as a colorless oil (40 mg, 90% yield). ^{1}**H NMR** (500 MHz, $CDCl_3$) δ 9.85 (s, 1H), 8.74 (dd, *J* = 6.0, 3.0 Hz, 1H), 8.02 (d, *J* = 8.4 Hz, 1H), 7.60 (d, *J* = 9.4 Hz, 1H), 7.47-7.42 (m, 2H), 7.31 (d, *J* = 8.6 Hz, 2H), 6.79 (dd, *J* = 8.6, 2.4 Hz, 1H), 6.75 (d, *J* = 2.4 Hz, 1H), 6.22 (d, *J* = 9.4 Hz, 1H), 4.00 (t, *J* = 6.4 Hz, 2H), 2.74 (s, 3H), 2.56 (dd, *J* = 14.3, 6.5 Hz, 1H), 2.41 (dd, *J* = 14.3, 7.6 Hz, 1H), 2.27 – 2.17 (m, 1H), 1.87 – 1.80 (m, 2H), 1.64 – 1.52 (m, 3H), 1.42 – 1.34 (m, 1H), 1.08 (d, *J* = 6.6 Hz, 3H). ^{13}**C NMR** (125 MHz, $CDCl_3$) δ 171.2, 162.5, 161.4, 157.3, 156.0, 143.6, 137.8, 136.6, 134.0, 128.8, 126.5, 126.2, 122.5, 121.3, 116.5, 113.1, 113.0, 112.5, 101.4, 68.6, 45.9, 36.4, 30.8, 29.2, 25.4, 23.6, 20.0. **HRMS** (**ESI**): Calcd. 467.1941 for $C_{27}H_{28}N_2NaO_4$ $(M+Na)^+$, found 467.1949.

7-(1,3-Dioxoisoindolin-2-yl)-3-methyl-*N*-(2-methylquinolin-8-yl)heptanamide (9f):

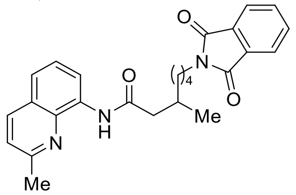

The title compound was prepared from **7a** (23 mg, 0.1 mmol) and 2-(4-iodobutyl)isoindoline-1,3-dione (66 mg, 0.2 mmol) according to the general procedure. Purification using flash silica gel column chromatography (eluent: 10:1 hexanes:EtOAc) gave the pure product as a colorlesssolid (40 mg, 94% yield). ^{1}**H NMR** (500 MHz, $CDCl_3$) δ 9.82 (s, 1H), 8.72 (dd, *J* = 6.1, 2.9 Hz, 1H), 8.00 (d, *J* = 8.4 Hz, 1H), 7.81 (dd, *J* = 5.4, 3.0 Hz, 2H), 7.67 (dd, *J* = 5.4, 3.0 Hz, 2H), 7.43-7.42 (m, 2H), 7.29 (d, *J* = 8.4 Hz, 1H), 3.68 (t, *J* = 7.3 Hz, 2H), 2.72 (s, 3H), 2.54 (dd, *J* = 14.3, 6.1 Hz, 1H), 2.34 (dd, *J* = 14.3, 8.1 Hz, 1H), 2.19 – 2.12 (m, 1H), 1.73 – 1.63 (m, 2H), 1.50 – 1.31 (m, 4H), 1.04 (d, *J* = 6.6 Hz, 3H). ^{13}**C NMR** (125 MHz, $CDCl_3$) δ 171.2, 168.5, 157.2, 137.8, 136.5, 134.0, 133.9, 132.2, 126.4, 126.1, 123.2, 122.5, 121.2, 116.5, 45.9, 38.0, 36.5, 30.8, 28.9, 25.4, 24.5, 19.8. **HRMS** (**ESI**): Calcd. 452.1945 for $C_{26}H_{27}N_3NaO_3$ $(M+Na)^+$, found 452.1941.

3-Methyl-*N*-(2-methylquinolin-8-yl)-7-(4-(trifluoromethyl)phenoxy)heptanamide (9g):

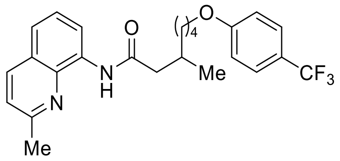

The title compound was prepared from **7a** (23 mg, 0.1 mmol) and 1-(4-iodobutoxy)-4-(trifluoromethyl)benzene (69 mg, 0.2 mmol) according to the general procedure. Purification using flash silica gel column chromatography (eluent: 15:1 hexanes:EtOAc) gave the pure product as a a colorless oil (32 mg, 72% yield, r.r. = 88:12).^{1}**H NMR** (400 MHz, $CDCl_3$) δ 9.89 (s, 1H), 8.76 – 8.74 (m, 1H), 8.03 (d, *J* = 8.4 Hz, 1H), 7.50 – 7.45 (m, 4H), 7.31 (d, *J* = 8.4 Hz, 1H), 6.90 (d, *J* = 8.5 Hz, 2H), 3.98 (t, *J* = 6.4 Hz, 2H), 2.74 (s, 3H), 2.57 (dd, *J* = 14.3, 6.5 Hz, 1H), 2.41 (dd, *J* = 14.3, 7.6 Hz, 1H), 2.28 – 2.18 (m, 1H), 1.85 – 1.78 (m, 2H), 1.64 – 1.36 (m, 4H), 1.09 (d, *J* = 6.7 Hz, 3H). ^{13}**C NMR** (100 MHz, $CDCl_3$) δ 171.5, 161.7, 157.4, 137.8, 136.6, 133.9, 126.9 (q, *J* = 3.7 Hz), 126.5,124.6 (q, *J* = 266.0 Hz), 123.1 (q, *J* = 43.4 Hz), 122.6, 121.5, 116.6, 115.7, 114.5, 68.2, 46.0, 36.5, 30.9, 29.3, 25.4, 23.6, 19.9. **HRMS** (**ESI**): Calcd. 467.1917 for $C_{25}H_{27}F_3N_2NaO_2(M+Na)^+$, found 467.1908.

7-(4-Formylphenoxy)-3-methyl-*N*-(2-methylquinolin-8-yl)heptanamide (9h):

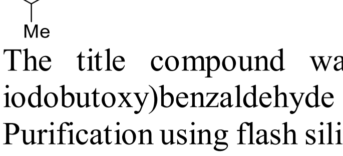

The title compound was prepared from **7a** (23 mg, 0.1 mmol) and 4-(4-iodobutoxy)benzaldehyde (61 mg, 0.2 mmol) according to the general procedure. Purification using flash silica gel column chromatography (eluent: 10:1 hexanes:EtOAc) gave the pure product as a colorless oil (25 mg, 62% yield). ^{1}**H NMR** (300 MHz, $CDCl_3$) δ 9.88 – 9.83 (m, 2H), 8.74 (t, *J* = 4.5 Hz, 1H), 8.03 (d, *J* = 8.3 Hz, 1H), 7.78 (d, *J* = 8.1 Hz, 2H), 7.45 (d, *J* = 4.6 Hz, 2H), 7.31 (d, *J* = 8.3 Hz, 1H), 6.94 (d, *J* = 8.7 Hz, 2H), 4.04 (t, *J* = 6.5 Hz, 2H), 2.73 (s, 3H), 2.56 (dd, *J* = 14.2, 6.5 Hz, 1H), 2.41 (dd, *J* = 14.3,

7.5 Hz, 1H), 2.27 – 2.19 (m, 1H), 1.88 – 1.80 (m, 2H), 1.63 – 1.36 (m, 4H), 1.09 (t, *J* = 6.3 Hz, 3H). **^{13}C NMR** (100 MHz, $CDCl_3$) δ 191.0, 171.3, 164.3, 157.3, 137.8, 136.6, 133.9, 132.1, 129.8, 126.4, 126.2, 122.5, 121.4, 116.5, 114.8, 68.3, 45.9, 36.4, 30.8, 29.2, 25.4, 23.5, 19.9. **HRMS** (**ESI**): Calcd. 427.1992 for $C_{25}H_{28}N_2NaO_3$ $(M+Na)^+$, found 427.1986.

7-(4-Bromophenoxy)-3-methyl-*N*-(2-methylquinolin-8-yl)heptanamide (9i):

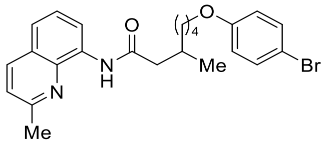

The title compound was prepared from **7a** (23 mg, 0.1 mmol) and 1-bromo-4-(4-iodobutoxy)benzene (71 mg, 0.2 mmol) according to the general procedure. Purification using flash silica gel column chromatography (eluent: 15:1 hexanes:EtOAc) gave the pure product as a colorless oil (34 mg, 74% yield, r.r. = 93:7). **^{1}H NMR** (500 MHz, $CDCl_3$) δ 9.86 (s, 1H), 8.75 (dd, *J* = 5.8, 3.3 Hz, 1H), 8.03 (d,*J* = 8.4 Hz, 1H), 7.46 – 7.45 (m, 2H), 7.34 – 7.30 (m, 3H), 6.73 (d, *J* = 8.9 Hz, 2H), 3.90 (t, *J* = 6.5 Hz, 2H), 2.74 (s, 3H), 2.56 (dd, *J* = 14.3, 6.4 Hz, 1H), 2.39 (dd, *J* = 14.3, 7.8 Hz, 1H), 2.26 – 2.17 (m, 1H), 1.82 – 1.75 (m, 2H), 1.61 – 1.45 (m, 4H), 1.08 (d, *J* = 6.6 Hz, 3H). **^{13}C NMR** (100 MHz, $CDCl_3$) δ 171.3, 158.3, 157.3, 136.6, 134.0, 132.3, 126.5, 126.2, 122.5, 121.4, 117.5, 116.4, 112.7, 68.2, 46.0, 36.5, 30.9, 29.4, 25.4, 23.6, 19.9. **HRMS** (**ESI**): Calcd. 477.1148 for $C_{24}H_{27}BrN_2NaO_2$ $(M+Na)^+$, found 477.1150.

7-(4-Iodophenoxy)-3-methyl-*N*-(2-methylquinolin-8-yl)heptanamide (9j):

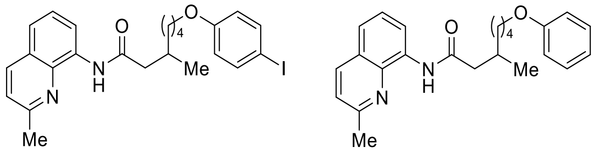

4 : 1

The title compound was prepared from **7a** (23 mg, 0.1 mmol) and 1-bromo-4-(4-iodobutoxy)benzene (80 mg, 0.2 mmol) according to the general procedure. Purification using flash silica gel column chromatography (eluent: 15:1 hexanes:EtOAc) gave the pure product as a colorless oil (contain an inseparable hydrodeiodination side product) (26 mg, 52% yield). **^{1}H NMR** (500 MHz, $CDCl_3$) δ 9.86 (s, 1H), 8.75 (dd, *J* = 5.7, 3.3 Hz, 1H), 8.03 (d, *J* = 8.3 Hz, 1H), 7.52 – 7.43 (m, 4H), 7.31 (d, *J* = 8.3 Hz, 1H), 6.63 (d, *J* = 8.9 Hz, 2H), 3.90 (t, *J* = 6.5 Hz, 2H), 2.74 (s, 3H), 2.56 (dd, *J* = 14.3, 6.4 Hz, 1H), 2.39 (dd, *J* = 14.3, 7.7 Hz, 1H), 2.25 – 2.16 (m, 1H), 1.81 – 1.75 (m,2H), 1.63 – 1.45 (m, 4H), 1.08 (d, *J* = 6.7 Hz, 3H). **^{13}C NMR** (125 MHz, $CDCl_3$) δ 171.3, 159.1, 157.3, 138.2, 136.6, 134.0, 126.5, 126.2, 122.5, 121.3, 118.1, 117.0, 116.5, 114.6, 68.1, 46.0, 36.5, 30.9, 29.5, 25.4, 23.6, 19.9. **HRMS** (**ESI**): Calcd. 503.1190 for $C_{24}H_{28}IN_2O_2$ $(M+H)^+$, found 503.1201.

7-Methyl-9-((2-methylquinolin-8-yl)amino)-9-oxononanoic acid (9k):

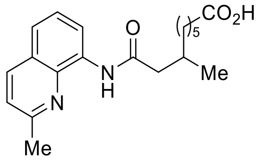

The title compound was prepared from **7a** (23 mg, 0.1 mmol) and 6-iodohexanoic acid (48 mg, 0.2 mmol) according to the general procedure. Purification using flash silica gel column chromatography (eluent: 1:1 hexanes:EtOAc) gave the pure product as a

colorless oil (19 mg, 55% yield). **^{1}H NMR** (400 MHz, $CDCl_3$) δ 9.85 (s, 1H), 8.74 (dd, *J* = 5.9, 3.1 Hz, 1H), 8.03 (d, *J* = 8.4 Hz, 1H), 7.46 – 7.44 (m, 2H), 7.31 (d, *J* = 8.4 Hz, 1H), 2.74 (s, 3H), 2.56 (dd, *J* = 14.3, 6.2 Hz, 1H), 2.38 – 2.31 (m, 3H), 2.20 – 2.12 (m, 1H), 1.64 (p, *J* = 7.4 Hz, 2H), 1.48 – 1.27 (m, 6H), 1.05 (d, *J* = 6.6 Hz, 3H). **^{13}C NMR** (100 MHz, $CDCl_3$) δ 179.3, 171.5, 157.3, 137.8, 136.6, 134.0, 126.5, 126.2, 122.5, 121.3, 116.6, 46.0, 36.7, 34.1, 30.9, 29.3, 26.8, 25.4, 24.8, 20.0. **HRMS** (**ESI**): Calcd. 343.2016 for $C_{20}H_{27}N_2O_3$ $(M+H)^+$, found 343.2025.

4-Methyl-6-((2-methylquinolin-8-yl)amino)-6-oxohexyl (2*R*,5*S*)-3,3-dimethyl-7-oxo-4-thia-1-azabicyclo[3.2.0]heptane-2-carboxylate 4,4-dioxide (9l):

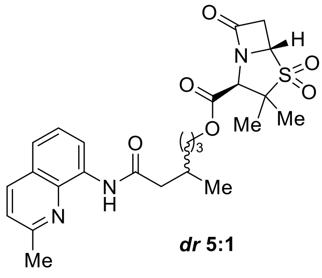

The title compound was prepared from **7a** (23 mg, 0.1 mmol) and 3-iodopropyl (2*R*,5*S*)-3,3-dimethyl-7-oxo-4-thia-1-azabicyclo[3.2.0]heptane-2-carboxylate 4,4-dioxide (80 mg, 0.2 mmol) according to the general procedure. Purification using flash silica gel column chromatography (eluent: 4:1 hexanes:EtOAc) gave the pure product as a colorlesssolid (38 mg, 75% yield, *dr* = 5:1). **^{1}H NMR** (500 MHz, $CDCl_3$) δ 9.84 (s, 1.2H), 8.72 (t, *J* = 4.5 Hz, 1.2H), 8.03 (d, *J* = 8.4 Hz, 1.2H), 7.45 (d, *J* = 4.5 Hz, 2.4H), 7.32 (d, *J* = 8.4 Hz, 1.2H), 4.61 (dd, *J* = 4.3, 2.1 Hz, 0.2H), 4.59 – 4.57 (m, 1H), 4.37 (d, *J* = 4.0 Hz, 0.2H), 4.34 (d, *J* = 3.6 Hz, 1H), 4.21 (t, *J* = 6.0 Hz, 2.4H), 3.46 – 3.38 (m, 2.4H), 2.74 (s, 3.6H), 2.59 – 2.42 (m, 2.4H), 2.25 – 2.18 (m, 1.2H), 1.83 – 1.70 (m, 4.8H), 1.60 (d, *J* = 6.3 Hz, 0.6H), 1.53 (d, *J* = 4.0 Hz, 3H), 1.40 (d, *J* = 5.2 Hz, 0.6H), 1.36 (d, *J* = 1.6 Hz, 3H), 1.08 (d, *J* = 6.6 Hz, 3.6H). **^{13}C NMR** (125 MHz, $CDCl_3$) δ 170.8, 170.8, 167.1, 157.4, 137.7, 136.6, 133.8, 126.4, 126.2, 122.6, 121.5 116.5, 66.7, 66.7, 63.4, 62.8, 61.2, 45.7, 45.7, 38.4, 32.8, 32.8, 30.4, 26.2, 26.1, 25.4, 20.4, 19.9, 19.9, 18.7, 18.6. **HRMS** (**ESI**): Calcd. 524.1826 for $C_{25}H_{31}N_3NaO_6S$ $(M+Na)^+$, found524.1821.

4-Methyl-6-((2-methylquinolin-8-yl)amino)-6-oxohexyl 2-(1-(4-chlorobenzoyl)-5-methoxy-2-methyl-1*H*-indol-3-yl)acetate (9m):

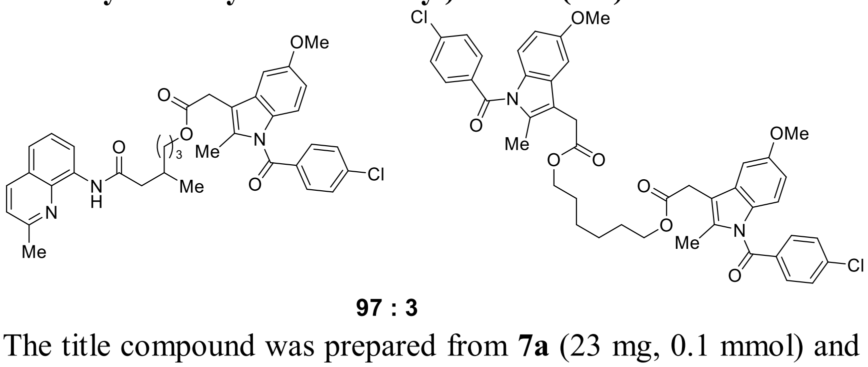

97 : 3

The title compound was prepared from **7a** (23 mg, 0.1 mmol) and 3-iodopropyl 2-(1-(4-chlorobenzoyl)-5-methoxy-2-methyl-1*H*-indol-3-yl)acetate (105 mg, 0.2 mmol) according to the general procedure. Purification using flash silica gel column chromatography (eluent: 4:1 hexanes:EtOAc) gave the pure product as a colorlesssolid (contain an inseparable self-coupling side product) (35 mg, 56% yield). **^{1}H NMR** (400 MHz, $CDCl_3$) δ 9.82 (s, 1H), 8.73 (dd, *J* = 5.1, 3.9 Hz, 1H), 8.02 (d, *J* = 8.4 Hz, 1H),

7.66 – 7.63 (m, 2H), 7.45 – 7.43 (m, 4H), 7.31 (d, *J* = 8.4 Hz, 1H), 6.97 (d, *J* = 2.5 Hz, 1H), 6.87 (d, *J* = 9.0 Hz, 1H), 6.67 (dd, *J* = 9.0, 2.5 Hz, 1H), 4.12 (t, *J* = 6.6 Hz, 2H), 3.82 (s, 3H), 3.64 (s, 2H), 2.73 (s, 3H), 2.51 – 2.46 (m, 1H), 2.36 (s, 3H), 2.30 (dd, *J* = 14.4, 8.0 Hz, 1H), 2.21 – 2.10 (m, 1H), 1.79 – 1.62 (m, 2H), 1.51 – 1.42 (m, 1H), 1.32 – 1.23 (m, 1H), 1.01 (d, *J* = 6.6 Hz, 3H). **^{13}C NMR** (126 MHz, $CDCl_3$) δ 170.9, 170.8, 168.2, 157.2, 156.0, 139.2, 137.6, 136.4, 135.9, 133.9, 133.8, 131.1, 130.8, 130.7, 129.1, 126.3, 126.0, 122.4, 121.2, 116.4, 114.9, 112.7, 111.6, 101.4, 65.1, 55.7, 45.5, 32.9, 30.4, 28.4, 26.2, 25.2, 19.6, 13.3 ppm. **HRMS** (**ESI**): Calcd. 626.2416 for $C_{36}H_{37}ClN_3O_5$ $(M+H)^+$, found 626.2422.

3-Methyl-*N*-(2-methylquinolin-8-yl)hept-6-enamide (9n):

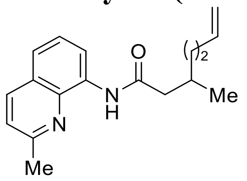

The title compound was prepared from **7a** (23 mg, 0.1 mmol) and 4-bromobut-1-ene (27 mg, 0.2 mmol) according to the general procedure. Purification using flash silica gel column chromatography (eluent: 15:1 hexanes:EtOAc) gave the pure product as a colorless oil (20 mg, 72% yield). **^{1}H NMR** (400 MHz, $CDCl_3$) δ 9.85 (s, 1H), 8.75 (dd, *J* = 6.1, 2.9 Hz, 1H), 8.02 (d, *J* = 8.4 Hz, 1H), 7.46 – 7.43 (m, 2H), 7.31 (d, *J* = 8.4 Hz, 1H), 5.83 (ddt, *J* = 16.9, 10.2, 6.6 Hz, 1H), 5.04 (dq, *J* = 17.1, 1.6 Hz, 1H), 4.96 (ddt, *J* = 10.2, 2.3, 1.2 Hz, 1H), 2.74 (s, 3H), 2.58 (dd, *J* = 14.3, 6.0 Hz, 1H), 2.36 (dd, *J* = 14.2, 8.1 Hz, 1H), 2.24 – 2.06 (m, 3H), 1.66 – 1.52 (m, 2H), 1.07 (d, *J* = 6.6 Hz, 3H). **^{13}C NMR** (75 MHz, $CDCl_3$) δ 171.2, 157.3, 138.8, 137.8, 136.5, 134.0, 126.5, 126.2, 122.5, 121.3, 116.5, 114.7, 45.9, 36.1, 31.4, 30.5, 25.4, 19.8. **HRMS** (**ESI**): Calcd. 305.1624 for $C_{18}H_{22}N_2NaO$ $(M+Na)^+$, found 305.1624.

5-(1,3-Dioxolan-2-yl)-3-methyl-*N*-(2-methylquinolin-8-yl)pentanamide (9o):

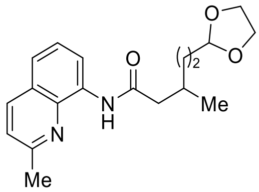

The title compound was prepared from **7a** (23 mg, 0.1 mmol) and 2-(2-bromoethyl)-1,3-dioxolane (36 mg, 0.2 mmol) according to the general procedure. Purification using flash silica gel column chromatography (eluent: 8:1 hexanes:EtOAc) gave the pure product as a colorless oil (21mg, 64% yield). **^{1}H NMR** (400 MHz, $CDCl_3$) δ 9.84 (s, 1H), 8.75 (dd, *J* = 5.9, 3.1 Hz, 1H), 8.03 (d, *J* = 8.4 Hz, 1H), 7.48 – 7.43 (m, 2H), 7.31 (d, *J* = 8.4 Hz, 1H), 4.87 (t, *J* = 4.7 Hz, 1H), 3.98 – 3.94 (m, 2H), 3.86 – 3.82 (m, 2H), 2.75 (s, 3H), 2.60 (dd, *J* = 14.2, 5.8 Hz, 1H), 2.35 (dd, *J* = 14.3, 8.3 Hz, 1H), 2.26 – 2.17 (m, 1H), 1.83 – 1.71 (m, 2H), 1.63 – 1.56 (m, 1H), 1.48 – 1.40 (m, 1H), 1.08 (d, *J* = 6.6 Hz, 3H). **^{13}C NMR** (125 MHz, $CDCl_3$) δ 171.1, 157.3, 137.8, 136.5, 134.0, 126.5, 126.2, 122.5, 121.3, 116.5, 104.8, 65.0, 45.8, 31.6, 31.1, 30.8, 25.4, 19.8. **HRMS** (**ESI**): Calcd. 351.1679 for $C_{19}H_{24}N_2NaO_3$ $(M+Na)^+$, found 351.1680.

5-Hydroxy-3-methyl-*N*-(2-methylquinolin-8-yl)pentanamide (9p):

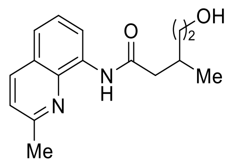

The title compound was prepared from **7a** (23 mg, 0.1 mmol) and 2-bromoethan-1-ol (25mg, 0.2 mmol) according to the general procedure. Purification using flash silica gel column chromatography (eluent: 3:1 hexanes:EtOAc) gave the pure product as a colorless oil (23 mg, 85% yield). **^{1}H NMR** (500 MHz, $CDCl_3$) δ 9.88 (s, 1H), 8.72 – 8.70 (m, 1H), 8.02 (d, *J* = 8.5 Hz, 1H), 7.44 (d, *J* = 4.2 Hz, 2H), 7.30 (d, *J* = 8.4 Hz, 1H), 3.79 – 3.66 (m, 2H), 2.73 (s, 3H), 2.59 (dd, *J* = 14.5, 7.2 Hz, 1H), 2.49 (dd, *J* = 14.5, 6.4 Hz, 1H), 2.38 (dq, *J* = 13.5, 6.7 Hz, 1H), 1.66 (q, *J* = 6.5 Hz, 2H), 1.10 (d, *J* = 6.6 Hz, 3H). **^{13}C NMR** (125 MHz, $CDCl_3$) δ 171.6, 157.4, 137.8, 136.6, 133.8, 126.4, 126.2, 122.5, 121.5, 116.9, 60.7, 45.4, 39.6, 27.6, 25.3, 20.7. **HRMS** (**ESI**): Calcd.295.1417 for $C_{16}H_{20}N_2NaO_2$ $(M+Na)^+$, found 295.1412.

6-Hydroxy-3-methyl-*N*-(2-methylquinolin-8-yl)hexanamide (9q):

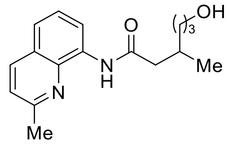

The title compound was prepared from **7a** (23 mg, 0.1 mmol) and 3-bromopropan-1-ol (28 mg, 0.2 mmol) according to the general procedure. Purification using flash silica gel column chromatography (eluent: 3:1 hexanes:EtOAc) gave the pure product as a colorless oil (20 mg, 70% yield). **^{1}H NMR** (500 MHz, $CDCl_3$) δ 9.85 (s, 1H), 8.73 (dd, *J* = 5.8, 3.2 Hz, 1H), 8.02 (d, *J* = 8.3 Hz, 1H), 7.46 – 7.43 (m, 2H), 7.30 (d, *J* = 8.3 Hz, 1H), 3.67 (t, *J* = 6.4 Hz, 2H), 2.74 (s, 3H), 2.56 (dd, *J* = 14.5, 6.7 Hz, 1H), 2.40 (dd, *J* = 14.4, 7.5 Hz, 1H), 2.25 – 2.16 (m, 1H), 1.89 (brs, 1H), 1.73 – 1.53 (m, 3H), 1.38-1.31 (m, 1H), 1.07 (d, *J* = 6.6 Hz, 3H). **^{13}C NMR** (125 MHz, $CDCl_3$) δ 171.4, 157.3, 137.8, 136.6, 133.9, 126.5, 126.2, 122.5, 121.4, 116.6, 63.0, 45.8, 32.8, 30.4, 30.1, 25.4, 20.0. **HRMS** (**ESI**): Calcd. 309.1573 for $C_{17}H_{22}N_2NaO_2$ $(M+Na)^+$, found 309.1579.

7-(4-Hydroxyphenoxy)-3-methyl-*N*-(2-methylquinolin-8-yl)heptanamide (9r):

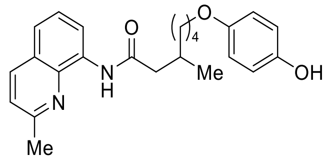

The title compound was prepared from **7a** (23 mg, 0.1 mmol) and 4-(4-bromobutoxy)phenol (49 mg, 0.2 mmol) according to the general procedure. Purification using flash silica gel column chromatography (eluent: 5:1 hexanes:EtOAc) gave the pure product as a colorless solid (30 mg, 77% yield). **^{1}H NMR** (400 MHz, DMSO-d_6) δ 9.91 (s, 1H), 8.87 (s, 1H), 8.59 (dd, *J* = 7.7, 1.3 Hz, 1H), 8.26 (d, *J* = 8.4 Hz, 1H), 7.59 (dd, *J* = 8.2, 1.4 Hz, 1H), 7.50 -7.46 (m, 2H), 6.73 – 6.63 (m, 4H), 3.82 (t, *J* = 6.5 Hz, 2H), 2.72 (s, 3H), 2.54 (dd, *J* = 14.4, 6.2 Hz, 1H), 2.39 (dd, *J* = 14.5, 7.9 Hz, 1H), 2.04 (td, *J* = 14.9, 14.0, 7.5 Hz, 1H), 1.71 – 1.60 (m, 2H), 1.52 – 1.21 (m, 4H), 0.97 (d, *J* = 6.6 Hz, 3H). **^{13}C NMR** (100 MHz, DMSO-d_6) δ 171.3, 157.9, 152.0, 151.5, 150.2, 137.8, 137.1, 134.2, 126.4, 123.3, 121.9, 116.8, 116.1, 115.8, 68.3, 44.9, 36.3, 30.6, 29.5, 25.4, 23.5, 20.0. **HRMS** (**ESI**): Calcd. 415.1992 for $C_{24}H_{28}N_2NaO_3$ $(M+Na)^+$, found415.1995.

7-Chloro-3-methyl-*N*-(2-methylquinolin-8-yl)heptanamide (9s):

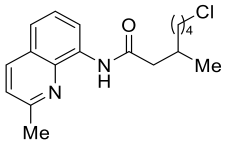

The title compound was prepared from **7a** (23 mg, 0.1 mmol) and 1-bromo-4-chlorobutane (34 mg, 0.2 mmol) according to the general procedure. Purification using flash silica gel column chromatography (eluent: 15:1 hexanes:EtOAc) gave the pure product as a colorless solid (21 mg, 66% yield). ^{1}**H NMR** (500 MHz, $CDCl_3$) δ 9.85 (s, 1H), 8.74 (dd, *J* = 6.1, 2.9 Hz, 1H), 8.03 (d, *J* = 8.4 Hz, 1H), 7.46 – 7.44 (m, 2H), 7.31 (d, *J* = 8.3 Hz, 1H), 3.54 (t, *J* = 6.7 Hz, 2H), 2.74 (s, 3H), 2.55 (dd, *J* = 14.3, 6.4 Hz, 1H), 2.38 (dd, *J* = 14.3, 7.8 Hz, 1H), 2.21 – 2.14 (m, 1H), 1.83- 1.76 (m, 2H), 1.59 – 1.48 (m, 3H), 1.35-1.30 (m, 1H), 1.07 (d, *J* = 6.7 Hz, 3H). ^{13}**C NMR** (125 MHz, $CDCl_3$) δ 171.2, 157.3, 137.8, 136.6, 134.0, 126.5, 126.2, 122.5, 121.3, 116.5, 45.9, 45.2, 36.0, 32.8, 30.8, 25.4, 24.5, 19.9. **HRMS** (**ESI**): Calcd.341.1391 for $C_{18}H_{23}ClN_2NaO$ $(M+Na)^+$, found 341.1399.

3-Methyl-*N*-(2-methylquinolin-8-yl)-7-(4,4,5,5-tetramethyl-1,3,2-dioxaborolan-2-yl)heptanamide (9t):

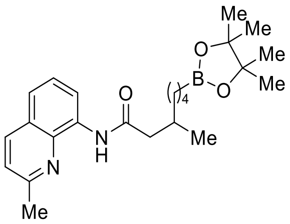

The title compound was prepared from **7a** (23 mg, 0.1 mmol) and 2-(4-bromobutyl)-4,4,5,5-tetramethyl-1,3,2-dioxaborolane (53 mg, 0.2 mmol) according to the general procedure. Purification using flash silica gel column chromatography (eluent: 15:1 hexanes:EtOAc) gave the pure product as a colorless solid (34 mg, 83% yield). ^{1}**H NMR** (500 MHz, $CDCl_3$) δ 9.83 (s, 1H), 8.75 (dd, *J* = 6.6, 2.4 Hz, 1H), 8.02 (d, *J* = 8.4 Hz, 1H), 7.47 – 7.42 (m, 2H), 7.30 (d, *J* = 8.4 Hz, 1H), 2.74 (s, 3H), 2.57 (dd, *J* = 14.2, 5.8 Hz, 1H), 2.31 (dd, *J* = 14.2, 8.4 Hz, 1H), 2.21 – 2.12 (m, 1H), 1.47 – 1.29 (m, 6H), 1.23 (s, 12H), 1.04 (d, *J* = 6.6 Hz, 3H), 0.79 (t, *J* = 7.3 Hz, 2H). ^{13}**C NMR** (75 MHz, $CDCl_3$) δ 171.5, 157.2, 137.8, 136.5, 134.1, 126.5, 126.1, 122.5, 121.2, 116.5, 83.0, 46.10, 36.8, 30.9, 29.9, 25.4, 24.9, 24.3, 19.9. **HRMS** (**ESI**): Calcd. 433.2637 for $C_{24}H_{35}BN_2NaO_3$ $(M+Na)^+$, found 433.2637.

7-(1*H*-Indol-1-yl)-3-methyl-*N*-(2-methylquinolin-8-yl)heptanamide (9u):

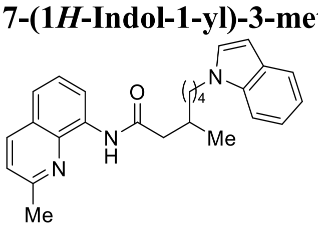

The title compound was prepared from **7a** (23 mg, 0.1 mmol) and 1-(4-bromobutyl)-1*H*-indole (50 mg, 0.2 mmol) according to the general procedure. Purification using flash silica gel column chromatography (eluent: 12:1 hexanes:EtOAc) gave the pure product as a colorless solid (35 mg, 88% yield, r.r. = 92:8). ^{1}**H NMR** (400 MHz, $CDCl_3$) δ 9.84 (s, 1H), 8.77 (dd, *J* = 6.3, 2.7 Hz, 1H), 8.03 (d, *J* = 8.3 Hz, 1H), 7.63 (dt, *J* = 7.8, 1.0 Hz, 1H), 7.47 – 7.44 (m, 2H), 7.35 – 7.31 (m, 2H), 7.19 (ddd, *J* = 8.3, 7.0, 1.2 Hz, 1H), 7.11 – 7.07 (m, 2H), 6.47 (dd, *J* = 3.1, 0.9 Hz, 1H), 4.12 (t, *J* = 7.1 Hz, 2H), 2.74

(s, 3H), 2.52 (dd, *J* = 14.5, 6.6 Hz, 1H), 2.37 (dd, *J* = 14.4, 7.6 Hz, 1H), 2.23 – 2.12 (m, 1H), 1.91 – 1.83 (m, 2H), 1.52 – 1.32 (m, 4H), 1.04 (d, *J* = 6.6 Hz, 3H). **^{13}C NMR** (100 MHz, $CDCl_3$) δ 171.2, 157.3, 137.79, 136.6, 136.0, 134.0, 128.7, 127.9, 126.5, 126.2, 122.5, 121.4, 121.3, 121.0, 119.2, 116.5, 109.5, 101.0, 46.4, 45.9, 36.4, 30.8, 30.4, 25.4, 24.6, 19.9. **HRMS** (**ESI**): Calcd. 400.2383 for $C_{26}H_{30}N_3O$ $(M+H)^+$, found 400.2389.

8-Benzo[*d*]oxazol-2-yl)-3-methyl-*N*-(2-methylquinolin-8-yl)octanamide (9v):

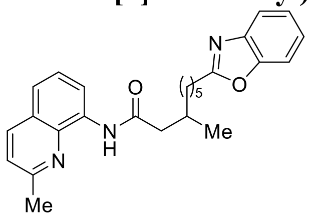

The title compound was prepared from **7a** (23 mg, 0.1 mmol) and 2-(5-bromopentyl)benzo[*d*]oxazole (54 mg, 0.2 mmol) according to the general procedure. Purification using flash silica gel column chromatography (eluent: 12:1 hexanes:EtOAc) gave the pure product as a colorless solid (37 mg, 90% yield). **^{1}H NMR** (400 MHz, $CDCl_3$) δ 9.76 (s, 1H), 8.67 (dd, *J* = 6.4, 2.6 Hz, 1H), 7.93 (d, *J* = 8.4 Hz, 1H), 7.59 – 7.57 (m, 1H), 7.40 – 7.33 (m, 3H), 7.23 – 7.17 (m, 3H), 2.84 (t, *J* = 7.6 Hz, 2H), 2.66 (s, 3H), 2.47 (dd, *J* = 14.3, 6.1 Hz, 1H), 2.27 (dd, *J* = 14.3, 8.0 Hz, 1H), 2.13 – 2.05 (m, 1H), 1.84 – 1.78 (m, 2H), 1.42 – 1.32 (m, 6H), 0.97 (d, *J* = 6.6 Hz, 3H). **^{13}C NMR** (100 MHz, $CDCl_3$) δ 171.4, 167.4, 157.2, 150.9, 141.5, 137.8, 136.5, 134.0, 126.5, 126.2, 124.5, 124.1, 122.5, 121.2, 119.6, 116.5, 110.4, 46.0, 36.7, 30.9, 29.4, 28.7, 26.8, 26.8, 25.4, 19.9. **HRMS** (**ESI**): Calcd.438.2152 for $C_{26}H_{29}N_3NaO_2$ $(M+Na)^+$, found 438.2151.

3,4-Dimethyl-*N*-(2-methylquinolin-8-yl)pentanamide (9x):

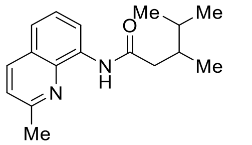

The title compound was prepared from **7a** (23 mg, 0.1 mmol) and 2-iodopropane (34 mg, 0.2 mmol) according to the general procedure. Purification using flash silica gel column chromatography (eluent: 15:1 hexanes:EtOAc) gave the pure product as a colorless oil (19 mg, 71% yield). **^{1}H NMR** (500 MHz, $CDCl_3$) δ 9.86 (s, 1H), 8.75 (dd, *J* = 6.6, 2.4 Hz, 1H), 8.03 (d, *J* = 8.4 Hz, 1H), 7.46 – 7.43 (m, 2H), 7.31 (d, *J* = 8.4 Hz, 1H), 2.75 (s, 3H), 2.62 (dd, *J* = 14.2, 5.3 Hz, 1H), 2.29 (dd, *J* = 14.2, 9.1 Hz, 1H), 2.15 – 2.08 (m, 1H), 1.73 (pd, *J* = 6.8, 4.6 Hz, 1H), 1.00 (d,*J* = 6.8 Hz, 3H), 0.97 (d, *J* = 6.8 Hz, 3H), 0.94 (d, *J* = 6.8 Hz, 3H). **^{13}C NMR** (125 MHz, $CDCl_3$) δ 171.8, 157.3, 137.8, 136.6, 134.1, 126.5, 126.2, 122.5, 121.2, 116.5, 43.3, 36.5, 32.2, 25.4, 20.2, 18.4, 15.9. **HRMS** (**ESI**): Calcd. 293.1624 for $C_{17}H_{22}N_2NaO$ $(M+Na)^+$, found 293.1625.

***tert*-Butyl 4-(4-((2-methylquinolin-8-yl)amino)-4-oxobutan-2-yl)piperidine-1-carboxylate (9y):**

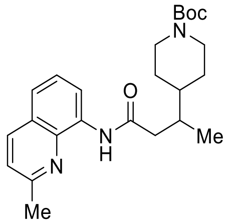

The title compound was prepared from **7a** (23 mg, 0.1 mmol) and *tert*-butyl 4-iodopiperidine-1-carboxylate (62 mg, 0.2 mmol) according to the general procedure. Purification using flash silica gel column chromatography (eluent: 15:1 hexanes:EtOAc) gave the pure product as a colorless solid (28 mg, 68% yield). ^{1}**H NMR** (500 MHz, $CDCl_3$) δ 9.85 (s, 1H), 8.73 (dd, *J* = 5.7, 3.3 Hz, 1H), 8.02 (d, *J* = 8.4 Hz, 1H), 7.45 – 7.44 (m, 2H), 7.31 (d, *J* = 8.4 Hz, 1H), 4.15 (brs, 2H), 2.74 (s, 3H), 2.64 (dd, *J* = 14.4, 5.3 Hz, 3H), 2.33 (dd, *J* = 14.4, 8.6 Hz, 1H), 2.18 – 2.10 (m, 1H), 1.70 – 1.65 (m, 3H), 1.45 (s, 9H), 1.35 – 1.24 (m, 2H), 1.02 (d, *J* = 6.8 Hz, 3H). ^{13}**C NMR** (125 MHz, $CDCl_3$) δ 171.2, 157.3, 155.0, 137.8, 136.6, 133.9, 126.5, 126.2, 122.5, 121.4, 116.5, 79.4, 44.3, 42.9, 41.0, 35.2, 29.8, 28.6, 28.2, 25.4, 22.8, 16.6. **HRMS** (**ESI**): Calcd. 434.2414 for $C_{24}H_{33}N_3NaO_3$ $(M+Na)^+$, found 434.2416.

3-((8*S*,9*S*,10*R*,13*R*,14*S*,17*R*)-10,13-Dimethyl-17-((*R*)-6-methylheptan-2-yl)-2,3,4,7,8,9,10,11,12,13,14,15,16,17-tetradecahydro-1*H*-cyclopenta[*a*]phenanthren-3-yl)-*N*-(2-methylquinolin-8-yl)butanamide (9z):

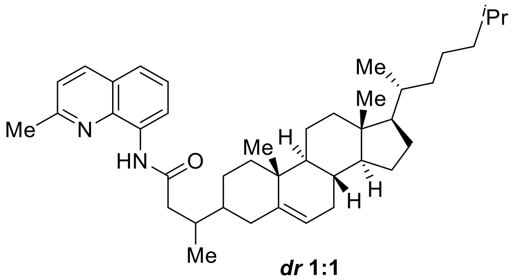

The title compound was prepared from **7a** (23 mg, 0.1 mmol) and (8*S*,9*S*,10*R*,13*R*,14*S*,17*R*)-3-iodo-10,13-dimethyl-17-((*R*)-6-methylheptan-2-yl)-2,3,4,7,8,9,10,11,12,13,14,15,16,17-tetradecahydro-1*H*-cyclopenta[*a*]phenanthrene (99 mg, 0.2 mmol) according to the general procedure. Purification using flash silica gel column chromatography (eluent: 15:1 hexanes:EtOAc) gave the pure product as a colorless solid (30 mg, 51% yield, *dr* = 1:1). ^{1}**H NMR** (500 MHz, $CDCl_3$) δ 9.85 (s, 1H), 8.75 (dt, *J* = 6.6, 2.3 Hz, 1H), 8.03 (d, *J* = 8.4 Hz, 1H), 7.47 – 7.42 (m, 2H), 7.31 (d, *J* = 8.4 Hz, 1H), 5.32 – 5.29 (m, 1H), 2.75 (s, 3H),2.69 (ddd, *J* = 14.3, 5.1, 1.8 Hz, 1H), 2.31 (dd, *J* = 14.3, 9.2 Hz, 1H), 2.15 – 1.79 (m, 8H), 1.66 – 1.30 (m, 16H), 1.16 – 1.07 (m, 6H), 1.04 (dd, *J* = 6.9, 2.7 Hz, 3H), 0.98 (s, 3H), 0.91 (d, *J* = 6.5 Hz, 3H), 0.86 (dd, *J* = 6.6, 2.3 Hz, 6H), 0.68 (s, 3H). ^{13}**C NMR** (125 MHz, $CDCl_3$) δ 171.7, 157.3, 143.3, 137.8, 136.6, 134.1, 126.5, 126.2, 122.5, 121.2, 119.8, 119.8, 116.5, 57.0, 56.3, 50.6, 44.3, 44.2, 43.3, 43.3, 42.5, 40.0, 39.8, 39.7, 39.7, 37.4, 36.9, 36.3, 35.9, 35.7, 35.6, 35.6, 32.1, 32.0, 28.4, 28.2, 26.4, 25.4, 24.9, 24.4, 24.0, 23.0, 22.7, 21.1, 19.7, 18.9, 16.8, 16.7, 12.0. **HRMS** (**ESI**): Calcd. 597.4778 for $C_{41}H_{61}N_2O$ $(M+H)^+$, found 597.4769.

3-Cyclopentyl-*N*-(2-methylquinolin-8-yl)butanamide (9aa):

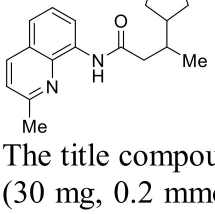

The title compound was prepared from **7a** (23 mg, 0.1 mmol) and bromocyclopentane (30 mg, 0.2 mmol) according to the general procedure. Purification using flash silica gel column chromatography (eluent: 15:1 hexanes:EtOAc) gave the pure product as a colorless oil (18 mg, 60% yield). ^{1}**H NMR** (400 MHz, $CDCl_3$) δ 9.85 (s, 1H), 8.75 (dd, *J* = 6.5, 2.5 Hz, 1H), 8.02 (d, *J* = 8.4 Hz, 1H), 7.48 – 7.42 (m, 2H), 7.31 (d, *J* = 8.4 Hz,

1H), 2.74 (s, 3H), 2.71 (dd, *J* = 14.2, 4.7 Hz, 1H), 2.30 (dd, *J* = 14.2, 9.2 Hz, 1H), 2.09-1.99 (m, 1H), 1.86 – 1.79 (m, 3H), 1.67 – 1.51 (m, 4H), 1.29 – 1.20 (m, 2H), 1.06 (d, *J* = 6.6 Hz, 3H). **^{13}C NMR** (100 MHz, $CDCl_3$) δ 171.7, 157.2, 137.8, 136.6, 134.1, 126.5, 126.2, 122.5, 121.2, 116.5, 46.3, 45.1, 36.2, 32.8, 31.0, 30.3, 25.6, 25.4, 18.5. **HRMS** (**ESI**): Calcd. 319.1781 for $C_{19}H_{24}N_2NaO$ $(M+Na)^+$, found 319.1782.

***N*-(2-methylquinolin-8-yl)-3-(tetrahydro-2*H*-pyran-4-yl)butanamide (9ab):**

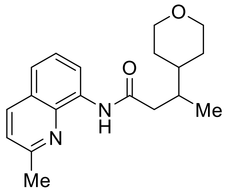

The title compound was prepared from **7a** (23 mg, 0.1 mmol) and 4-bromotetrahydro-2*H*-pyran (33 mg, 0.2 mmol) according to the general procedure. Purification using flash silica gel column chromatography (eluent: 10:1 hexanes:EtOAc) gave the pure product as a colorless oil (23 mg, 73% yield, r.r. = 90:10). **^{1}H NMR** (300 MHz, $CDCl_3$) δ 9.86 (s, 1H), 8.74 (dd, *J* = 5.9, 3.1 Hz, 1H), 8.03 (d, *J* = 8.3 Hz, 1H), 7.49 – 7.44 (m, 2H), 7.32 (d, *J* = 8.3 Hz, 1H), 4.01 (dt, *J* = 10.4, 5.0 Hz, 2H), 3.38 (tdd, *J* = 11.6, 4.6, 2.3 Hz, 2H), 2.74 (s, 3H), 2.67 (dd, *J* = 14.3, 5.2 Hz, 1H), 2.32 (dd, *J* = 14.2, 8.7 Hz, 1H), 2.24 – 2.02 (m, 1H), 1.64 – 1.38 (m, 5H), 1.04 (d, *J* = 6.7 Hz, 3H). **^{13}C NMR** (75 MHz, $CDCl_3$) δ 171.3, 157.3, 137.8, 136.6, 133.9, 126.5, 126.2, 122.5, 121.3, 116.5, 68.4, 68.4, 42.8, 40.0, 35.5, 30.7, 29.3, 25.4, 16.5. **HRMS** (**ESI**): Calcd. 335.1730 for $C_{19}H_{24}N_2NaO_2$ $(M+Na)^+$, found 335.1736.

***tert*-Butyl 4-(1-((2-methylquinolin-8-yl)amino)-1-oxopentan-3-yl)piperidine-1-carboxylate (9ac):**

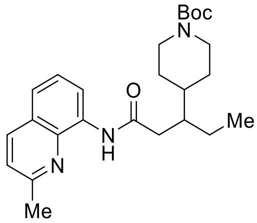

The title compound was prepared from **7b** (24 mg, 0.1 mmol) and *tert*-butyl 4-iodopiperidine-1-carboxylate (62 mg, 0.2 mmol) according to the general procedure. Purification using flash silica gel column chromatography (eluent: 6:1 hexanes:EtOAc) gave the pure product as a colorless solid (21 mg, 50% yield). **^{1}H NMR** (500 MHz, $CDCl_3$) δ 9.87 (s, 1H), 8.72 (dd, *J* = 5.6, 3.4 Hz, 1H), 8.03 (d, *J* = 8.4 Hz, 1H), 7.49-7.42 (m, 2H), 7.32 (d, *J* = 8.4 Hz, 1H), 4.14 (brs, 2H), 2.74 (s, 3H), 2.64 (brs, 2H), 2.56 (dd, *J* = 14.8, 6.2 Hz, 1H), 2.45 (dd, *J* = 15.1, 7.3 Hz, 1H), 2.00-1.94 (m, 1H), 1.68 – 1.63 (m, 5H), 1.44 (s, 9H), 1.31 – 1.27 (m, 2H), 0.98 (t, *J* = 7.4 Hz, 3H). **^{13}C NMR** (125 MHz, $CDCl_3$) δ 171.6, 157.3, 155.0, 137.8, 136.6, 134.0, 126.5, 126.2, 122.6, 121.3, 116.5, 79.4, 44.3, 41.7, 39.7, 38.7, 36.8, 29.1, 28.6, 25.4, 23.7, 11.9. **HRMS** (**ESI**): Calcd. 448.2571 for $C_{25}H_{35}N_3NaO_3$ $(M+Na)^+$, found 448.2570.

Ethyl 5-(2-((2-methylquinolin-8-yl)amino)-2-oxoethyl)octanoate (9ad):

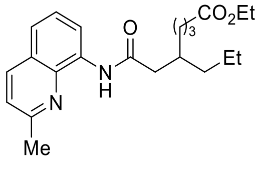

The title compound was prepared from **7c** (25 mg, 0.1 mmol) and ethyl 4-bromobutanoate (39 mg, 0.2 mmol) according to the general procedure. Purification using flash silica gel column chromatography (eluent: 10:1 hexanes:EtOAc) gave the pure product as a colorless oil (23 mg,61% yield). **^{1}H NMR** (500 MHz, $CDCl_3$) δ 9.85 (s, 1H), 8.73 (dd, *J* = 6.1, 2.9 Hz, 1H), 8.02 (d, *J* = 8.4 Hz, 1H), 7.47-7.43 (m, 2H), 7.31 (d, *J* = 8.4 Hz, 1H), 4.10 (q, *J* = 7.1 Hz, 2H), 2.74 (s, 3H), 2.50 (dd, *J* = 6.9, 1.8 Hz, 2H), 2.31 (t, *J* = 7.5 Hz, 2H), 2.11 (qd, *J* = 6.7, 3.4 Hz, 1H), 1.73 – 1.70 (m, 2H), 1.46 – 1.40 (m, 6H), 1.22 (t, *J* = 7.1 Hz, 3H), 0.93 – 0.90 (m, 3H). **^{13}C NMR** (125 MHz, $CDCl_3$) δ 173.8, 171.4, 157.3, 137.8, 136.6, 134.0, 126.5, 126.2, 122.5, 121.3, 116.5, 60.3, 43.1, 36.1, 35.1, 34.7, 33.4, 25.4, 22.2, 19.9, 14.5, 14.4. **HRMS** (**ESI**): Calcd. 393.2149 for $C_{22}H_{30}N_2NaO_3$ $(M+Na)^+$, found 393.2153.

3-(2-Hydroxyethyl)-*N*-(2-methylquinolin-8-yl)hexanamide (9ae):

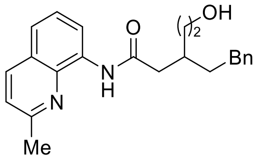

The title compound was prepared from **7d** (32 mg, 0.1 mmol) and 2-bromoethan-1-ol (25 mg, 0.2 mmol) according to the general procedure. Purification using flash silica gel column chromatography (eluent: 10:1 hexanes:EtOAc) gave the pure product as a colorless oil (21 mg, 58% yield). **^{1}H NMR** (500 MHz, $CDCl_3$) δ 9.99 (s, 1H), 8.74 (t, *J* = 4.5 Hz, 1H), 8.08 (d, *J* = 8.2 Hz, 1H), 7.50 (d, *J* = 4.5 Hz, 2H), 7.35 (d, *J* = 8.4 Hz, 1H), 7.30 – 7.26 (m, 2H), 7.23 – 7.18 (m, 3H), 3.84 – 3.75 (m, 2H), 2.78 – 2.74 (m, 6H), 2.68 – 2.63 (m, 1H), 2.40 – 2.32 (m, 1H), 1.91 – 1.87 (m, 1H), 1.81 (dt, *J* = 8.9, 7.0 Hz, 2H), 1.69 – 1.65 (m, 1H). **^{13}C NMR** (125 MHz, $CDCl_3$) δ 171.7, 157.5, 142.4, 137.9, 136.7, 133.8, 128.5, 128.5, 126.5, 126.3, 125.9, 122.6, 121.7, 117.1, 60.7, 42.7, 37.4, 37.2, 33.6, 32.3, 25.3. **HRMS** (**ESI**): Calcd. 385.1886 for $C_{23}H_{26}N_2NaO_2$ $(M+Na)^+$, found 385.1893.

3-Benzyl-*N*-(2-methylquinolin-8-yl)heptanamide (9af):

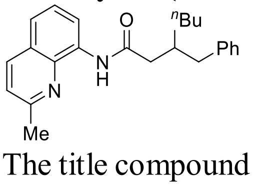

The title compound was prepared from **7e** (30 mg, 0.1 mmol) and 1-iodobutane (37 mg, 0.2 mmol) according to the general procedure. Purification using flash silica gel column chromatography (eluent: 15:1 hexanes:EtOAc) gave the pure product as a colorless oil (21 mg, 57% yield, r.r. 91:9). **^{1}H NMR** (400 MHz, $CDCl_3$) δ 9.74 (s, 1H), 8.66 (dd, *J* = 6.2, 2.8 Hz, 1H), 7.94 (d, *J* = 8.3 Hz, 1H), 7.37 – 7.34 (m, 2H), 7.24 – 7.17 (m, 5H), 7.12 – 7.07 (m, 1H), 2.72 – 2.59 (m, 5H), 2.40 (d, *J* = 7.2 Hz, 2H), 2.35 – 2.27 (m, 1H), 1.37 – 1.31 (m, 4H), 1.26 -1.18 (m, 2H), 0.79 (t, *J* = 7.2 Hz, 3H). **^{13}C NMR** (75 MHz, $CDCl_3$) δ 171.3, 157.2, 140.6, 137.8, 136.5, 134.0, 129.5, 128.4, 126.5, 126.2, 126.0, 122.5, 121.2, 116.5, 42.4, 40.4, 37.6, 33.4, 29.1, 25.4, 23.0, 14.2. **HRMS** (**ESI**): Calcd. 383.2094 for $C_{24}H_{28}N_2NaO$ $(M+Na)^+$, found 383.2099.

3-Benzyl-4-methyl-*N*-(2-methylquinolin-8-yl)pentanamide (9ag):

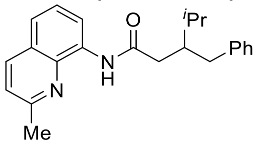

The title compound was prepared from **7e** (30 mg, 0.1 mmol) and 2-bromopropane (25mg, 0.2 mmol) according to the general procedure. Purification using flash silica gel column chromatography (eluent: 15:1 hexanes:EtOAc) gave the pure product as a colorless oil (21 mg, 60% yield). **^{1}H NMR** (500 MHz, $CDCl_3$) δ 9.83 (s, 1H), 8.70 (dd, *J* = 5.9, 3.2 Hz, 1H), 8.03 (d, *J* = 8.4 Hz, 1H), 7.45 – 7.43 (m, 2H), 7.32 (d, *J* = 8.4 Hz, 1H), 7.26 (d, *J* = 4.3 Hz, 4H), 7.17 – 7.13 (m, 1H), 2.79 – 2.75 (m, 4H), 2.62 (dd, *J* = 13.7, 7.5 Hz, 1H), 2.57 – 2.52 (m, 1H), 2.42– 2.37 (m, 2H), 1.89 (ddq, *J* = 10.0, 6.9, 3.4 Hz, 1H), 1.01 (d, *J* = 6.9 Hz, 3H), 1.00 (d, *J* = 6.9 Hz, 3H). **^{13}C NMR** (125 MHz, $CDCl_3$) δ 171.6, 157.2, 141.1, 137.8, 136.6, 134.0, 129.4, 128.4, 126.5, 126.2, 126.0, 122.5, 121.2, 116.5, 43.5, 39.1, 37.2, 29.0, 25.4, 19.5, 18.8. **HRMS** (**ESI**): Calcd. 369.1937 for $C_{23}H_{26}N_2NaO$ $(M+Na)^+$, found 369.1941.

3-Ethyl-*N*-(2-methylquinolin-8-yl)heptanamide (18a):

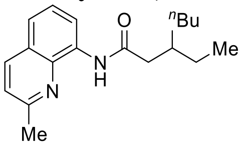

The title compound was prepared from **17a** (24 mg, 0.1 mmol) and 1-iodobutane (37 mg, 0.2 mmol) according to the general procedure. Purification using flash silica gel column chromatography (eluent: 15:1 hexanes:EtOAc) gave the pure product as a colorless oil (24 mg, 82% yield). **^{1}H NMR** (400 MHz, $CDCl_3$) δ 9.87 (s, 1H), 8.75 (dd, *J* = 6.4, 2.6 Hz, 1H), 8.02 (d, *J* = 8.4 Hz, 1H), 7.47 – 7.42 (m, 2H), 7.31 (d, *J* = 8.4 Hz, 1H), 2.74 (s, 3H), 2.49 (d, *J* = 7.0 Hz, 2H), 2.06 – 1.97 (m, 1H), 1.51 – 1.30 (m, 8H), 0.96 (t, *J* = 7.5 Hz, 3H), 0.92 – 0.86 (m, 3H). **^{13}C NMR** (100 MHz, $CDCl_3$) δ 171.8, 157.2, 137.8, 136.5, 134.1, 126.5, 126.2, 122.5, 121.2, 116.5, 43.0, 37.0, 33.2, 29.0, 26.5, 25.4, 23.1, 14.2, 11.1. **HRMS** (**ESI**): Calcd. 321.1937 for $C_{19}H_{26}N_2NaO$ $(M+Na)^+$, found321.1943.

3-Ethyl-7-(4-hydroxyphenoxy)-*N*-(2-methylquinolin-8-yl)heptanamide (18b):

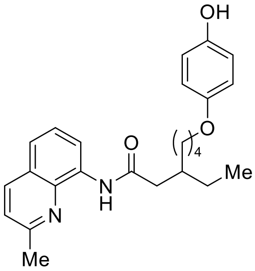

The title compound was prepared from **17a** (24 mg, 0.1 mmol) and 4-(4-bromobutoxy)phenol (49 mg, 0.2 mmol) according to the general procedure. Purification using flash silica gel column chromatography (eluent: 5:1 hexanes:EtOAc) gave the pure product as a colorless solid (27 mg, 66% yield). **^{1}H NMR** (500 MHz, DMSO-d_6) δ 9.92 (s, 1H), 8.86 (s, 1H), 8.58 (dd, *J* = 7.6, 1.4 Hz, 1H), 8.26 (d, *J* = 8.5 Hz, 1H), 7.59 (dd, *J* = 8.2, 1.4 Hz, 1H), 7.50 – 7.46 (m, 2H), 6.69 – 6.61 (m, 4H), 3.81 (t, *J* = 6.5 Hz, 2H), 2.72 (s, 3H), 2.52 – 2.49 (m, 2H), 1.94 – 1.85 (m, 1H), 1.67 – 1.62 (m, 2H), 1.48 – 1.32 (m, 6H), 0.90 (t, *J* = 7.4 Hz, 3H). **^{13}C NMR** (125 MHz, DMSO-d_6) δ 171.5, 157.9, 151.9, 151.5, 137.8, 137.1, 134.3, 126.4, 126.4, 123.3, 121.9, 116.8, 116.1, 115.8, 68.3, 41.9, 36.6, 33.1, 29.6, 26.2, 25.4, 23.1, 11.2. **HRMS** (**ESI**): Calcd. 429.2149 for$C_{25}H_{30}N_2NaO_3$ $(M+Na)^+$, found 429.2145.

***N*-(2-Methylquinolin-8-yl)-3-(tetrahydro-2*H*-pyran-4-yl)pentanamide (18c):**

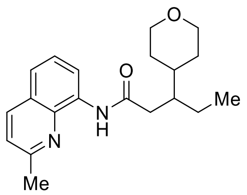

The title compound was prepared from **17a** (24 mg, 0.1 mmol) and 4-bromotetrahydro -2*H*-pyran (33 mg, 0.2 mmol) according to the general procedure. Purification using flash silica gel column chromatography (eluent: 10:1 hexanes:EtOAc) gave the pure product as a colorless oil (20 mg, 60% yield). **^{1}H NMR** (500 MHz, $CDCl_3$) δ 9.88 (s, 1H), 8.73 (dd, *J* = 5.9, 3.2 Hz, 1H), 8.03 (d, *J* = 8.3 Hz, 1H), 7.46-7.44 (m, 2H), 7.32 (d, *J* = 8.4 Hz, 1H), 4.00 (dd, *J* = 11.5, 4.4 Hz, 2H), 3.37 (td, *J* = 11.7, 2.3 Hz, 2H), 2.74 (s, 3H), 2.59 (dd, *J* = 14.9, 6.1 Hz, 1H), 2.45 (dd, *J* = 14.8, 7.3 Hz, 1H), 1.94 (ddt, *J* = 12.8, 7.1, 5.5 Hz, 1H), 1.77 – 1.72 (m, 1H), 1.62 – 1.47 (m, 6H), 0.98 (t, *J* = 7.4 Hz, 3H). **^{13}C NMR** (125 MHz, $CDCl_3$) δ 171.6, 157.3, 137.8, 136.6, 134.0, 126.5, 126.2, 122.5, 121.3, 116.5, 68.5, 41.8, 39.5, 37.6, 30.1, 29.9, 25.4, 23.5, 11.7. **HRMS** (**ESI**): Calcd. 349.1886 for $C_{20}H_{26}N_2NaO_2$ $(M+Na)^+$, found 349.1896.

***N*-(2-Methylquinolin-8-yl)-3-propylheptanamide (18d):**

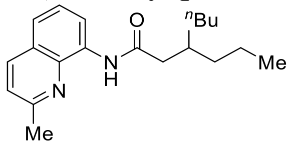

The title compound was prepared from **17b** (25 mg, 0.1 mmol) and 1-iodobutane (37 mg, 0.2 mmol) according to the general procedure. Purification using flash silica gel column chromatography (eluent: 15:1 hexanes:EtOAc) gave the pure product as a colorless oil (20 mg, 63% yield). The title compound could also be prepared from **4i** (25 mg, 0.1 mmol) and 1-iodobutane (37 mg, 0.2 mmol) according to the general procedure. Purification using flash silica gel column chromatography (eluent: 15:1 hexanes:EtOAc) gave the pure product as a colorless oil (22 mg, 71% yield). **^{1}H NMR** (500 MHz, $CDCl_3$) δ 9.86 (s, 1H), 8.75 (dd, *J* = 6.6, 2.4 Hz, 1H), 8.03 (d, *J* = 8.4 Hz, 1H), 7.48 – 7.42 (m, 2H), 7.31 (d, *J* = 8.4 Hz, 1H), 2.74 (s, 3H), 2.49 (d, *J* = 7.0 Hz, 2H), 2.14 – 2.01 (m, 1H), 1.44 – 1.29 (m, 10H), 0.93 – 0.87 (m, 6H). **^{13}C NMR** (100 MHz, $CDCl_3$) δ 171.8, 157.2, 137.8, 136.6, 134.1, 126.5, 126.2, 122.5, 121.2, 116.5, 43.4, 36.4, 35.4, 33.7, 29.0, 25.4, 23.1, 20.0, 14.5, 14.3. **HRMS** (**ESI**): Calcd. 335.2094 for $C_{20}H_{28}N_2NaO$ $(M+Na)^+$, found 335.2099.

(1*R*,2*S*)-2-Butyl-*N*-(2-methylquinolin-8-yl)cyclopentane-1-carboxamide (18e):

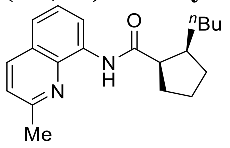

The title compound was prepared from **17c** (25 mg, 0.1 mmol) and 1-iodobutane (37 mg, 0.2 mmol) according to the general procedure. Purification using flash silica gel column chromatography (eluent: 15:1 hexanes:EtOAc) gave the pure product as a colorless oil (15 mg, 47% yield). ^{1}H NMR (500 MHz, $CDCl_3$) δ 9.87 (s, 1H), 8.74 (dd, *J* = 6.7, 2.4 Hz, 1H), 8.03 (d, *J* = 8.4 Hz, 1H), 7.47 – 7.42 (m, 2H), 7.31 (d, *J* = 8.3 Hz, 1H), 3.01 (td, *J* = 7.7, 5.6 Hz, 1H), 2.74 (s, 3H), 2.24 – 2.12 (m, 2H), 2.01 – 1.94 (m, 2H), 1.90 – 1.85 (m, 1H), 1.70 – 1.62 (m, 2H), 1.36 – 1.20 (m, 6H), 0.79 (t, *J* = 7.2 Hz, 3H). **^{13}C NMR** (125 MHz, $CDCl_3$) δ 174.1, 157.2, 138.0, 136.5, 134.2, 126.6, 126.2, 122.5, 121.1, 116.4, 51.2, 44.5, 31.3, 31.3, 30.7, 28.7, 25.4, 24.1, 22.9, 14.2. **HRMS**

(**ESI**): Calcd.333.1937 for $C_{20}H_{26}N_2NaO$ $(M+Na)^+$, found 333.1931.

3-Cyclopentyl-*N*-(2-methylquinolin-8-yl)hexanamide (18f):

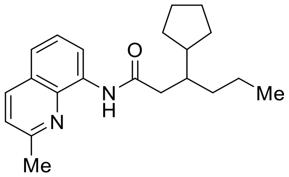

The title compound was prepared from **17d** (25 mg, 0.1 mmol) and bromocyclopentane (30 mg, 0.2 mmol) according to the general procedure. Purification using flash silica gel column chromatography (eluent: 15:1 hexanes:EtOAc) gave the pure product as a colorless oil (21 mg, 65% yield). **^{1}H NMR** (500 MHz, $CDCl_3$) δ 9.88 (s, 1H), 8.74 (dd, J = 6.7, 2.3 Hz, 1H), 8.03 (d, J = 8.4 Hz, 1H), 7.47 – 7.42 (m, 2H), 7.32 (d, J = 8.4 Hz, 1H), 2.74 (s, 3H), 2.58 (dd, J = 14.5, 5.6 Hz, 1H), 2.49 (dd, J = 14.5, 7.2 Hz, 1H), 2.02 – 1.78 (m, 5H), 1.65 – 1.47 (m, 9H), 0.92 – 0.89 (m, 3H). **^{13}C NMR** (100 MHz, $CDCl_3$) δ 172.0, 157.2, 137.9, 136.6, 134.2, 126.6, 126.2, 122.5, 121.1, 116.5, 44.1, 41.8, 40.4, 35.1, 30.4, 25.6, 25.6, 25.4, 20.0, 14.7. **HRMS** (**ESI**): Calcd.347.2094 for $C_{21}H_{28}N_2NaO$ $(M+Na)^+$, found 347.2095.

3-Butyl-*N*-(2-methylquinolin-8-yl)heptanamide (18g):

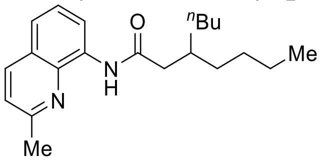

The title compound was prepared from **17e** (27 mg, 0.1 mmol) and 1-iodobutane (92 mg, 0.5 mmol) according to the general procedure. Purification using flash silica gel column chromatography (eluent: 15:1 hexanes:EtOAc) gave the pure product as a colorless oil (19.5 mg, 60% yield). **^{1}H NMR** (400 MHz, $CDCl_3$) δ 9.87 (s, 1H), 8.75 (dd, J = 6.5, 2.5 Hz, 1H), 8.03 (d, J = 8.1 Hz, 1H), 7.49-7.40 (m, 2H), 7.31 (d, J = 8.4 Hz, 1H), 2.75 (s, 3H), 2.49 (d, J = 6.9 Hz, 2H), 2.09 – 2.01 (m, 1H), 1.43 – 1.30 (m, 12H), 0.89 (t, J = 7.0 Hz, 6H). **^{13}C NMR** (100 MHz, $CDCl_3$) δ 171.8, 157.2, 137.8, 136.6, 134.1, 126.6, 126.2, 122.5, 121.2, 116.5, 43.4, 35.6, 33.7, 29.0, 25.3, 23.1, 14.3. **HRMS** (**ESI**): Calcd. 349.2250 for $C_{21}H_{30}N_2NaO$ $(M+Na)^+$, found 349.2253.

Regioconvergent hydroalkylation

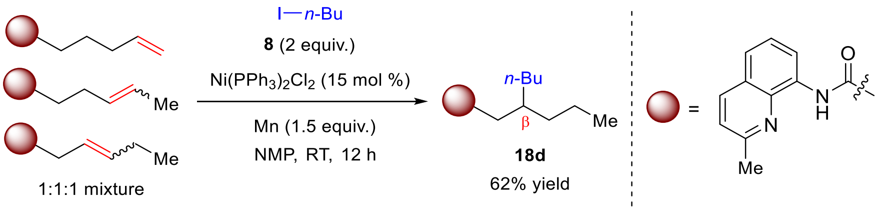

In a N_2-filled glovebox, to an oven-dried 5 mL vial equipped with a magnetic stir bar were added alkene substrate **7c** (25 mg, 0.1 mmol), **17b** (25 mg, 0.1 mmol), **17d** (25 mg, 0.1 mmol), $Ni(PPh_3)_2Cl_2$ (29 mg, 0.045 mmol), Mn powder (25 mg, 0.45 mmol) and dry NMP (1 mL). sequentially. The vial was tightly capped and removed from the glovebox followed by addition of 1-iodobutane **8** (110 mg, 0.6 mmol) by a micro-syringe. The mixture was allowed to vigorously stir at ambient temperature for 12 h. When alkene was almost fully consumed (monitored by GC-MS), the mixture was directly subjected to flash silica gel column chromatography (eluent: 10:1

hexanes:EtOAc) to afford the pure product **18d** (61 mg, 62% yield).

2.4 Application to Synthesis of Biologically Active Molecules

A:

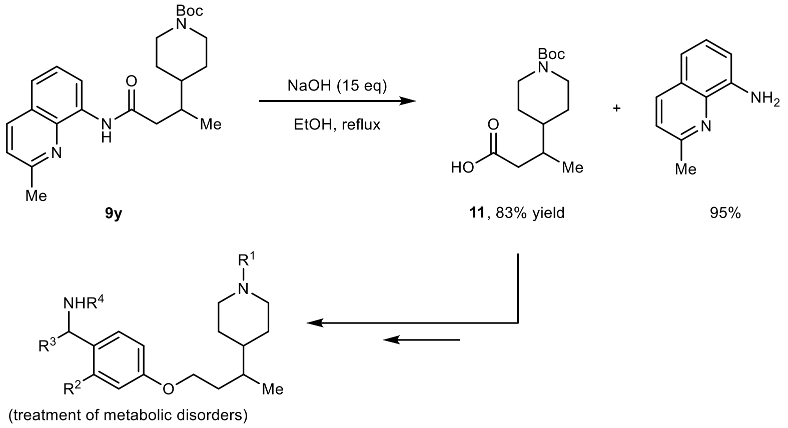

The directing group was removed by the following literature procedure[4]. To a flame-dried 50 mL sealed vessel equipped with a stir bar were added *tert*-butyl 4-(4-((2-methylquinolin-8-yl)amino)-4-oxobutan-2-yl)piperidine-1-carboxylate (**9y**, 41 mg, 0.1 mmol), NaOH (60 mg, 15 eq) and EtOH (2 mL). The resulting mixture was allowed to stir at 130 °C for 16 h, after which the reaction mixture was allowed to cool to RT, diluted with 20 ml EtOAc and washed with HCl (1 M, 3×3 mL). The organic layer was dried over Na_2SO_4 and concentrated under reduced pressure. The residue was purified by flash column chromatography on silica gel (eluent: 2:1 hexanes:EtOAc) to afford 3-(1-(*tert*-butoxy carbonyl)piperidin-4-yl) butanoic acid.

3-(1-(*tert*-Butoxycarbonyl)piperidin-4-yl)butanoic acid (11):

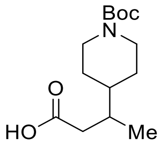

A colorless oil, 85% yield. **^{1}H NMR** (500 MHz, DMSO-d_6) δ 12.02 (s, 1H), 3.96 (brs, 2H), 2.60 (brs, 2H), 2.29 (dd, J = 15.1, 5.1 Hz, 1H), 2.00 – 1.93 (m, 1H), 1.80 – 1.70 (m, 1H), 1.58 – 1.51 (m, 2H), 1.38 (s, 9H), 1.12 – 0.92 (m, 3H), 0.84 (d, J = 6.8 Hz, 3H). **^{13}C NMR** (125 MHz, $CDCl_3$) δ177.9, 155.0, 79.5, 44.3, 44.3, 41.0, 38.8, 34.7, 29.5, 28.6, 28.2, 16.6. **HRMS (ESI)**: Calcd. 294.1676 for $C_{14}H_{25}NNaO_4$ $(M+Na)^+$, found 294.1673.

B:

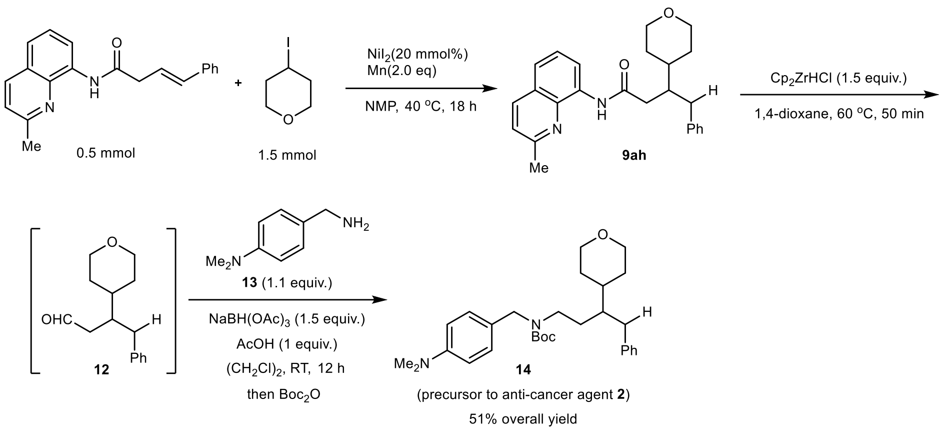

In a N_2-filled glovebox, to an oven-dried 5 mL vial equipped with a magnetic stir bar were added alkene substrate **7e** (151 mg, 0.5 mmol), NiI_2 (31.2 mg, 0.1 mmol), Mn powder (55 mg, 1.0 mmol) and dry NMP (2 mL) sequentially. The vial was tightly capped and removed from the glovebox followed by addition of 4-iodotetrahydro-*2H*-pyran (318 mg, 1.5 mmol) by a micro-syringe. The mixture was allowed to vigorously stir at 40 °C for 18 h. The mixture was then directly subjected to flash silica gel column chromatography (eluent: 5:1 hexanes:EtOAc) to afford the pure product **9ah** (83 mg, 43% yield, 96:4 r.r.).

In a N_2-filled glovebox, to an oven-dried 5 mL vial equipped with a magnetic stir bar were added **9ah** (0.1 mmol), Cp_2ZrHCl (0.15 mmol) and dry 1,4-dioxane (1.0 mL). The vial was tightly capped and removed from the glovebox. The mixture was allowed to stir at 60 °C for 50 min, after which the solution was cooled to RT, diluted with Et_2O (5 mL) and transferred to 25 mL round-bottom flask. To the mixture was added 5 mL aq. HCl (2 M). The mixture was allowed to stir for 30 min. The organic phase was separated and the aqueous phase was washed with Et_2O (5 mL). The combined organic layers were dried over anhydrous Na_2SO_4 and concentrated to a small volume (about 1 mL). The residual solution was passed through a short pad of silica gel and eluted with 1:1 hexanes:EtOAc. The solution was collected and concentrated, and the resulting aldehyde **12** (75% yield) was used directly in the next step without further purification.

Under a N_2 atmosphere, **12** was dissolved in dry $(CH_2Cl)_2$ (1 mL). Benzyl amine **13** (0.083 mmol, 1.1 eq) and AcOH (1.0 eq) were subsequently added. The mixture was allowed to stir for 10 min followed by the addition of $NaBH(OAc)_3$ (1.5 eq). The resulting mixture was allowed to stir for another 12 h, after which the mixture was quenched with saturated $NaHCO_3$ (5 mL) and washed with EtOAc (2×5 mL). The combined organic layers were dried over anhydrous Na_2SO_4 and concentrated under reduced pressure to afford the crude amine product.

The crude amine was dissolved in *t*BuOH (0.5 mL), then 0.3 mL aq. NaOH (1 M) and Boc_2O (2.0 eq) were added. The mixture was allowed to stir at RT for 4 h, after which the mixture was diluted with EtOAc (5 mL) and H_2O (5 mL). The organic phase was separated and the aqueous phase was washed with EtOAc (5 mL). The combined organic layers were dried over anhydrous Na_2SO_4 and concentrated. The residue was purified by silica gel flash chromatography to give the pure product **14** (23.7 mg, 51% yield over three steps).

***N*-(2-Methylquinolin-8-yl)-4-phenyl-3-(tetrahydro-2*H*-pyran-4-yl)butanamide (9ah)**:

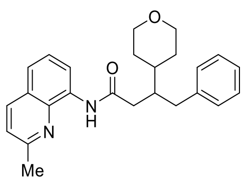

^1H NMR (300 MHz, $CDCl_3$) δ 9.84 (s, 1H), 8.76 – 8.72 (m, 1H), 8.08 (d, *J* = 8.3 Hz, 1H), 7.49 (d, *J* = 4.4 Hz, 2H), 7.36 (d, *J* = 8.4 Hz, 1H), 7.33 – 7.28 (m, 4H), 7.25 – 7.18 (m, 1H), 4.05 (d, *J* = 11.1 Hz, 2H), 3.40 (tdd, *J* = 11.3, 3.9, 2.5 Hz, 2H), 2.89 (dd, *J* = 13.7, 6.2 Hz, 1H), 2.79 (s, 3H), 2.67 (dd, *J* = 13.7, 8.1 Hz, 1H), 2.56 (dd, *J* = 18.8, 6.1 Hz, 1H), 2.46 – 2.40 (m, 1H), 1.91 – 1.79 (m, 2H), 1.76 – 1.55 (m, 4H); ^{13}C NMR (126 MHz, $CDCl_3$) δ 171.1, 157.3, 140.7, 137.7, 136.6, 133.9, 129.3, 128.6, 128.6, 126.5, 126.2, 122.5, 121.3, 116.5, 68.4, 42.3, 38.8, 37.3, 37.1, 30.1, 29.8, 25.4 ppm. **HRMS** (**ESI**): Calcd. 389.2224 for $C_{25}H_{29}N_2O_2^+$ (M+H)$^+$, found 389.2235.

***tert*-Butyl (4-(dimethylamino)benzyl)(4-phenyl-3-(tetrahydro-2*H*-pyran-4-yl) butyl) carbamate** (**14**):

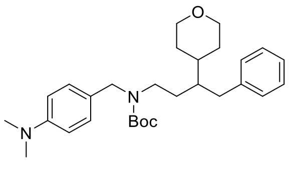

^1H NMR (400 MHz, $CDCl_3$) δ 7.30 – 7.26 (m, 2H), 7.19 (t, *J* = 7.3 Hz, 1H), 7.12 (d, *J* = 6.4 Hz, 2H), 7.02 (s, 2H), 6.69 (d, *J* = 6.8 Hz, 2H), 4.18 – 4.13 (m, 2H), 3.97 (t, *J* = 9.5 Hz, 2H), 3.35 – 3.27 (m, 2H), 3.22 – 3.02 (m, 2H), 2.93 (s, 6H), 2.66 (dd, *J* = 13.6, 5.2 Hz, 1H), 2.42 (d, *J* = 40.8 Hz, 1H), 1.76 – 1.64 (m, 2H), 1.50 – 1.39 (m, 15H); ^{13}C NMR (126 MHz, $CDCl_3$) δ 155.8, 149.7, 141.2, 129.1, 128.4, 128.3, 126.1, 125.8, 112.7, 79.3, 68.4, 49.1(48.9), 44.2(43.9), 42.6, 40.8, 37.1, 37.0, 1.5(31.4), 30.0(29.7), 29.5(29.2), 28.5, 27.8(27.5) ppm(two rotating isomers). **HRMS** (**ESI**): Calcd. 467.3268 for $C_{29}H_{43}N_2O_3^+$ (M+H)$^+$, found 467.3282.

C:

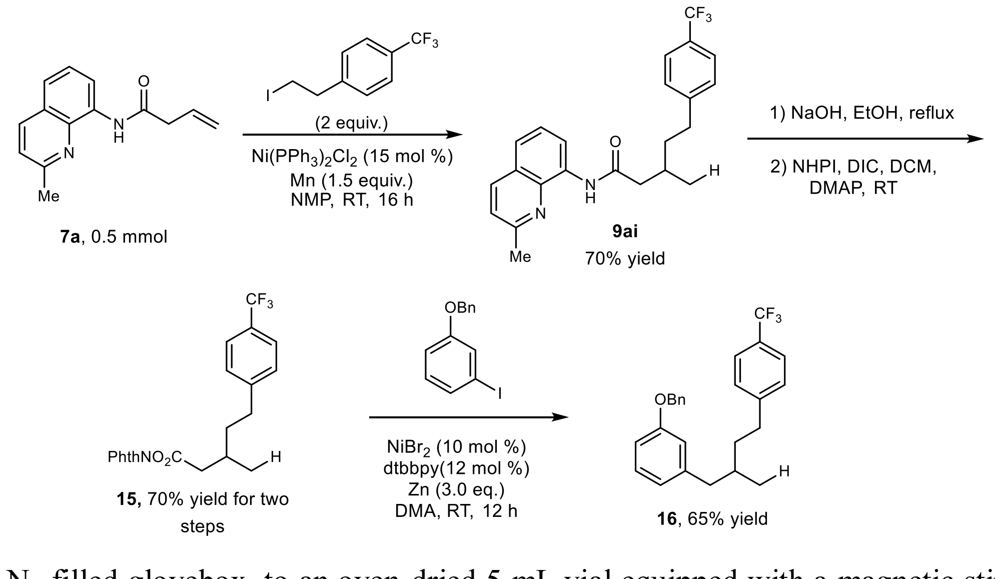

In a N_2-filled glovebox, to an oven-dried 5 mL vial equipped with a magnetic stir bar were added alkene substrate **7a** (113 mg, 0.5 mmol), $Ni(PPh_3)_2Cl_2$ (49 mg, 0.075 mmol),

Mn powder (41 mg, 0.75 mmol) and dry NMP (2 mL) sequentially. The vial was tightly capped and removed from the glovebox followed by addition of 1-(2-iodoethyl)-4-(trifluoromethyl)benzene (300 mg, 1.0 mmol) by a micro-syringe. The mixture was allowed to vigorously stir at ambient temperature for 12 h. When alkene was almost fully consumed (monitored by GC-MS), the mixture was directly subjected to flash silica gel column chromatography (eluent: 10:1 hexanes:EtOAc) to afford the pure product **9ai** (140 mg, 70% yield).

To a flame-dried 50 mL sealed vessel equipped with a stir bar were added 3-methyl-*N*-(2-methylquinolin-8-yl)-5-(4-(trifluoromethyl)phenyl)pentanamide (**9ai**, 140 mg, 0.35 mmol), NaOH (210 mg, 15 eq) and EtOH (5 mL). The resulting mixture was allowed to stir at 130 °C for 16 h, after which the reaction mixture was allowed to cool to RT, diluted with 20 ml EtOAc and washed with HCl (1M, 3×5 mL). The organic layer was dried over Na_2SO_4 and concentrated under reduced pressure. The residue was purified by flash column chromatography on silica gel (eluent: 3:1 hexanes:EtOAc) to afford 3-methyl-5-(4-(trifluoromethyl)phenyl) pentanoic acid (77.4 mg, 85% yield).

The acid prepared above (68 mg, 0.26 mmol), 2-hydroxyisoindoline-1,3-dione (NHPI, 46 mg, 1.1eq), DIC (39 mg, 1.2 eq) and DMAP (2 mg) were added sequentially in a 10 mL round-bottomed flask containing DCM (3 mL). The reaction mixture was allowed to stir at ambient temperature for 1 h. The white precipitate was filtered and washed with Et_2O, and the resulting solution was concentrated under reduced pressure. The residue was purified by flash column chromatography (eluent: 6:1 hexanes:EtOAc) to afford NHPI ester **15** (83 mg, 81% yield).

In a N_2-filled glovebox, to an oven-dried 5 mL vial equipped with a magnetic stir bar were added **15** (27 mg, 0.065 mmol), $NiBr_2$(diglyme) (1.8 mg, 0.005 mmol), 4,4'-di-*tert*-butyl-2,2'-bypyridine (dtbbpy, 1.7 mg, 0.006 mmol), Zn powder (10 mg, 0.15 mmol) and dry DMA (0.2 mL) sequentially. The vial was tightly capped and removed from the glovebox followed by addition of 1-(benzyloxy)-3-iodobenzene (16 mg, 0.05 mmol) by a micro-syringe. The mixture was allowed to vigorously stir at ambient temperature for 12 h. When 1-(benzyloxy)-3-iodobenzene was almost fully consumed (monitored by GC-MS), the mixture was directly subjected to flash silica gel column chromatography (eluent: 50:1 hexanes:EtOAc) to afford **16** (13.0 mg, 65% yield).

3-Methyl-*N*-(2-methylquinolin-8-yl)-5-(4-(trifluoromethyl)phenyl)pentanamide (9ai):

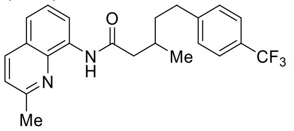

^{1}H NMR (500 MHz, $CDCl_3$) δ 9.85 (s, 1H), 8.75 (dd, J = 5.7, 3.2 Hz, 1H), 8.03 (d, J = 8.4 Hz, 1H), 7.50 (d, J = 8.1 Hz, 2H), 7.47 – 7.45 (m, 2H), 7.31 (t, J = 8.5 Hz, 3H), 2.84 – 2.78 (m, 1H), 2.70 – 2.70 (m, 1H), 2.72 (s, 3H), 2.61 (dd, J = 14.3, 6.3 Hz, 1H), 2.44 (dd, J = 14.3, 7.8 Hz, 1H), 2.30 – 2.20 (m, 1H), 1.87 – 1.80 (m, 1H), 1.66 – 1.59 (m, 1H), 1.14 (d, J = 6.7 Hz, 3H); ^{13}C NMR (126 MHz, $CDCl_3$) δ 170.8, 157.2, 146.5, 137.6, 136.4, 133.8, 128.6, 128.1 (q, J = 32.1 Hz), 126.3, 126.0, 125.2 (q, J = 3.5 Hz), 124.3 (q, J = 252.0 Hz), 122.4, 121.2, 116.4, 45.7, 38.2, 33.3, 30.5, 25.2, 19.7; ^{19}F NMR (471 MHz, $CDCl_3$) δ -62.28 (s, 3F) ppm. **HRMS** (**ESI**): Calcd. 401.1835 for $C_{23}H_{24}F_3N_2O^+$ $(M+H)^+$, found 401.1834.

1,3-Dioxoisoindolin-2-yl 3-methyl-5-(4-(trifluoromethyl)phenyl)pentanoate (15):

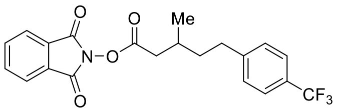
^{1}H NMR (400 MHz, $CDCl_3$) δ 7.89 (dd, *J* = 5.5, 3.1 Hz, 2H), 7.79 (dd, *J* = 5.5, 3.1 Hz, 2H), 7.54 (d, *J* = 8.1 Hz, 2H), 7.33 (d, *J* = 7.9 Hz, 2H), 2.82 – 2.69 (m, 3H), 2.57 (dd, *J* = 15.1, 7.4 Hz, 1H), 2.21 – 2.12 (m, 1H), 1.90 – 1.81 (m, 1H), 1.70 – 1.60 (m, 1H), 1.16 (d, *J* = 6.7 Hz, 3H); **^{13}C NMR** (101 MHz, $CDCl_3$) δ 168.8, 162.1, 146.2, 134.9, 129.1, 128.8, 128.4 (q, *J* = 32.1 Hz), 125.5 (q, *J* = 3.7 Hz), 124.5 (q, *J* = 273.7 Hz), 124.1, 38.3, 37.8, 33.2, 30.4, 19.6. **^{19}F NMR** (377 MHz, $CDCl_3$) δ -62.27 (s, 3F) ppm. **HRMS** (**ESI**): Calcd. 428.1080 for $C_{21}H_{18}F_3NNaO_4^+$ $(M+Na)^+$, found 428.1087.

1-(Benzyloxy)-3-(2-methyl-4-(4-(trifluoromethyl)phenyl)butyl)benzene (**16**):

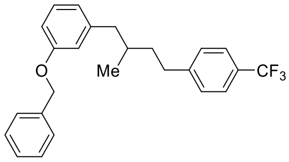

^{1}H NMR (500 MHz, $CDCl_3$) δ 7.51 (d, *J* = 8.0 Hz, 2H), 7.43 (d, *J* = 7.3 Hz, 2H), 7.38 (t, *J* = 7.4 Hz, 2H), 7.32 (t, *J* = 7.2 Hz, 1H), 7.25 (d, *J* = 8.1 Hz, 2H), 7.19 (t, *J* = 7.8 Hz, 2H), 6.81 (dd, *J* = 8.0, 2.3 Hz, 1H), 6.76 (d, *J* = 2.1 Hz, 1H), 6.74 (d, *J* = 7.6 Hz, 1H), 5.04 (s, 2H), 2.78 – 2.72 (m, 1H), 2.66 – 2.60 (m, 2H), 2.42 (dd, *J* = 13.4, 7.9 Hz, 1H), 1.80 – 1.74 (m, 1H), 1.72 – 1.65 (m, 1H), 1.49 – 1.45 (m, 1H), 0.93 (d, *J* = 6.6 Hz, 3H); ^{13}C NMR (126 MHz, $CDCl_3$) δ 158.9, 147.0, 142.9, 137.3, 129.3, 128.8, 128.7, 128.1, 127.7, 125.3 (q, *J* = 3.9 Hz), 124.6 (q, *J* = 279.1 Hz), 122.0, 116.1, 112.1, 70.1, 43.7, 38,2, 34.6, 33.5, 19.6; ^{19}F NMR (471 MHz, $CDCl_3$) δ -62.25 (s, 3F) ppm. **HRMS** (**ESI**): Calcd. 399.1930 for $C_{25}H_{26}F_3O^+$ $(M+H)^+$, found 399.1924.

2.5 Mechanistic Studies

Control experiment to preclude the intermediacy of α,β-unsaturated amide 7a'

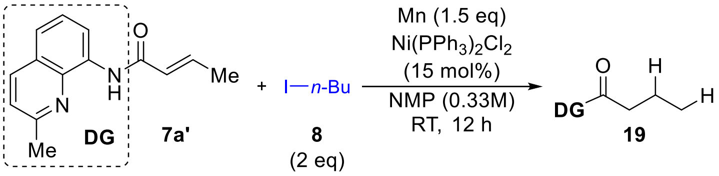

In a N_2-filled glovebox, to an oven-dried 5 mL vial equipped with a magnetic stir bar were added (*E*)-*N*-(2-methylquinolin-8-yl)but-2-enamide **7a'** (23 mg, 0.1 mmol), $Ni(PPh_3)_2Cl_2$ (10 mg, 0.015 mmol) and Mn powder (0.15 mmol). The mixture was then dissolved in dry NMP (0.3 mL). The vial was tightly capped and removed from the glovebox followed by addition of 1-iodobutane **8** (37 mg, 0.2 mmol) by a micro-syringe. The mixture was allowed to vigorously stir at ambient temperature for 12 h. When alkene was almost fully consumed (monitored by GC-MS), the mixture was directly subjected to flash silica gel column chromatography to afford the hydrogenation product **19** as colorless oil (14 mg, 61% yield).

(*E*)-*N*-(2-methylquinolin-8-yl)but-2-enamide (7a'):

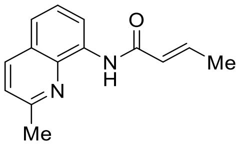

A colorless solid. **^{1}H NMR** (300 MHz, $CDCl_3$) δ 9.88 (s, 1H), 8.80 (dd, *J* = 6.4, 2.6 Hz, 1H), 8.02 (d, *J* = 8.4 Hz, 1H), 7.50 – 7.42 (m, 2H), 7.31 (d, *J* = 8.4 Hz, 1H), 7.06 (dq, *J* = 15.0, 6.9 Hz, 1H), 6.29 – 6.16 (m, 1H), 2.75 (s, 3H), 1.97 (dd, *J* = 6.9, 1.7 Hz, 3H). **^{13}C NMR** (75 MHz, $CDCl_3$) δ 164.3, 157.3, 141.1, 136.6, 134.2, 126.5, 126.3, 126.2, 122.5, 121.4, 116.8, 25.4, 18.0. **HRMS (ESI)**: Calcd. 249.0998 for $C_{14}H_{14}N_2NaO$ $(M+Na)^+$, found 249.1001.

_N_-(2-Methylquinolin-8-yl)butyramide (19):

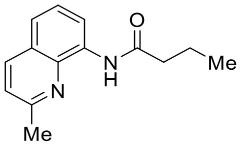

A colorless oil, 61% yield. **^{1}H NMR** (300 MHz, $CDCl_3$) δ 9.87 (s, 1H), 8.75 (dd, *J* = 6.0, 3.0 Hz, 1H), 8.03 (d, *J* = 8.3 Hz, 1H), 7.49 – 7.42 (m, 2H), 7.31 (d, *J* = 8.4 Hz, 1H), 2.74 (s, 3H), 2.55 (t, *J* = 7.5 Hz, 2H), 1.85 (dt, *J* = 14.8, 7.4 Hz, 2H), 1.07 (t, *J* = 7.4 Hz, 3H). **^{13}C NMR** (75 MHz, $CDCl_3$) δ 171.8, 157.3, 137.8, 136.6, 134.1, 126.5, 126.2, 122.5, 121.2, 116.5, 40.3, 25.4, 19.2, 14.0. **HRMS (ESI)**: Calcd. 251.1155 for $C_{14}H_{16}N_2NaO$ $(M+Na)^+$, found 251.1157.

Deuterium labeling experiment

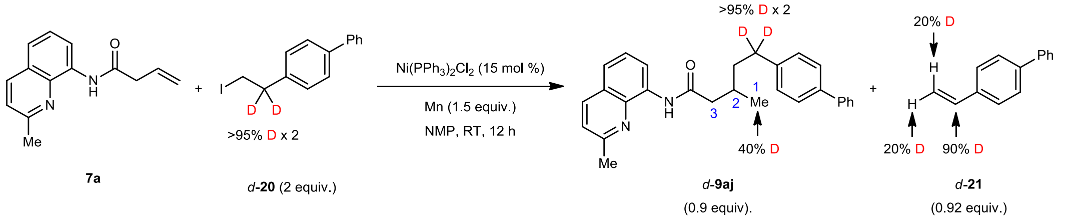

In a N_2-filled glovebox, to an oven-dried 5 mL vial equipped with a magnetic stir bar were added alkene substrate **7a** (23 mg, 0.1 mmol), deuterated iodide *d*-**20** (62 mg, 0.2 mmol), $Ni(PPh_3)_2Cl_2$ (10 mg, 0.015 mmol) and Mn powder (0.15 mmol). The mixture was then dissolved in dry NMP (0.3 mL). The vial was tightly capped and removed from the glovebox. The mixture was allowed to vigorously stir at ambient temperature for 12 h. When alkene was almost fully consumed (monitored by GC-MS), the mixture was directly subjected to flash silica gel column chromatography to afford the β-H elimination product *d*-**21**(a colorless solid, 0.92 mmol) and hydroalkylation product *d*-**9aj** (a colorless solid, 0.9 mmol).

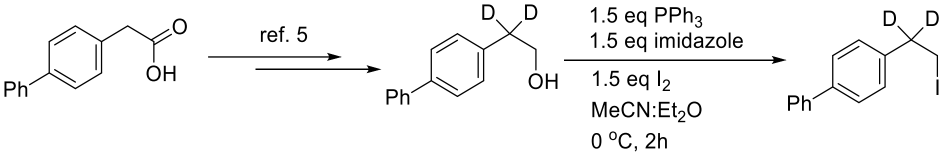

Procedure for the synthesis of *d*-20: 2-([1,1'-Biphenyl]-4-yl)ethanol-d_2 was synthesized according to a literature procedure[5]. Triphenylphosphine (1.18 g, 4.5 mmol) and imidazole (306 mg, 4.5 mmol) were dissolved in Et_2O (6 mL) and acetonitrile (6

mL). The mixture was cooled to 0°C and iodine (1.14 g, 4.5 mmol) was added in one portion. The mixture was allowed to vigorously stir for 20 min. The resulting yellow slurry was warmed to RT for 10 min and then cooled to 0°C again. 2-([1,1'-Biphenyl]-4-yl)ethanol-d_2 (600 mg, 3 mmol) was dissolved in Et_2O (6 mL) and the resulting solution was added dropwise via syringe over a period of 10 min. The reaction was warmed to RT and allowed to stir overnight. The reaction was quenched by addition of a saturated solution of sodium thiosulfate and extracted 3 times with DCM. The combined organic layers were washed with brine, dried over $MgSO_4$, filtered, and concentrated under reduced pressure. The crude residue was dissolved in a minimal amount of DCM and triphenylphosphine oxide was precipitated upon slow addition of pentane. The mixture was filtered and the filtrate was concentrated. The crude product was purified by flash silica chromatography to afford ***d*-20**.

4-(2-Iodoethyl-1,1-*d*$_2$)-1,1'-biphenyl (*d*-20):

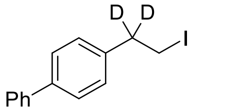

A colorless solid. 62% yield. **^{1}H NMR** (500 MHz, $CDCl_3$) δ 7.58 (dd, *J* = 17.1, 7.7 Hz, 4H), 7.44 (t, *J* = 7.6 Hz, 2H), 7.36 (d, *J* = 7.1 Hz, 1H), 7.28 (d, *J* = 8.2 Hz, 2H), 3.37 (s, 2H). **^{13}C NMR** (125 MHz, $CDCl_3$) δ 140.9, 140.0, 139.7, 128.9, 128.9, 127.5, 127.4, 127.2, 5.3. ^{2}D NMR (77 MHz, $CHCl_3$) δ 3.17 (s, 2D) ppm. **HRMS** (**EI**): Calcd. 310.0182 for $C_{14}H_{11}D_2I$ (M), found 310.0186.

4-(vinyl-1-*d*)-1,1'-biphenyl (*d*-21)[6,7]:

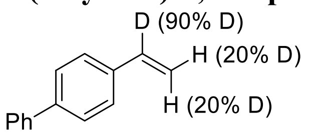

A colorless solid, 92% yield. **^{1}H NMR** (500 MHz, $CDCl_3$) δ 7.60 (ddd, *J* = 15.5, 7.4, 1.5 Hz, 4H), 7.50 (d, *J* = 8.4 Hz, 2H), 7.45 (t, *J* = 7.8 Hz, 2H), 7.35 (t, *J* = 7.4 Hz, 1H), 6.81 – 6.74 (m, 0.09H), 5.83 – 5.77 (m, 0.8H), 5.30 – 5.26 (m, 0.8H). **^{13}C NMR** (125 MHz, $CDCl_3$) δ 140.9, 140.7, 136.7, 128.9, 127.5, 127.4, 127.1, 126.8, 113.9.

5-([1,1'-biphenyl]-4-yl)-3-methyl-*N*-(2-methylquinolin-8-yl)pentanamide (*d*-9aj):

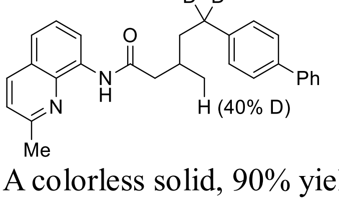

A colorless solid, 90% yield. **^{1}H NMR** (500 MHz, $CDCl_3$) δ 9.88 (s, 1H), 8.78 (dd, *J* = 6.9, 2.2 Hz, 1H), 8.02 (d, *J* = 8.4 Hz, 1H), 7.57 (dd, *J* = 8.3, 1.3 Hz, 2H), 7.52 – 7.41 (m, 6H), 7.35 – 7.27 (m, 4H), 2.73 (s, 3H), 2.65 (dd, *J* = 14.3, 6.1 Hz, 1H), 2.44 (dd, *J* = 14.3, 8.0 Hz, 1H), 2.38 – 2.16 (m, 1H), 1.86 (dd, *J* = 13.4, 5.6 Hz, 1H), 1.66 (dd, *J* = 13.5, 7.6 Hz, 1H), 1.16 (t, *J* = 8.1 Hz, 2.6H). **^{13}C NMR** (125 MHz, $CDCl_3$) δ 171.1, 157.3, 141.7, 141.2, 138.8, 137.8, 136.5, 134.0, 128.9, 128.8, 127.2, 127.1, 126.5, 126.17, 122.5, 121.3, 116.5, 46.0, 38.7, 30.8, 30.7, 25.4, 20.0. **^{2}D NMR** (77 MHz, $CHCl_3$) δ 2.79 – 2.72 (m, 2D), 1.19 (s, 0.4D) ppm. **HRMS** (**ESI**): Calcd. 412.2463 for $C_{28}H_{26}D_3N_2O$ $(M+H)^+$, found 412.2454.

Radical clock experiment

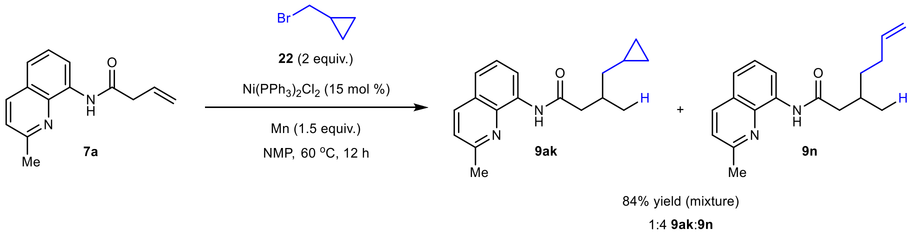

In a N_2-filled glovebox, to an oven-dried 5 mL vial equipped with a magnetic stir bar were added alkene substrate **7a** (23 mg, 0.1 mmol), $Ni(PPh_3)_2Cl_2$ (10 mg, 0.015 mmol) and Mn powder (0.15 mmol). The mixture was then dissolved in dry NMP (0.3 mL). The vial was tightly capped and removed out of the glovebox followed by the addition of (bromomethyl)cyclopropane **22** (27 mg, 0.2 mmol). The mixture was allowed to vigorously stir at 60 °C for 12 h. When alkene was almost fully consumed (monitored by GC-MS), the mixture was directly subjected to flash silica gel column chromatography to afford **9ak** and **9n** (mixture, 84% yield, 1:4 **9ak**: **9n**).

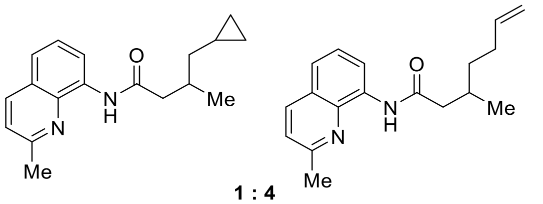

^{1}H NMR (300 MHz, $CDCl_3$) δ 9.86 (s, 1.25H), 8.75 (dd, J = 5.7, 3.3 Hz, 1.25H), 8.03 (d, J = 8.3 Hz, 1.25H), 7.46 (dd, J = 8.1, 5.6 Hz, 2.5H), 7.31 (d, J = 8.4 Hz, 1.25H), 5.84 (ddt, J = 16.9, 10.2, 6.6 Hz, 1H), 5.04 (dq, J = 17.1, 1.7 Hz, 1H), 4.96 (ddd, J = 10.1, 2.2, 1.1 Hz, 1H), 2.75 (s, 3.75H), 2.59 (dd, J = 14.1, 6.0 Hz, 1.25H), 2.36 (dd, J = 14.3, 8.0 Hz, 1.25H), 2.29 – 2.06 (m, 3.75H), 1.67 – 1.32 (m, 6H), 1.07 (d, J = 6.6 Hz, 3H). **HRMS** (**ESI**): Calcd. 305.1624 for $C_{18}H_{22}N_2NaO$ $(M+Na)^+$, found 305.1624.

Complete stereochemical erosion with an enantioenriched alkyl halide

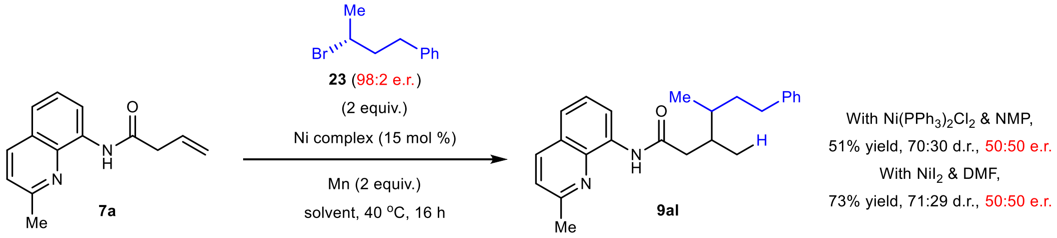

In a N_2-filled glovebox, to an oven-dried 5 mL vial equipped with a magnetic stir bar were added alkene substrate **7a** (23 mg, 0.1 mmol), $Ni(PPh_3)_2Cl_2$ (10 mg, 0.015 mmol) or NiI_2 (4.8 mg, 0.015 mmol) and Mn powder (0.15 mmol). The mixture was then dissolved in dry NMP (0.3 mL) or dry DMF (0.3 mL). The vial was tightly capped and removed out of the glovebox followed by the addition of **23** (44 mg, 0.2 mmol). The mixture was allowed to vigorously stir at 40 °C for 16 h. When alkene was almost fully consumed (monitored by GC-MS), the mixture was directly subjected to flash silica gel column chromatography to afford **9al** (51% yield with $NiCl_2(PPh_3)_2$ and 73% yield with NiI_2). The enantiomeric ratios were determined by chiral HPLC analysis.

(*R*)-(3-bromobutyl)benzene (23):

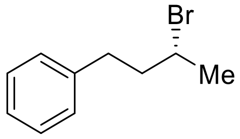

the title compound was synthesized according to a literature

procedure.[8] **^{1}H NMR** (500 MHz, $CDCl_3$) δ 7.31 – 7.28 (m, 2H), 7.22 – 7.19 (m, 3H), 4.08 (dqd, *J* = 8.9, 6.7, 4.5 Hz, 1H), 2.87 (ddd, *J* = 14.1, 9.0, 5.3 Hz, 1H), 2.75 (ddd, *J* = 13.8, 8.8, 7.3 Hz, 1H), 2.14 (dtd, *J* = 14.2, 8.9, 5.3 Hz, 1H), 2.08 – 2.01 (m, 1H), 1.73 (d, *J* = 6.7 Hz, 3H). **^{13}C NMR** (126 MHz, $CDCl_3$) δ 140.9, 128.5, 128.5, 126.1, 50.9, 42.7, 33.9, 26.5 ppm. Enantiomeric ratio was determined by HPLC analysis (OJ-H column, hexane/*i*PrOH 96/4, 0.50 mL/min, 190 nm): t_1 = 9.8 min (major), t_2 = 10.3 min (minor). 98:2 e.r.

<u>HPLC spectra for **23**</u>

Racemic compound:

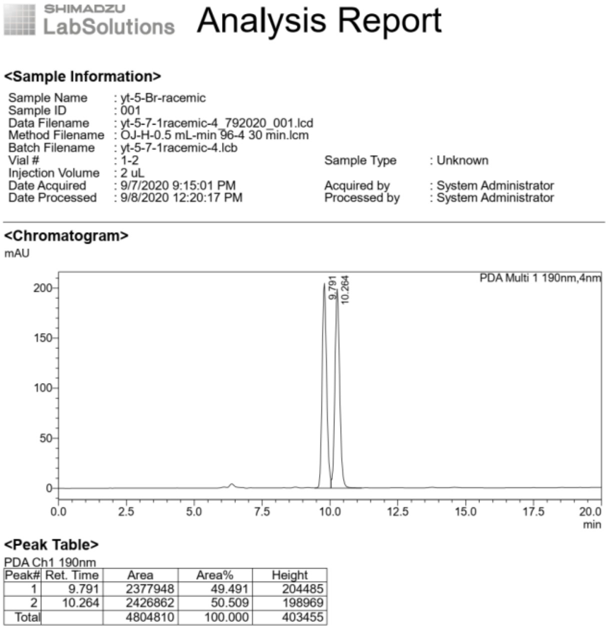
SHIMADZU LabSolutions Analysis Report

<Sample Information>

Sample Name : yt-5-Br-racemic
Sample ID : 001
Data Filename : yt-5-7-1racemic-4_792020_001.lcd
Method Filename : OJ-H-0.5 mL-min 96-4 30 min.lcm
Batch Filename : yt-5-7-1racemic-4.lcb
Vial # : 1-2 Sample Type : Unknown
Injection Volume : 2 uL
Date Acquired : 9/7/2020 9:15:01 PM Acquired by : System Administrator
Date Processed : 9/8/2020 12:20:17 PM Processed by : System Administrator

<Chromatogram>

<Peak Table>

PDA Ch1 190nm

Peak#	Ret. Time	Area	Area%	Height
1	9.791	2377948	49.491	204485
2	10.264	2426862	50.509	198969
Total		4804810	100.000	403455

Enantioenriched compound:

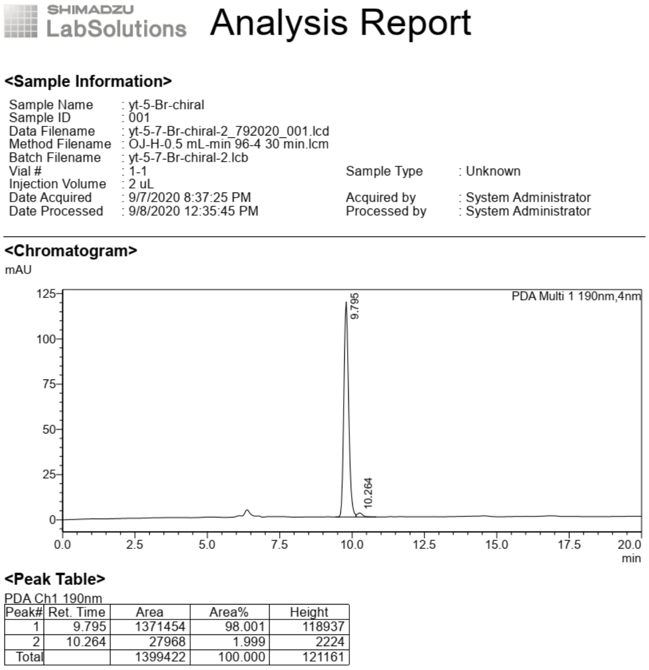
SHIMADZU LabSolutions Analysis Report

<Sample Information>

Sample Name : yt-5-Br-chiral
Sample ID : 001
Data Filename : yt-5-7-Br-chiral-2_792020_001.lcd
Method Filename : OJ-H-0.5 mL-min 96-4 30 min.lcm
Batch Filename : yt-5-7-Br-chiral-2.lcb
Vial # : 1-1 Sample Type : Unknown
Injection Volume : 2 uL
Date Acquired : 9/7/2020 8:37:25 PM Acquired by : System Administrator
Date Processed : 9/8/2020 12:35:45 PM Processed by : System Administrator

<Chromatogram>

<Peak Table>

PDA Ch1 190nm

Peak#	Ret. Time	Area	Area%	Height
1	9.795	1371454	98.001	118937
2	10.264	27968	1.999	2224
Total		1399422	100.000	121161

3,4-Dimethyl-*N*-(2-methylquinolin-8-yl)-6-phenylhexanamide (9al):

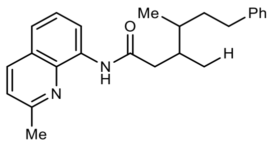

^{1}H NMR (500 MHz, $CDCl_3$) δ 9.87 (s, 1H), 8.76 (dd, *J* = 6.6, 2.2 Hz, 1H), 8.03 (d, *J* = 8.4 Hz, 1H), 7.48 – 7.44 (m, 2H), 7.32 (d, *J* = 8.4 Hz, 1H), 7.27 – 7.24 (m, 2H), 7.21 – 7.19 (m, 2H), 7.17 – 7.14 (m, 1H), 2.77 – 2.75 (m, 3H), 2.74 – 2.68 (m, 1H), 2.64 – 2.56 (m, 2H), 2.40 – 2.27 (m, 2H), 1.81 – 1.74 (m, 1H), 1.73 – 1.64 (m, 1H), 1.56 – 1.48 (m, 1H), 1.04 – 0.97 (m, 6H). **^{13}C NMR** (126 MHz, $CDCl_3$) δ 171.6, 171.4, 157.1, 142.8, 142.8, 137.7, 136.4, 133.9, 128.3, 128.3, 126.3, 126.0, 125.6, 125.6, 122.3, 121.1, 116.4, 116.3, 43.7, 42.1, 37.0, 36.8, 36.4, 35.2, 35.2, 34.5, 33.9, 29.7, 25.3, 16.7, 16.4, 14.7, 14.6 ppm. **HRMS** (**ESI**): Calcd. 383.2094 for $C_{24}H_{28}N_2NaO^+$ $(M+Na)^+$, found 383.2099.
Enantiomeric excess was determined by HPLC (IB column, hexane/*i*PrOH 97/3, 0.30 mL/min, 220 nm): t_1 = 42.6 min, t_2 = 44.5 min, t_3= 64.0 min, t_4= 72.1 min.

<u>HPLC spectra for **9al**</u>

Reaction with racemic **23**:

SHIMADZU LabSolutions **Analysis Report**

<Sample Information>

Sample Name	: yt-5-78-7		
Sample ID	: 001		
Data Filename	: yt-5-78-7-1racemic-11_892020_001.lcd		
Method Filename	: IB-0.3 mL-min 97-3 190-300nm 70 min.lcm		
Batch Filename	: yt-5-78-7-1racemic-11.lcb		
Vial #	: 1-10	Sample Type	: Unknown
Injection Volume	: 5 uL		
Date Acquired	: 9/8/2020 11:36:56 AM	Acquired by	: System Administrator
Date Processed	: 9/8/2020 1:16:59 PM	Processed by	: System Administrator

<Chromatogram>

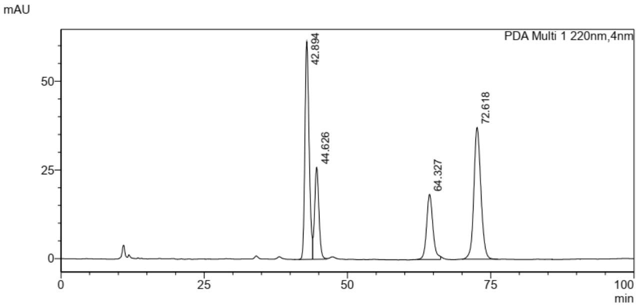

<Peak Table>

PDA Ch1 220nm

Peak#	Ret. Time	Area	Area%	Height
1	42.894	3141505	34.633	61354
2	44.626	1357684	14.968	25928
3	64.327	1410529	15.550	18359
4	72.618	3161039	34.849	37089
Total		9070756	100.000	142730

Reaction with enantioenriched **23** using NiI_2:

Analysis Report

<Sample Information>

Sample Name	: yt-5-78-7chiral		
Sample ID	: 001		
Data Filename	: yt-5-78-7-1chiral-1_892020_002.lcd		
Method Filename	: IB-0.3 mL-min 97-3 190-300nm 70 min.lcm		
Batch Filename	: yt-5-78-7-1chiral-1.lcb		
Vial #	: 1-12	Sample Type	: Unknown
Injection Volume	: 2 uL		
Date Acquired	: 9/8/2020 2:46:18 PM	Acquired by	: System Administrator
Date Processed	: 9/8/2020 4:26:20 PM	Processed by	: System Administrator

<Chromatogram>

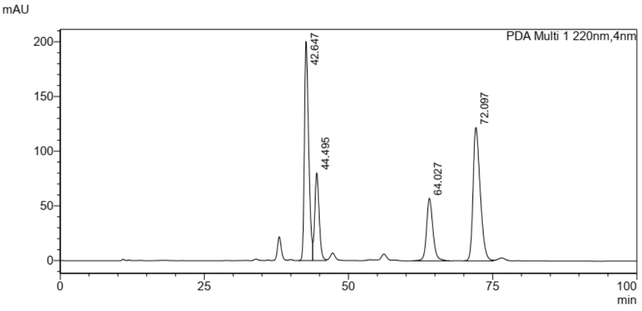

<Peak Table>

PDA Ch1 220nm

Peak#	Ret. Time	Area	Area%	Height
1	42.647	10364630	35.111	200120
2	44.495	4277307	14.490	80056
3	64.027	4381708	14.843	57087
4	72.097	10496072	35.556	121900
Total		29519718	100.000	459163

Reaction with enantioenriched **23** using $NiCl_2(PPh_3)_2$:

Analysis Report

<Sample Information>

Sample Name	: yt-5-78-7chiral		
Sample ID	: 001		
Data Filename	: yt-5-78-7-1chiral-2_892020_001.lcd		
Method Filename	: IB-0.3 mL-min 97-3 190-300nm 70 min.lcm		
Batch Filename	: yt-5-78-7-1chiral-2.lcb		
Vial #	: 1-13	Sample Type	: Unknown
Injection Volume	: 2 uL		
Date Acquired	: 9/8/2020 4:39:51 PM	Acquired by	: System Administrator
Date Processed	: 9/8/2020 6:51:53 PM	Processed by	: System Administrator

<Chromatogram>

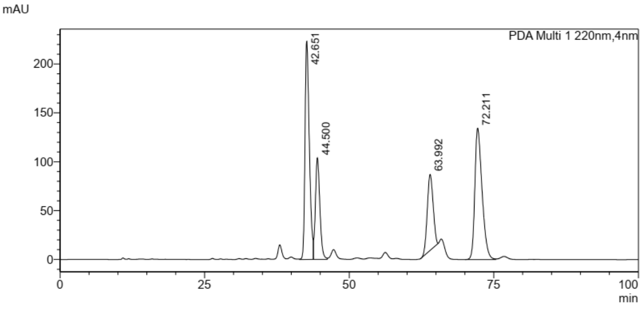

<Peak Table>

PDA Ch1 220nm

Peak#	Ret. Time	Area	Area%	Height
1	42.651	11620954	33.713	223203
2	44.500	5618995	16.301	103873
3	63.992	5508350	15.980	77755
4	72.211	11722085	34.006	134430
Total		34470384	100.000	539261

2.6 Attempted Hydroalkylation in the Presence of Hydrosilane/Base

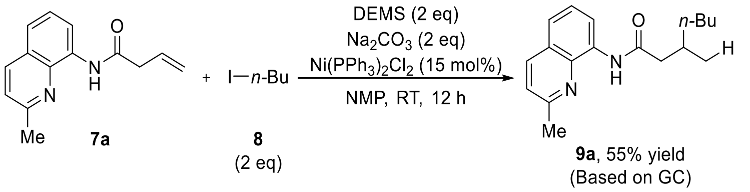

7a **8** (2 eq) **9a**, 55% yield (Based on GC)

According to a literature procedure[9], in a N_2-filled glovebox, to an oven-dried 5 mL

vial equipped with a magnetic stir bar were added alkene substrate **7a** (23 mg, 0.1 mmol), $Ni(PPh_3)_2Cl_2$ (10 mg, 0.015 mmol), Na_2CO_3 (21 mg, 0.2 mmol), and dry NMP (0.3 mL) sequentially. The vial was tightly capped and removed from the glovebox followed by addition of DEMS (27 mg, 0.2 mmol) and 1-iodobutane **8** (37 mg, 0.2 mmol) by micro-syringe. The mixture was allowed to vigorously stir at ambient temperature for 12 h. After that, 20 mg *n*-tridecane was added as an internal standard. 3 μL of the reaction mixture was taken out and subjected to GC analysis.

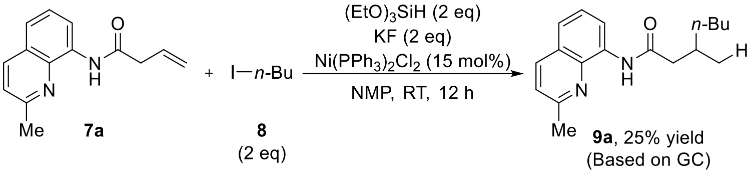

According to a literature procedure[10], in a N_2-filled glovebox, to an oven-dried 5 mL vial equipped with a magnetic stir bar were added alkene substrate **7a** (23 mg, 0.1 mmol), $Ni(PPh_3)_2Cl_2$ (10 mg, 0.015 mmol), KF (11 mg, 0.2 mmol), and dry NMP (0.3 mL) sequentially. The vial was tightly capped and removed from the glovebox followed by addition of $(EtO)_3SiH$ (33 mg, 0.2 mmol) and 1-iodobutane **8** (37 mg, 0.2 mmol) by micro-syringe. The mixture was allowed to vigorously stir at ambient temperature for 12 h. After that, 20 mg *n*-tridecane was added as an internal standard. 3 μL of the reaction mixture was taken out and subjected to GC analysis.

2.7 Attempted Hydroalkylation Using Substrates Without C_β-H Bonds

General procedure for condition optimization: In a N_2-filled glovebox, to an oven-dried 5 mL vial equipped with a magnetic stir bar were added alkene substrate **7a** (22.6 mg, 0.1 mmol), NiX_2(0.015 mmol), Mn powder (11 mg, 0.2 mmol) and dry solvent (0.3 mL) sequentially. The vial was tightly capped and removed out of the glovebox followed by the addition of neopentyl bromide and isopropyl bromide via a micro-syringe. The mixture was allowed to vigorously stir at 30 °C for 16 h. After that, 20 mg *n*-tridecane was added as an internal standard. 3 μL of the reaction mixture was taken out and subjected to GC analysis to determine the conversion of alkene **7a** as well as the calibrated GC yields of products **9x**, **9w** and **9w'**.

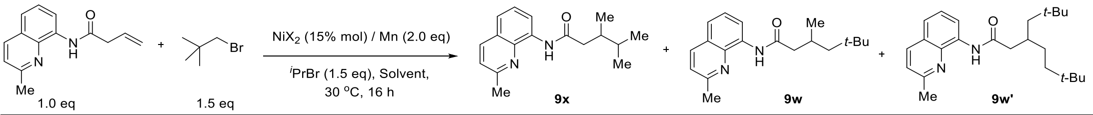

entry	NiX_2	additive[c]	Solvent	Con. Of alkene	Yield (**9x**)	Yield (**9w**)	Yield (**9w'**)
1	$NiCl_2(PPh_3)_2$	--	NMP	>95%	47%	26%	trace
2	NiI_2	--	NMP	>95%	46%	31%	6%
3	$NiCl_2$·dme	--	NMP	>95%	43%	30%	9%
4	$NiCl_2(PPh_3)_2$	--	DMF	>95%	48%	27%	3%
5	NiI_2	--	DMF	>95%	42%	26%	10%
6	$NiCl_2$·dme	--	DMF	>95%	39%	27%	13%
7	$NiCl_2(PPh_3)_2$	--	DMA	>95%	51%	24%	trace
8	NiI_2	--	DMA	>95%	47%	18%	4%
9	$NiCl_2$·dme	--	DMA	>95%	42%	23%	12%
10	$NiCl_2(PPh_3)_2$	--	DMPU	38%	20%	8%	3%

11	NiI_2	--	DMPU	89%	43%	12%	trace
12	$NiCl_2$·dme	--	DMPU	88%	42%	21%	3%
13	$NiCl_2(PPh_3)_2$	--	NMP	>95%	46%	18%	trace
14	NiI_2	--	NMP	>95%	39%	21%	11%
15	$NiCl_2$·dme	--	NMP	91%	8%	6%	51%
16	$NiBr_2$·diglyme	--	NMP	>95%	27%	17%	30%
17	$NiCl_2(PPh_3)_2$	NaI	NMP	80%	52%	trace	trace
18	NiI_2	NaI	NMP	>95%	44%	23%	6%
19	$NiCl_2$·dme	NaI	NMP	82%	42%	20%	5%
20	NiI_2	LiCl	NMP	65%	27%	17%	3%
21	NiI_2	LiBr	NMP	74%	31%	19%	2%
22	NiI_2	$MgCl_2$	NMP	>95%	14%	11%	50%
23	NiI_2	$MgBr_2$	NMP	89%	30%	17%	19%
24	NiI_2	$ZnBr_2$	NMP	35%	21%	3%	trace
25[a]	$NiCl_2(PPh_3)_2$	--	NMP	>95%	35%	31%	3%
**26[a]**	**NiI_2**	**--**	**NMP**	**>95%**	**26%**	**32%**	**15%**
27[b]	NiI_2	--	NMP	85%	--	trace	51%

[a]3.0 equiv of neopentyl bromide and 1.2 equiv of isopropyl bromide were used, yields reported are for isolated yields. [b]1.5 equiv of neopentyl bromide was used without isopropyl bromide. [c]1.0 equiv of additive was used.

3,5,5-Trimethyl-*N*-(2-methylquinolin-8-yl)hexanamide (9w):

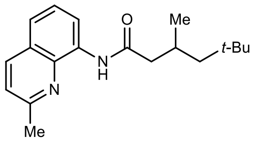

^{1}H NMR (400 MHz, $CDCl_3$) δ 9.86 (s, 1H), 8.74 (dd, *J* = 6.4, 2.6 Hz, 1H), 8.02 (d, *J* = 8.4 Hz, 1H), 7.48 – 7.42 (m, 2H), 7.31 (d, *J* = 8.4 Hz, 1H), 2.74 (s, 3H), 2.59 (dd, *J* = 13.9, 5.5 Hz, 1H), 2.35 (dd, *J* = 13.9, 8.4 Hz, 1H), 2.30 – 2.23 (m, 1H), 1.40 (dd, *J* = 14.0, 4.0 Hz, 1H), 1.22 (dd, *J* = 14.0, 6.2 Hz, 1H),1.10 (d, *J* = 6.5 Hz, 3H), 0.96 (s, 9H). **^{13}C NMR** (101 MHz, $CDCl_3$) δ 171.2, 157.1, 137.7, 136.4, 133.9, 126.4, 126.0, 122.3, 121.0, 116.3, 50.8, 48.1, 31.2, 30.0, 27.6, 25.2, 22.8 ppm. **HRMS** (**ESI**): Calcd. 321.1937 for $C_{19}H_{26}N_2NaO^+$ $(M+Na)^+$, found 321.1931.

6,6-Dimethyl-*N*-(2-methylquinolin-8-yl)-3-neopentylheptanamide (9w'):

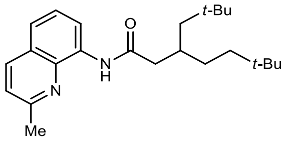

^{1}H NMR (500 MHz, $CDCl_3$) δ 9.87 (s, 1H), 8.74 (dd, *J* = 6.7, 1.7 Hz, 1H), 8.03 (d, *J* = 8.3 Hz, 1H), 7.47 – 7.43 (m, 2H), 7.31 (d, *J* = 8.5 Hz, 1H), 2.74 (s, 3H), 2.57 – 2.46 (m, 2H), 2.07 – 2.01 (m, 1H), 1.41 – 1.38 (m, 2H), 1.32 – 1.26 (m, 4H), 0.96 (s, 9H), 0.84 (s, 9H). **^{13}C NMR** (126 MHz, $CDCl_3$) δ 171.4, 157.0, 137.7, 136.4, 134.0, 128.5, 126.4, 122.3, 121.0, 116.3, 47.9, 45.4, 40.9, 32.7, 31.2, 31.0, 30.1, 29.9, 29.4, 25.2 ppm. **HRMS** (**ESI**): Calcd. 391.2720 for $C_{24}H_{36}N_2NaO^+$ $(M+Na)^+$, found 391.2726.

2.8. NMR Spectra

Supplementary Figure 1 ^{1}H NMR (500 MHz, $CDCl_3$) of **7a:**

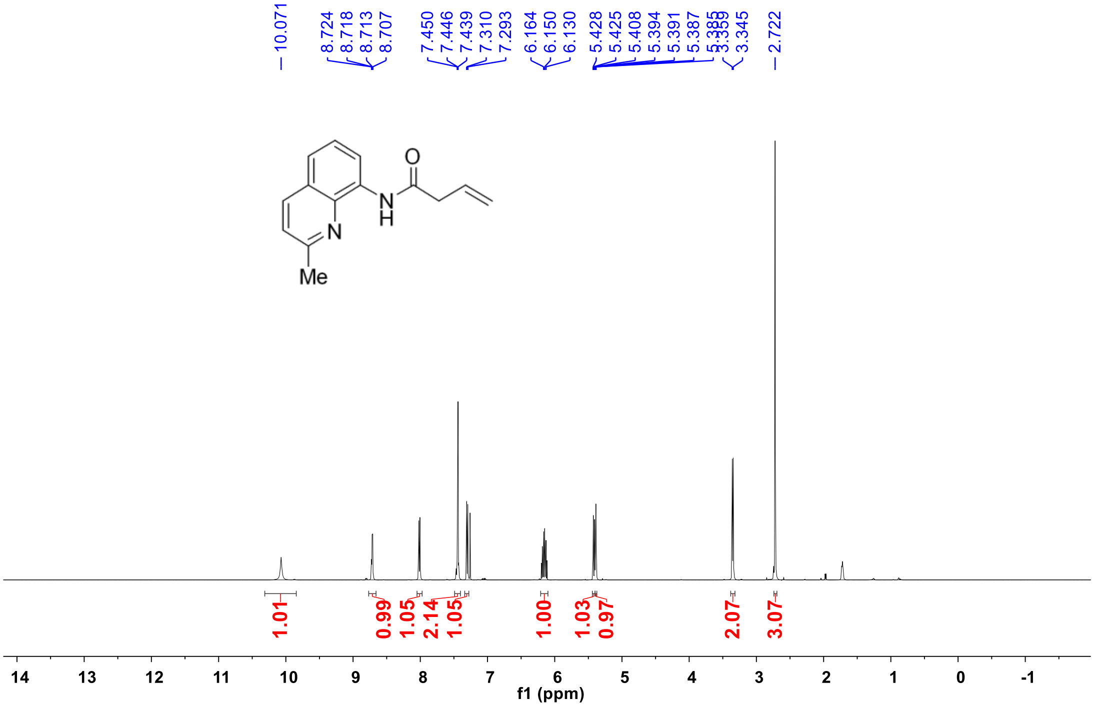

Supplementary Figure 2 ^{13}C NMR (100 MHz, $CDCl_3$) of **7a:**

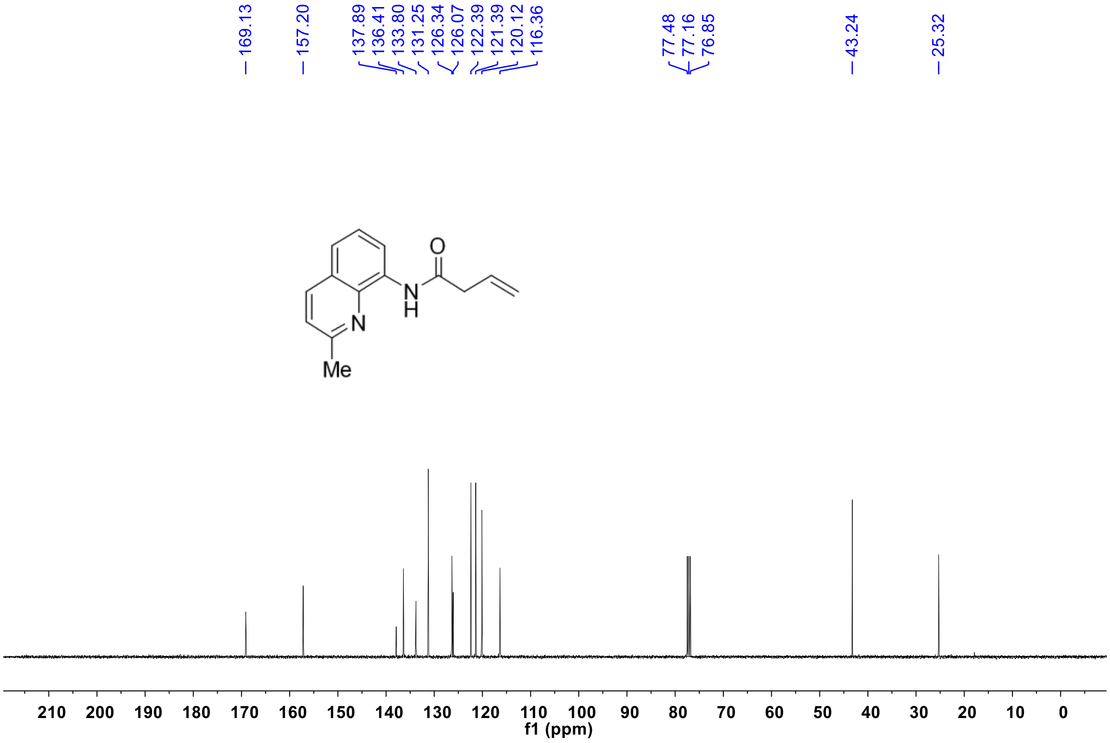

Supplementary Figure 3 ^{1}H NMR (500 MHz, $CDCl_3$) of **7b:**

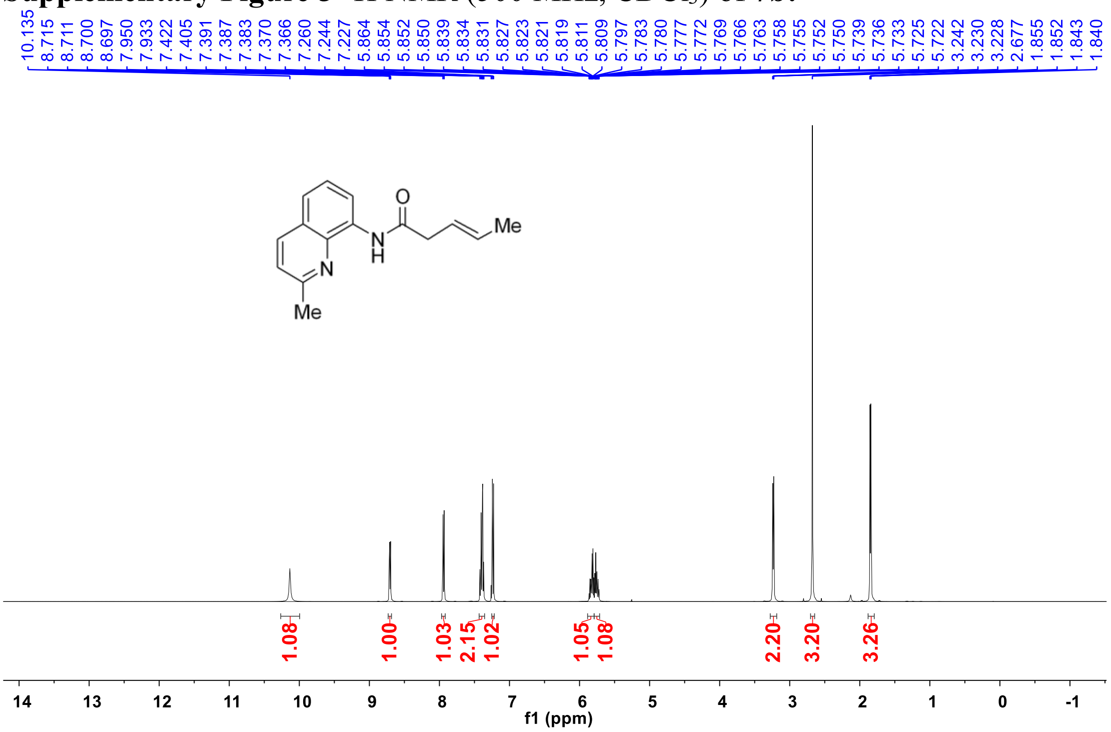

Supplementary Figure 4 ^{13}C NMR (125 MHz, $CDCl_3$) of **7b:**

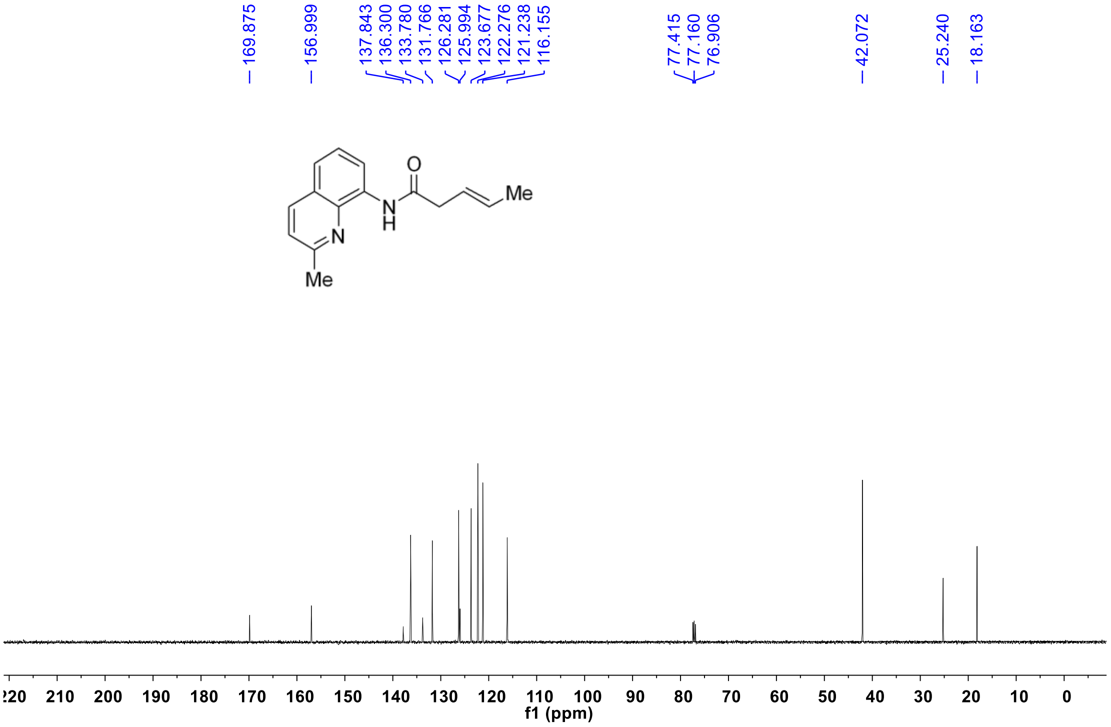

Supplementary Figure 5 ^{1}H NMR (400 MHz, $CDCl_3$) of **7c:**

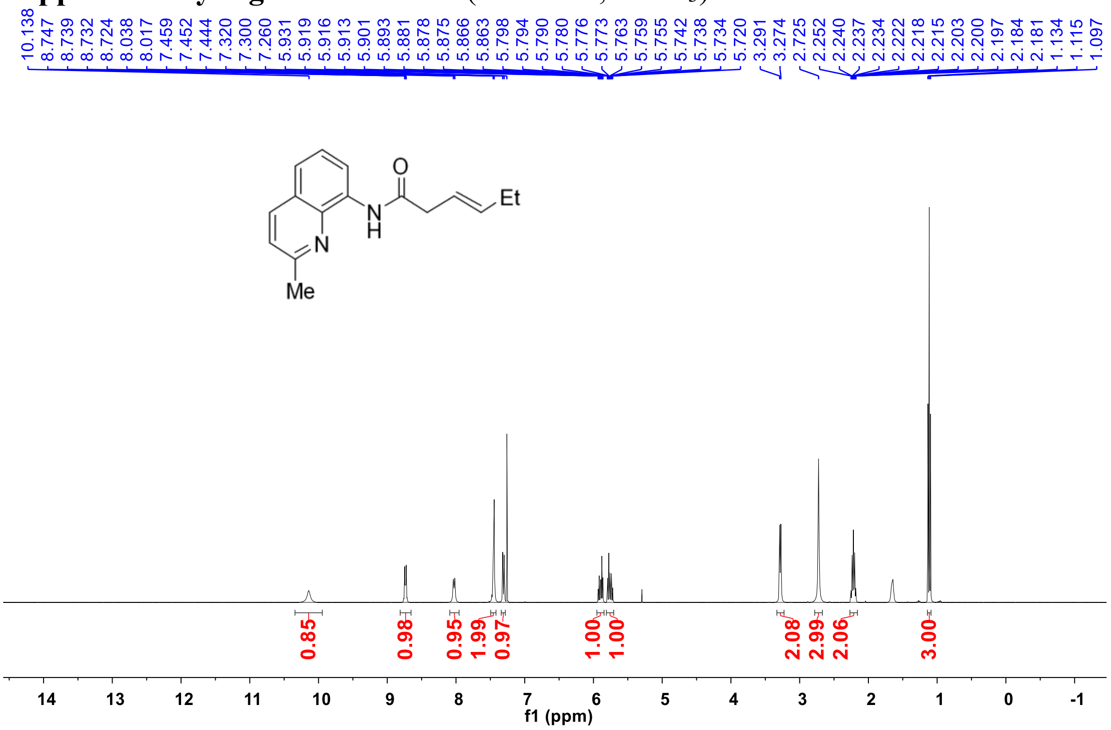

Supplementary Figure 6 ^{13}C NMR (100 MHz, $CDCl_3$) of **7c:**

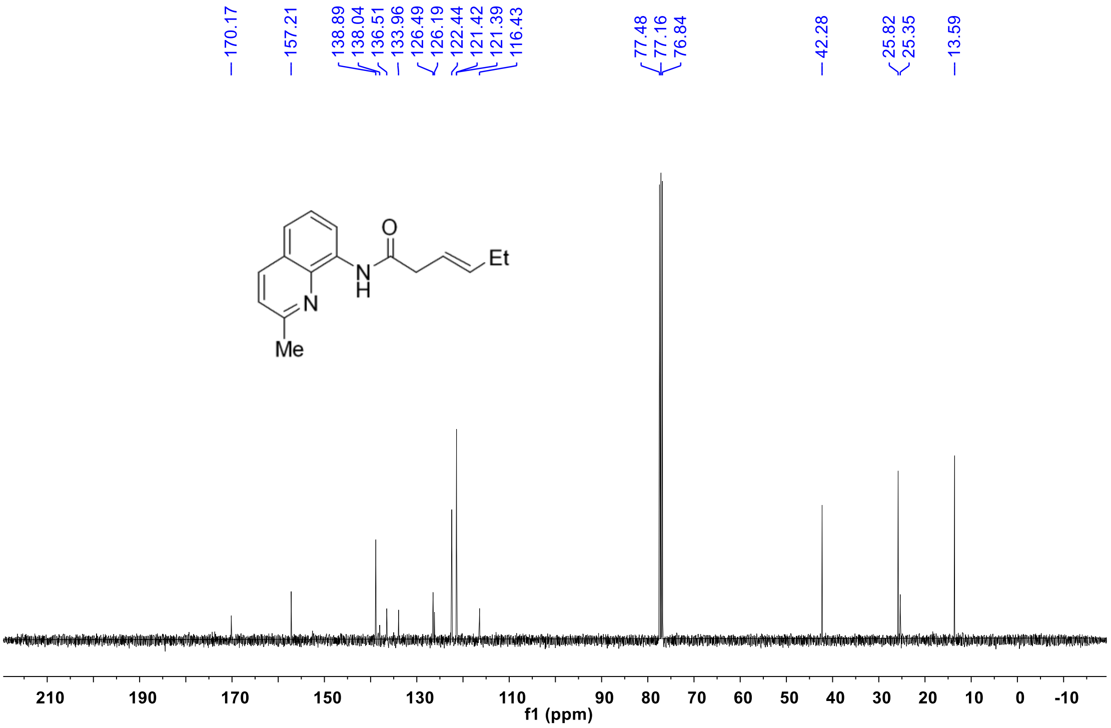

Supplementary Figure 7 ^{1}H NMR (400 MHz, $CDCl_3$) of **7d:**

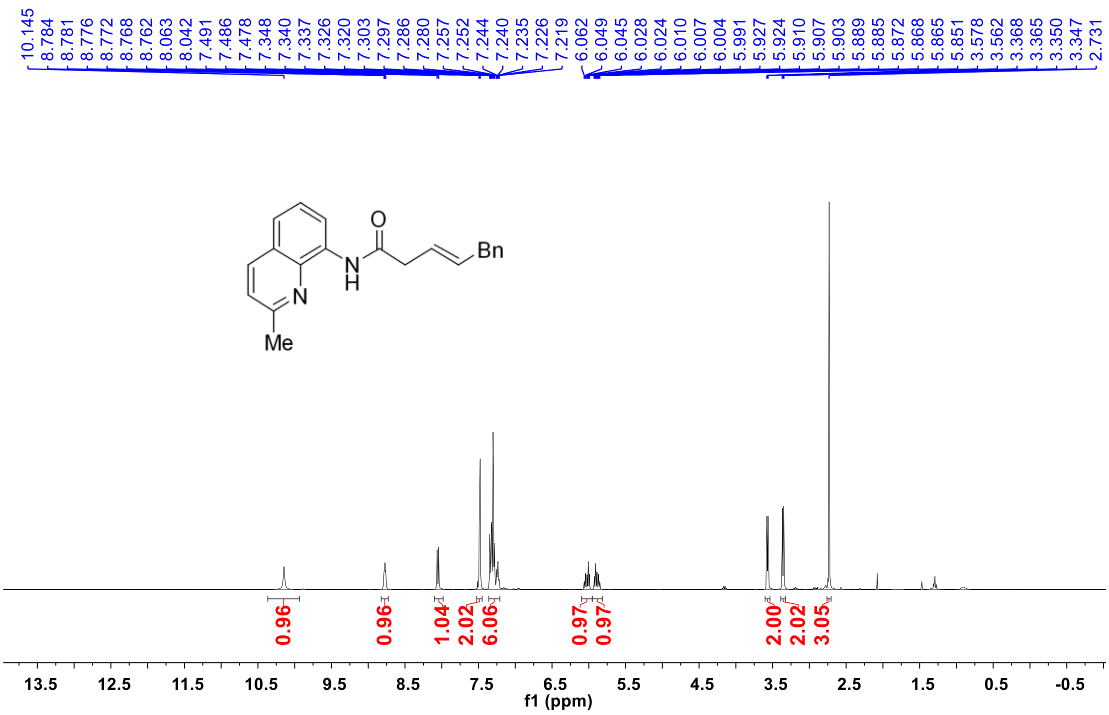

Supplementary Figure 8 ^{13}C NMR (100 MHz, $CDCl_3$) of **7d:**

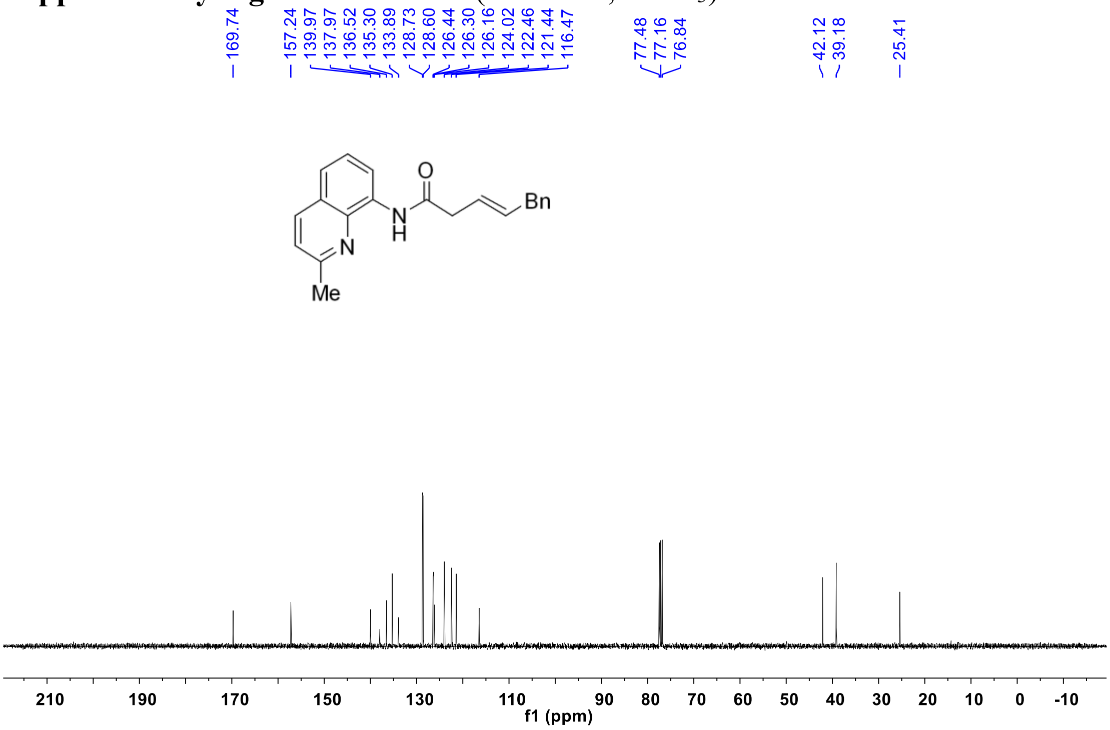

Supplementary Figure 9 ^{1}H NMR (500 MHz, $CDCl_3$) of **7e:**

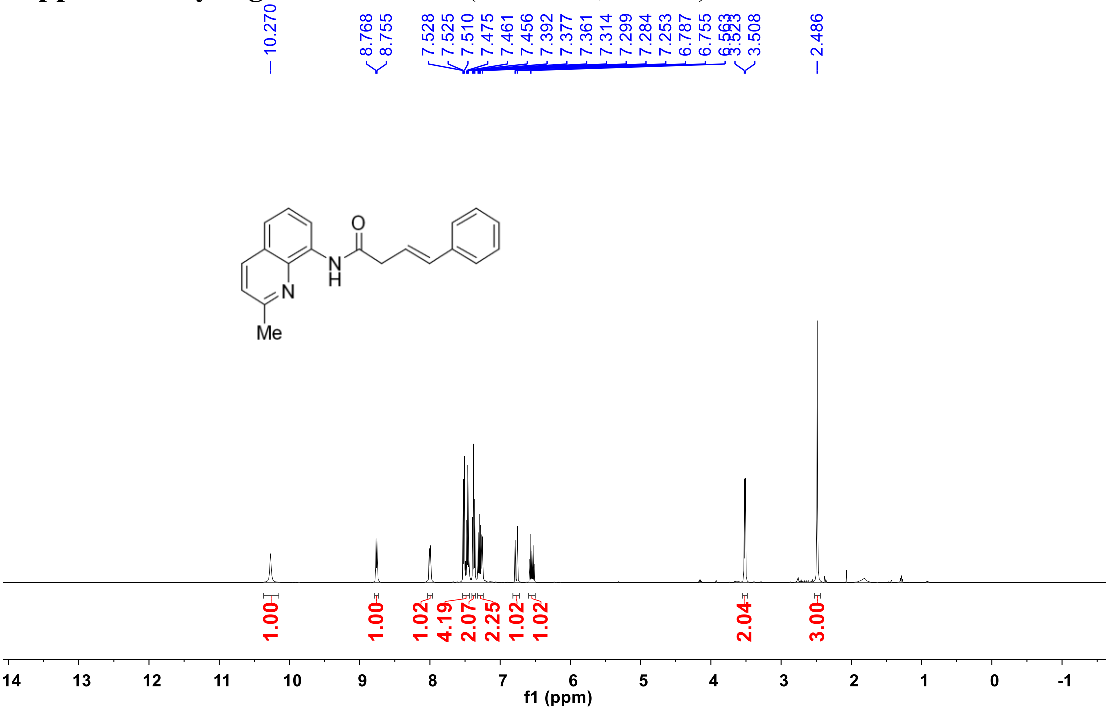

Supplementary Figure 10 ^{13}C NMR (125 MHz, $CDCl_3$) of **7e:**

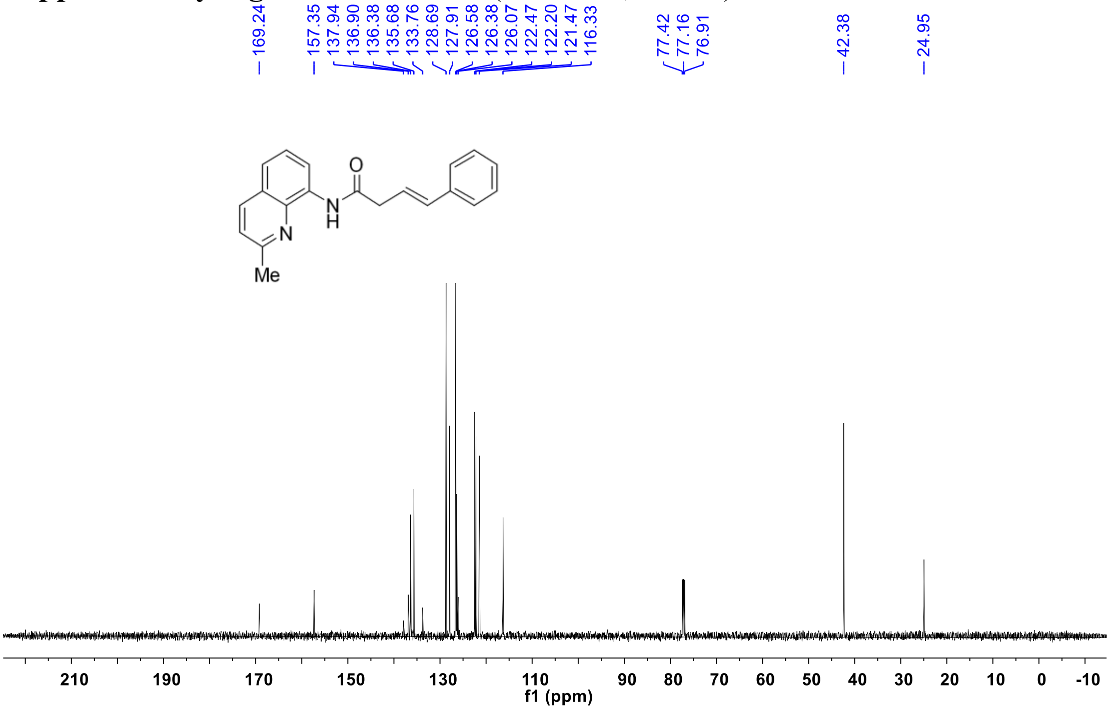

Supplementary Figure 11 ^{1}H NMR (400 MHz, $CDCl_3$) of 1**7a:**

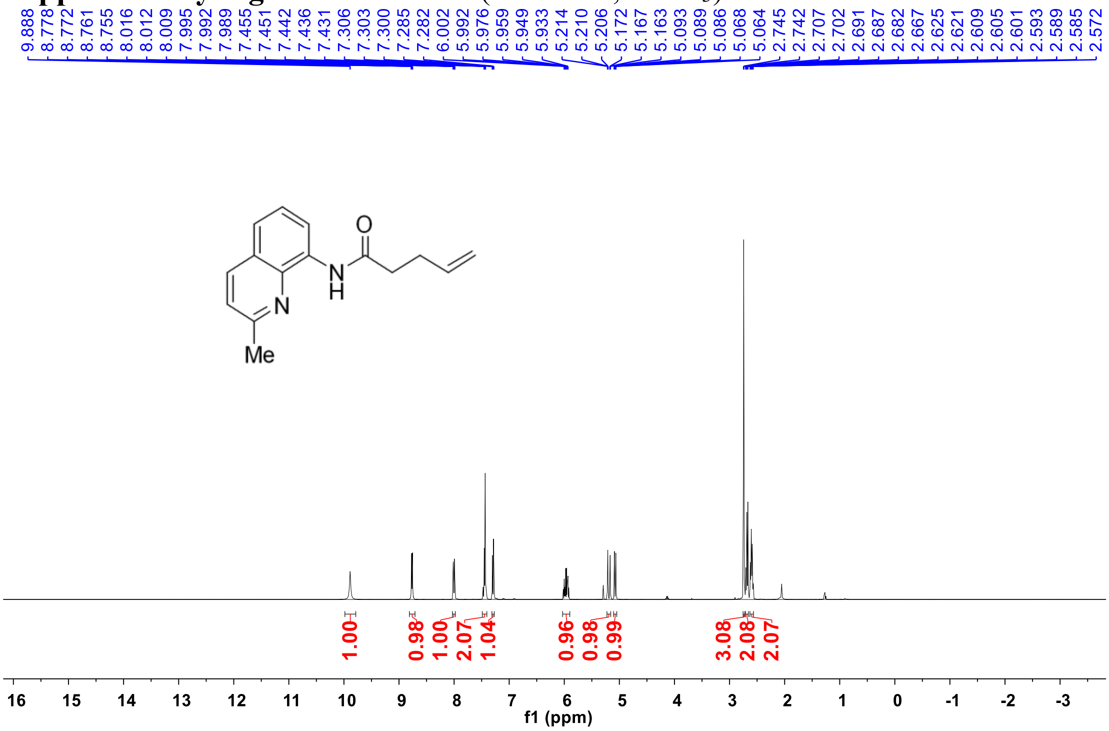

Supplementary Figure 12 ^{13}C NMR (100 MHz, $CDCl_3$) of 1**7a:**

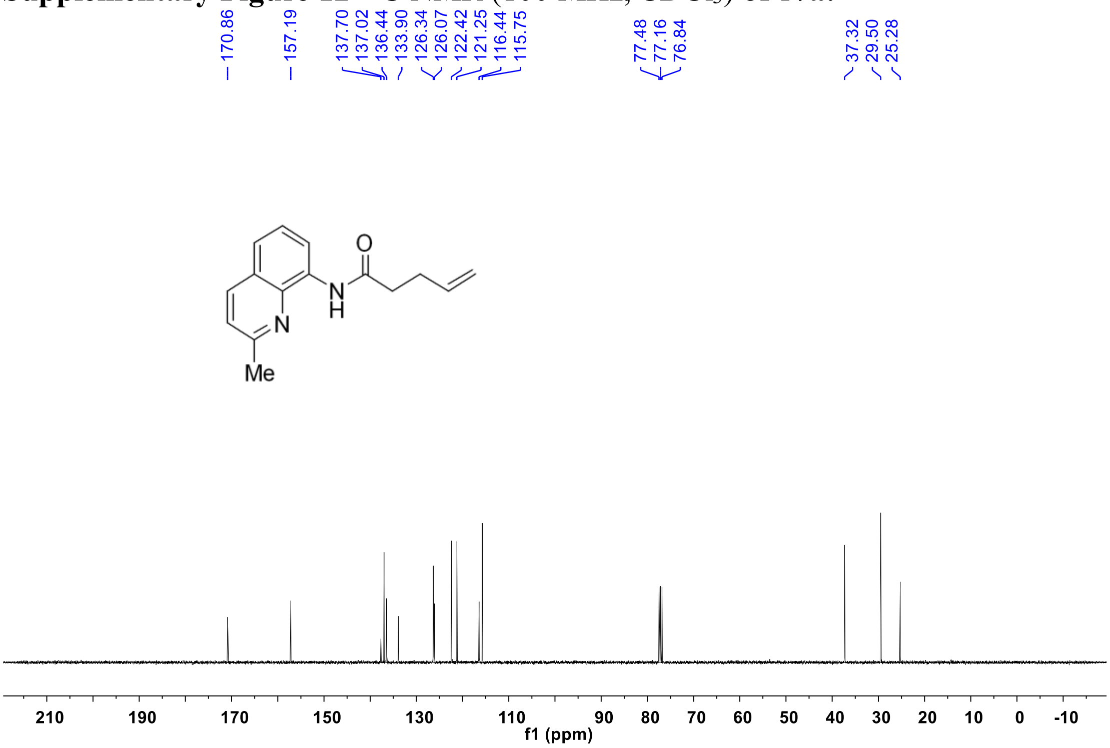

Supplementary Figure 13 ^{1}H NMR (500 MHz, $CDCl_3$) of **17b:**

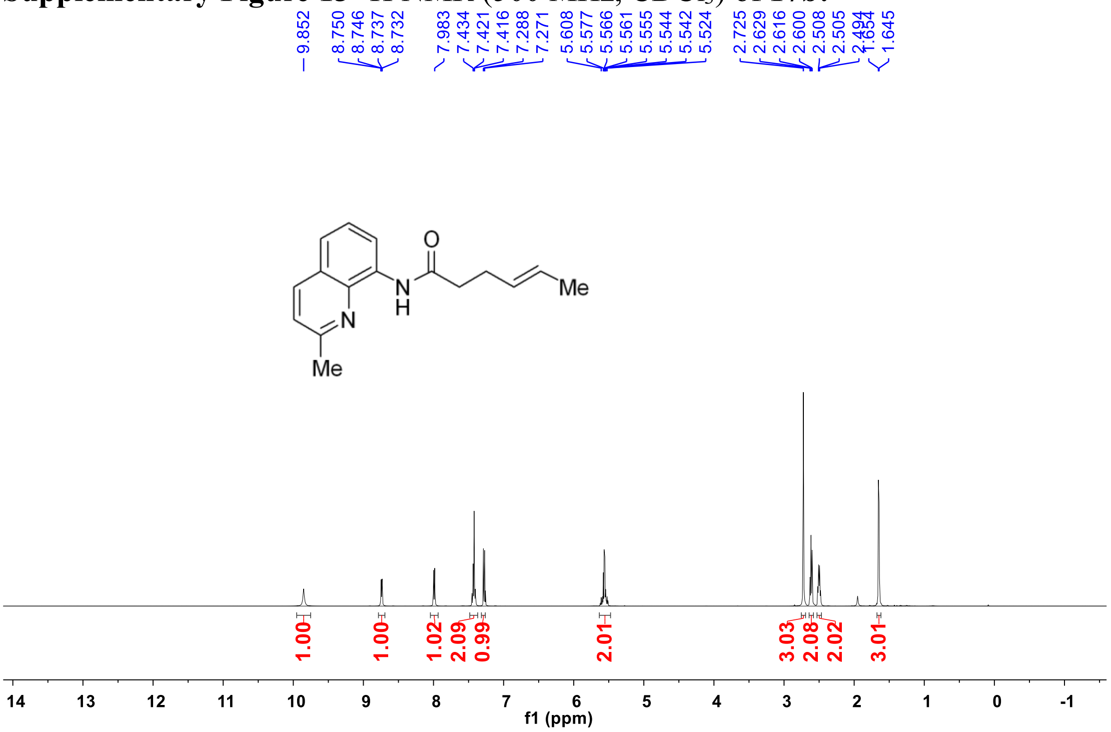

Supplementary Figure 14 ^{13}C NMR (125 MHz, $CDCl_3$) of **17b:**

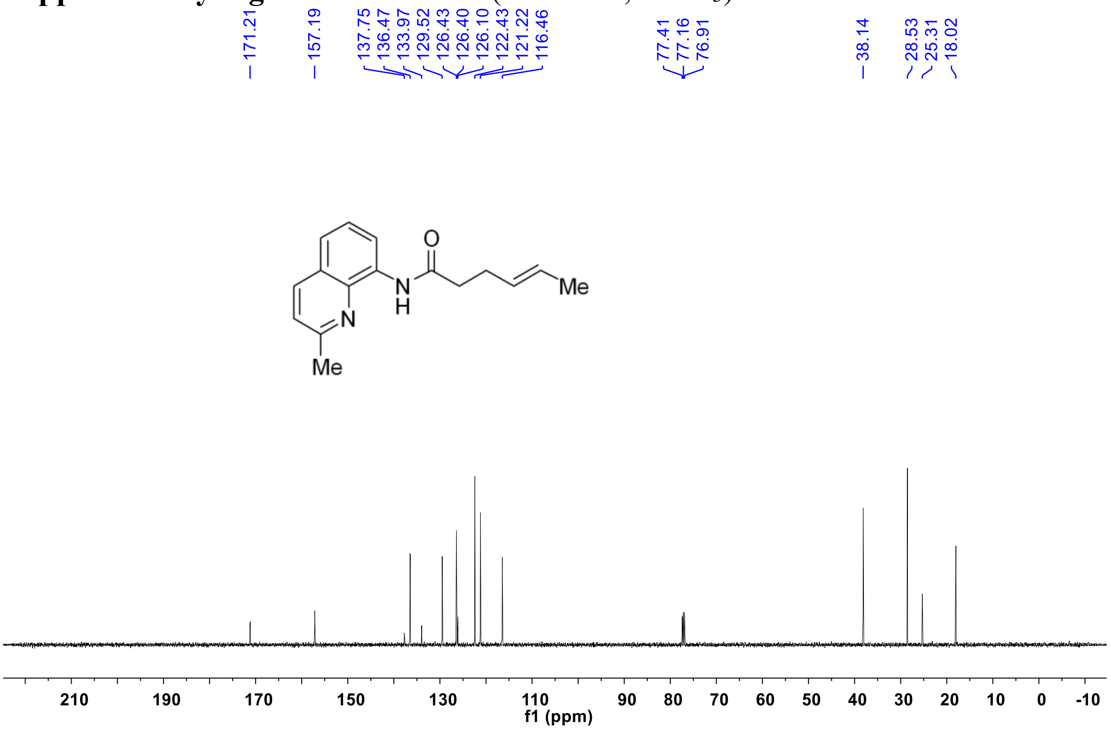

Supplementary Figure 15 ^{1}H NMR (400 MHz, $CDCl_3$) of **17c:**

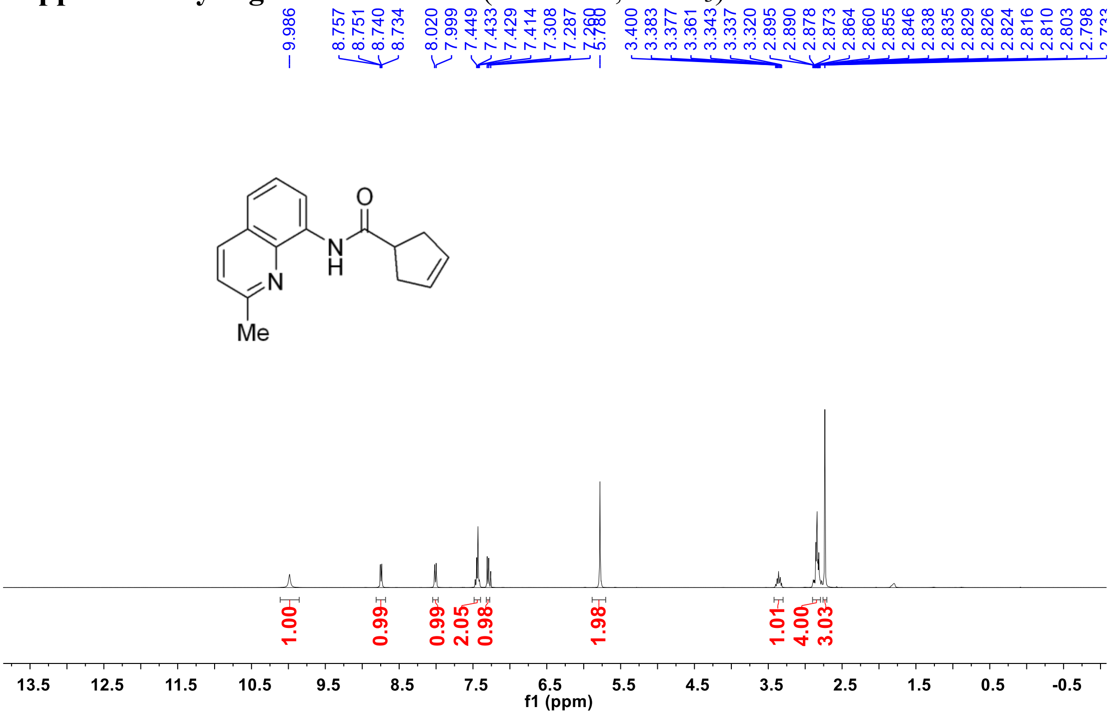

Supplementary Figure 16 ^{13}C NMR (100 MHz, $CDCl_3$) of **17c:**

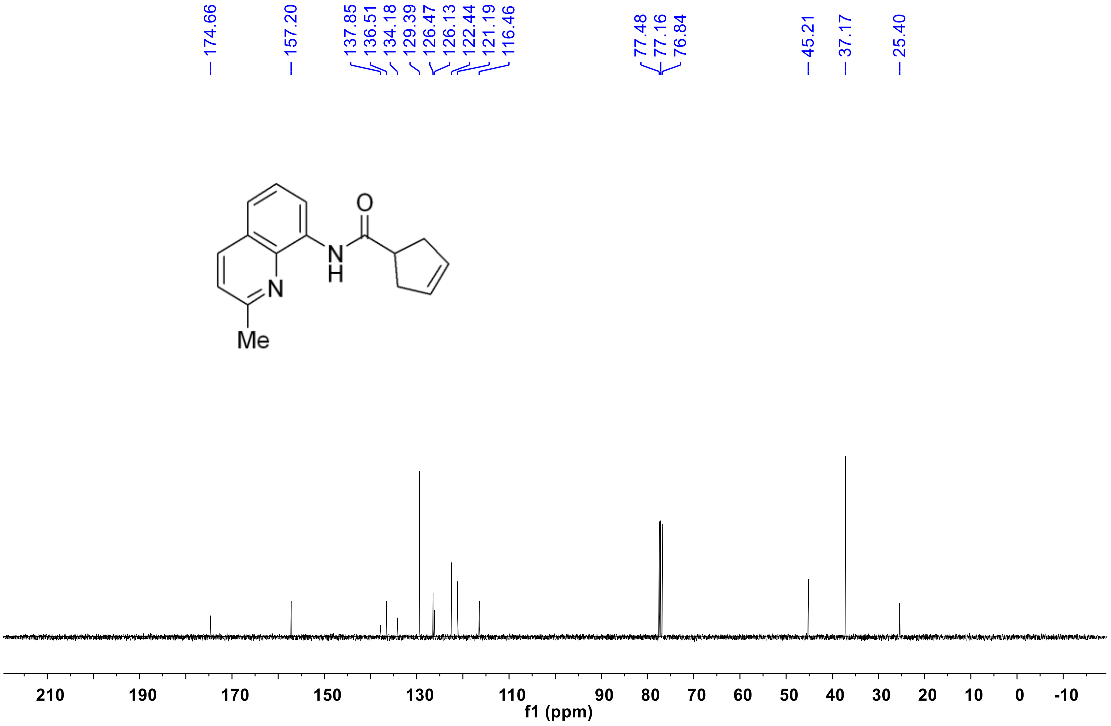

Supplementary Figure 17 ^{1}H NMR (500 MHz, $CDCl_3$) of **17d:**

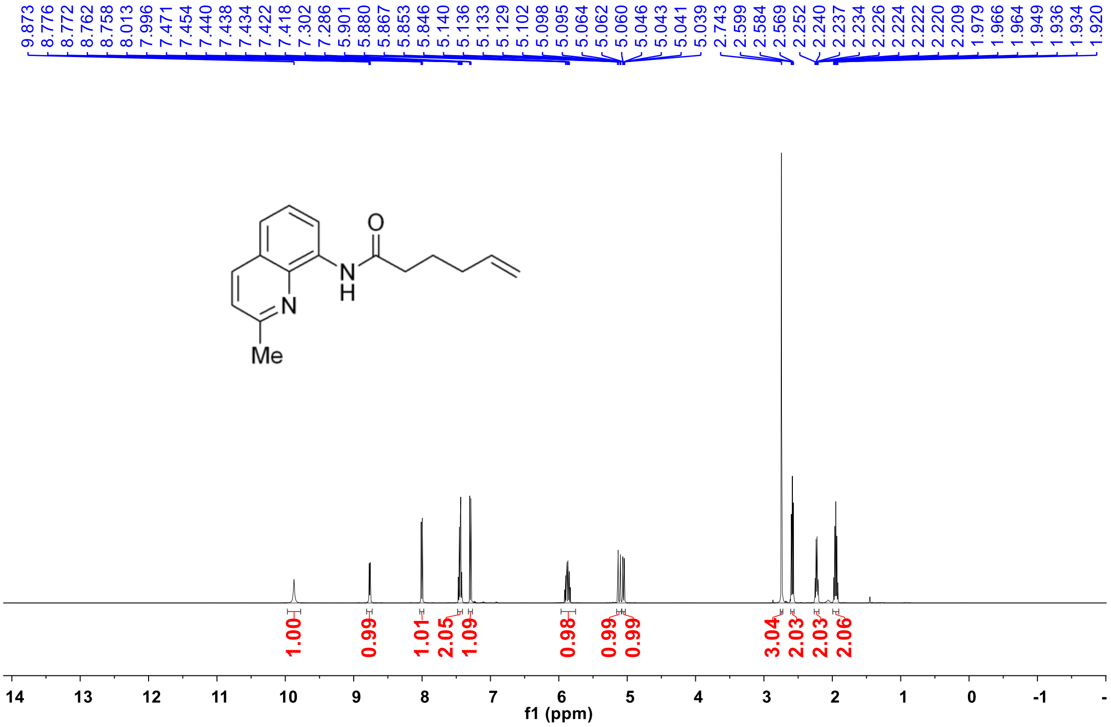

Supplementary Figure 18 ^{13}C NMR (125 MHz, $CDCl_3$) of **17d:**

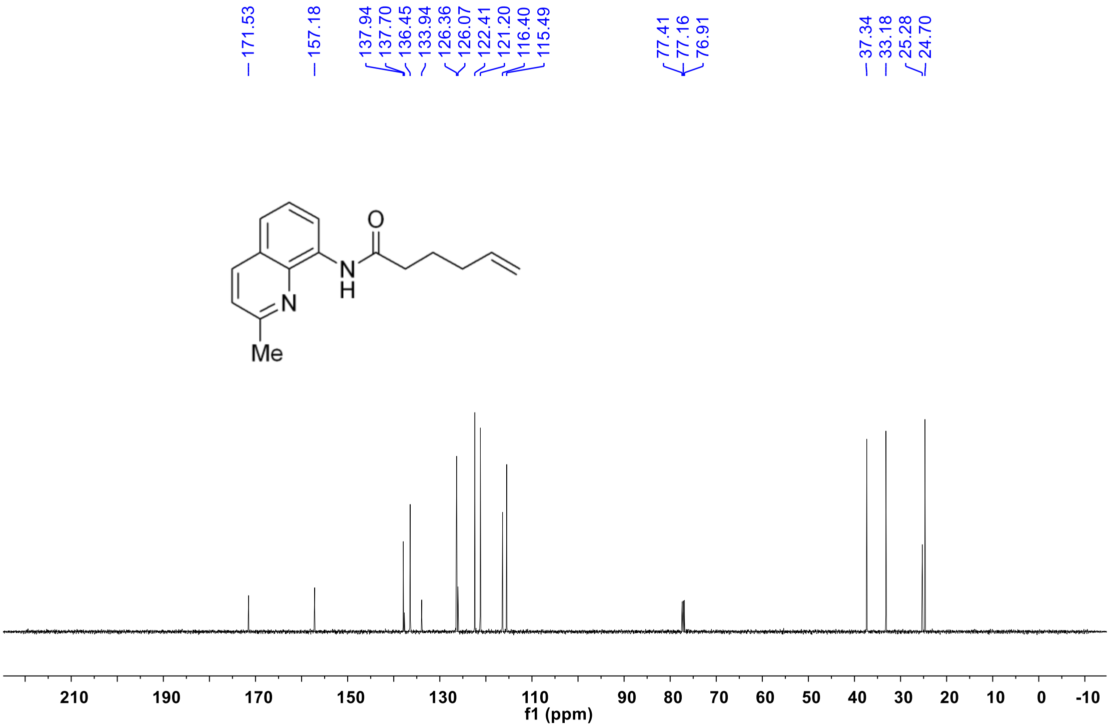

Supplementary Figure 19 ^{1}H NMR (400 MHz, $CDCl_3$) of **17e:**

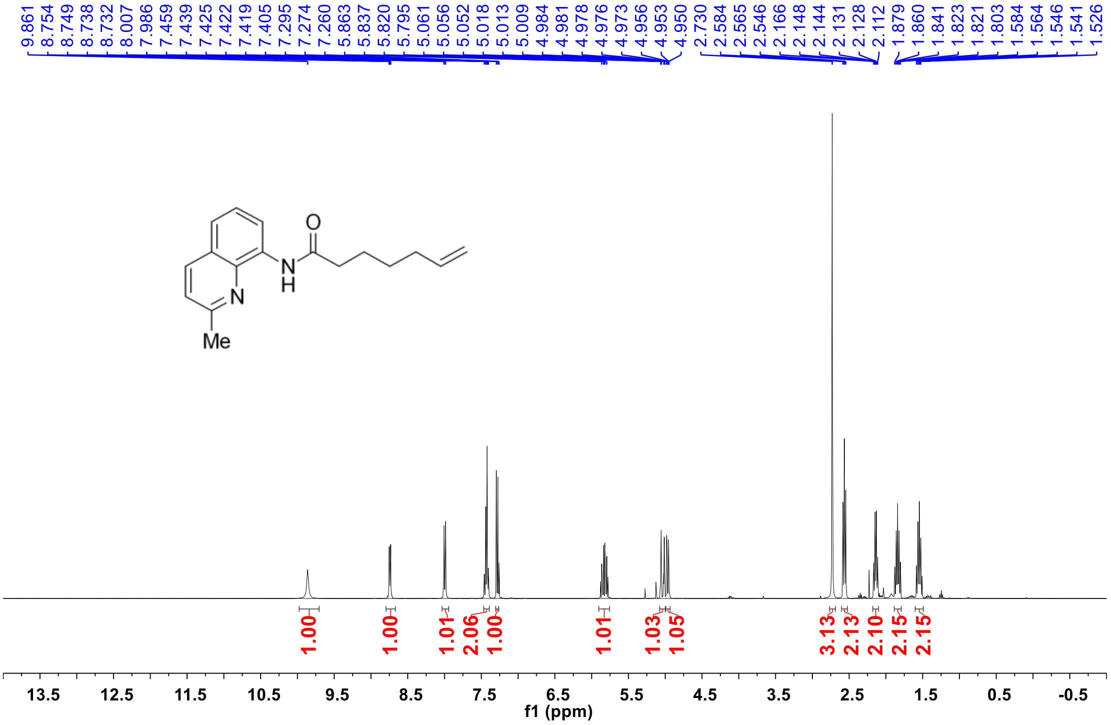

Supplementary Figure 20 ^{13}C NMR (100 MHz, $CDCl_3$) of **17e:**

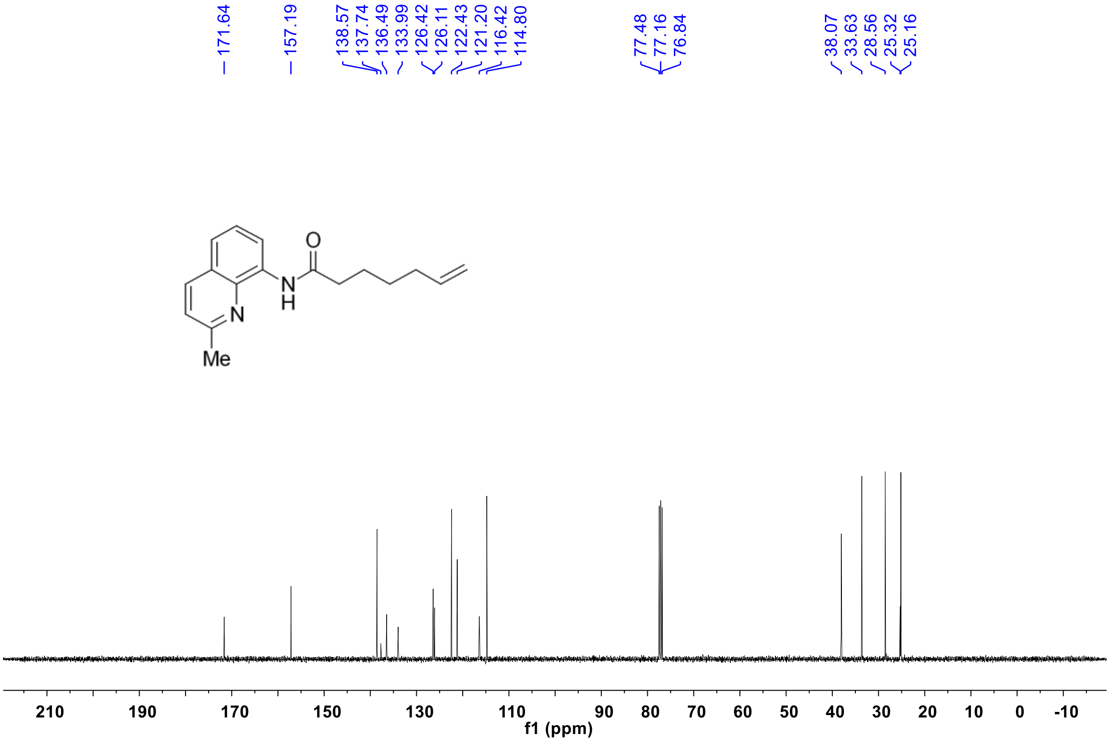

Supplementary Figure 21 ^{1}H NMR (400 MHz, $CDCl_3$) of **7g:**

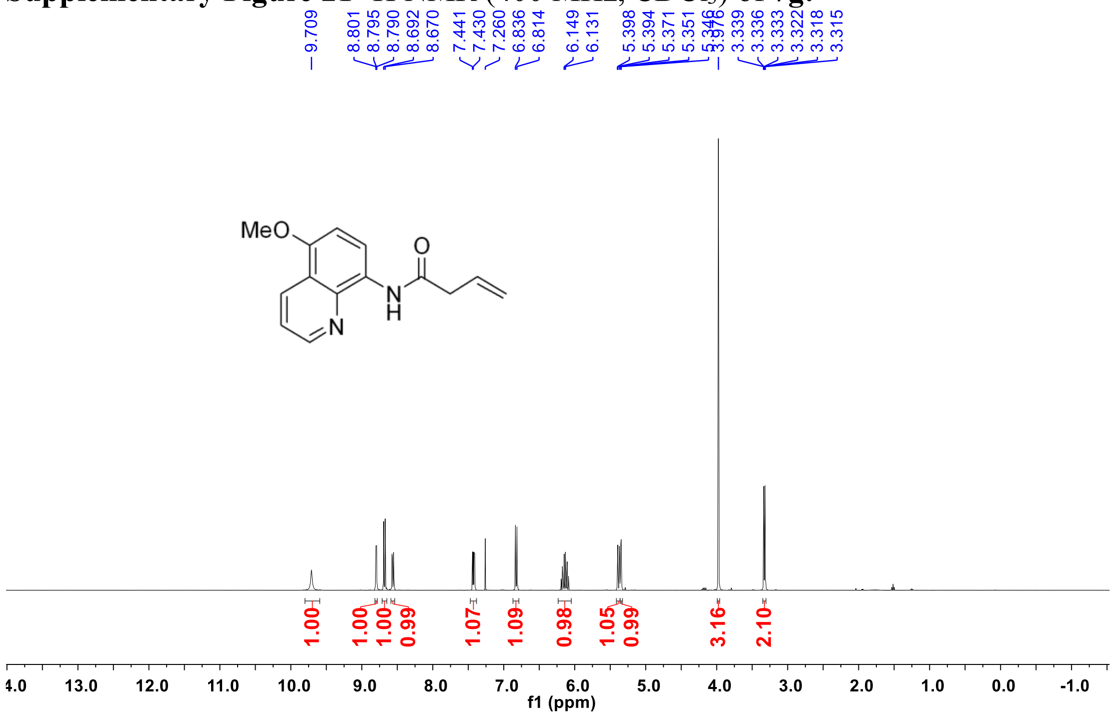

Supplementary Figure 22 ^{13}C NMR (100 MHz, $CDCl_3$) of **7g:**

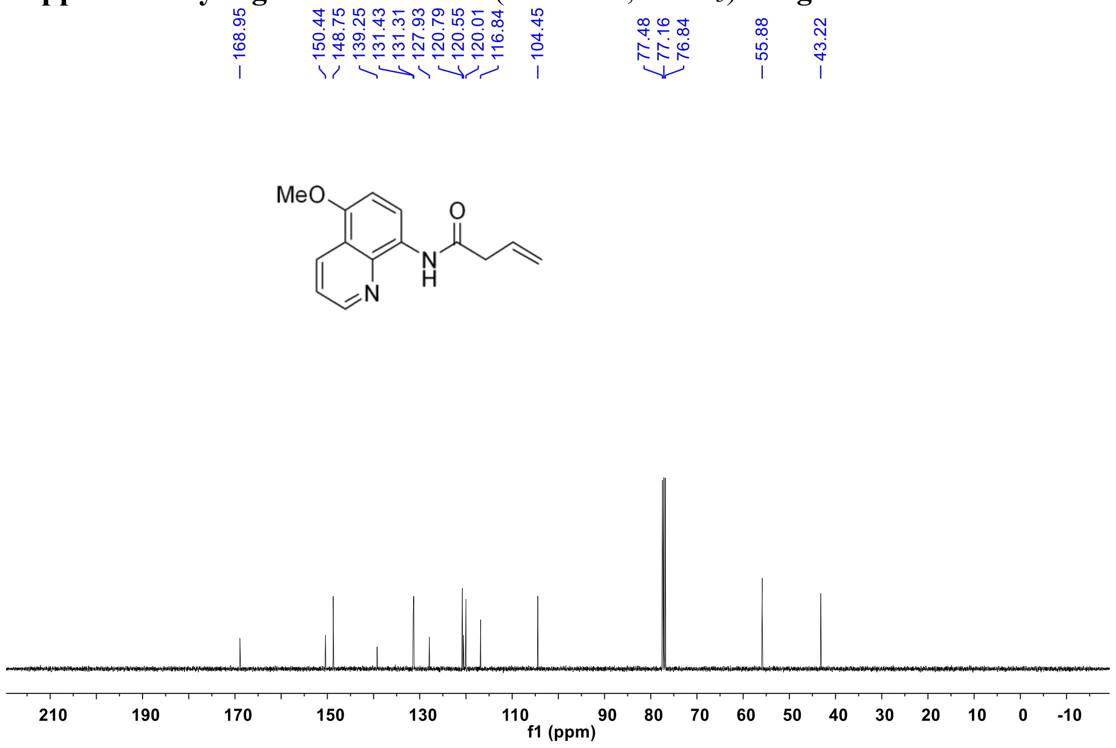

Supplementary Figure 23 ^{1}H NMR (400 MHz, $CDCl_3$)of **7h:**

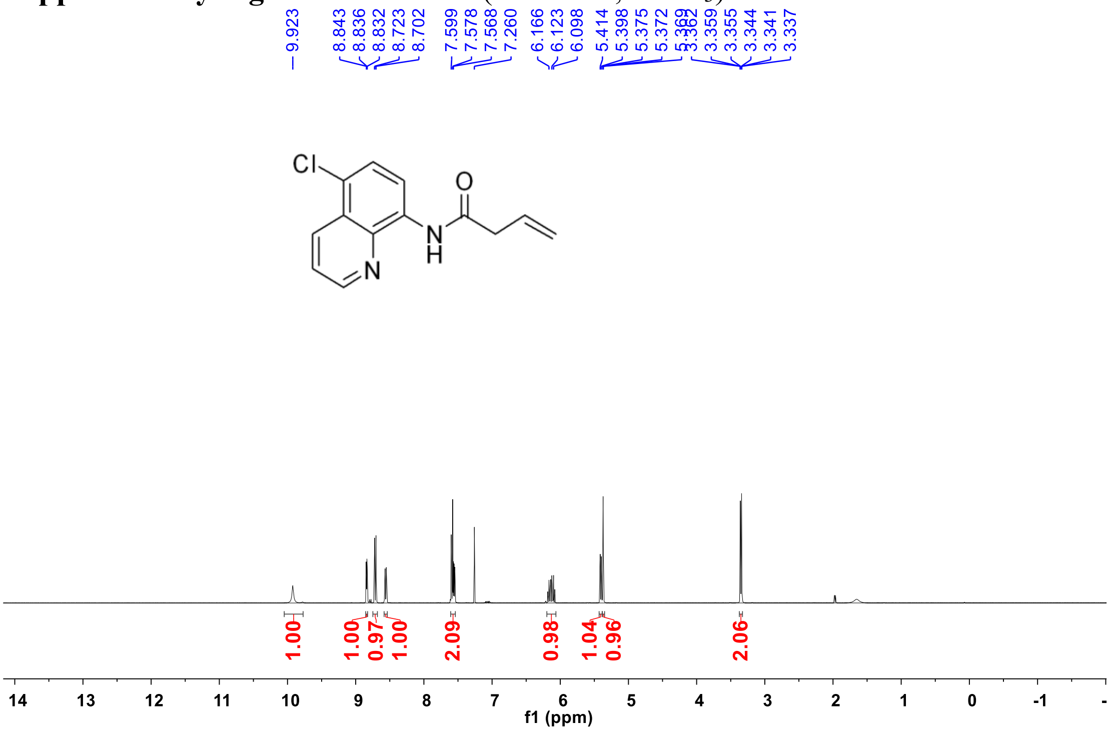

Supplementary Figure 24 ^{13}C NMR (100 MHz, $CDCl_3$) of **7h:**

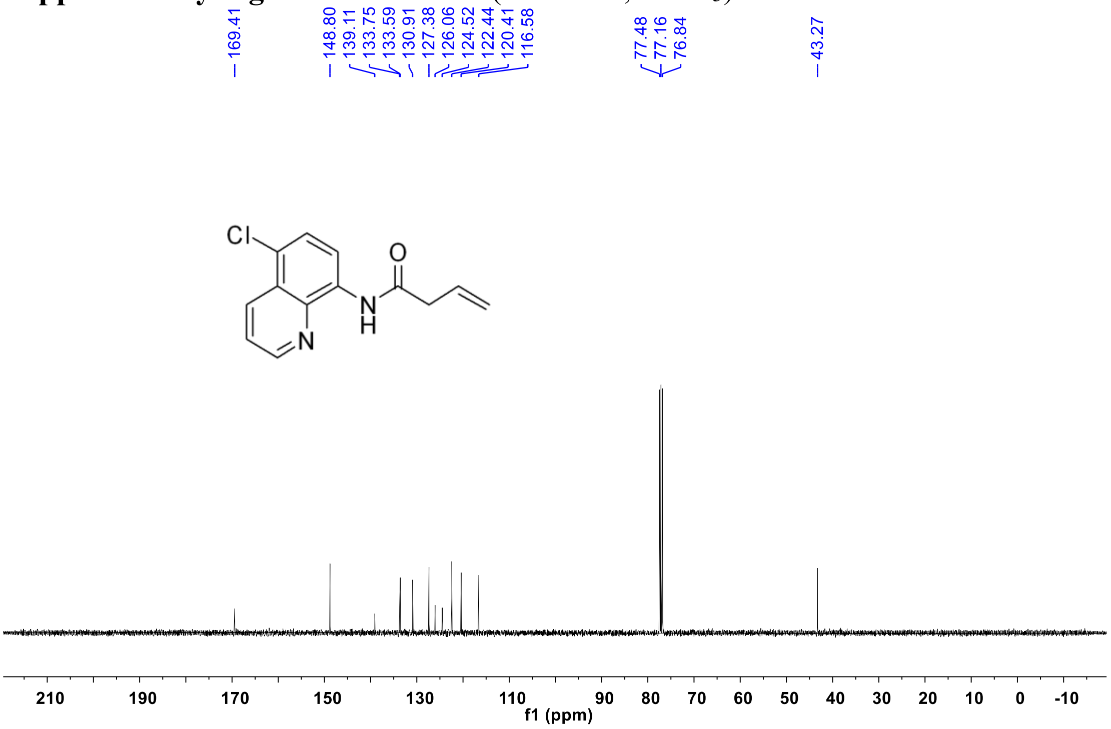

Supplementary Figure 25 ^{1}H NMR (500 MHz, $CDCl_3$) of **9a:**

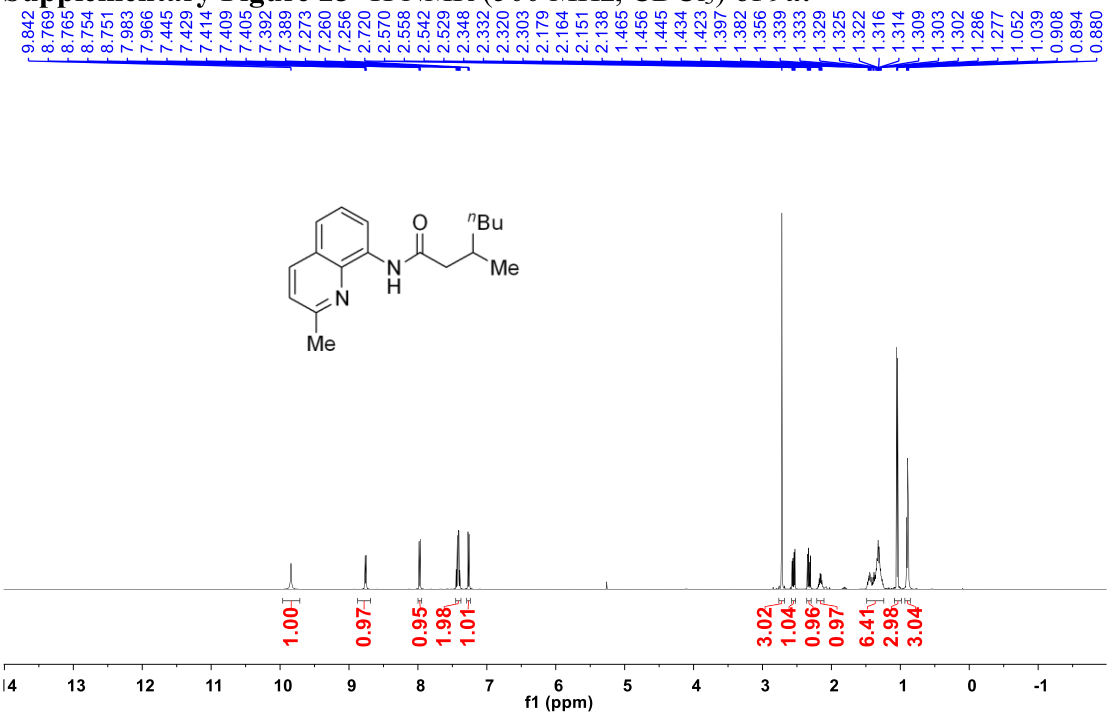

Supplementary Figure 26 ^{13}C NMR (125 MHz, $CDCl_3$) of **9a:**

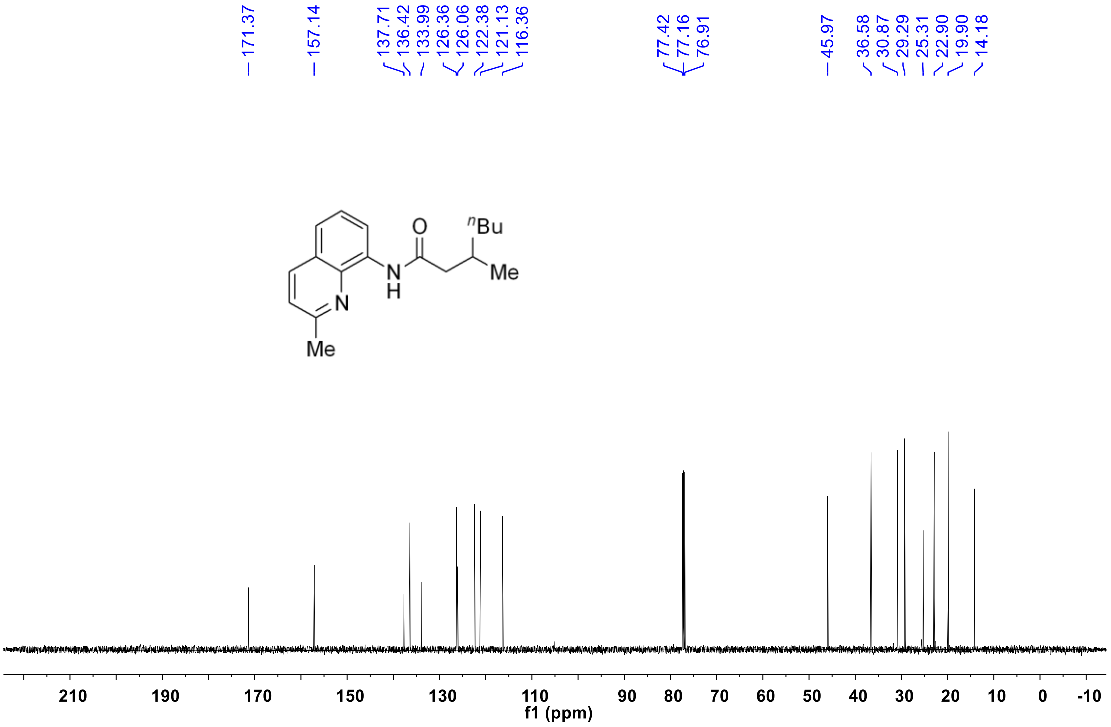

Supplementary Figure 27 ^{1}H NMR (300 MHz, CDCl$_3$) of **9b:**

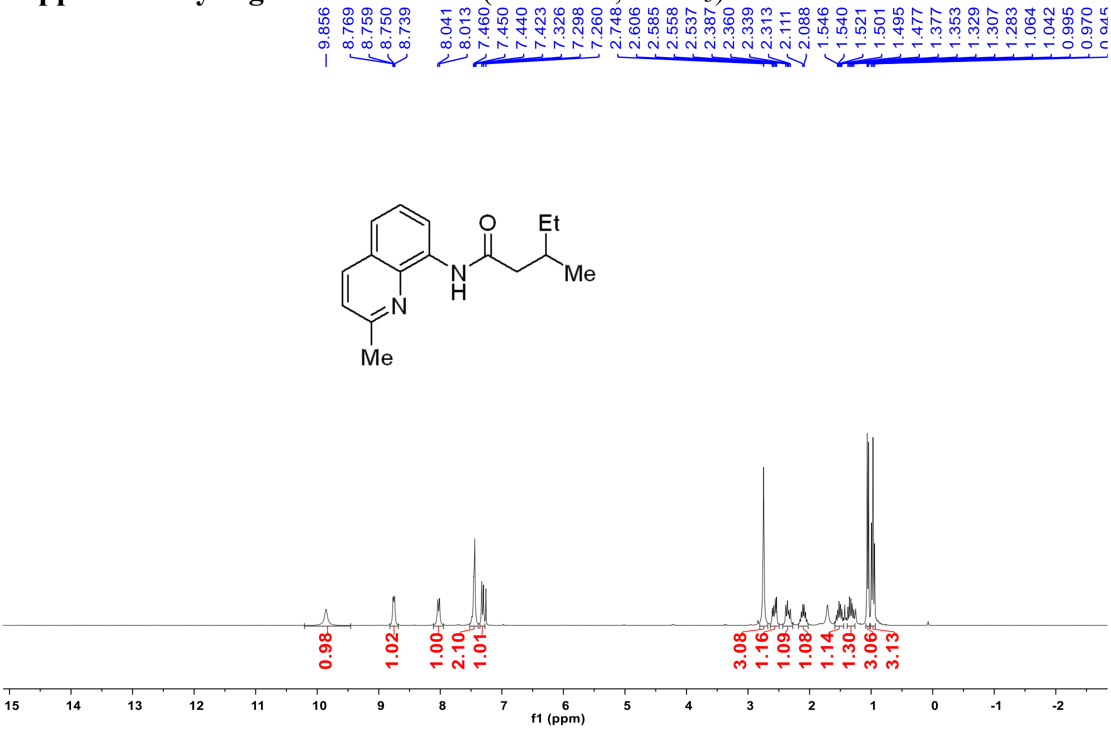

Supplementary Figure 28 ^{13}C NMR (75 MHz, CDCl$_3$) of **9b:**

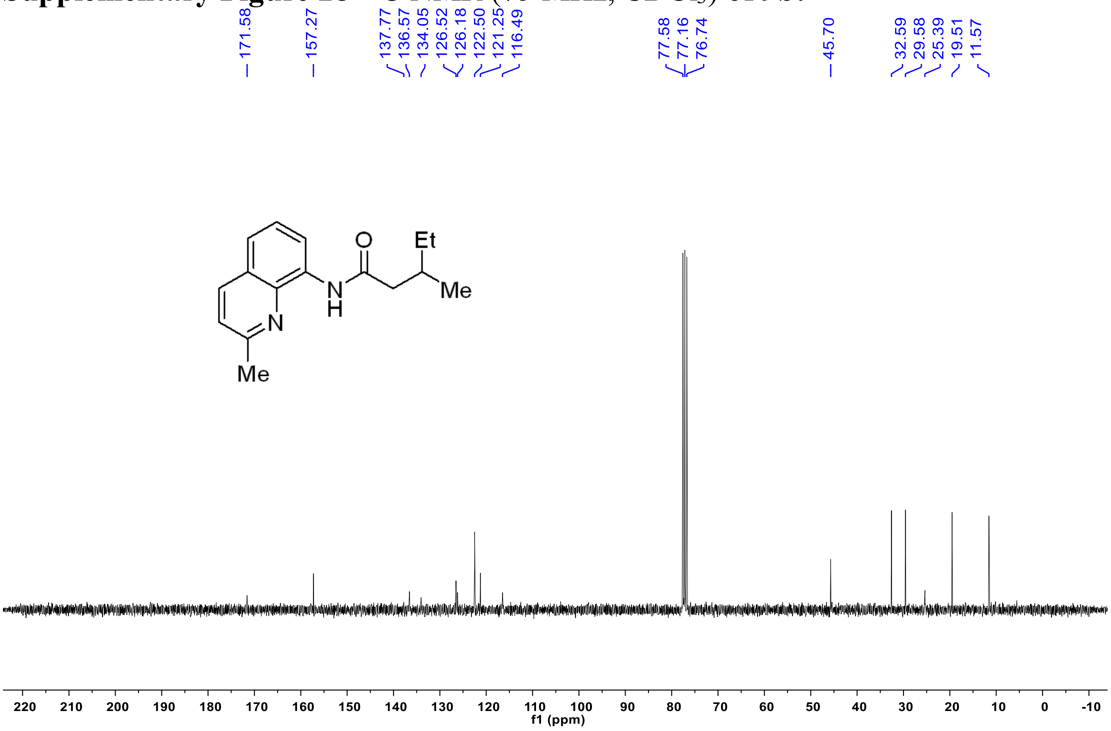

Supplementary Figure 29 ^{1}H NMR (500 MHz, $CDCl_3$) of **9c:**

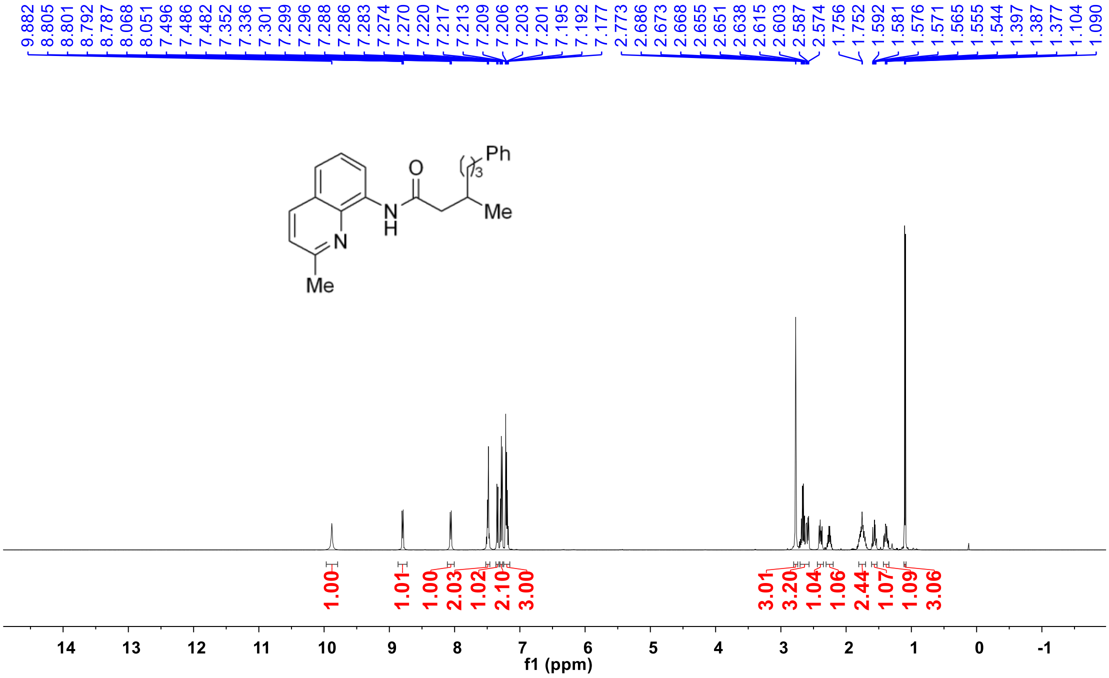

Supplementary Figure 30 ^{13}C NMR (100 MHz, $CDCl_3$) of **9c:**

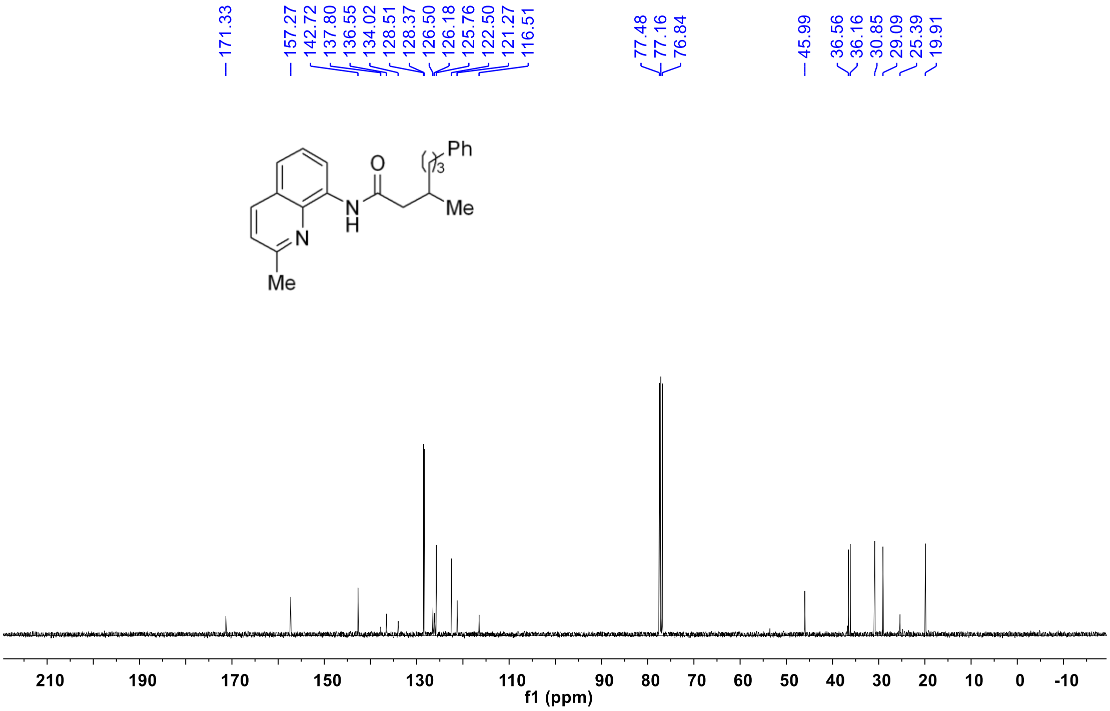

Supplementary Figure 31 ^{1}H NMR (400 MHz, $CDCl_3$) of **9d:**

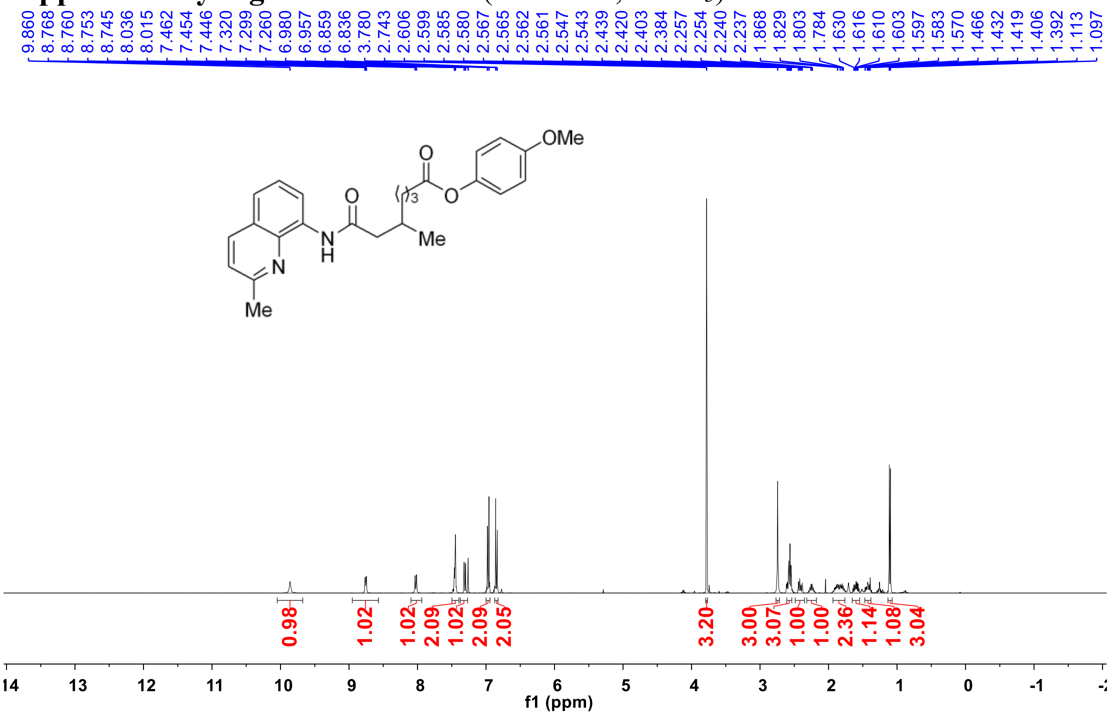

Supplementary Figure 32 ^{13}C NMR (100 MHz, $CDCl_3$) of **9d:**

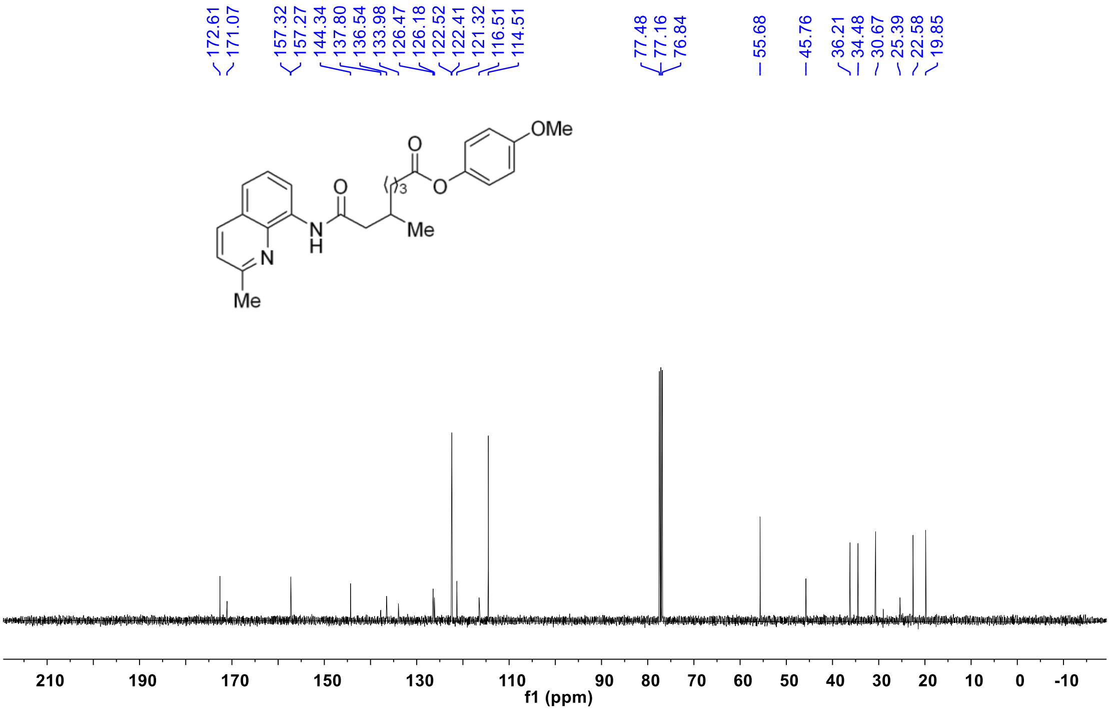

Supplementary Figure 33 ^{1}H NMR (500 MHz, $CDCl_3$) of **9e:**

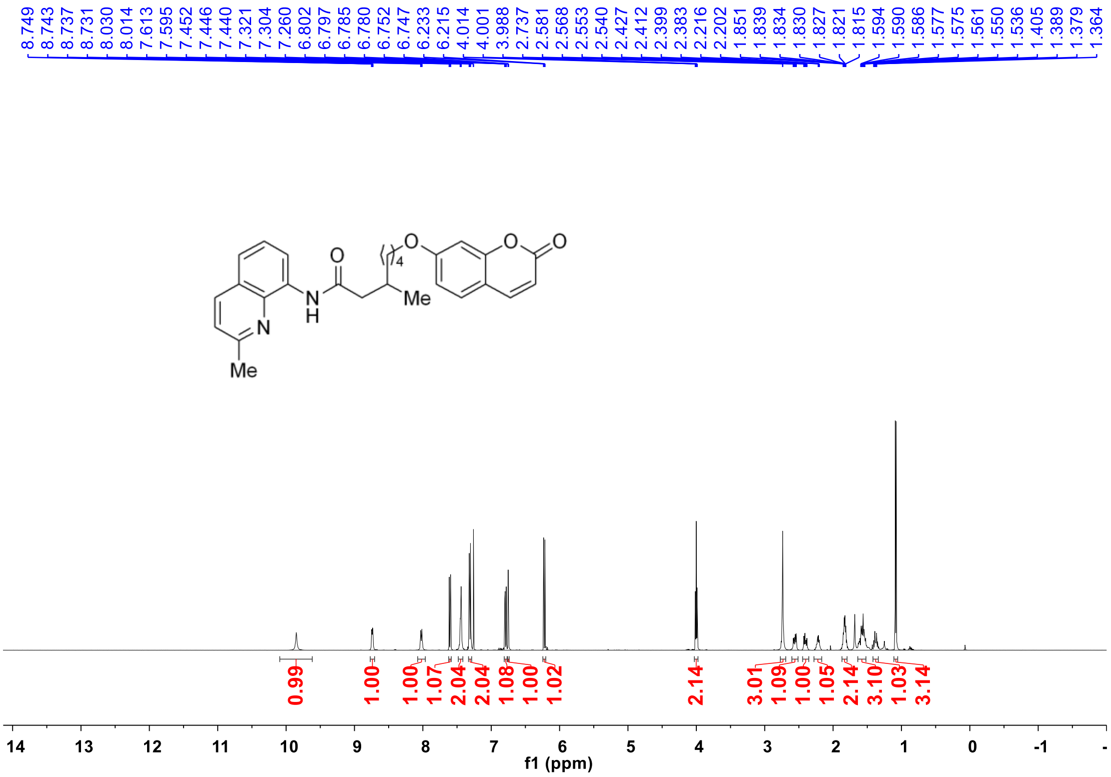

Supplementary Figure 34 ^{13}C NMR (125 MHz, $CDCl_3$) of **9e:**

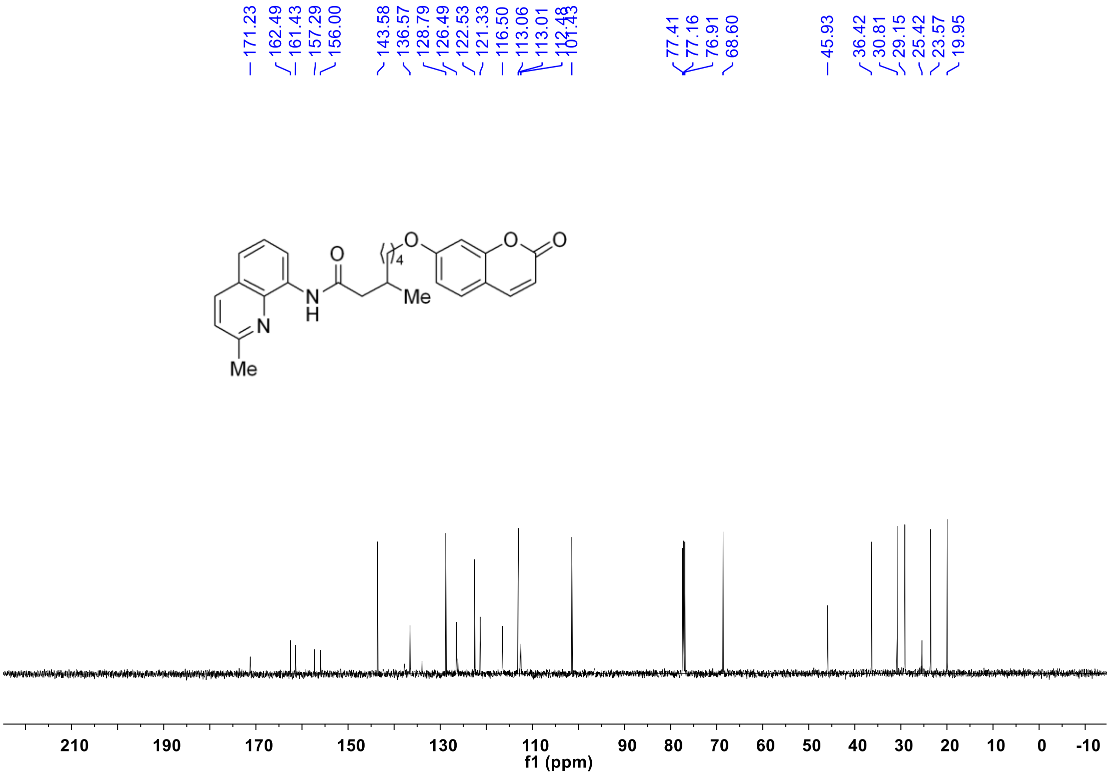

Supplementary Figure 35 ^{1}H NMR (500 MHz, $CDCl_3$) of **9f:**

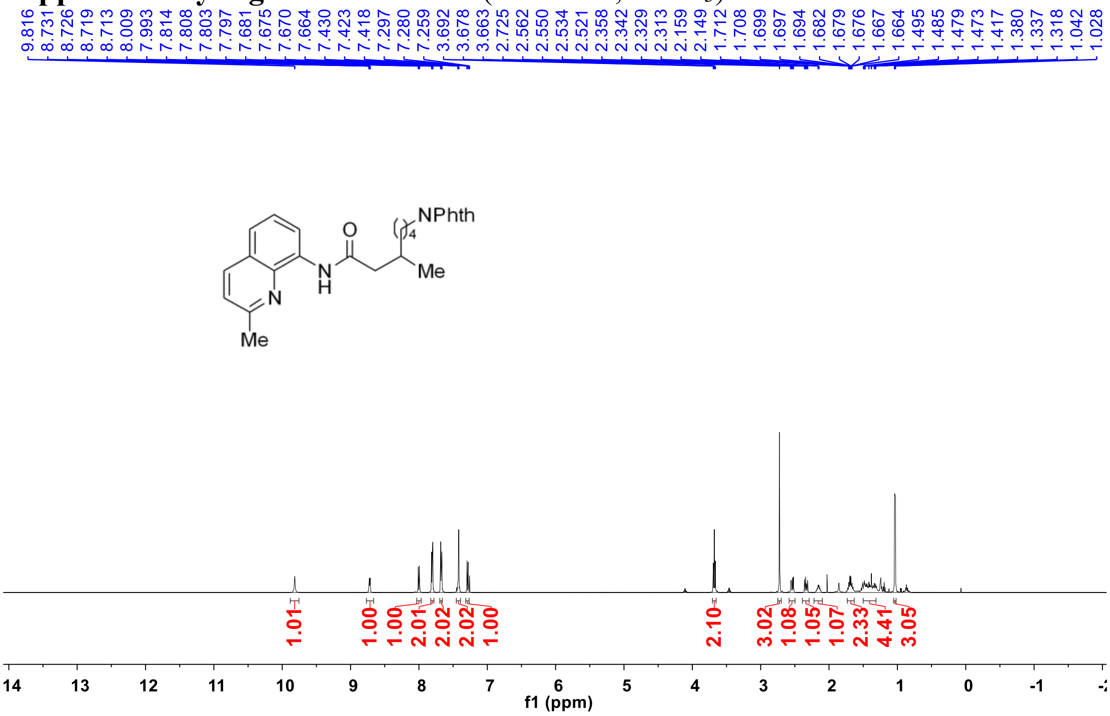

Supplementary Figure 36 ^{13}C NMR (125 MHz, $CDCl_3$) **9f:**

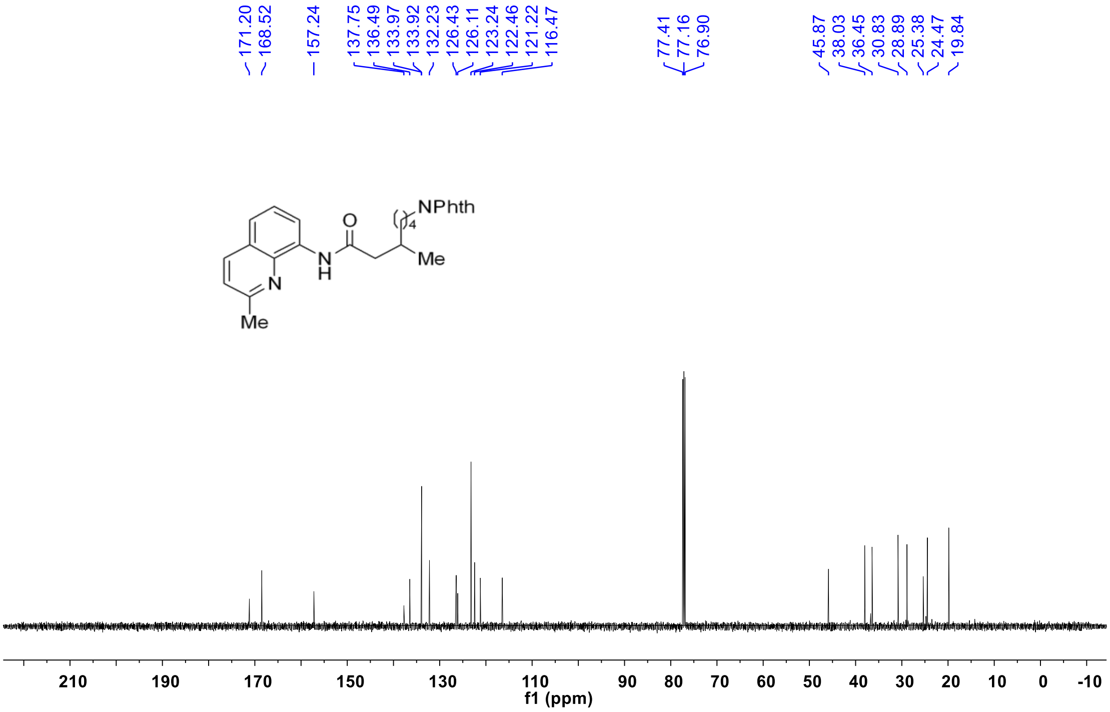

Supplementary Figure 37 ^{1}H NMR (400 MHz, $CDCl_3$) of **9g**:

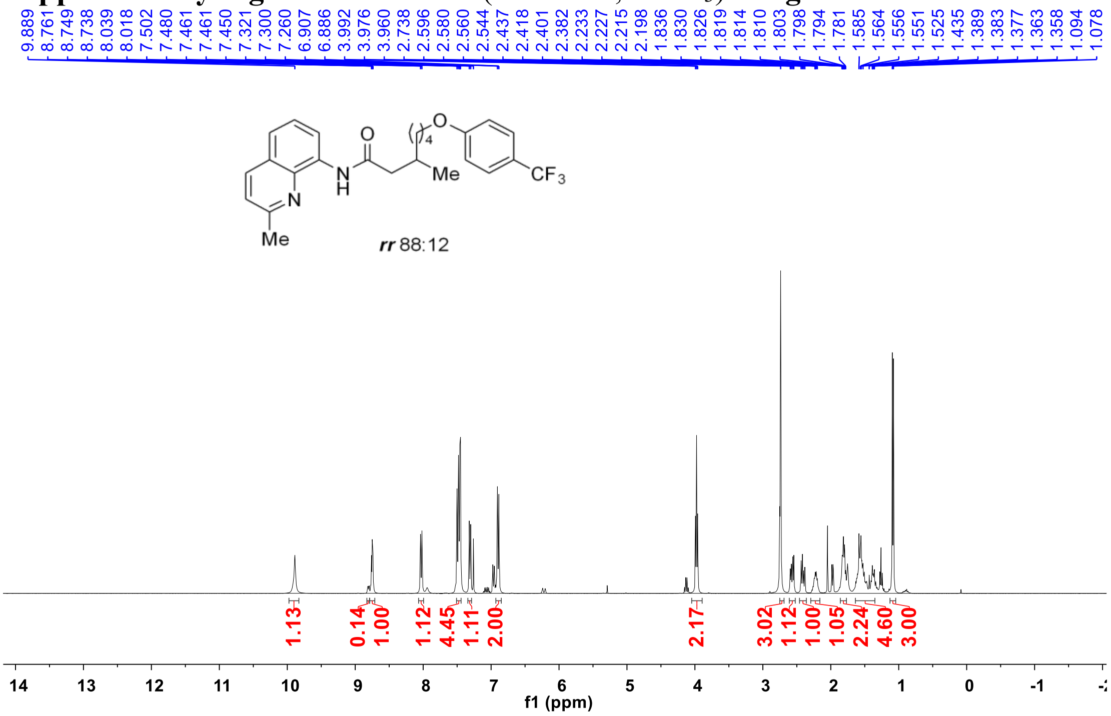

Supplementary Figure 38 ^{13}C NMR (100 MHz, $CDCl_3$) of **9g**:

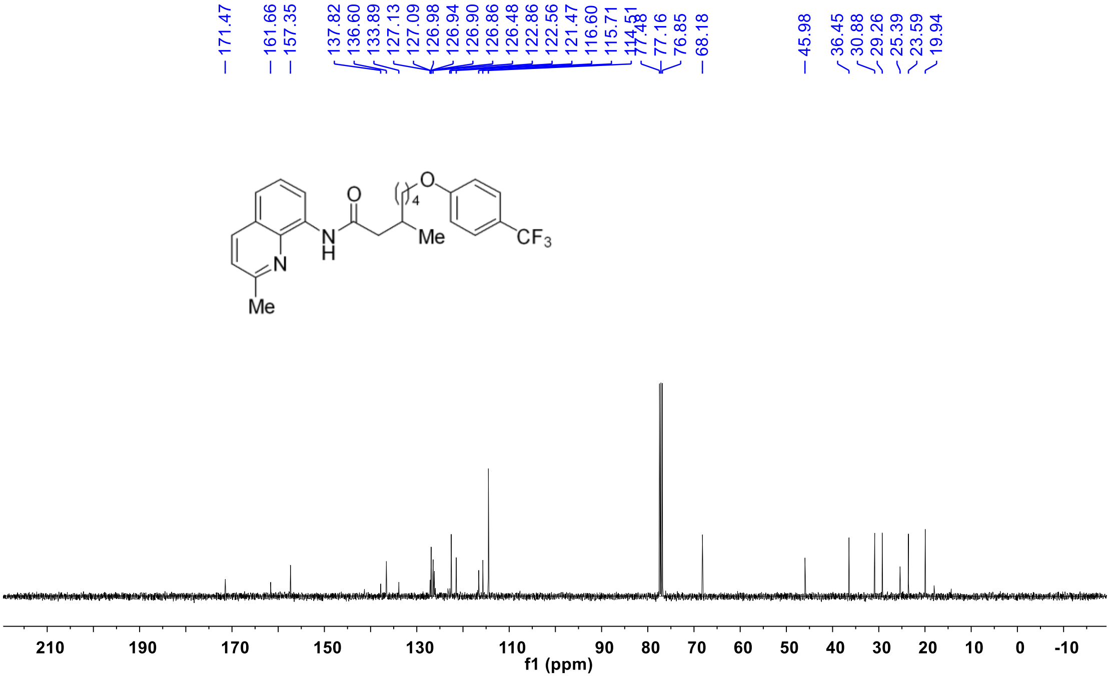

Supplementary Figure 39 ^{1}H NMR (300 MHz, $CDCl_3$) of **9h:**

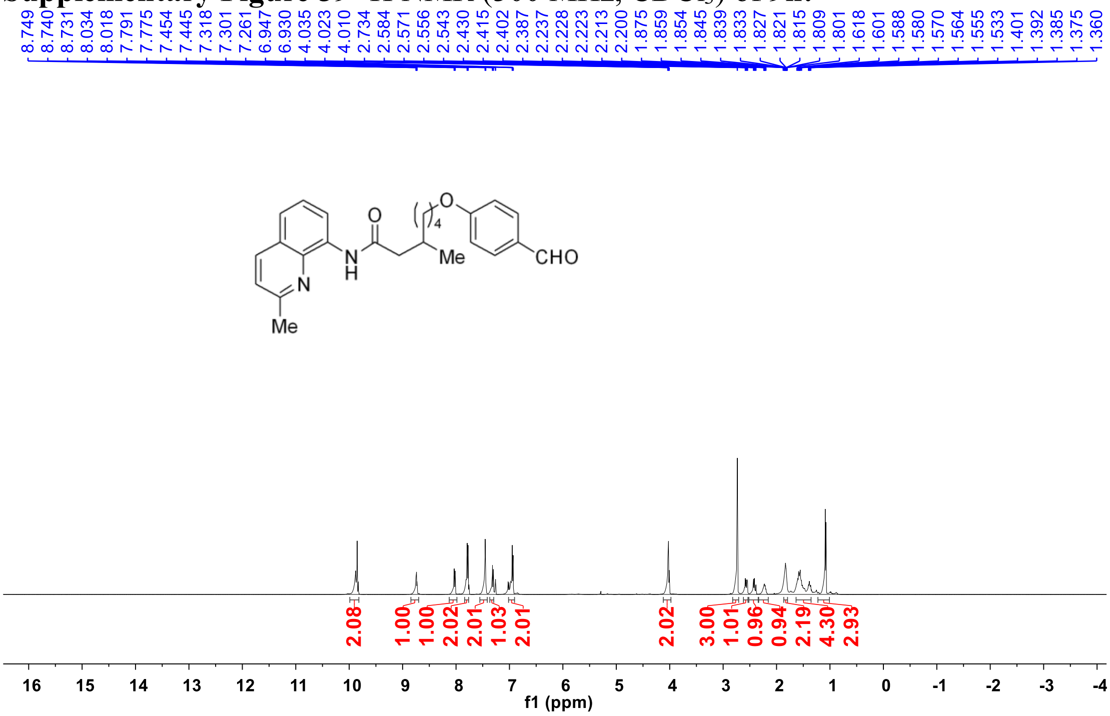

Supplementary Figure 40 ^{13}C NMR (100 MHz, $CDCl_3$) of **9h:**

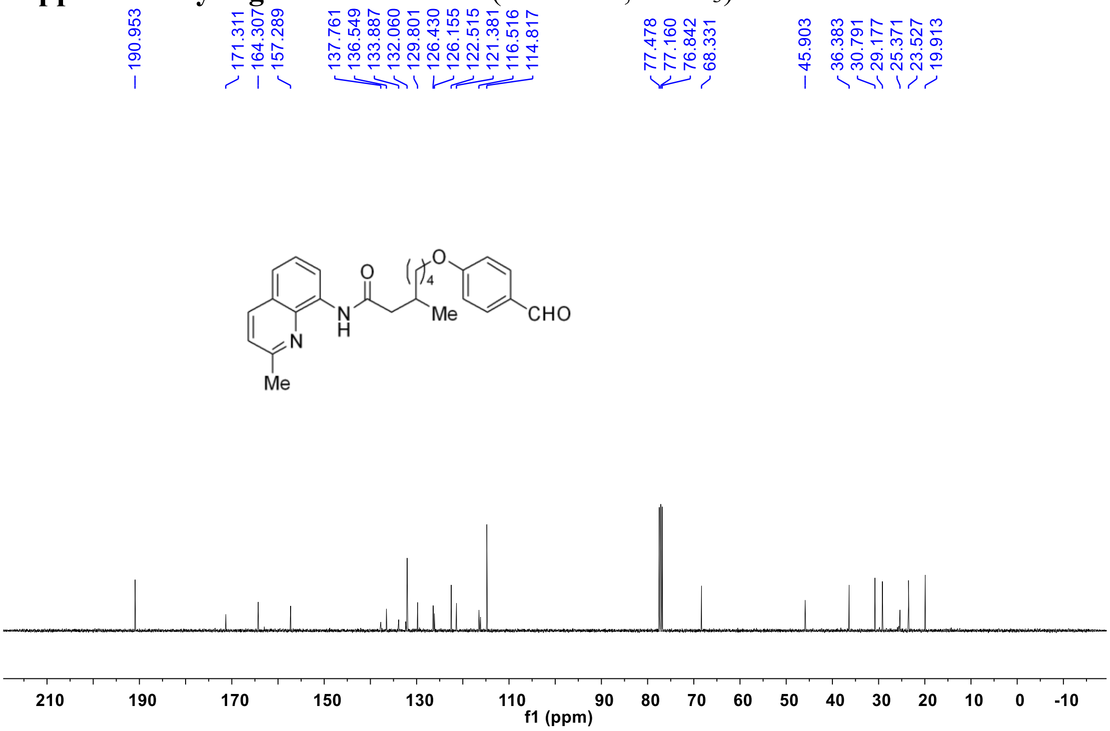

Supplementary Figure 41 ^{1}H NMR (500 MHz, $CDCl_3$) of **9i:**

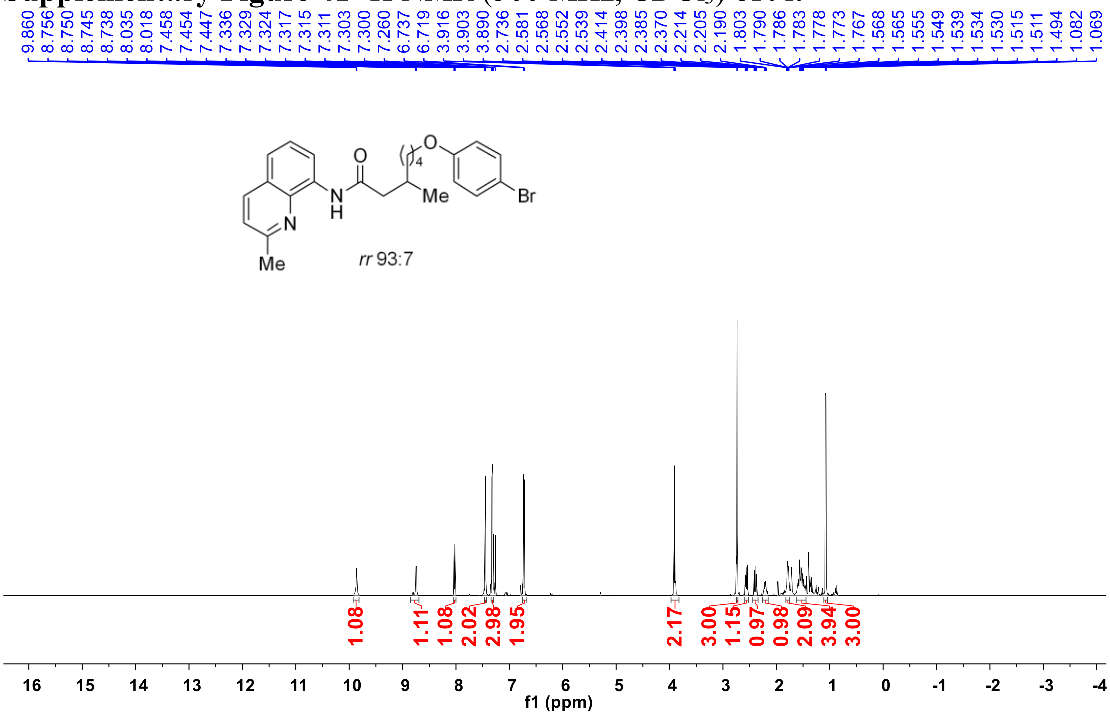

Supplementary Figure 42 ^{13}C NMR (100 MHz, $CDCl_3$) of **9i:**

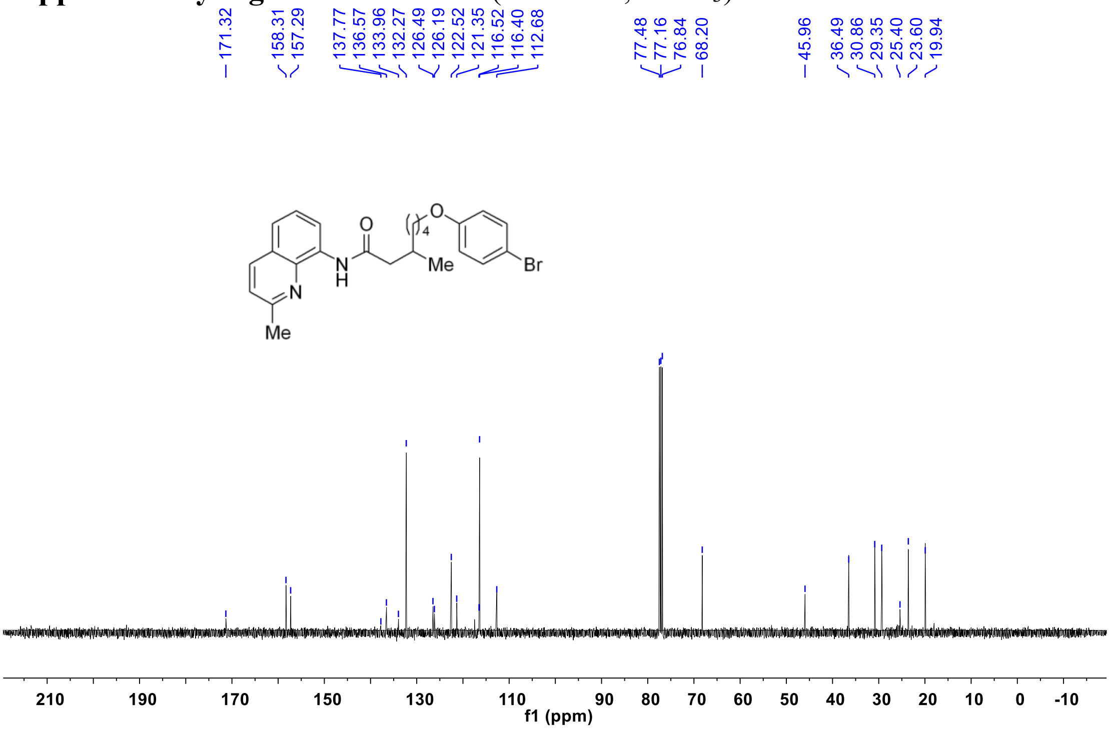

Supplementary Figure 43 ^{1}H NMR (500 MHz, $CDCl_3$) of **9j:**

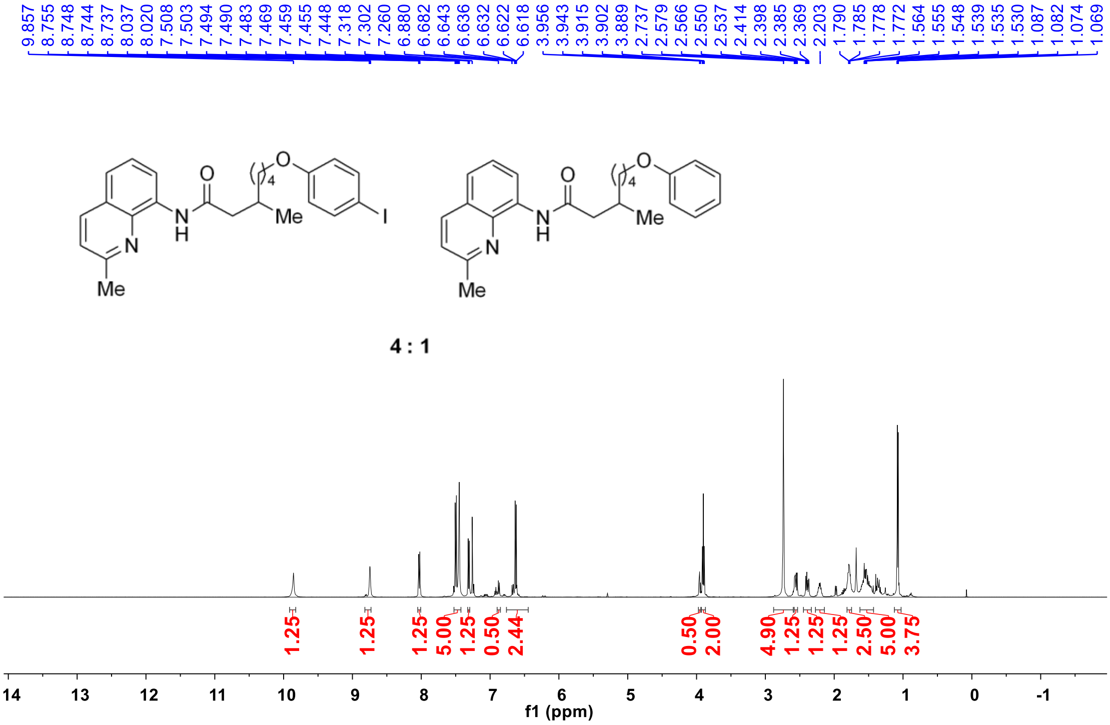

Supplementary Figure 44 ^{13}C NMR (125 MHz, $CDCl_3$) of **9j:**

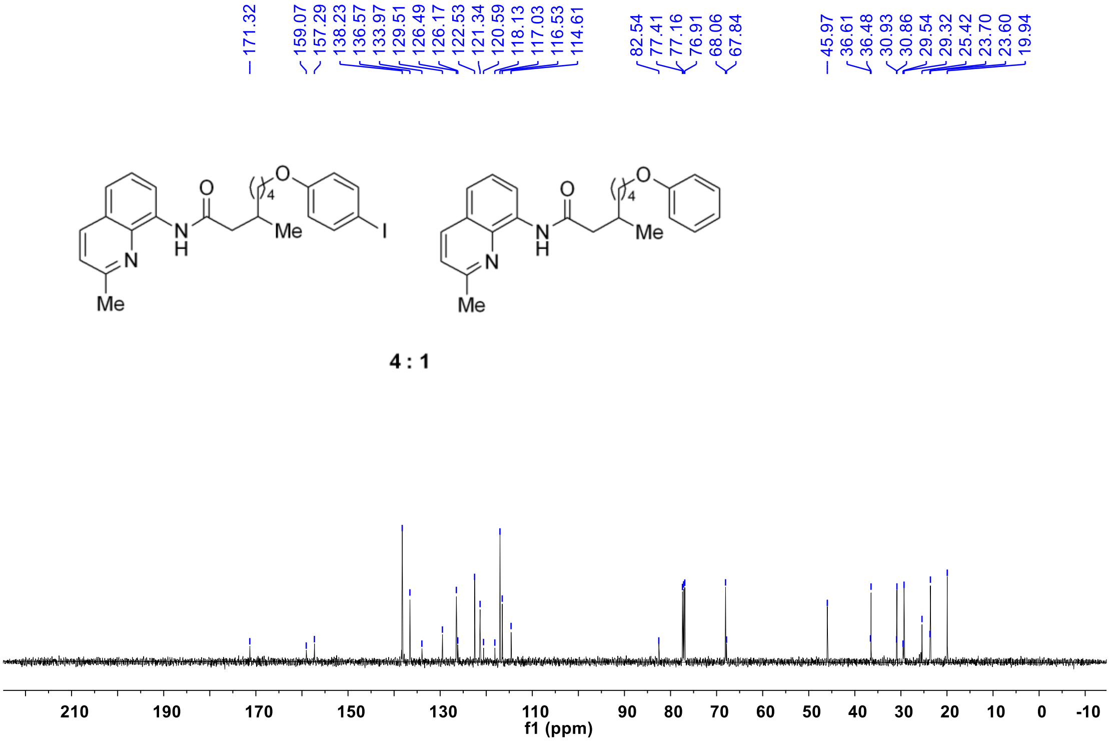

Supplementary Figure 45 ^{1}H NMR (400 MHz, $CDCl_3$) of **9k:**

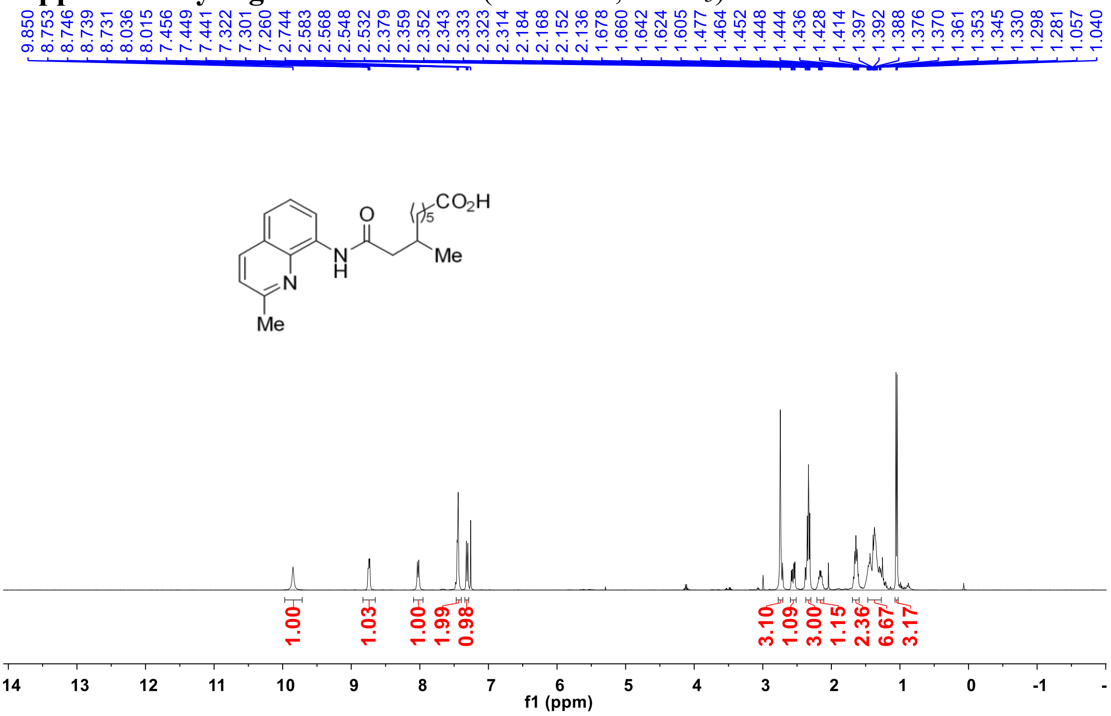

Supplementary Figure 46 ^{13}C NMR (100 MHz, $CDCl_3$) of **9k:**

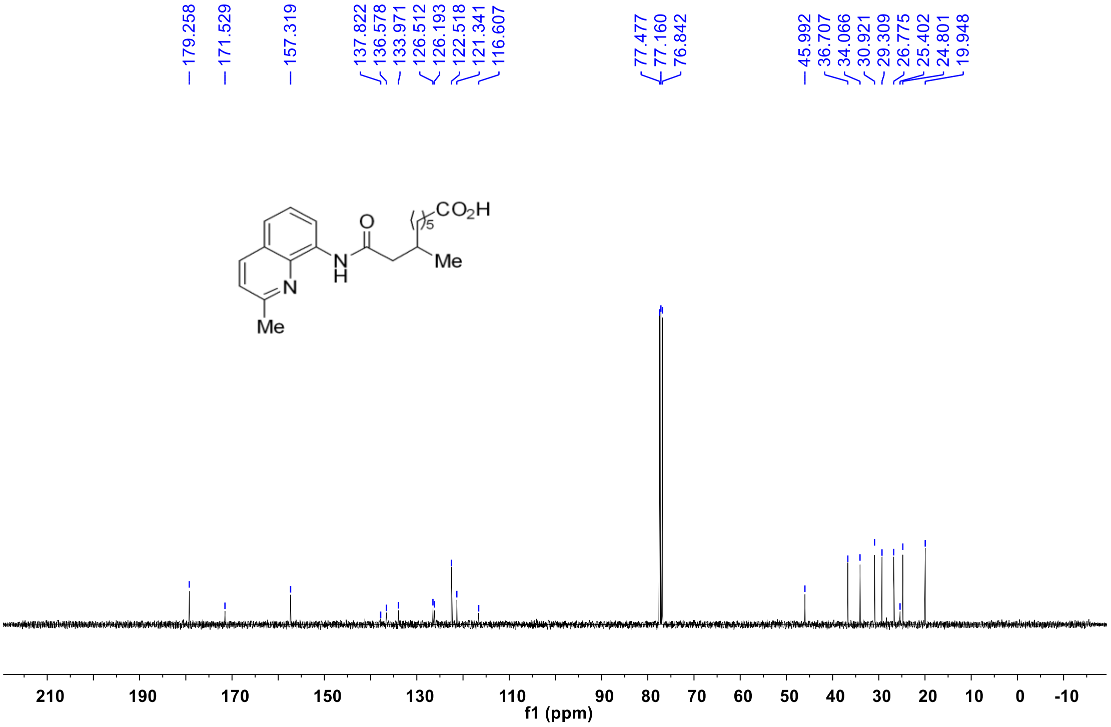

Supplementary Figure 47 ^{1}H NMR (500 MHz, $CDCl_3$) of **9l:**

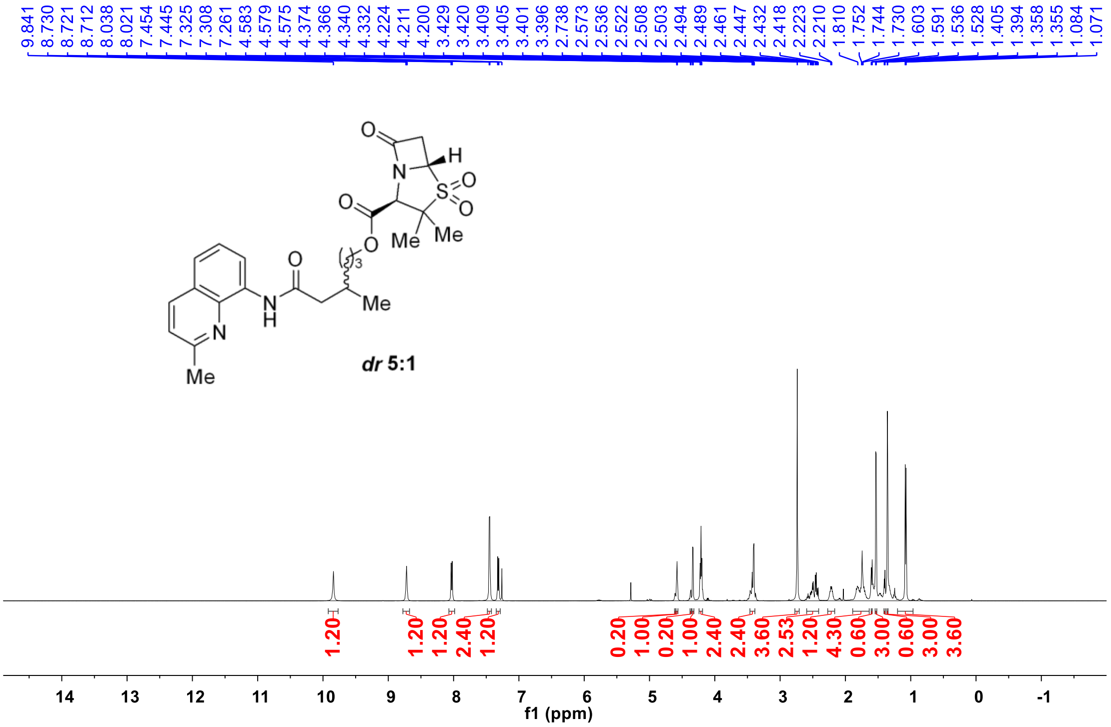

Supplementary Figure 48 ^{13}C NMR (125 MHz, $CDCl_3$) of **9l:**

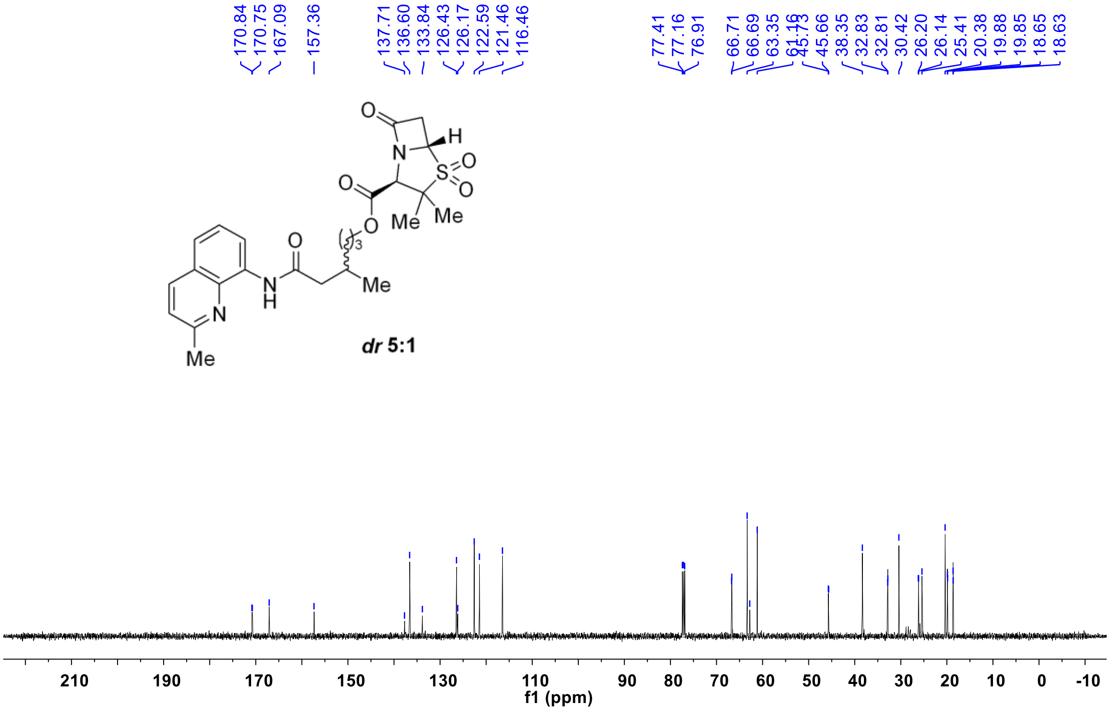

Supplementary Figure 49 ^{1}H NMR (400 MHz, $CDCl_3$) of **9m:**

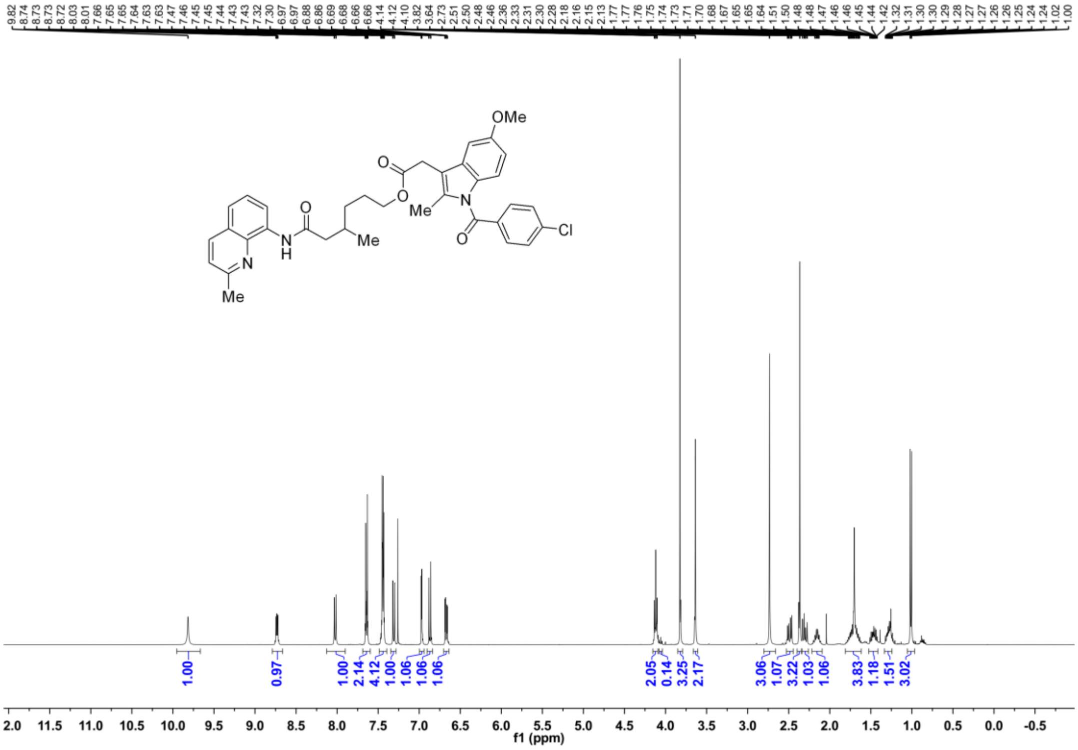

Supplementary Figure 50 ^{13}C NMR (126 MHz, $CDCl_3$) of **9m:**

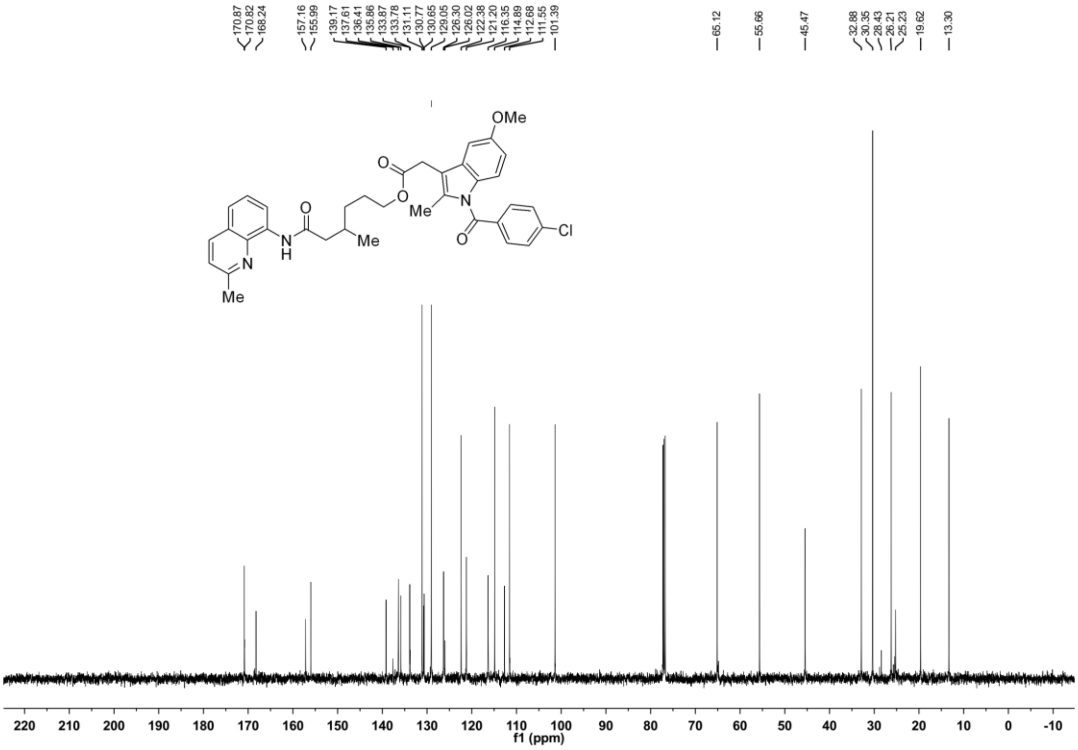

Supplementary Figure 51 ^{1}H NMR (400 MHz, $CDCl_3$) of **9n:**

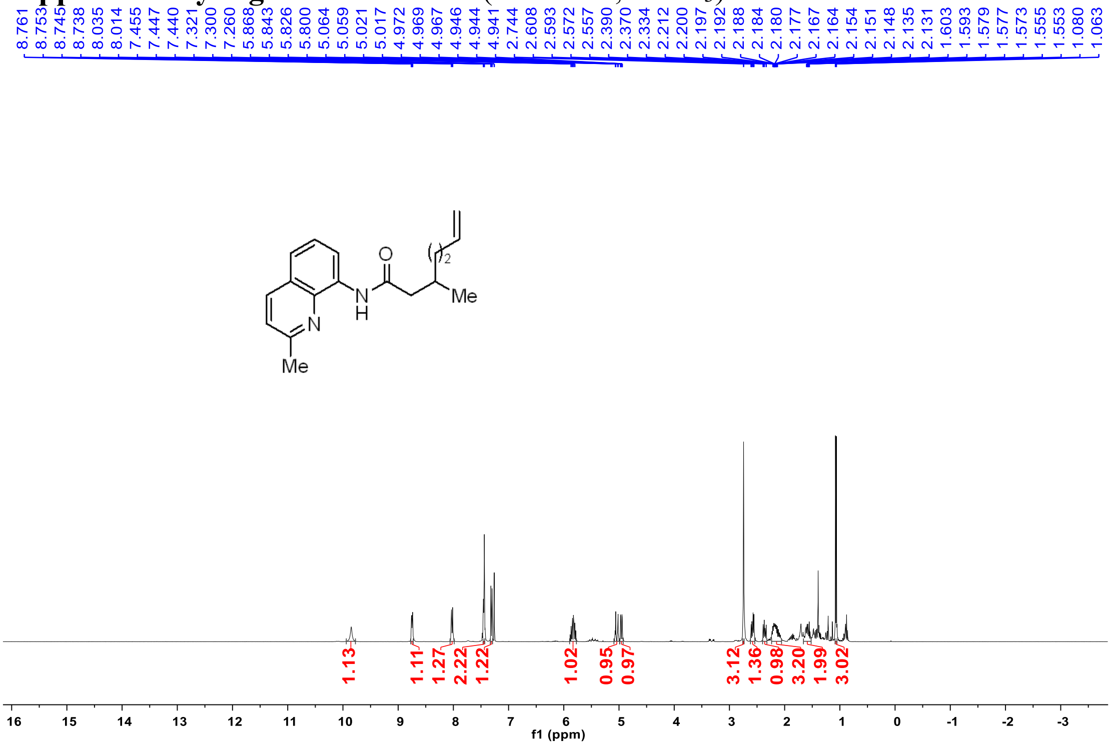

Supplementary Figure 52 ^{13}C NMR (100 MHz, $CDCl_3$) of **9n:**

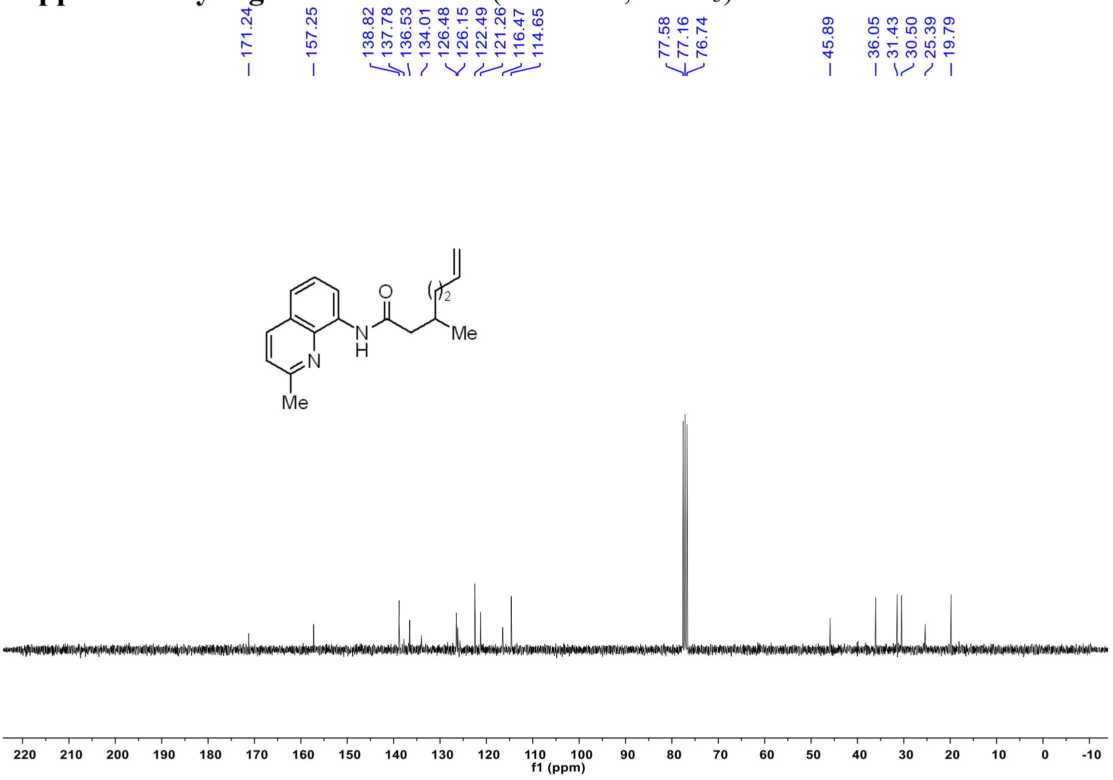

Supplementary Figure 53 ^{1}H NMR (400 MHz, $CDCl_3$) of **9o:**

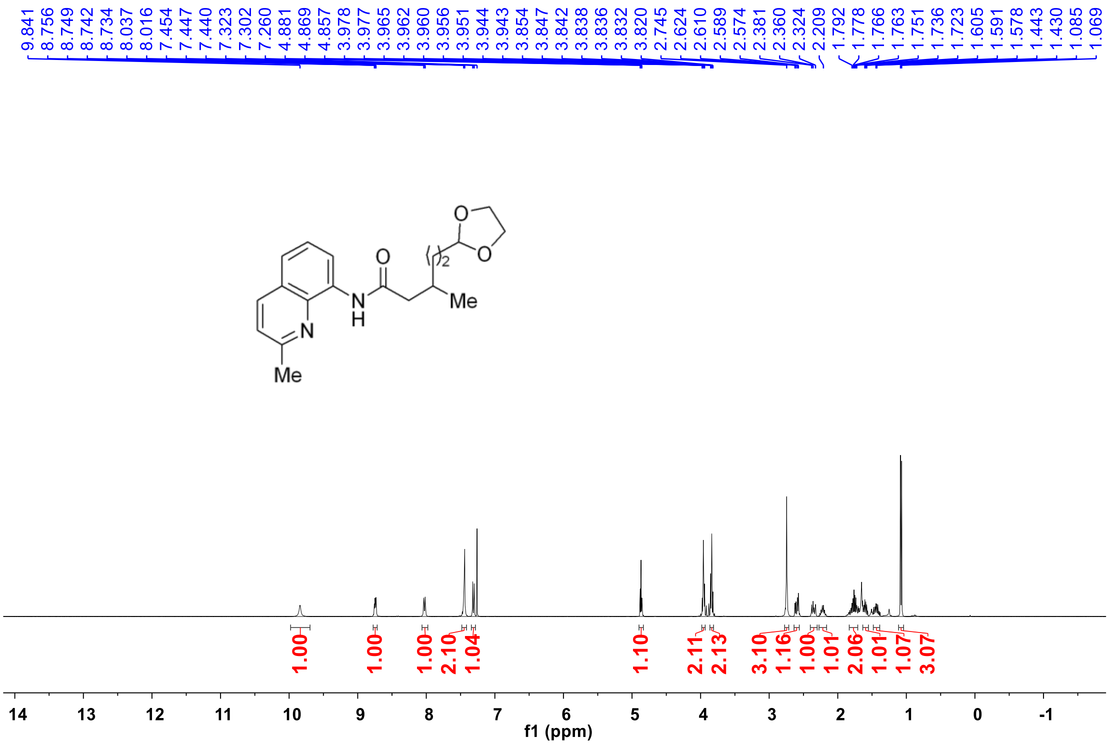

Supplementary Figure 54 ^{13}C NMR (125 MHz, $CDCl_3$) of **9o:**

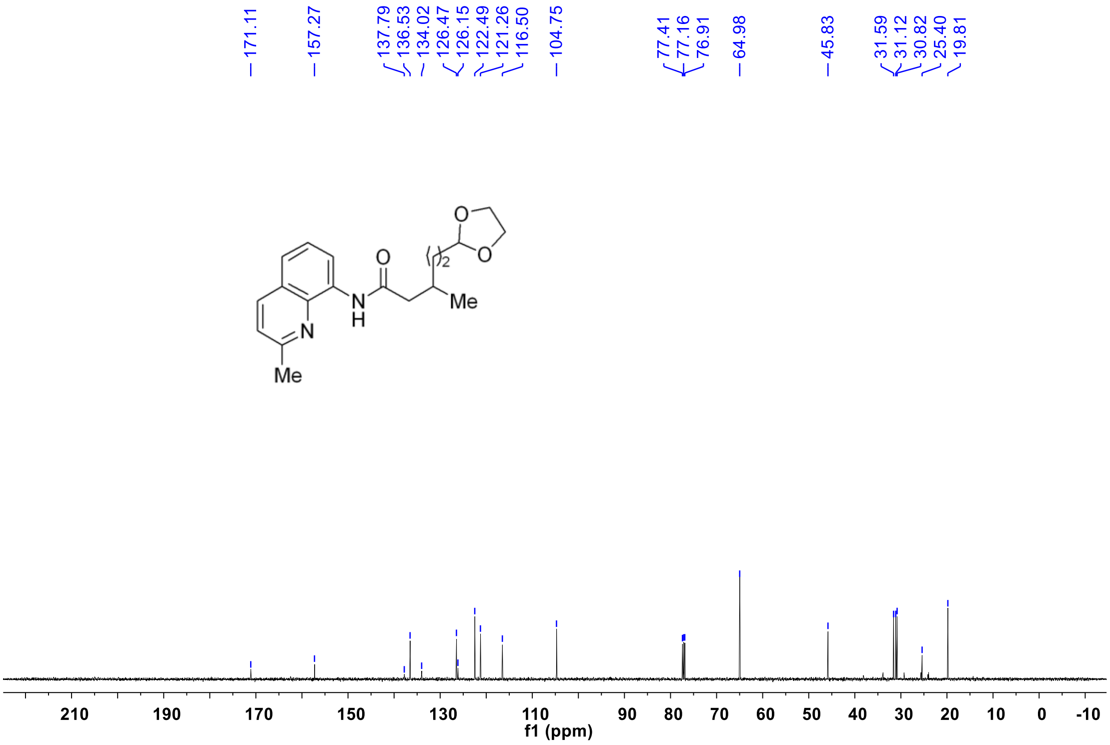

Supplementary Figure 55 ^{1}H NMR (500 MHz, $CDCl_3$) of **9p:**

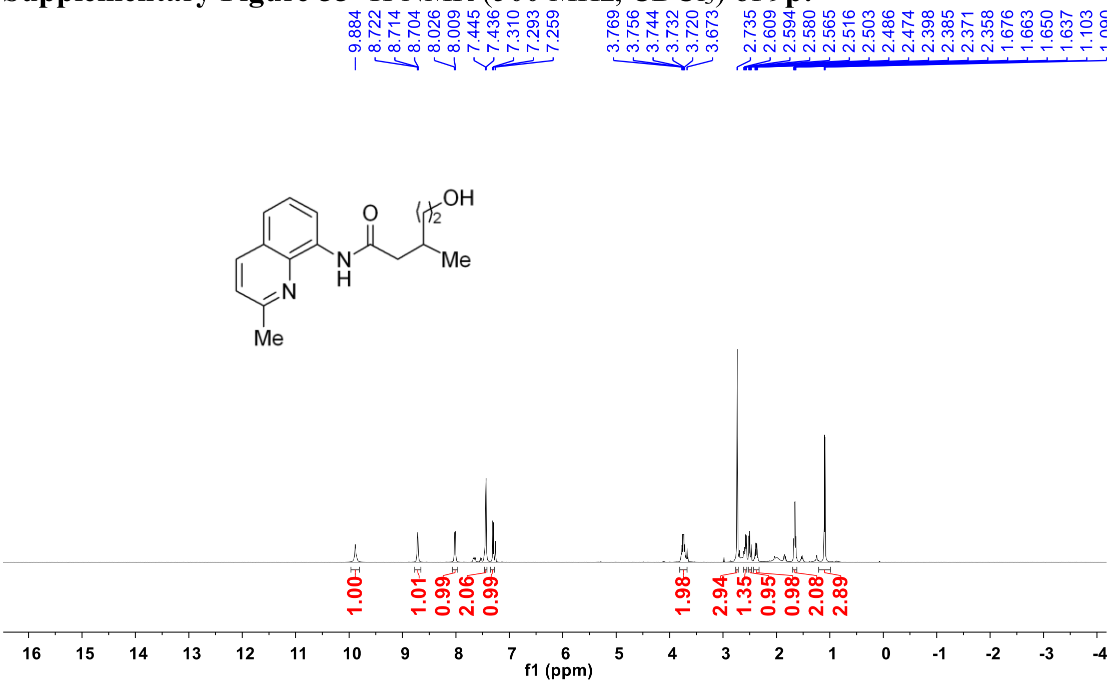

Supplementary Figure 56 ^{13}C NMR (125 MHz, $CDCl_3$) of **9p:**

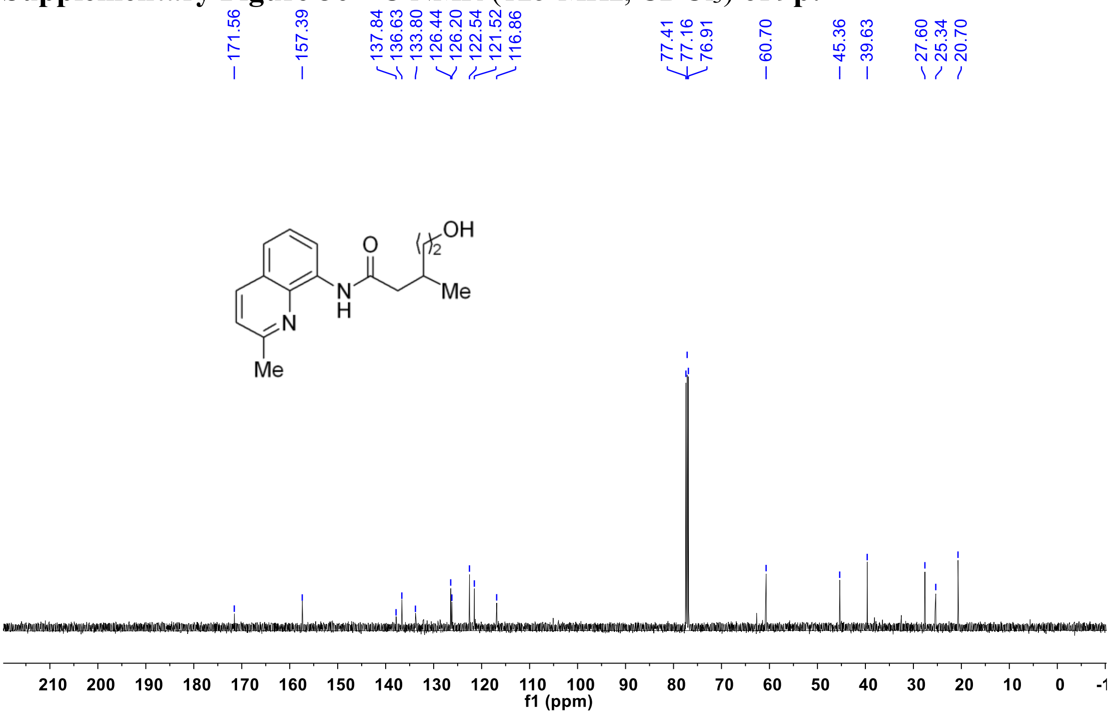

Supplementary Figure 57 ^{1}H NMR (500 MHz, $CDCl_3$) of **9q:**

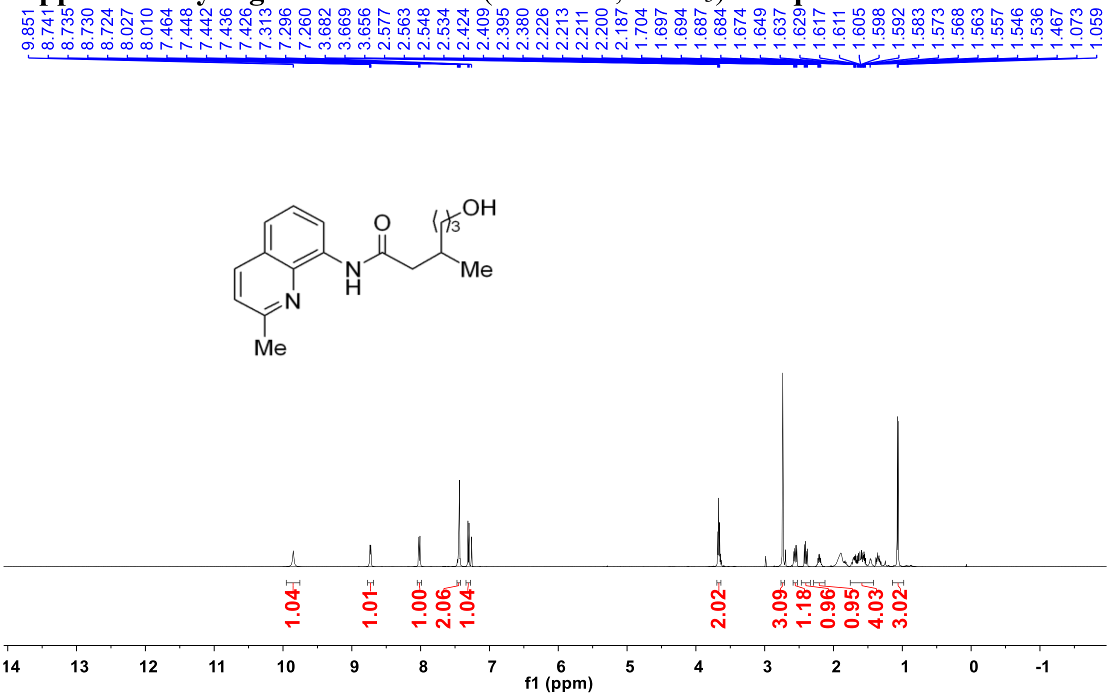

Supplementary Figure 58 ^{13}C NMR (100 MHz, $CDCl_3$) of **9q:**

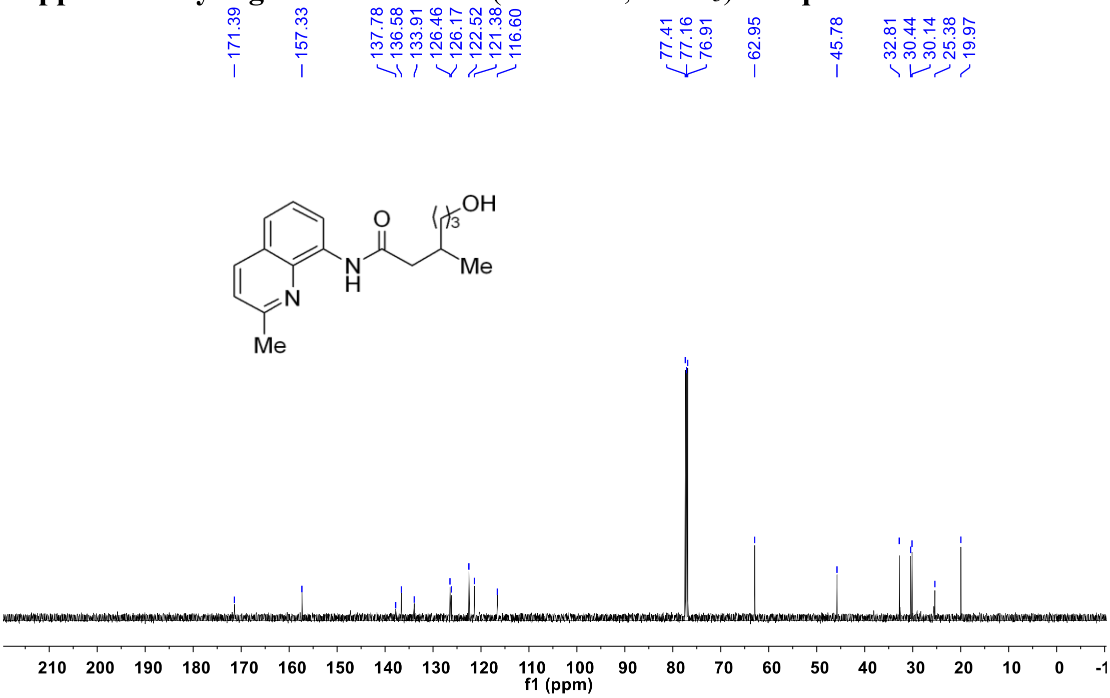

Supplementary Figure 59 ^{1}H NMR (400 MHz, DMSO-d_6) of **9r:**

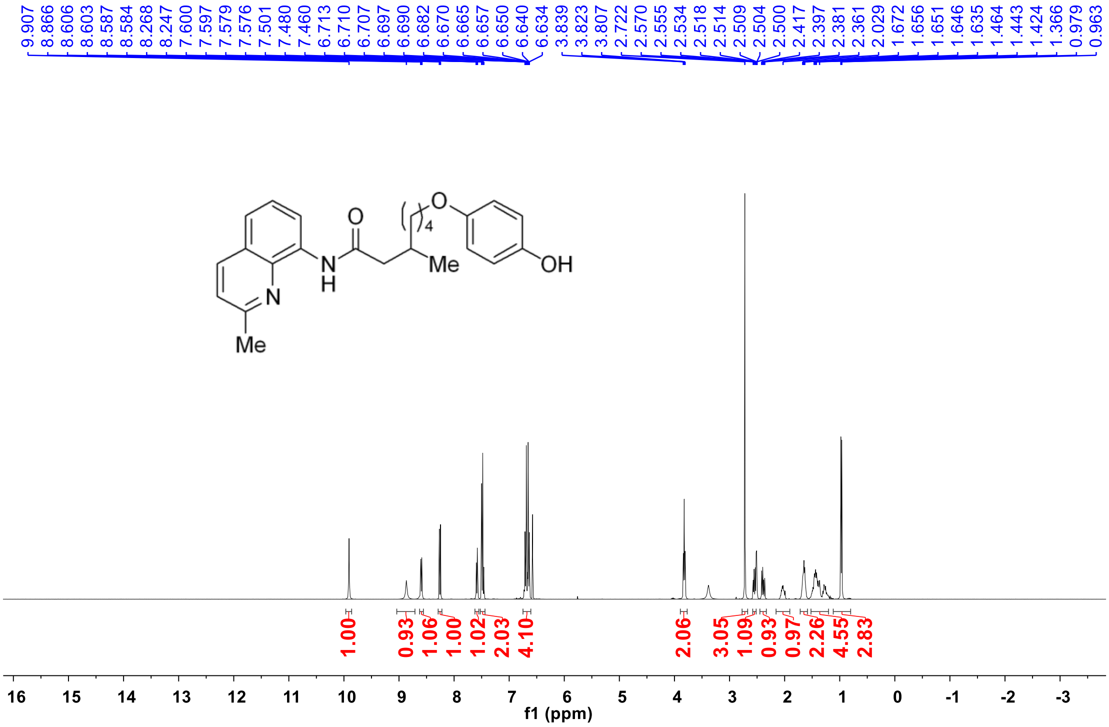

Supplementary Figure 60 ^{13}C NMR (100 MHz, DMSO-d_6) of **9r:**

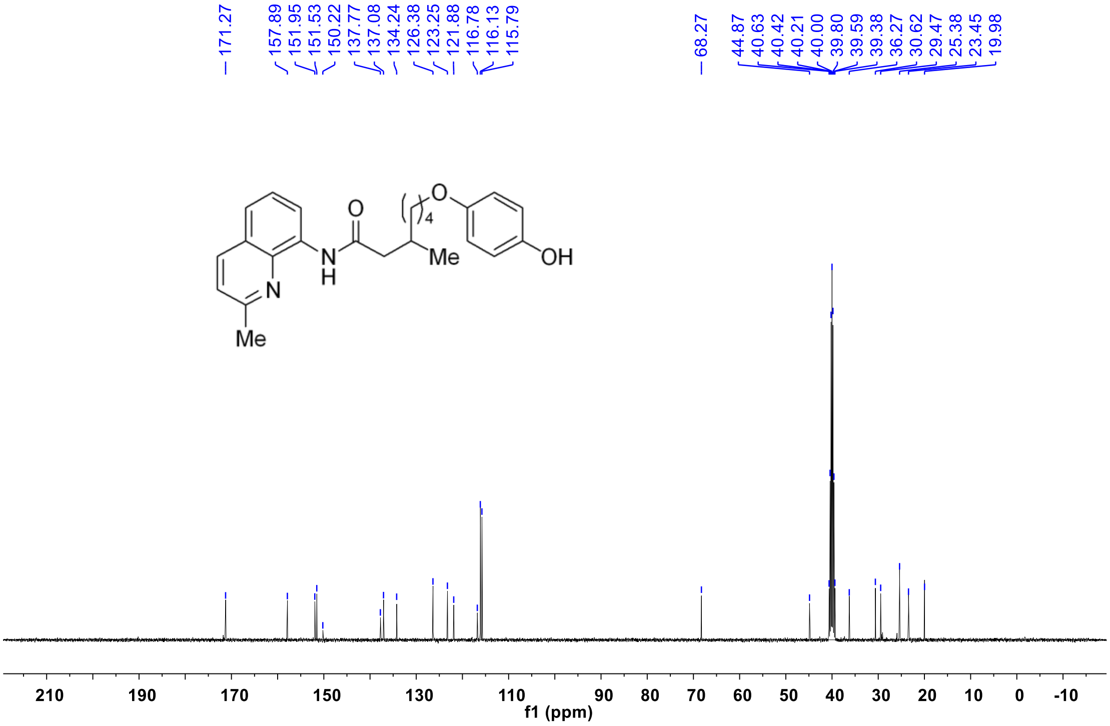

Supplementary Figure 61 ^{1}H NMR (500 MHz, $CDCl_3$) of **9s:**

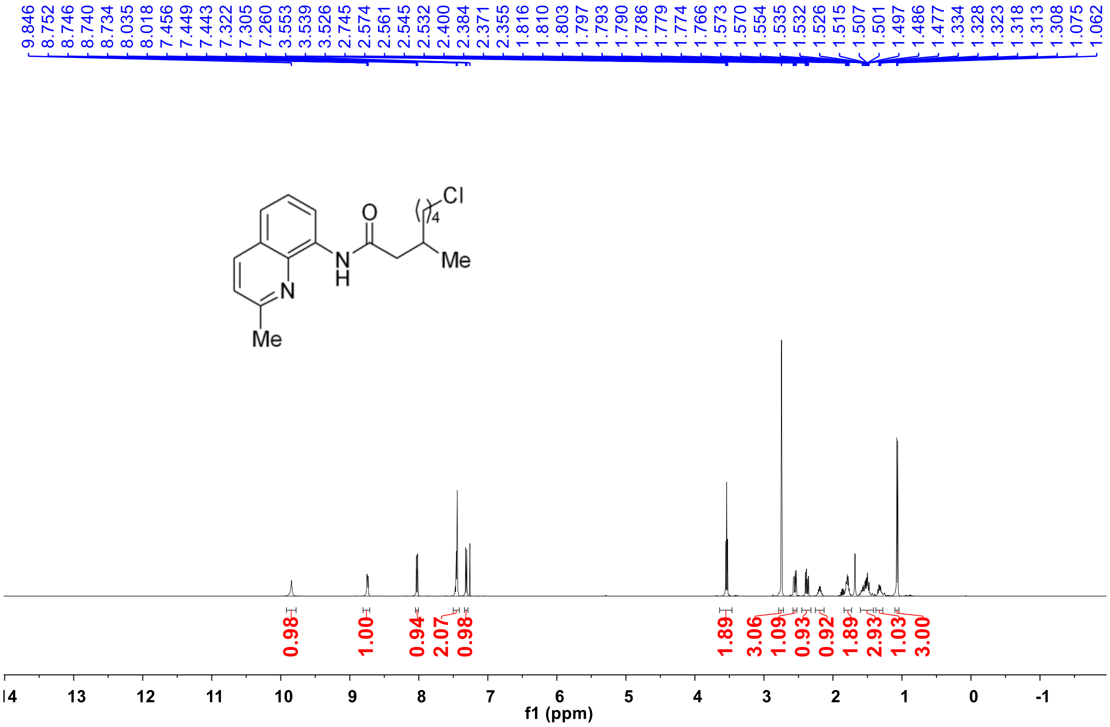

Supplementary Figure 62 ^{13}C NMR (125 MHz, $CDCl_3$) of **9s:**

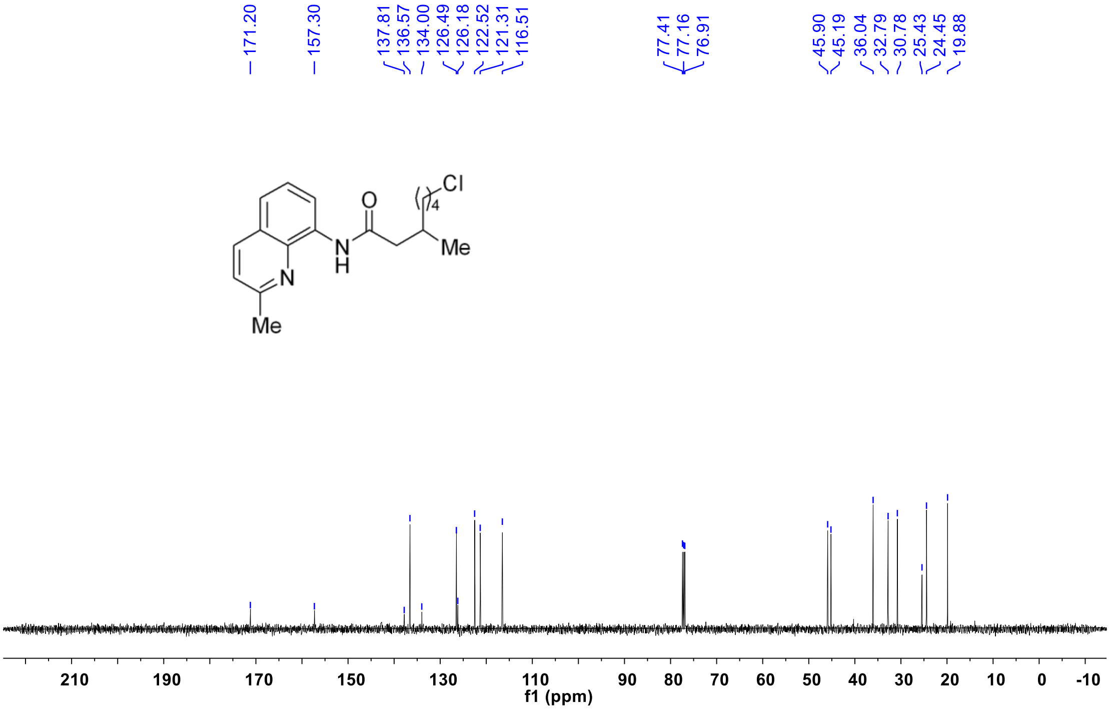

Supplementary Figure 63 ¹H NMR (500 MHz, $CDCl_3$) of **9t:**

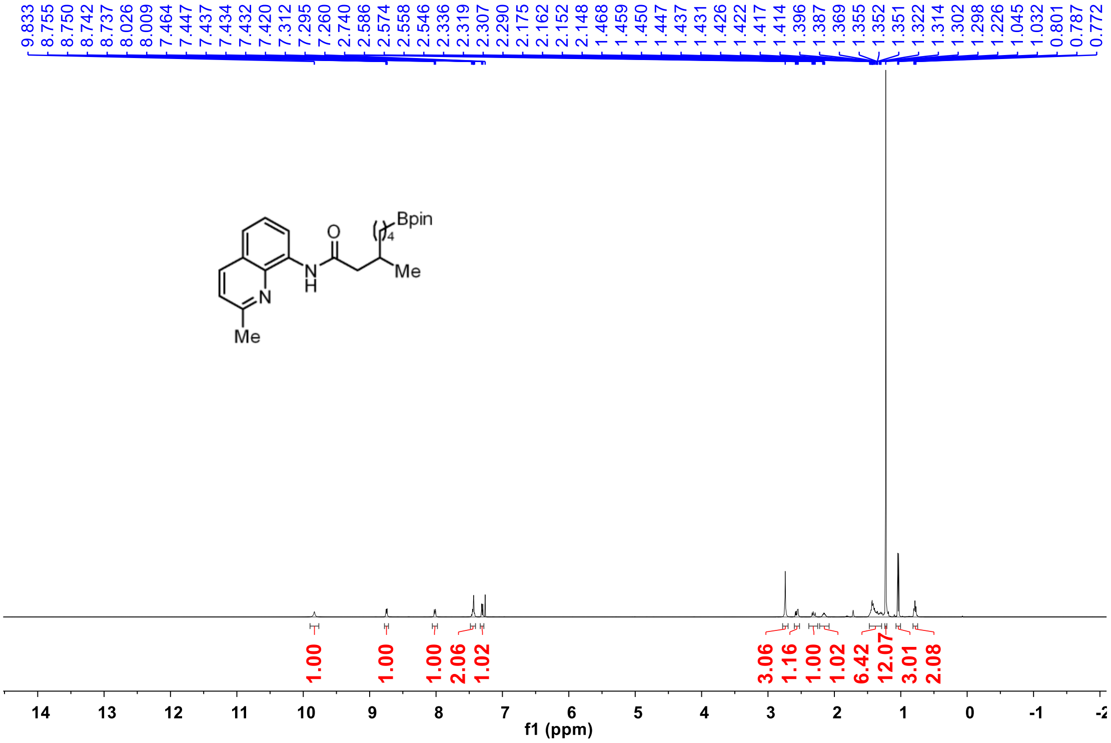

Supplementary Figure 64 ¹³C NMR (75 MHz, $CDCl_3$) of **9t:**

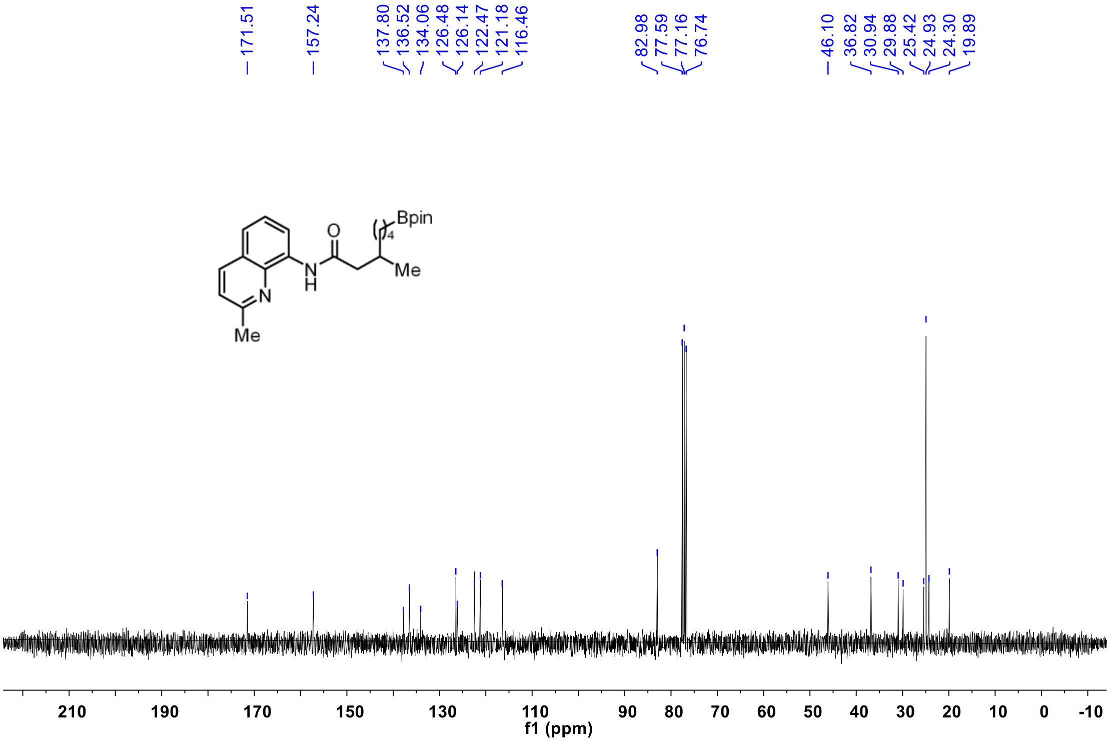

Supplementary Figure 65 ^{1}H NMR (400 MHz, $CDCl_3$) of **9u:**

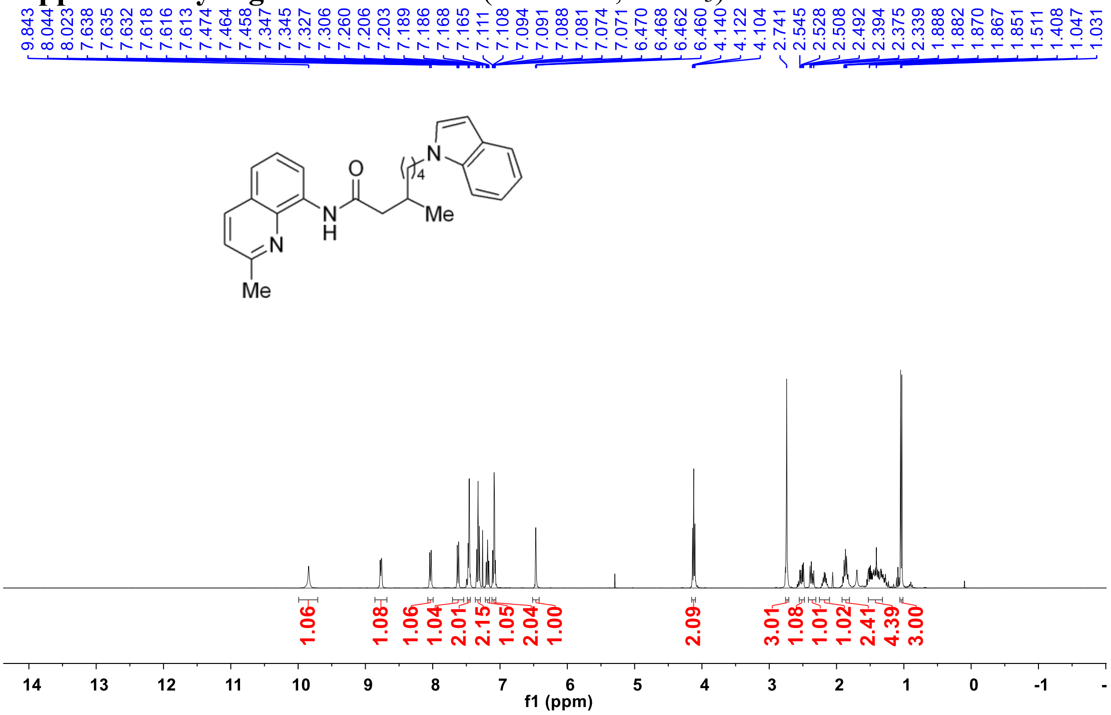

Supplementary Figure 66 ^{13}C NMR (100 MHz, $CDCl_3$) of **9u:**

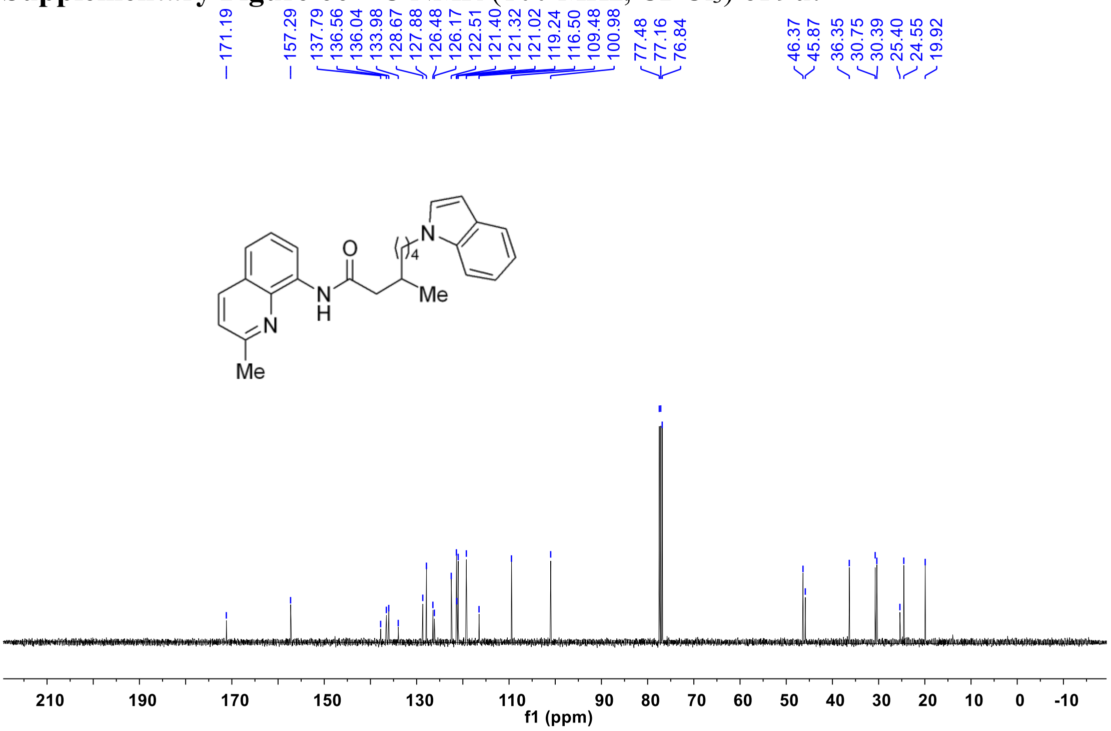

Supplementary Figure 67 ^{1}H NMR (400 MHz, $CDCl_3$) of **9v:**

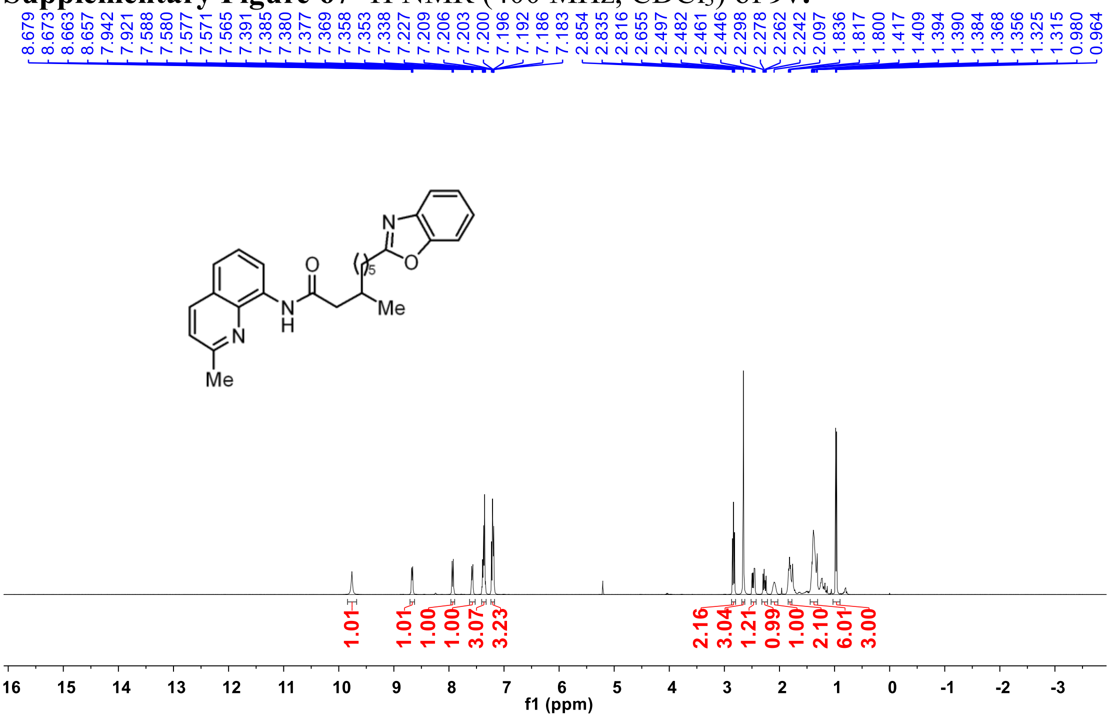

Supplementary Figure 68 ^{13}C NMR (100 MHz, $CDCl_3$) of **9v:**

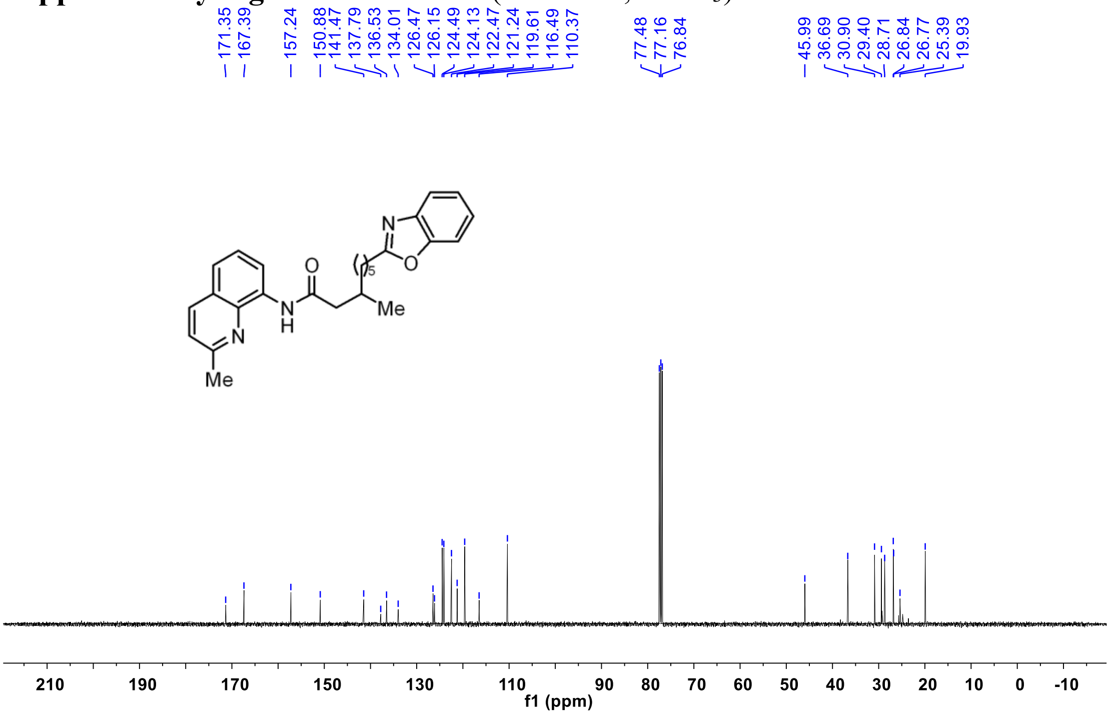

Supplementary Figure 69 ^{1}H NMR (500 MHz, $CDCl_3$) of **9x:**

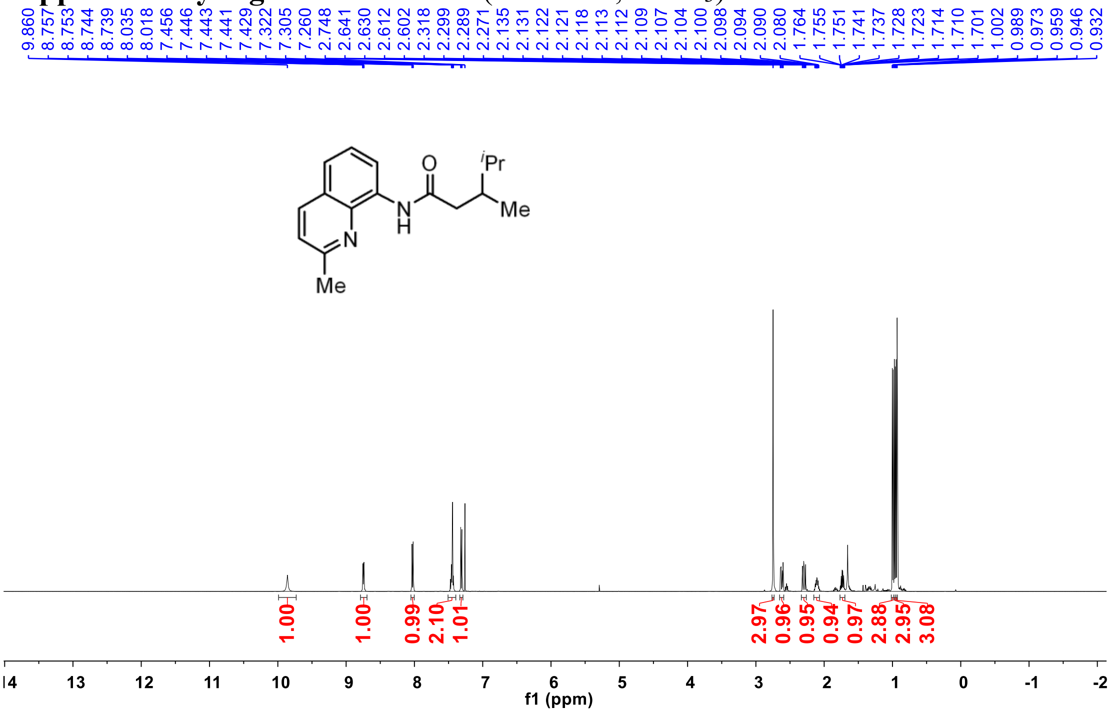

Supplementary Figure 70 ^{13}C NMR (125 MHz, $CDCl_3$) of **9x:**

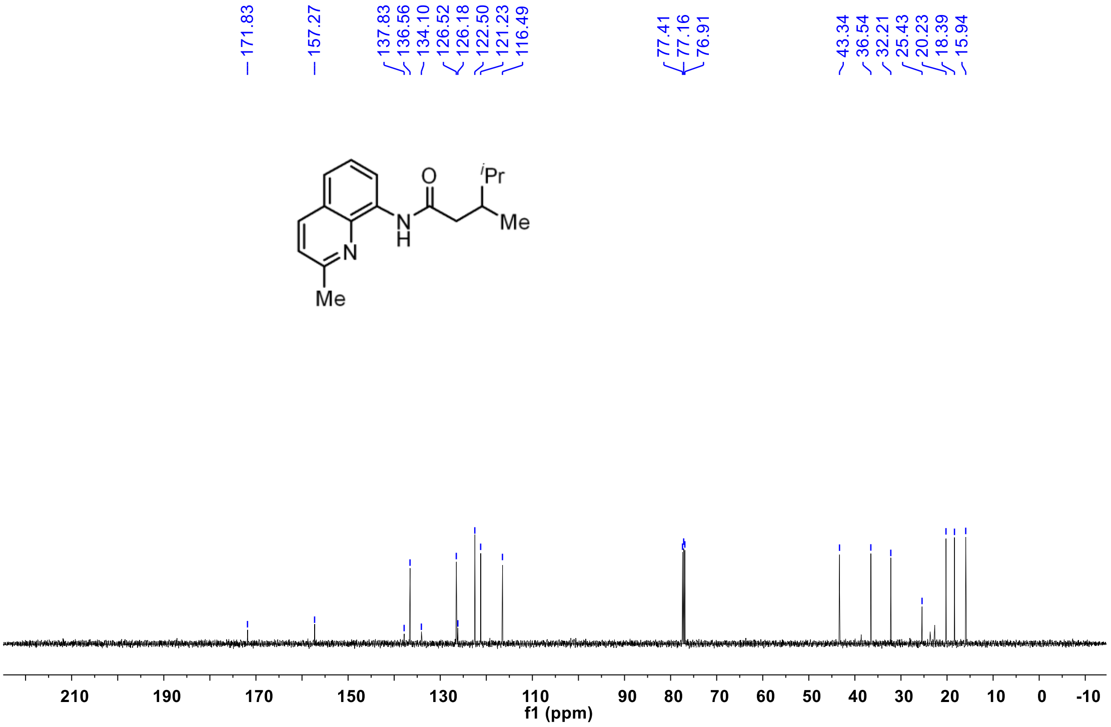

Supplementary Figure 71 ^{1}H NMR (500 MHz, $CDCl_3$) of **9y:**

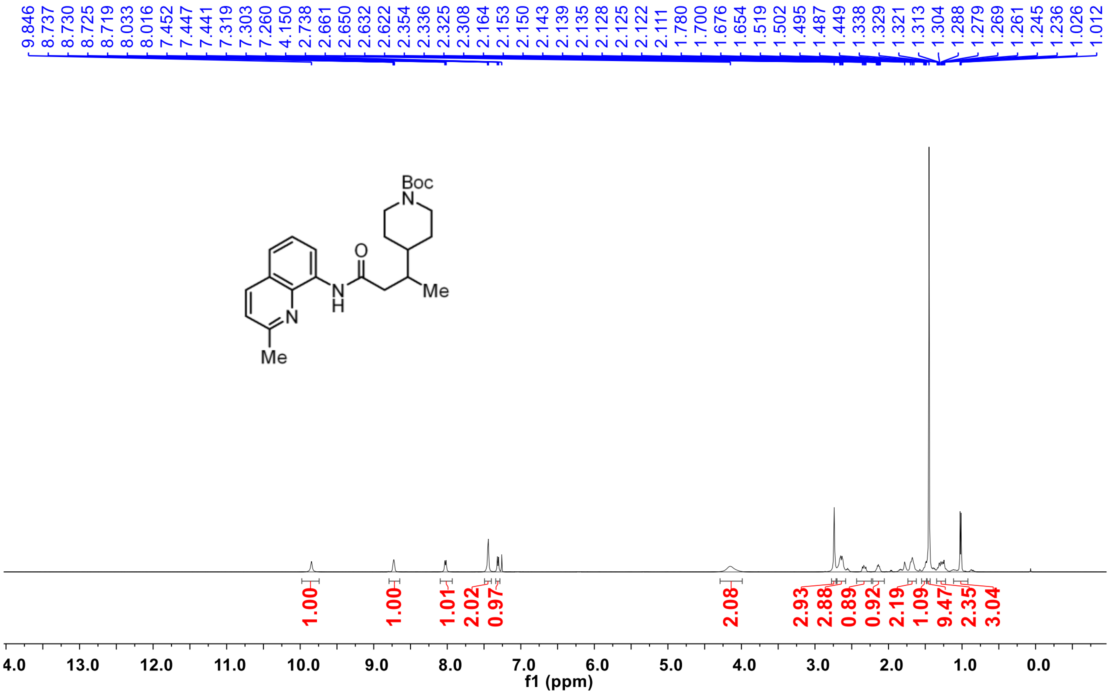

Supplementary Figure 72 ^{13}C NMR (125 MHz, $CDCl_3$) of **9y:**

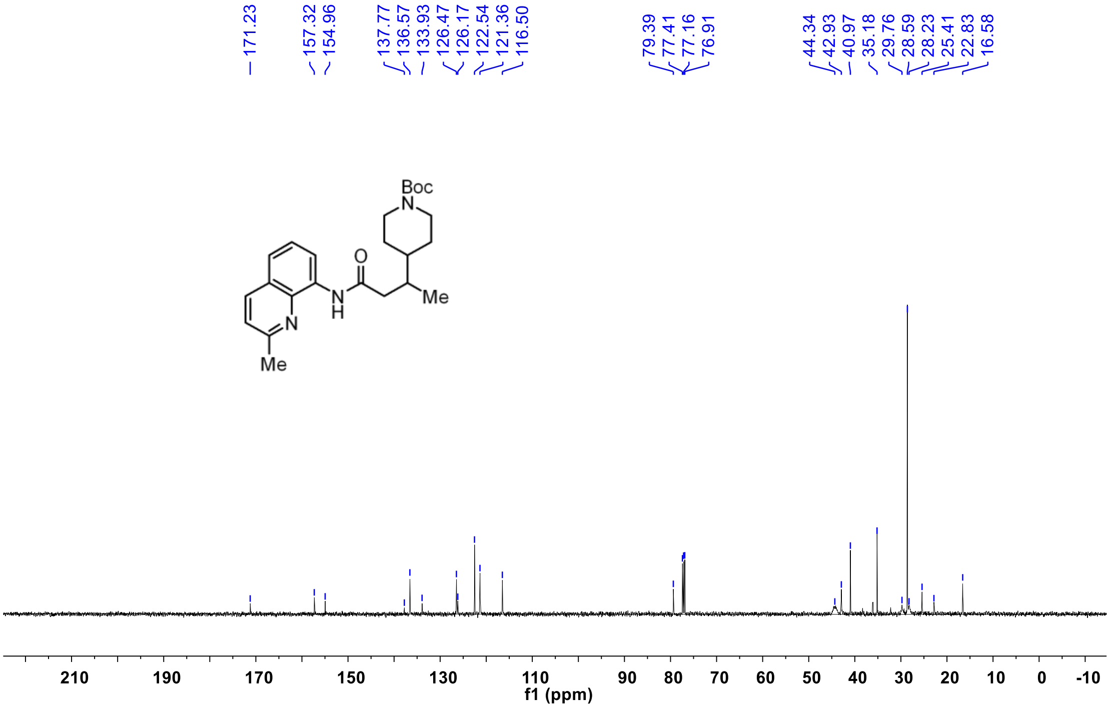

Supplementary Figure 73 ^{1}H NMR (500 MHz, $CDCl_3$) of **9z:**

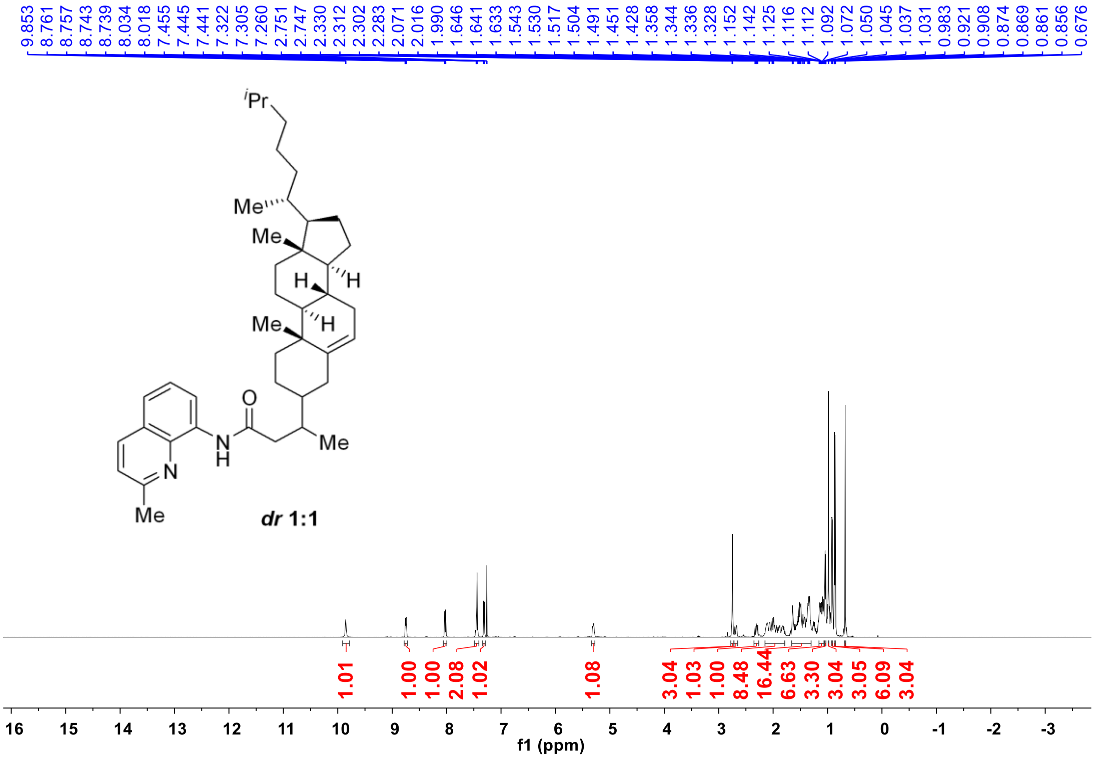

Supplementary Figure 74 ^{13}C NMR (125 MHz, $CDCl_3$) of **9z:**

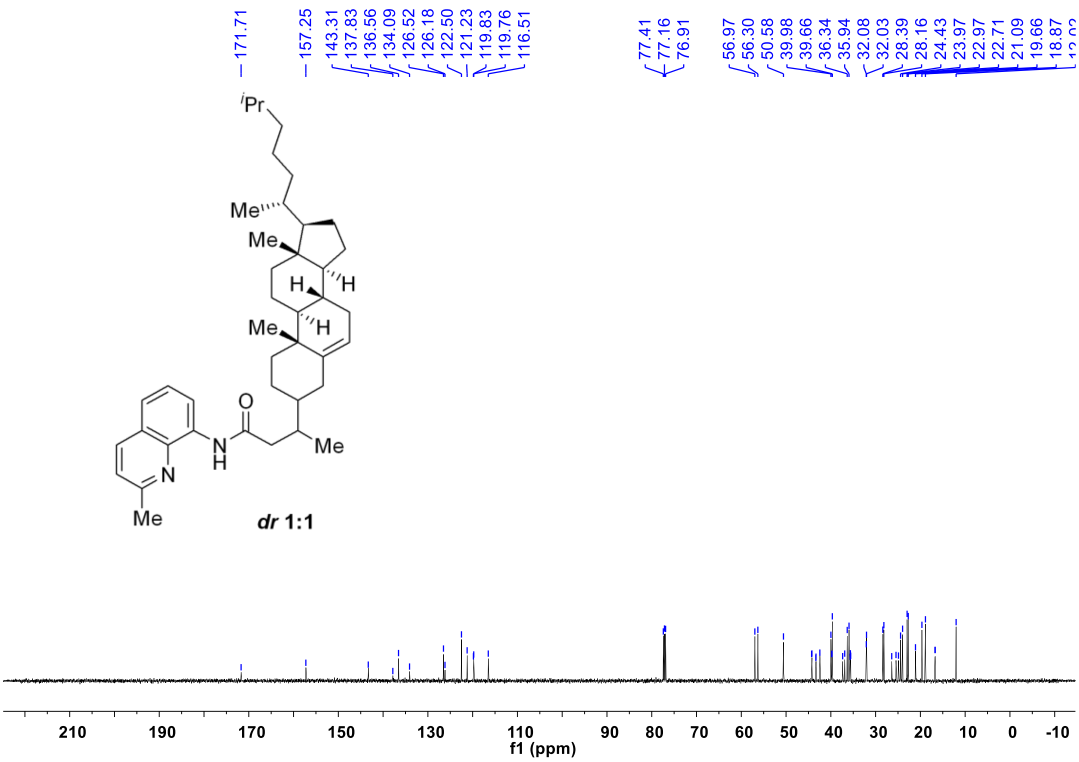

Supplementary Figure 75 ^{1}H NMR (400 MHz, $CDCl_3$) of **9aa:**

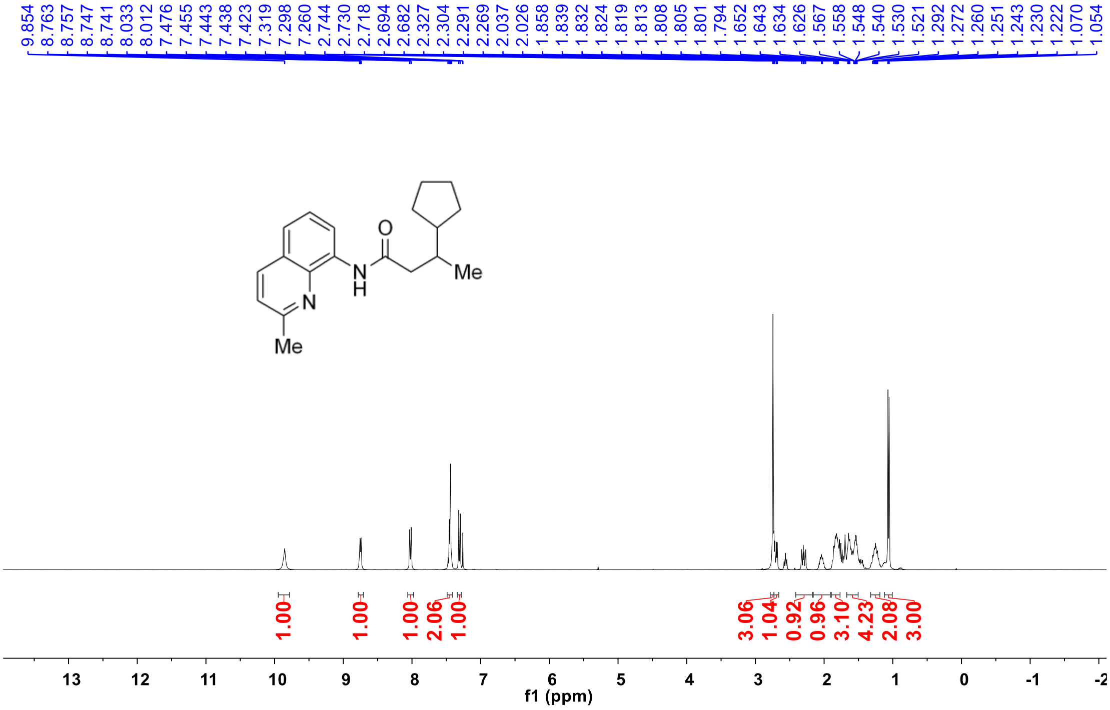

Supplementary Figure 76 ^{13}C NMR (100 MHz, $CDCl_3$) of **9aa:**

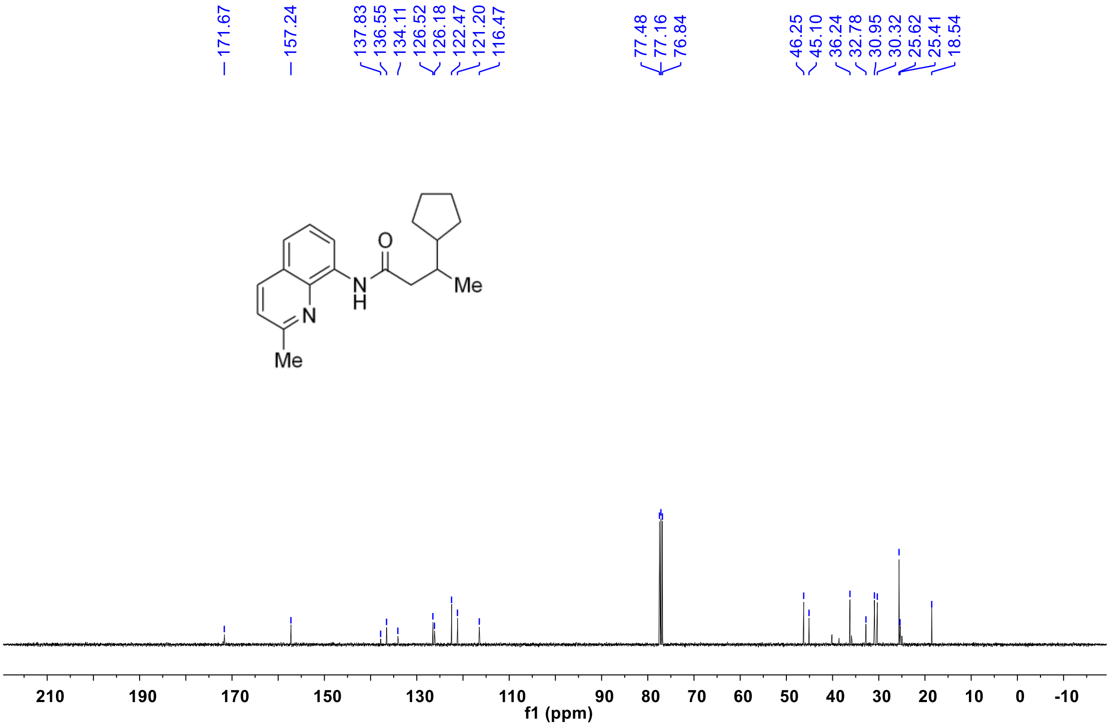

Supplementary Figure 77 ^{1}H NMR (300 MHz, $CDCl_3$) of **9ab:**

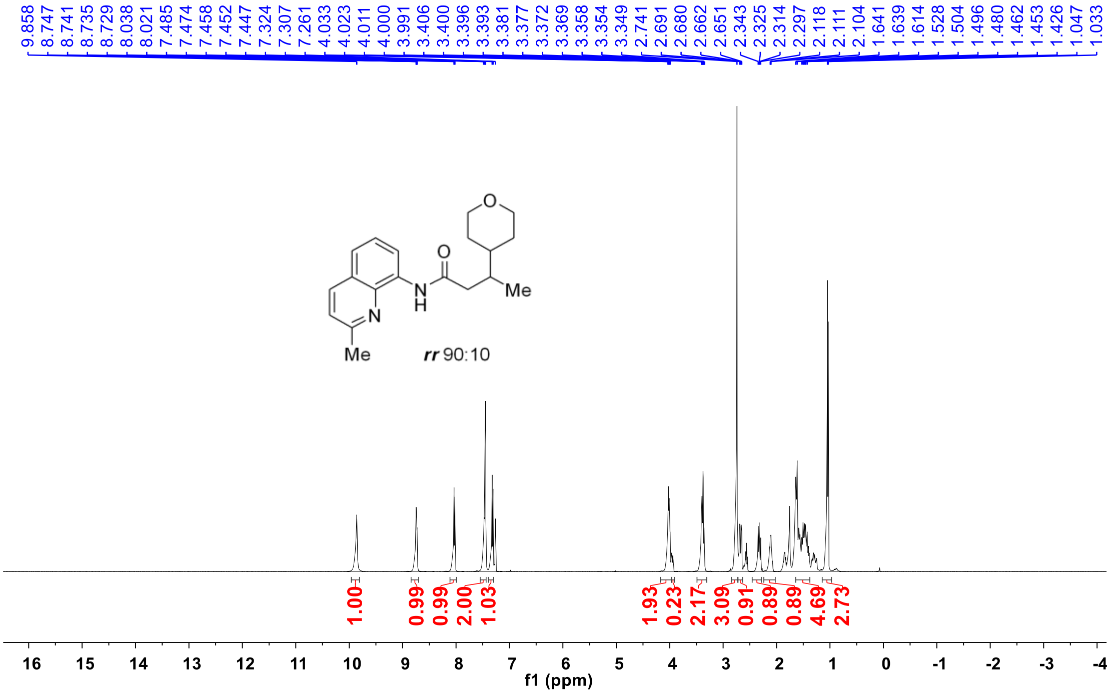

Supplementary Figure 78 ^{13}C NMR (75 MHz, $CDCl_3$) of **9ab:**

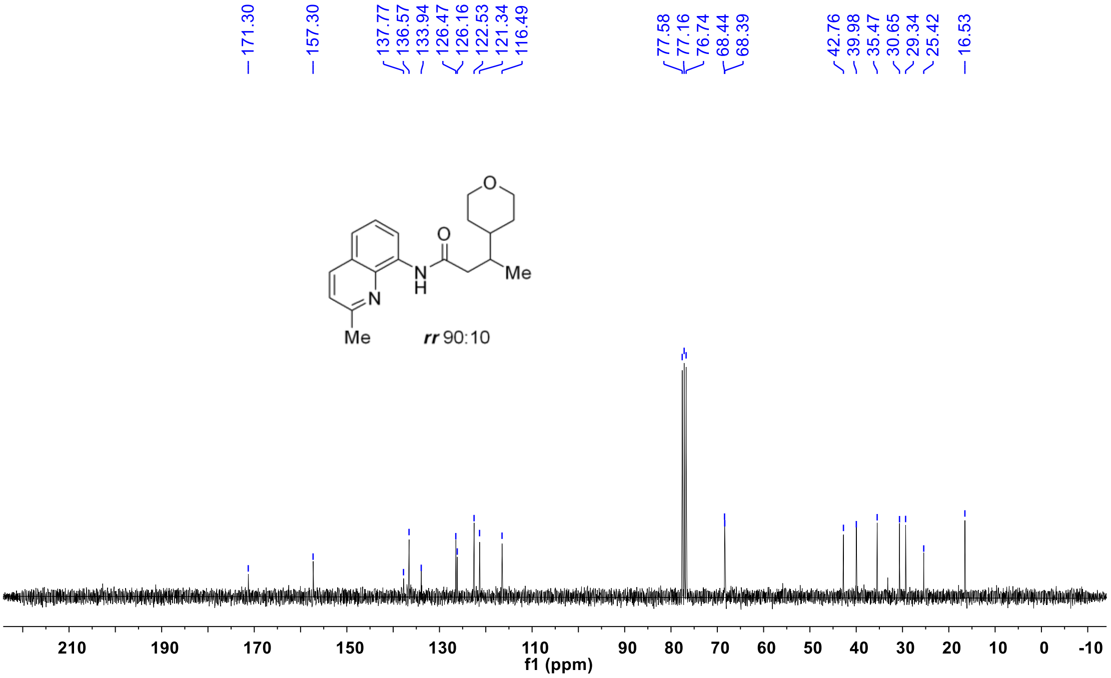

Supplementary Figure 79 ^{1}H NMR (500 MHz, $CDCl_3$)of **9ac:**

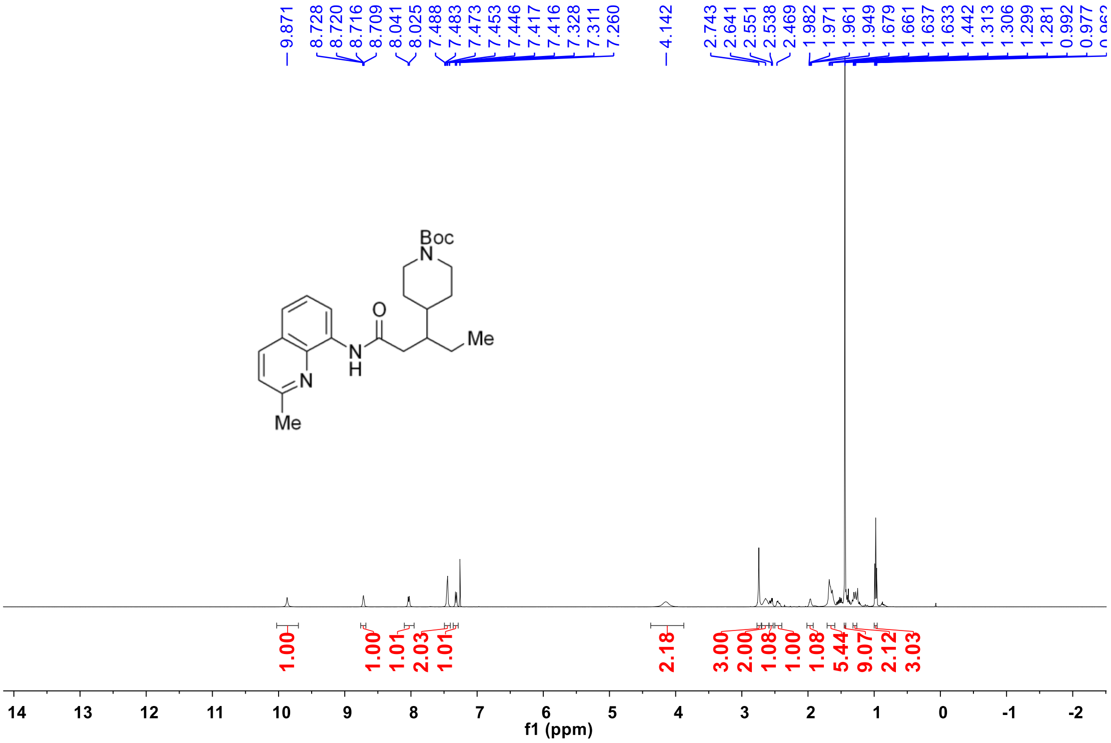

Supplementary Figure 80 ^{13}C NMR (125 MHz, $CDCl_3$) of **9ac:**

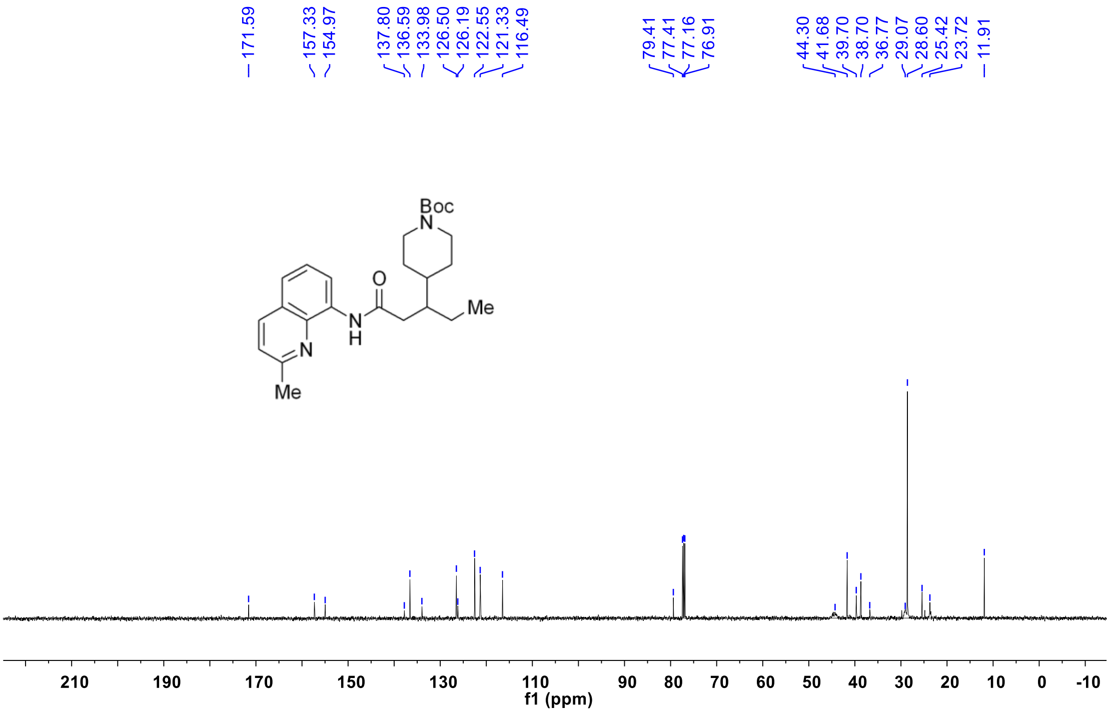

Supplementary Figure 81 ^{1}H NMR (500 MHz, $CDCl_3$) of **9ad:**

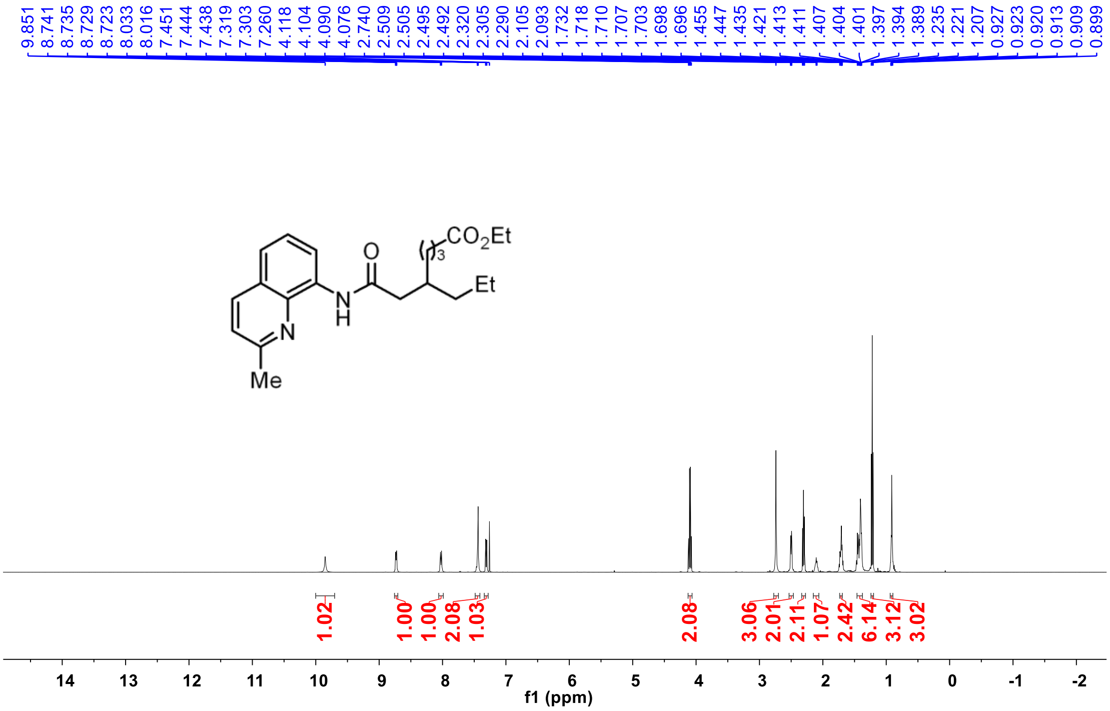

Supplementary Figure 82 ^{13}C NMR (125 MHz, $CDCl_3$) of **9ad:**

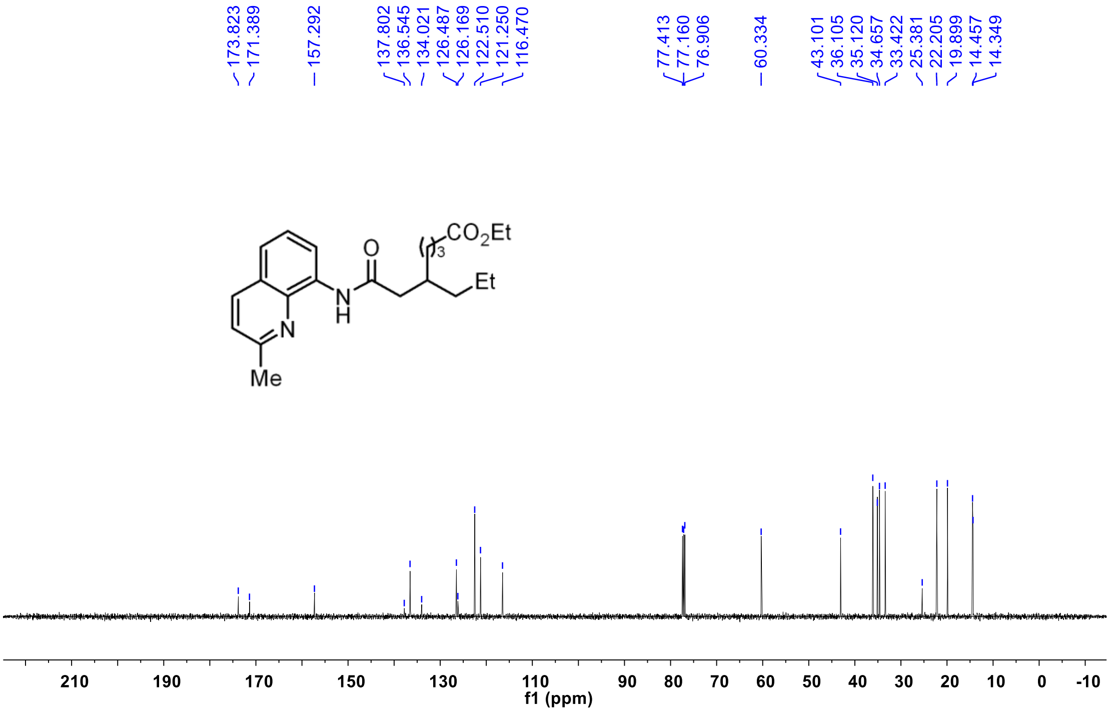

Supplementary Figure 83 ^{1}H NMR (500 MHz, $CDCl_3$) of **9ae:**

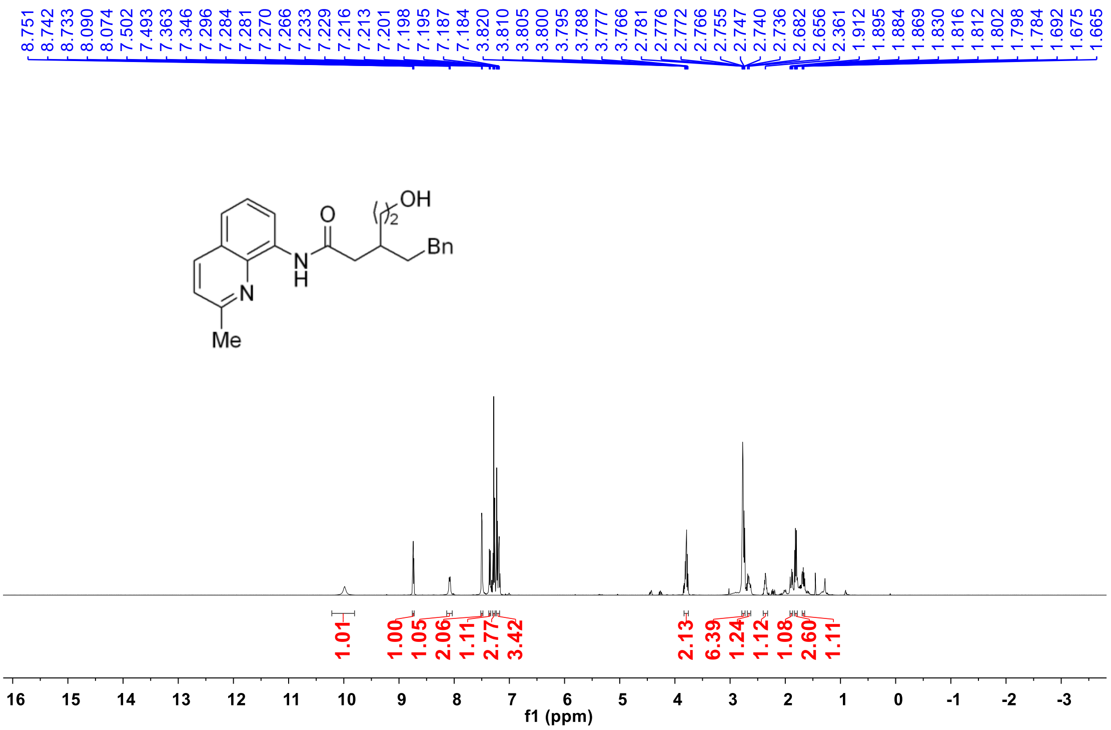

Supplementary Figure 84 ^{13}C NMR (125 MHz, $CDCl_3$) of **9ae:**

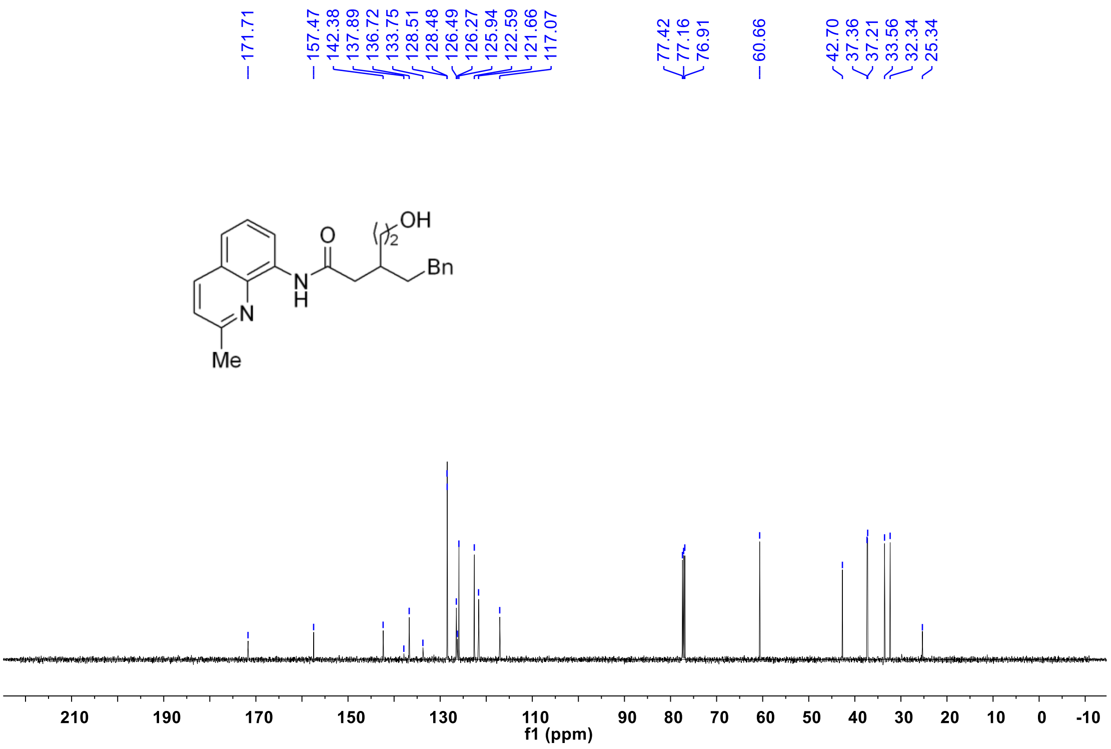

Supplementary Figure 85 ^{1}H NMR (400 MHz, $CDCl_3$) of **9af:**

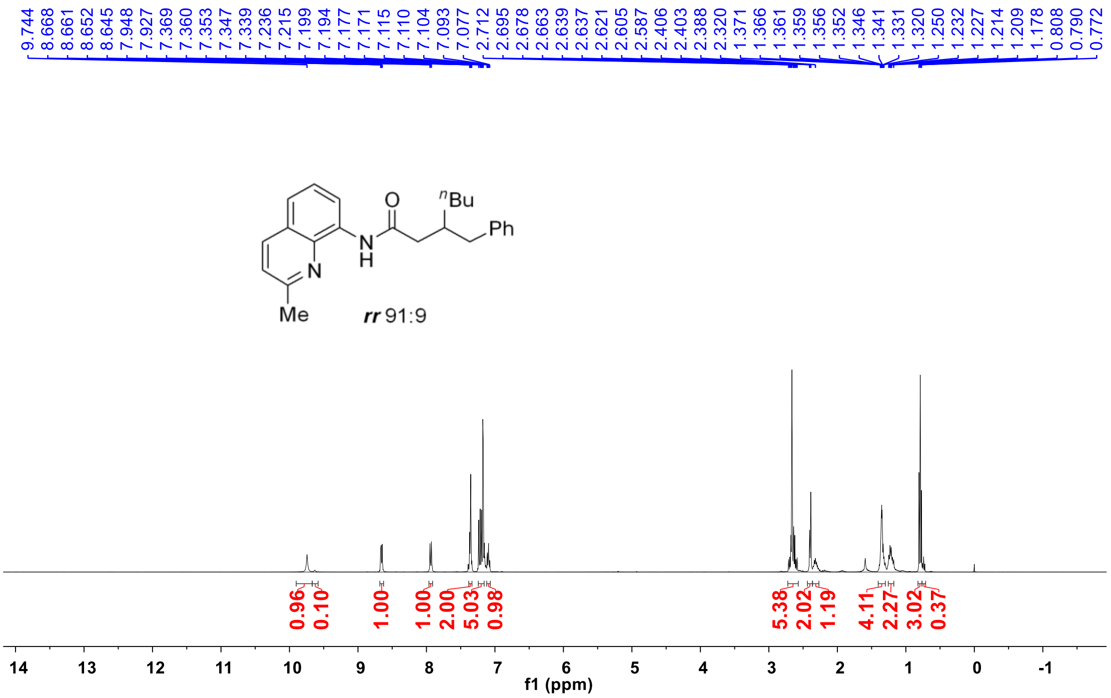

Supplementary Figure 86 ^{13}C NMR (75 MHz, $CDCl_3$) of **9af:**

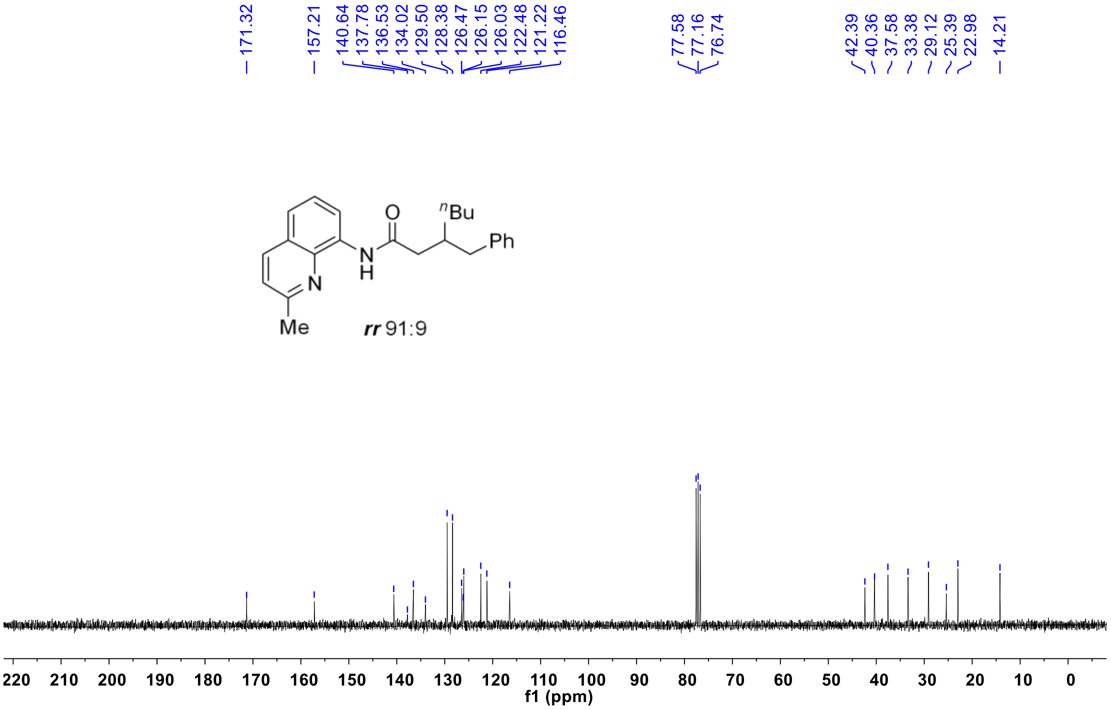

Supplementary Figure 87 ^{1}H NMR (500 MHz, $CDCl_3$) of **9ag:**

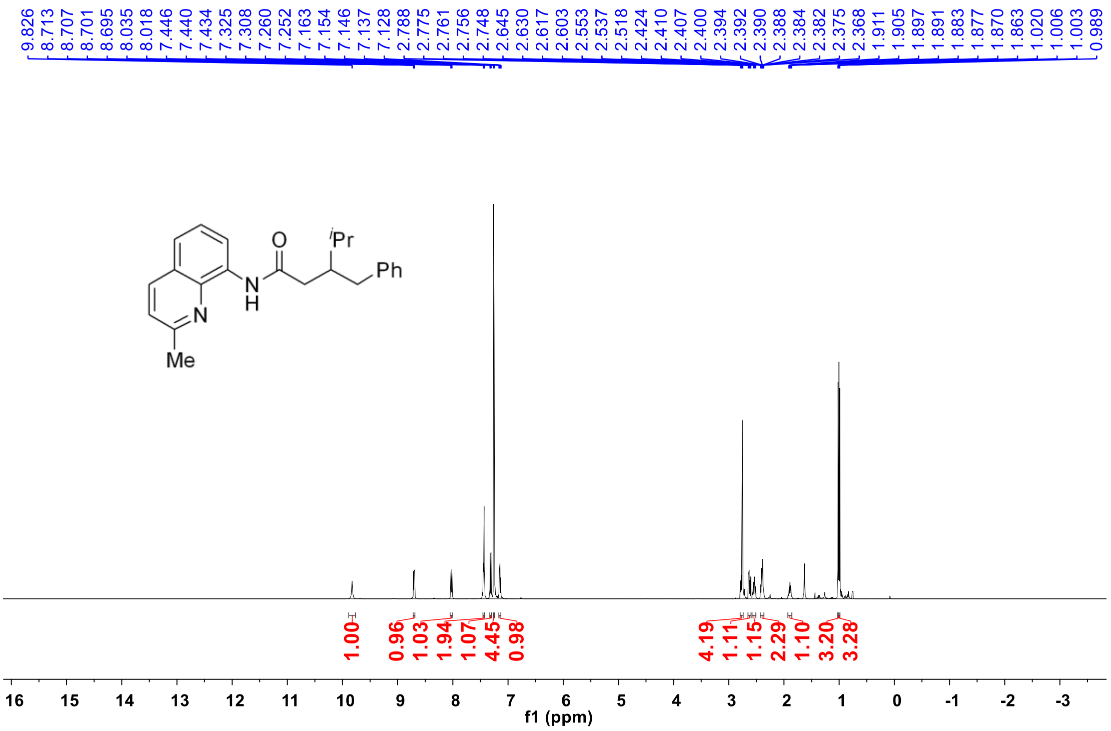

Supplementary Figure 88 ^{13}C NMR (125 MHz, $CDCl_3$) of **9ag:**

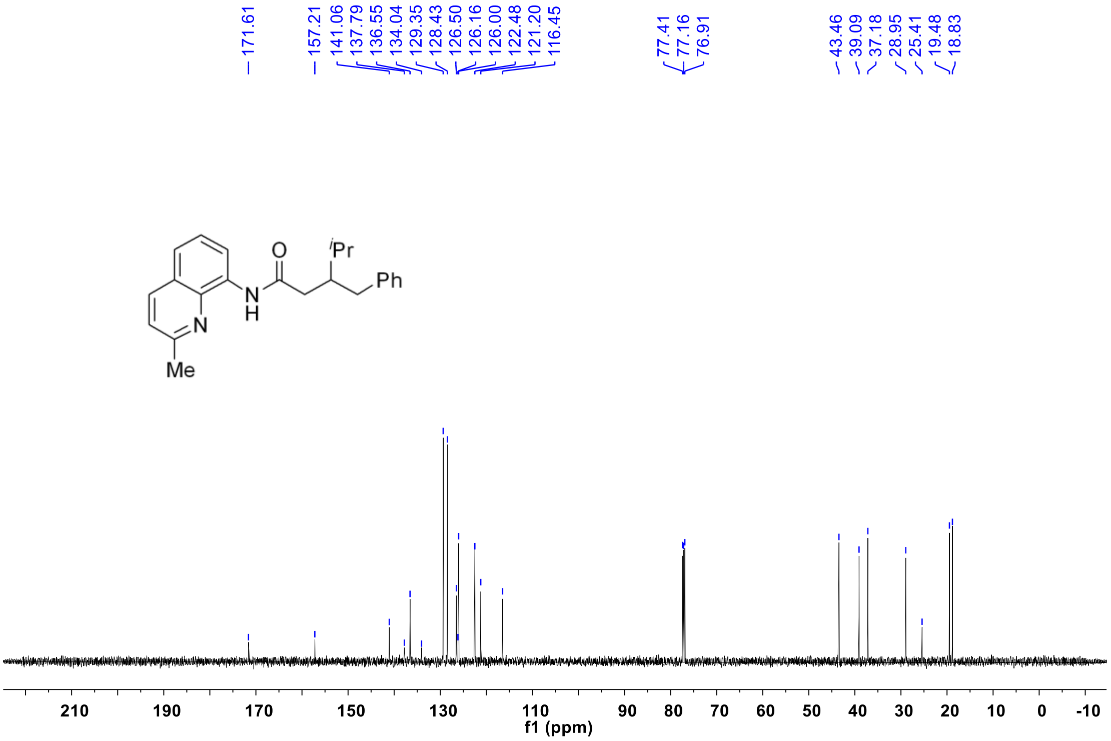

Supplementary Figure 89 ^{1}H NMR (300 MHz, $CDCl_3$) of **9ah:**

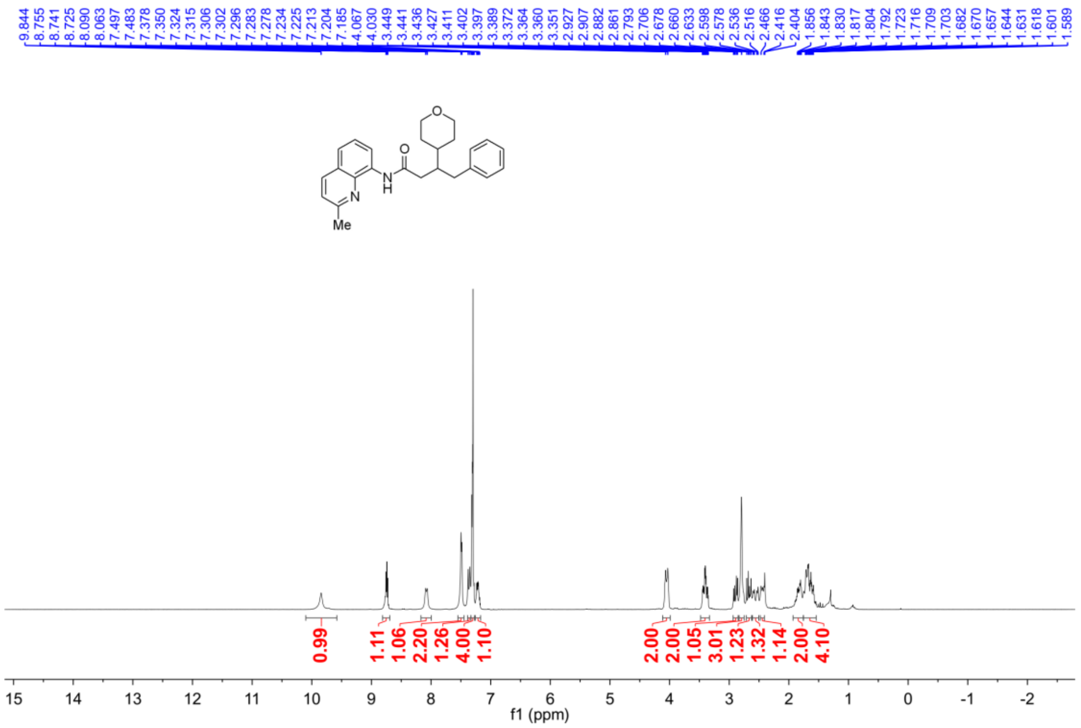

Supplementary Figure 90 ^{13}C NMR (126 MHz, $CDCl_3$) of **9ah:**

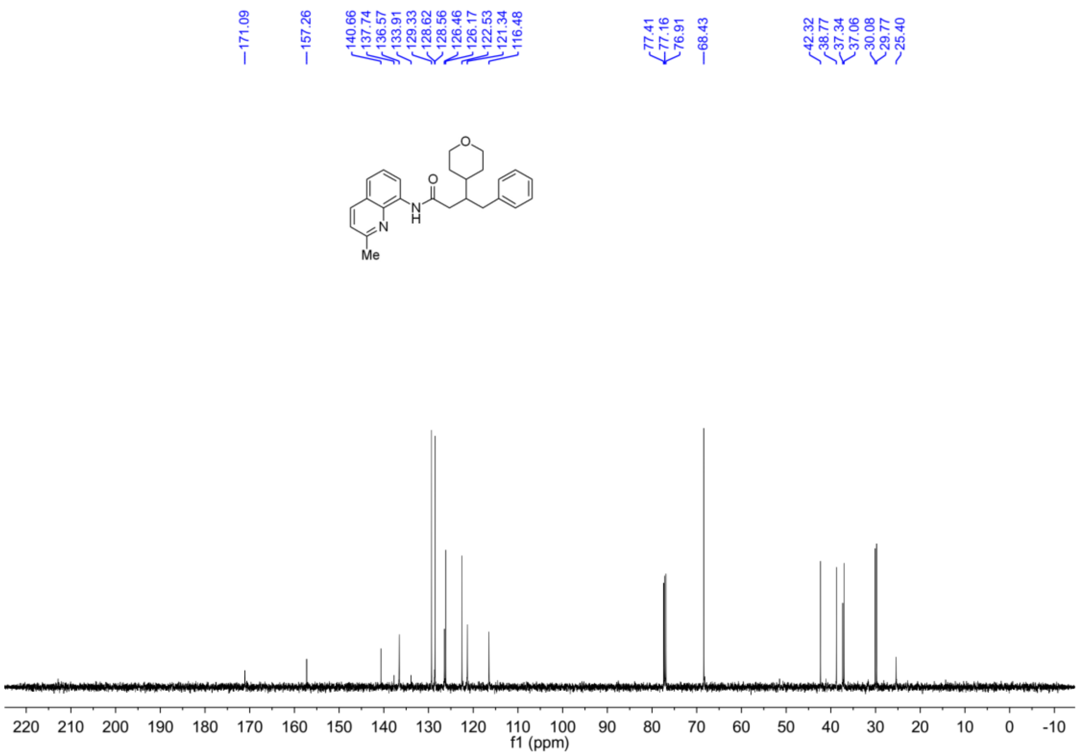

Supplementary Figure 91 ^{1}H NMR (400 MHz, $CDCl_3$) of **14:**

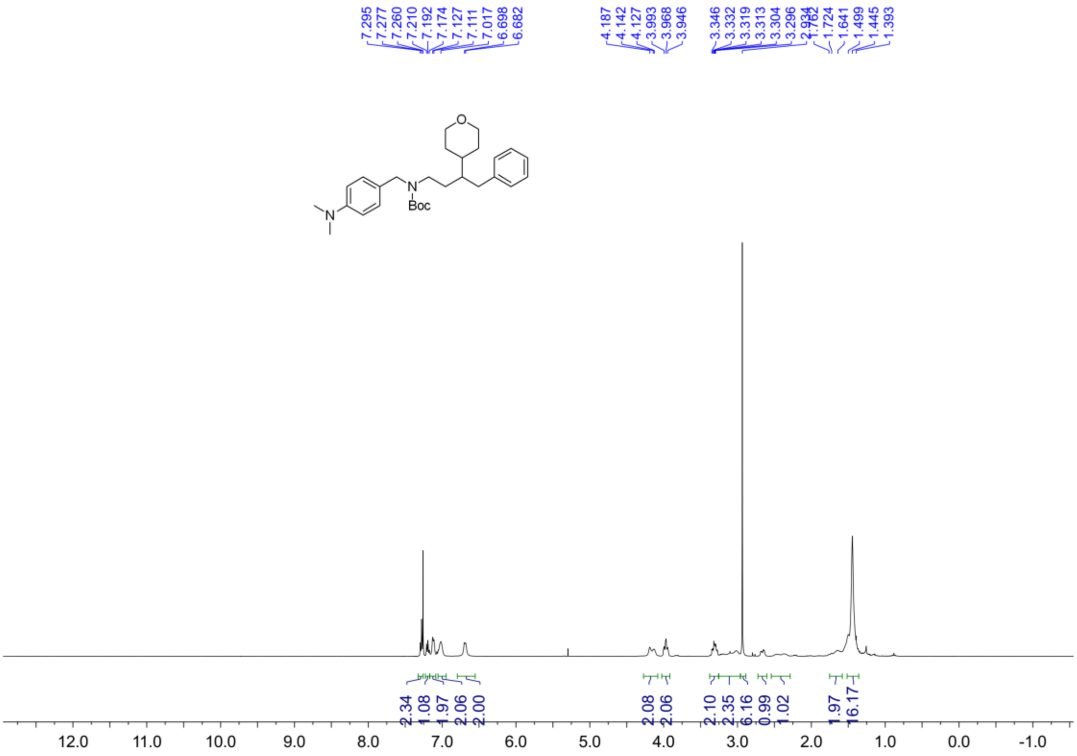

Supplementary Figure 92 ^{13}C NMR (126 MHz, $CDCl_3$) of **14:**

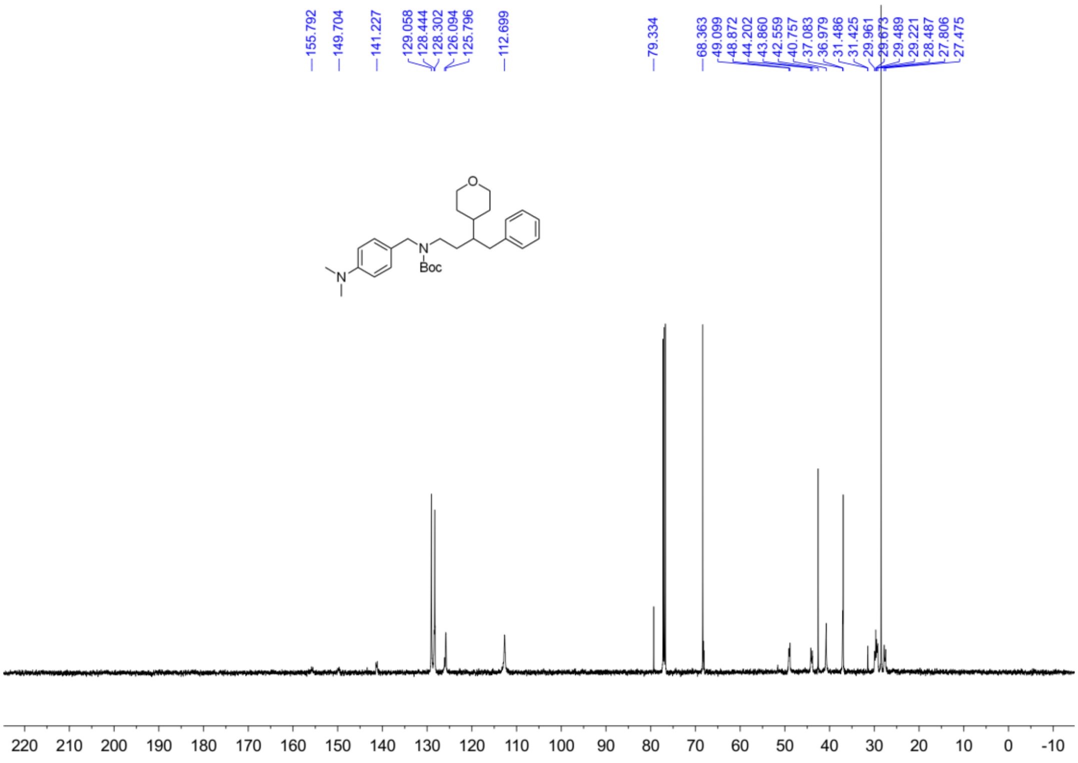

Supplementary Figure 93 ^{1}H NMR (500 MHz, $CDCl_3$) of **9ai:**

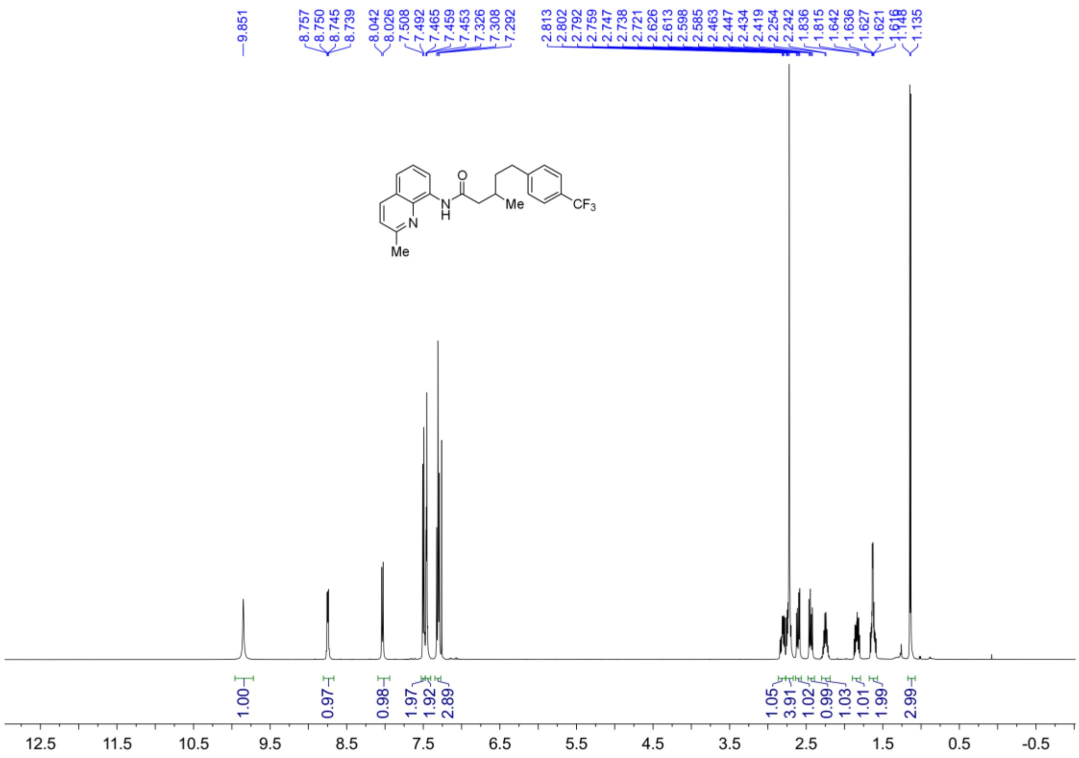

Supplementary Figure 94 ^{13}C NMR (126 MHz, $CDCl_3$) of **9ai:**

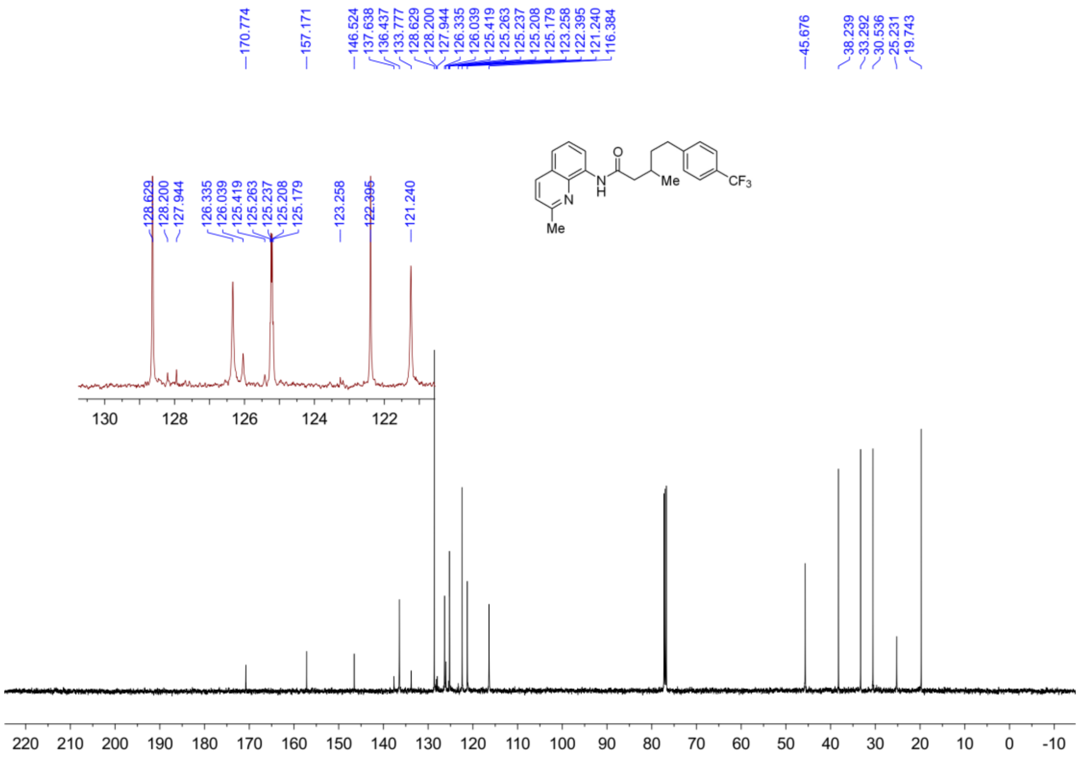

Supplementary Figure 95 ^{19}F NMR (471 MHz, $CDCl_3$) of **9ai:**

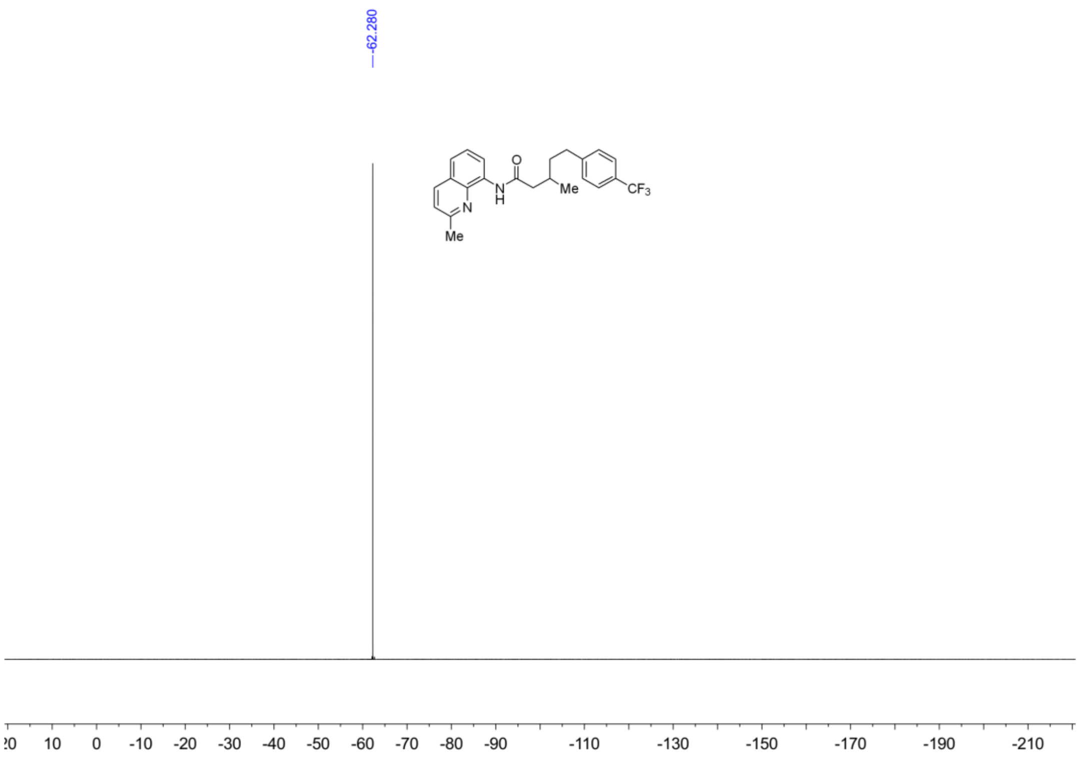

Supplementary Figure 96 ^{1}H NMR (400 MHz, $CDCl_3$) of **15:**

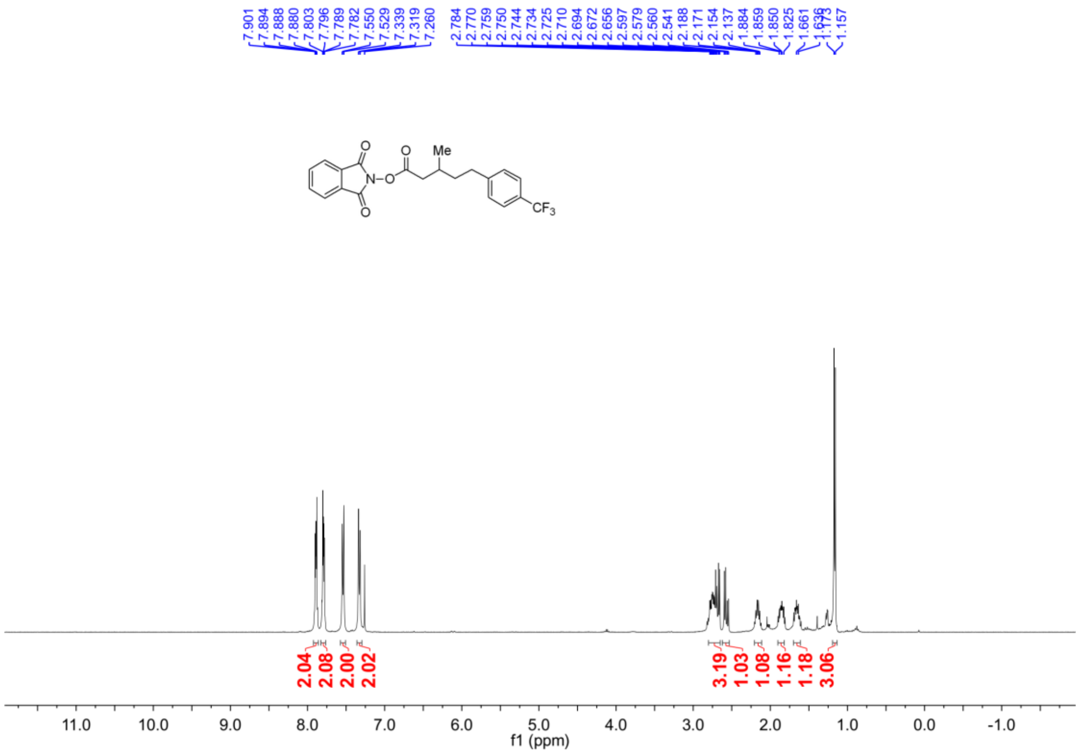

Supplementary Figure 97 ^{13}C NMR (101 MHz, $CDCl_3$) of **15:**

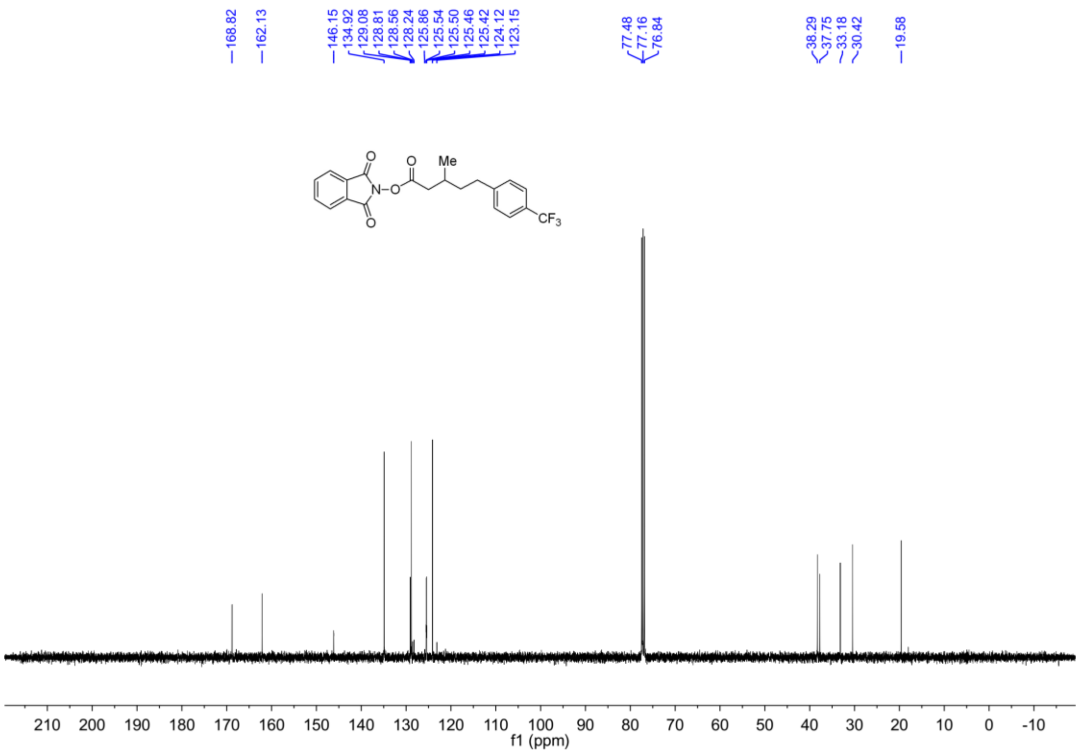

Supplementary Figure 98 ^{19}F NMR (377 MHz, $CDCl_3$) of **15:**

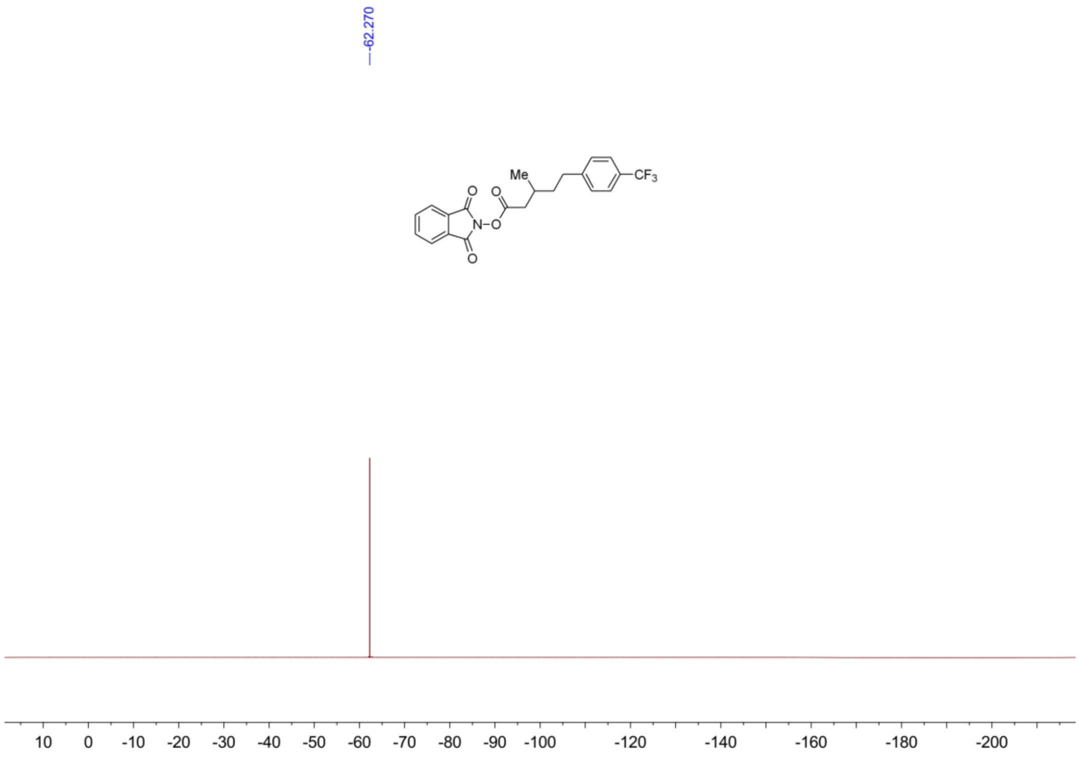

Supplementary Figure 98 ^{1}H NMR (500 MHz, $CDCl_3$) of **16:**

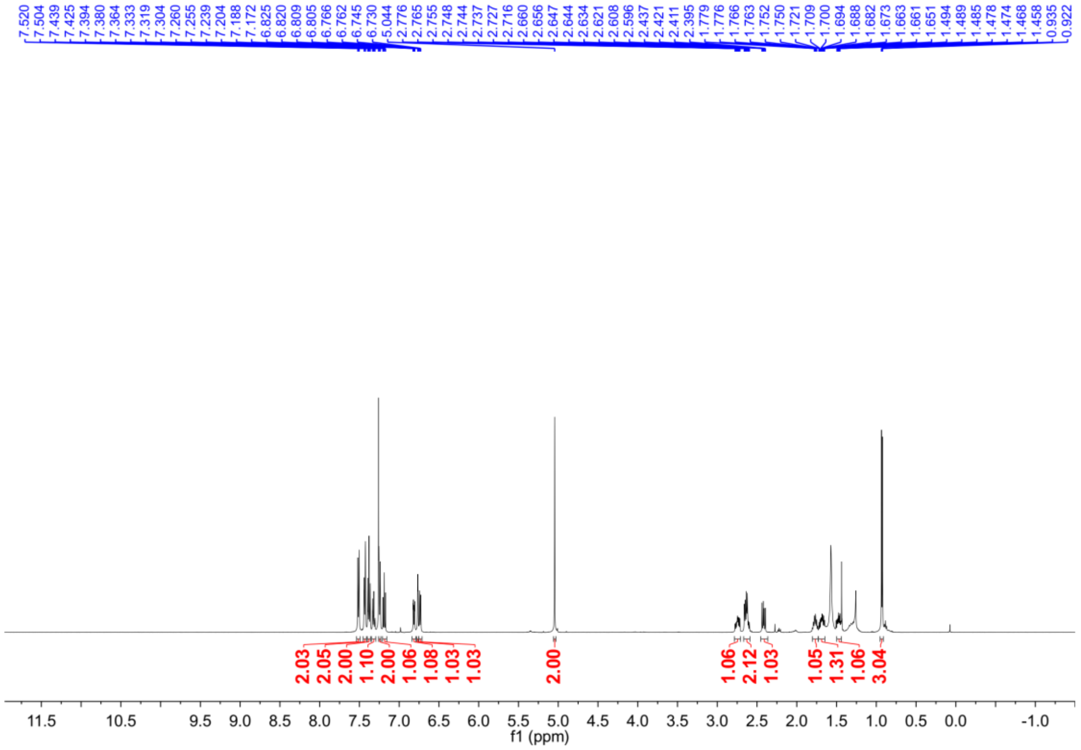

Supplementary Figure 99 ^{13}C NMR (125 MHz, $CDCl_3$) of **16:**

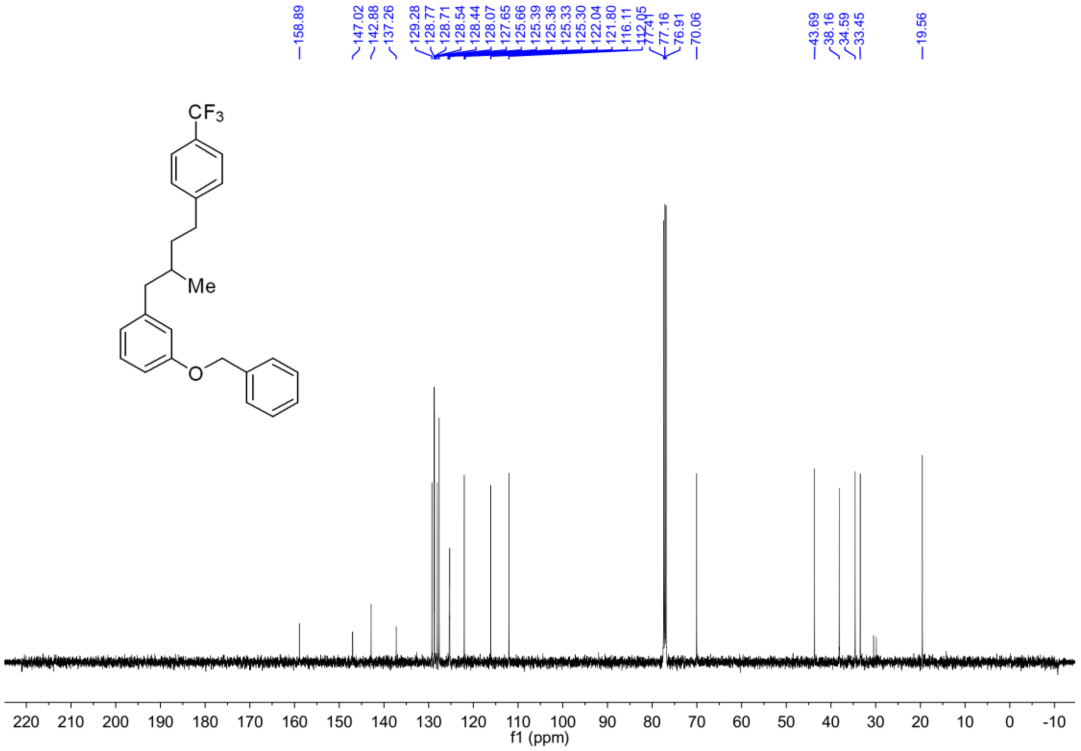

Supplementary Figure 100 ^{19}F NMR (471 MHz, $CDCl_3$) of **16:**

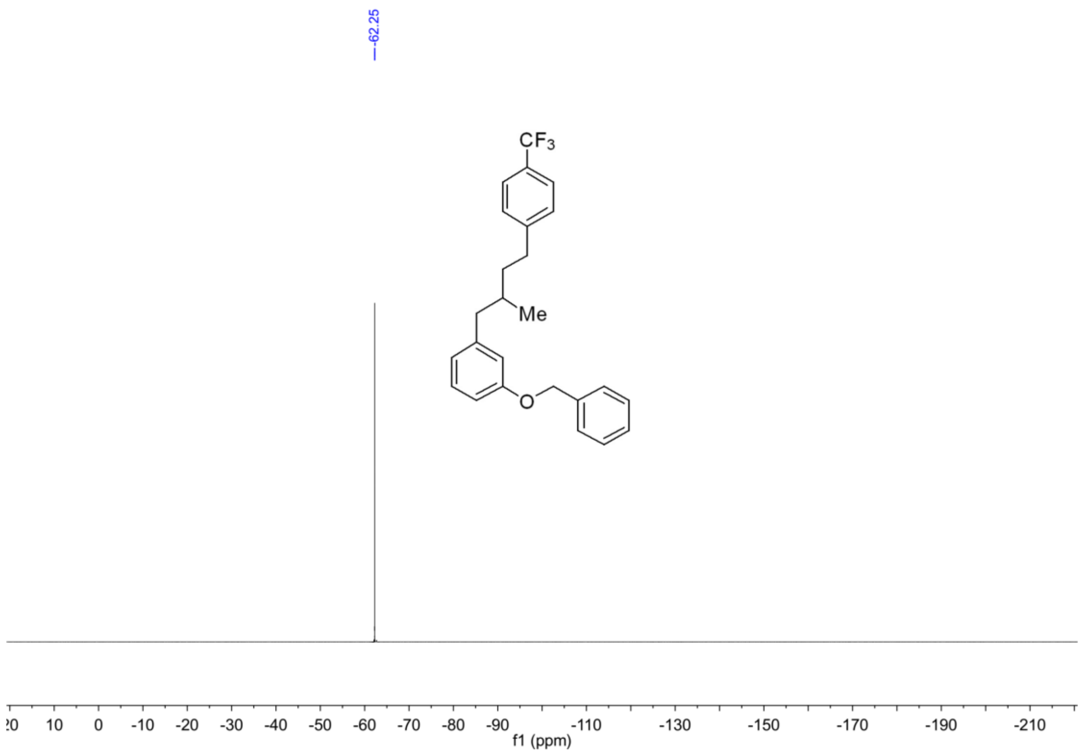

Supplementary Figure 101 ^{1}H NMR (400 MHz, $CDCl_3$) of **18a:**

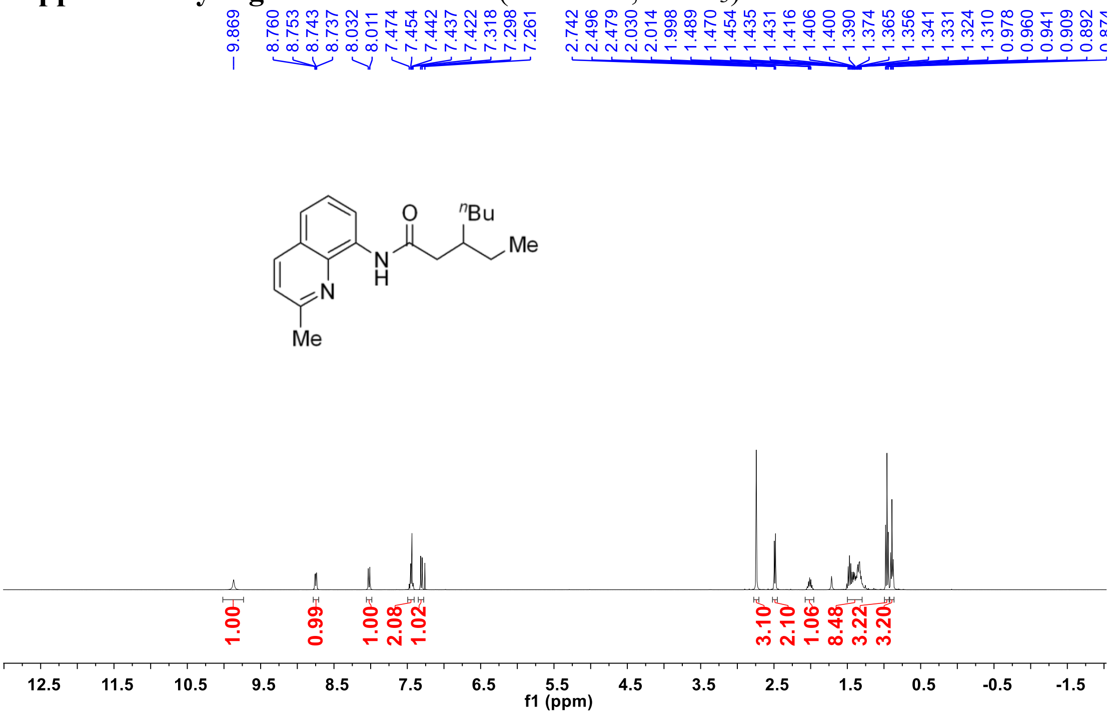

Supplementary Figure 102 ^{13}C NMR (100 MHz, $CDCl_3$) of **18a:**

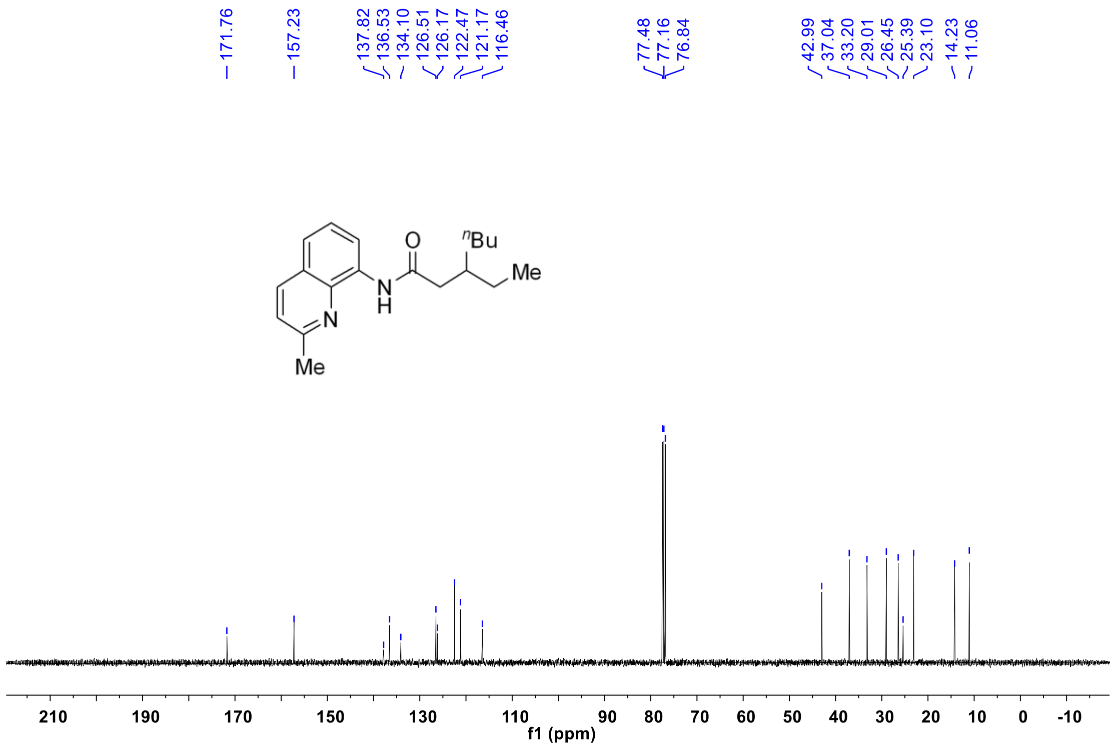

Supplementary Figure 103 DEPT135(100 MHz, $CDCl_3$) of **18a:**

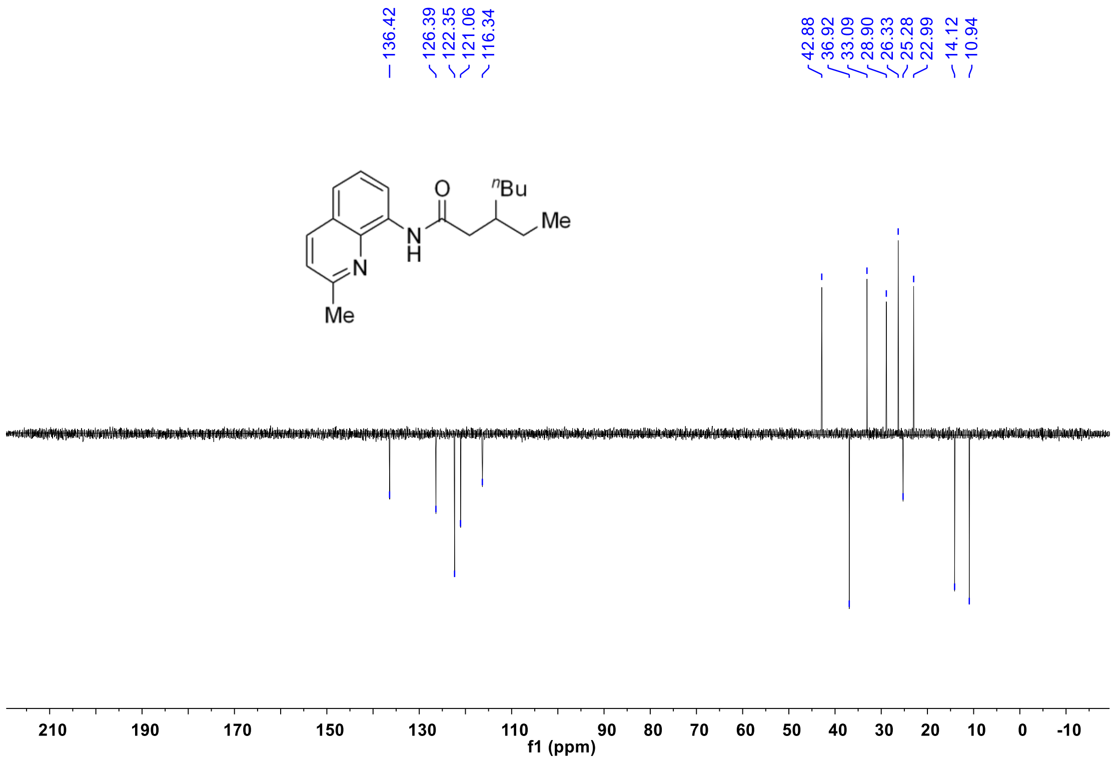

Supplementary Figure 104 ^{1}H NMR (500 MHz, DMSO-d_6) of **18b:**

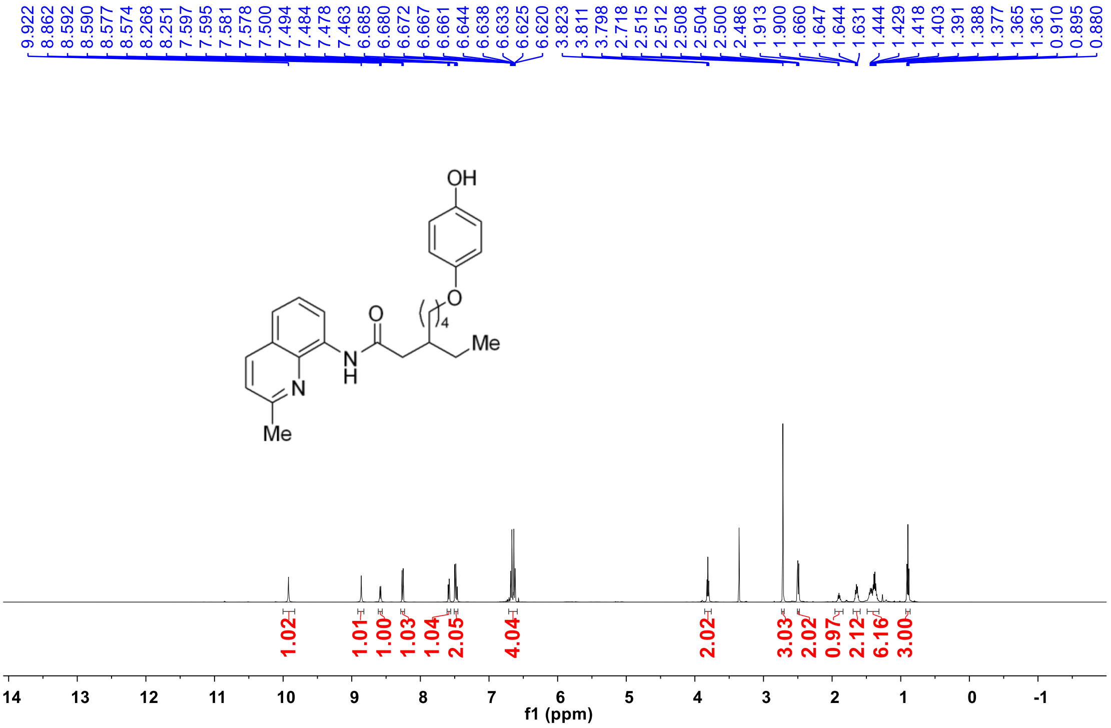

Supplementary Figure 105 ^{13}C NMR (125 MHz, DMSO-d_6) of **18b:**

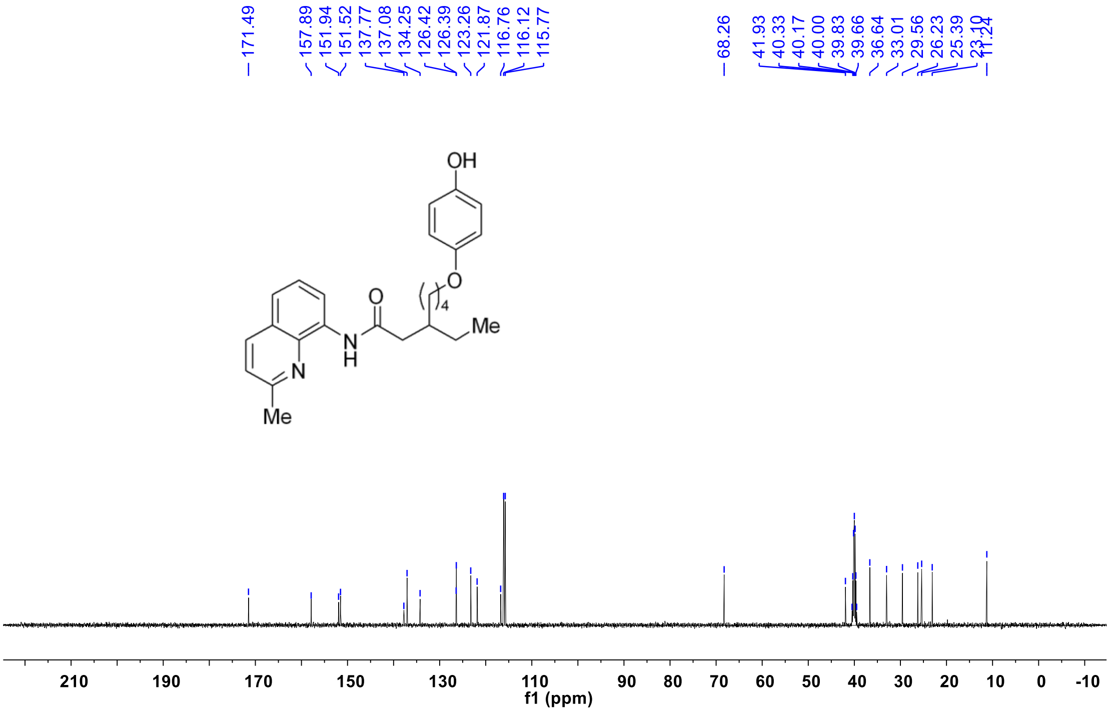

Supplementary Figure 106 ^{1}H NMR (500 MHz, $CDCl_3$) of **18c:**

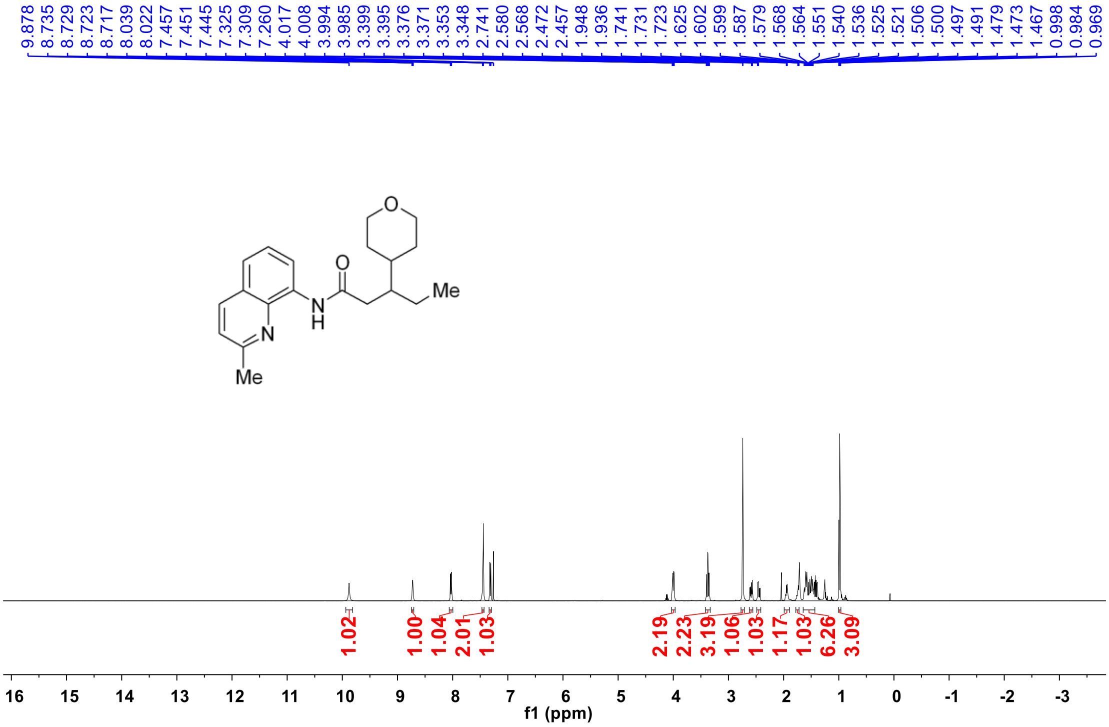

Supplementary Figure 107 ^{13}C NMR (125 MHz, $CDCl_3$) of **18c:**

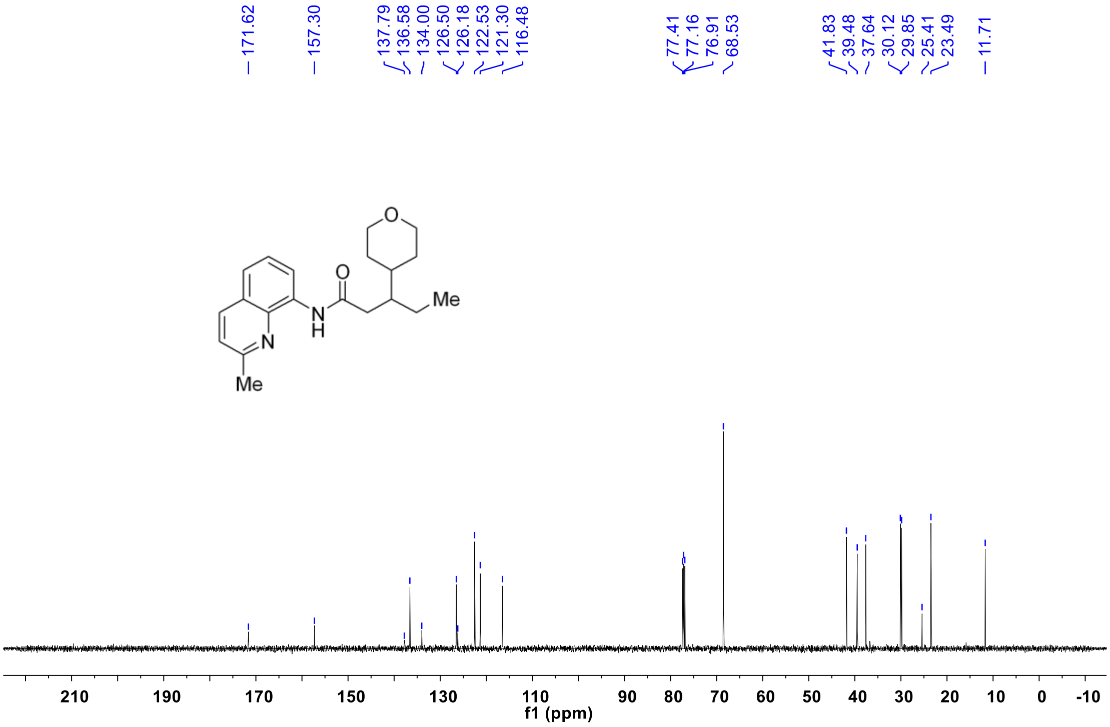

Supplementary Figure 108 ^{1}H NMR (500 MHz, $CDCl_3$) of **18d:**

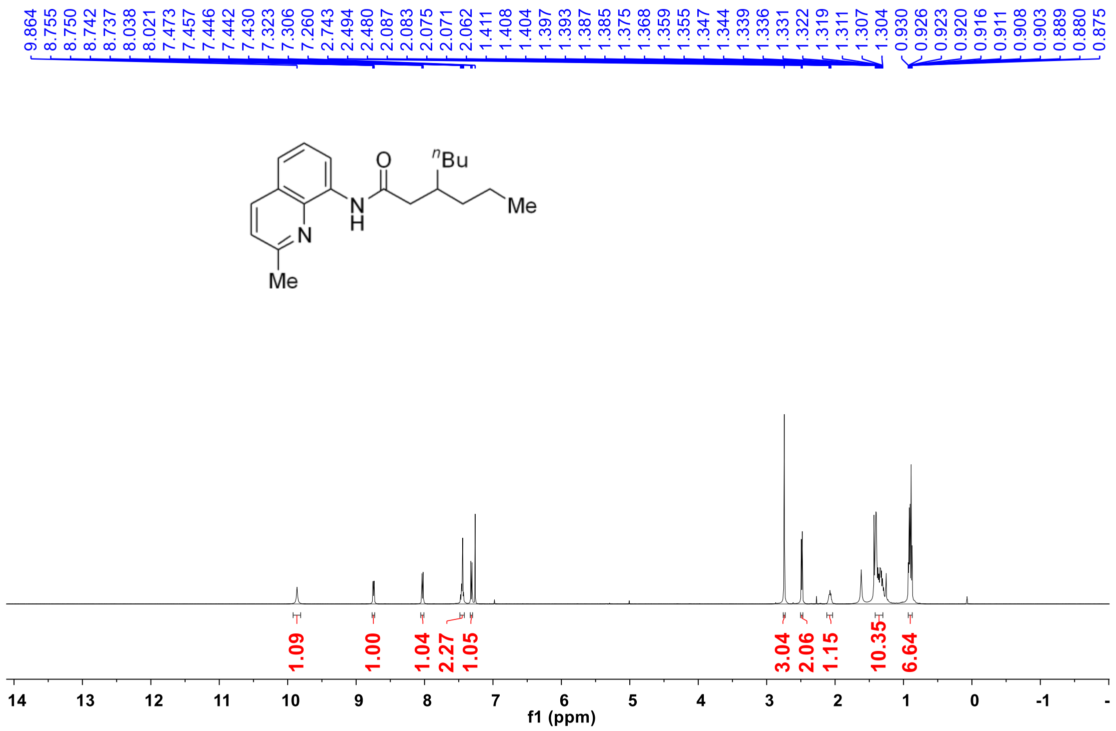

Supplementary Figure 109 ^{13}C NMR (100 MHz, $CDCl_3$) of **18d:**

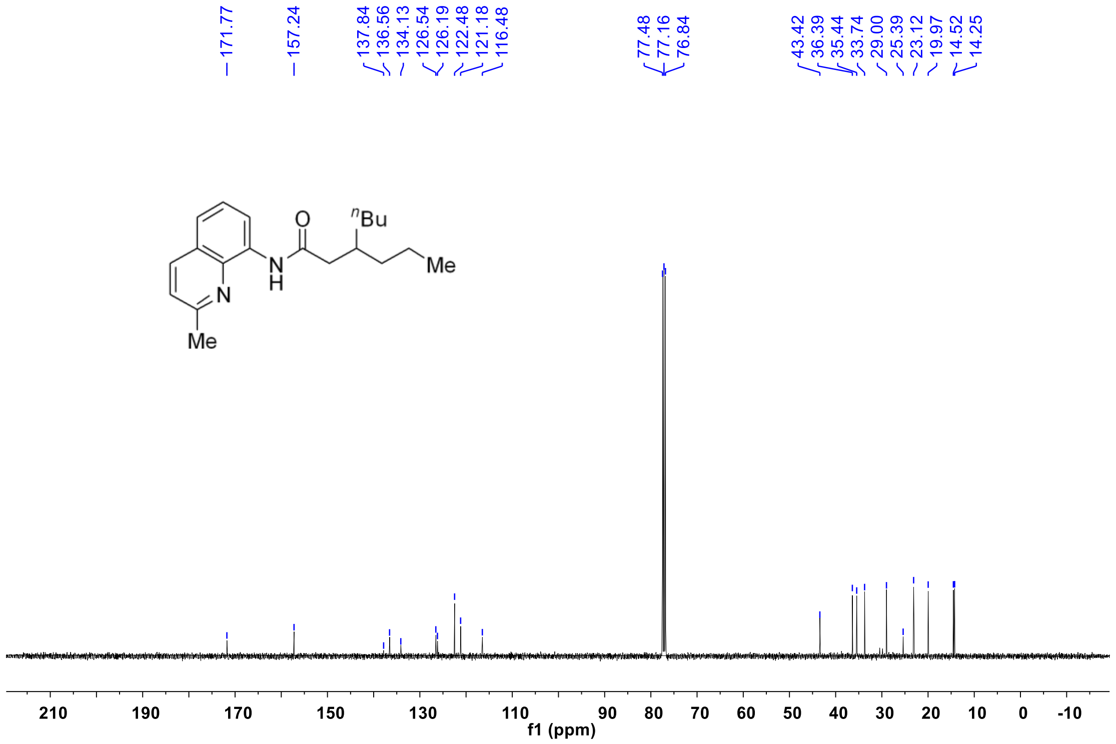

Supplementary Figure 110 ^{1}H NMR (500 MHz, $CDCl_3$) of **18e:**

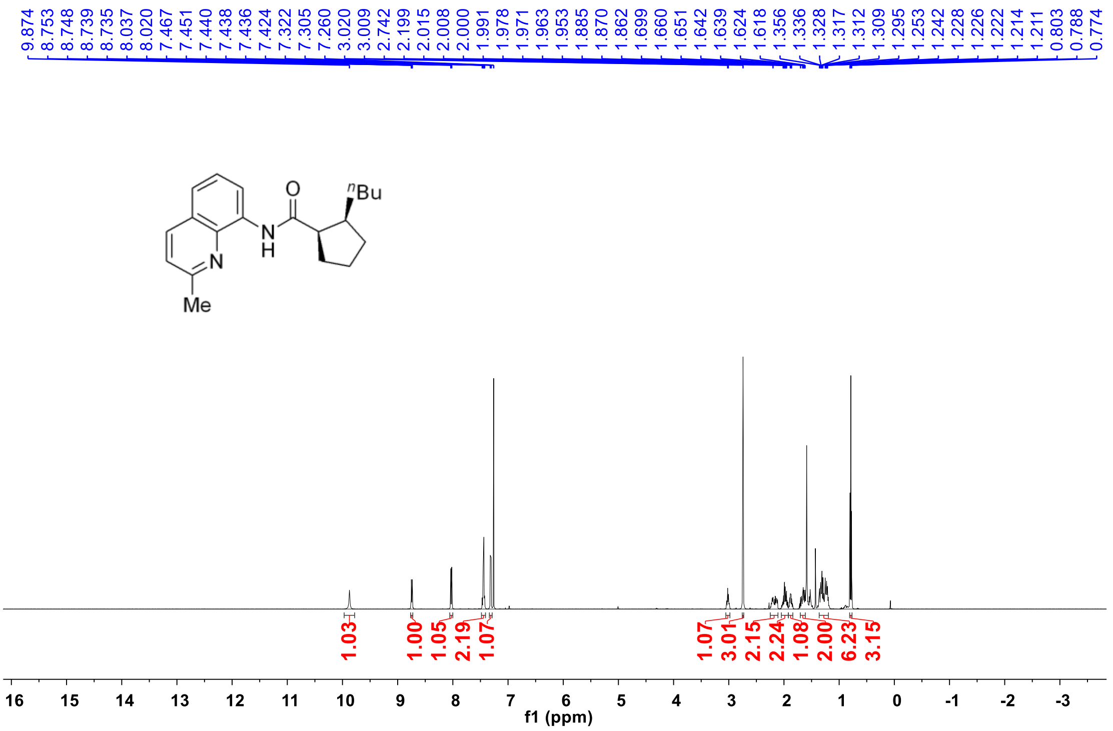

Supplementary Figure 111 ^{13}C NMR (125 MHz, $CDCl_3$) of **18e:**

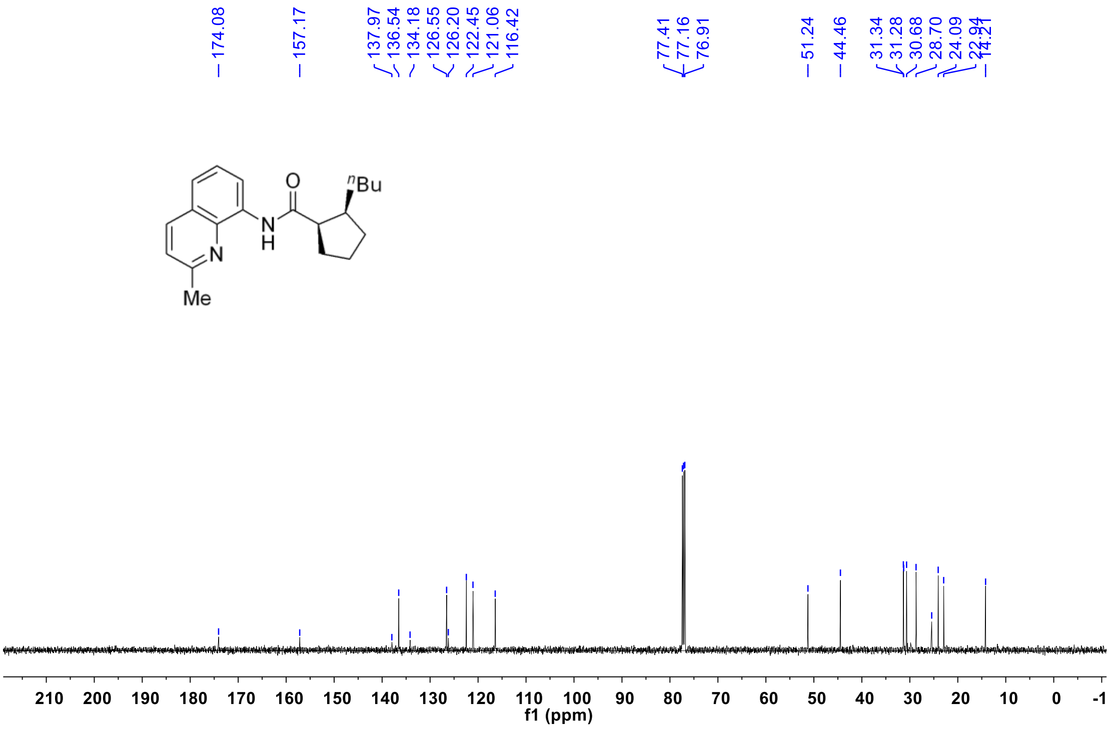

Supplementary Figure 112 NOESY of **18e:**

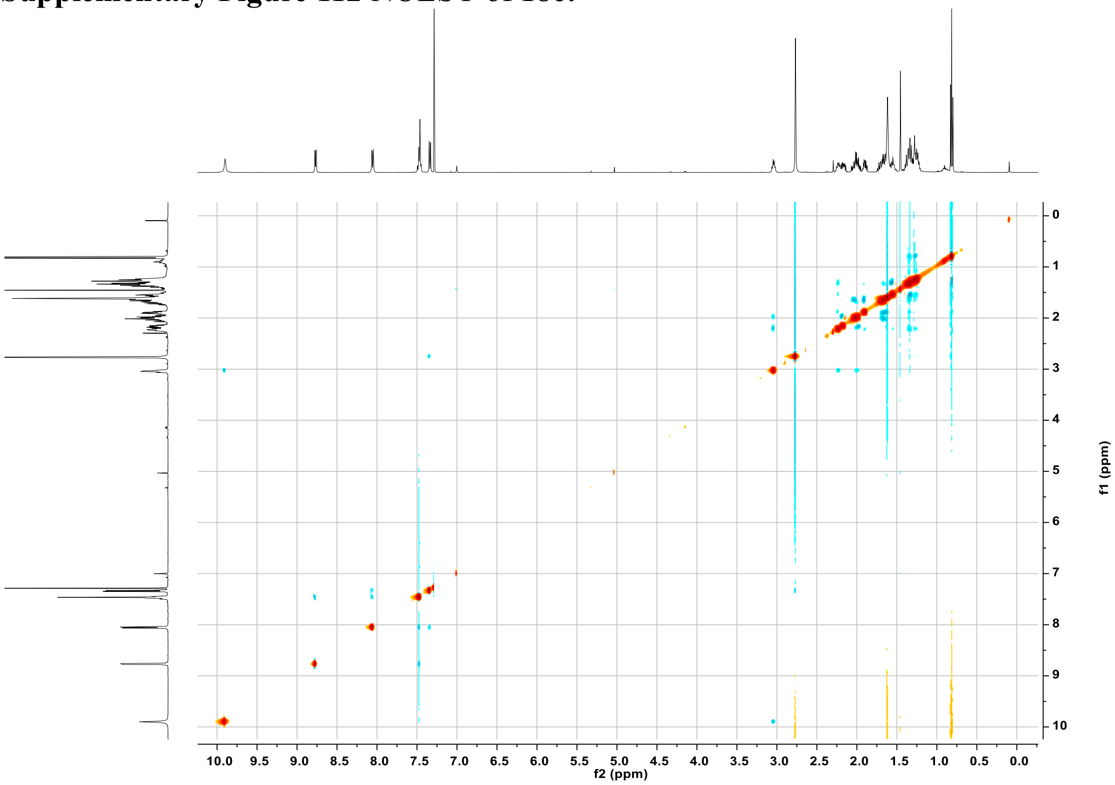

Supplementary Figure 113 ^{1}H NMR (500 MHz, $CDCl_3$) of **18f:**

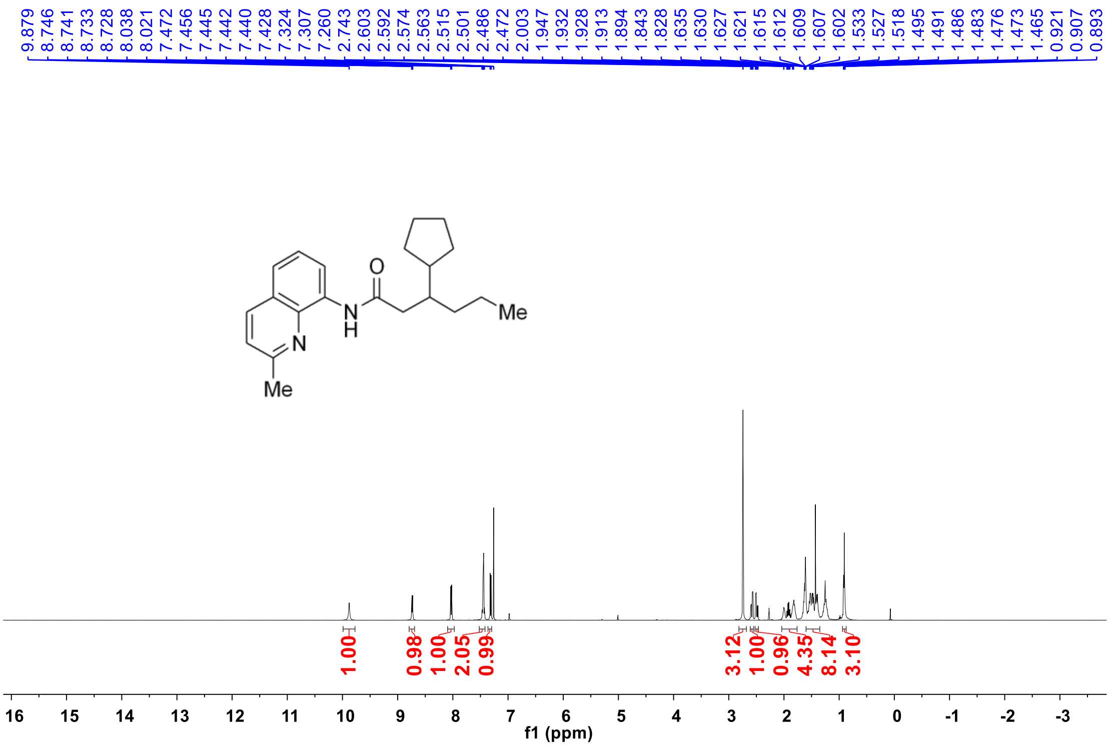

Supplementary Figure 114 ^{13}C NMR (100 MHz, $CDCl_3$) of **18f:**

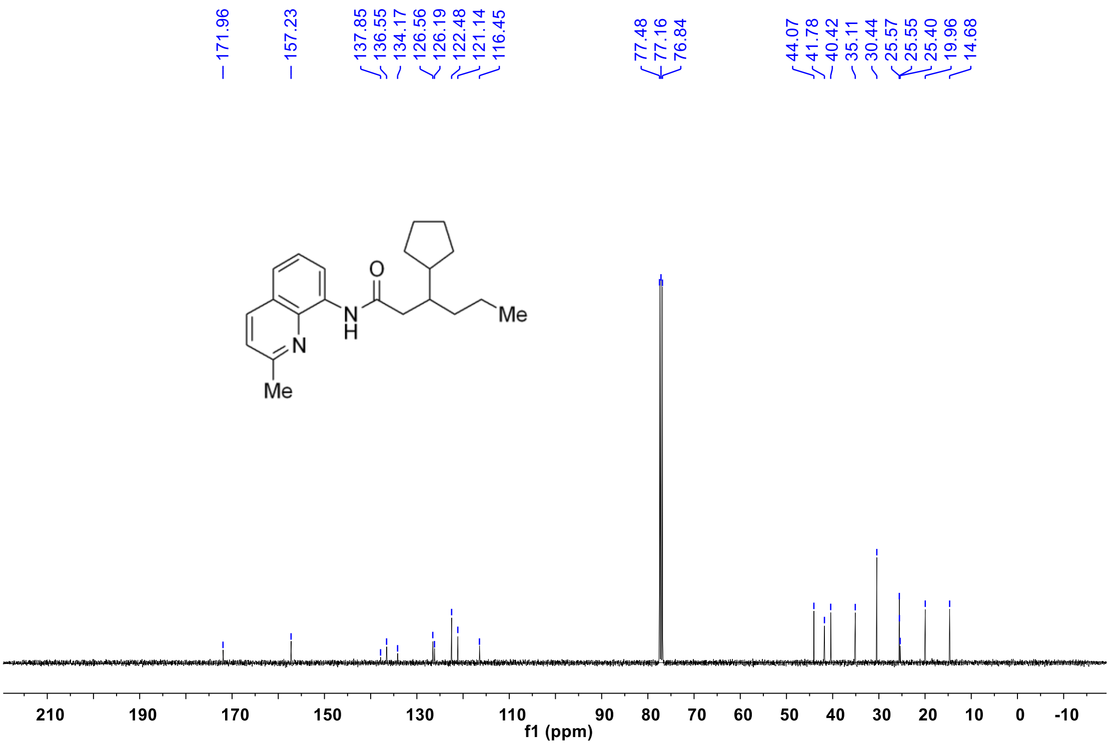

Supplementary Figure 115 ^{1}H NMR (400 MHz, $CDCl_3$) of **18g:**

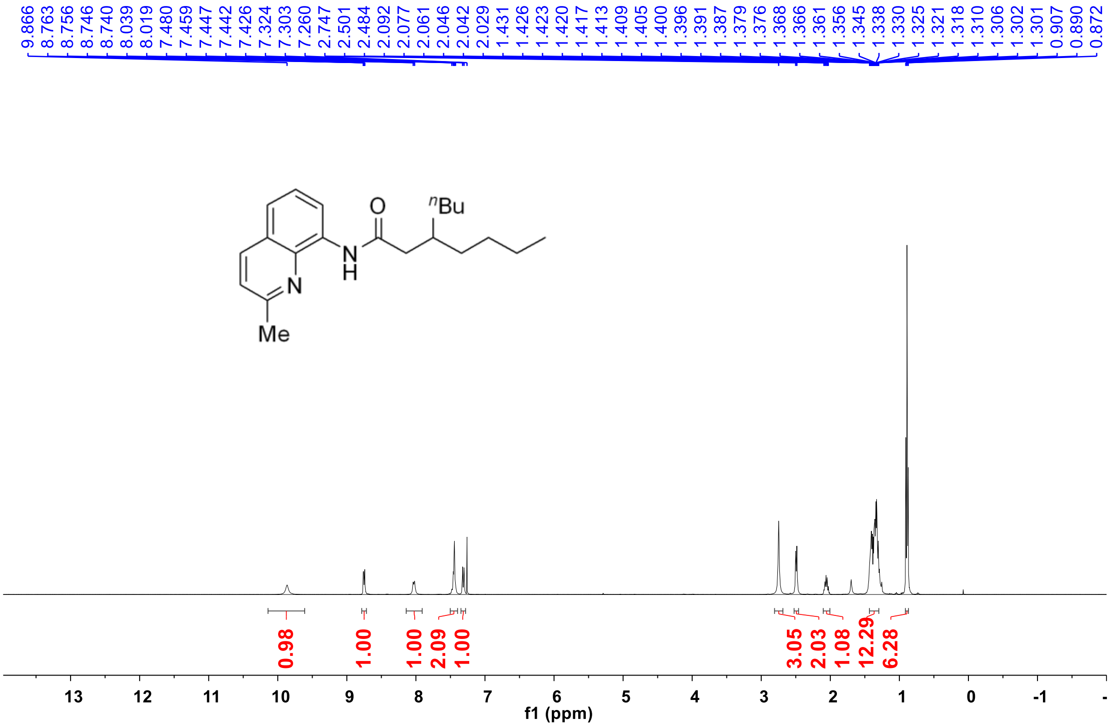

Supplementary Figure 116 ^{13}C NMR (100 MHz, $CDCl_3$) of **18g:**

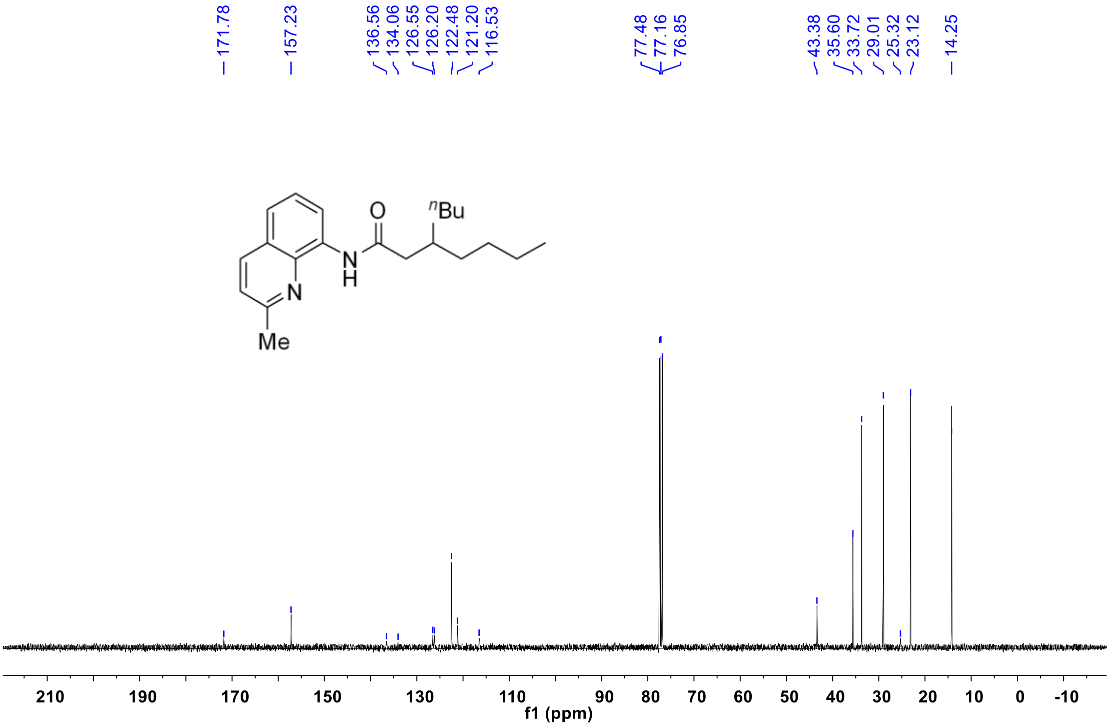

Supplementary Figure 117 ^{1}H NMR (500 MHz, DMSO-d_6) of **11:**

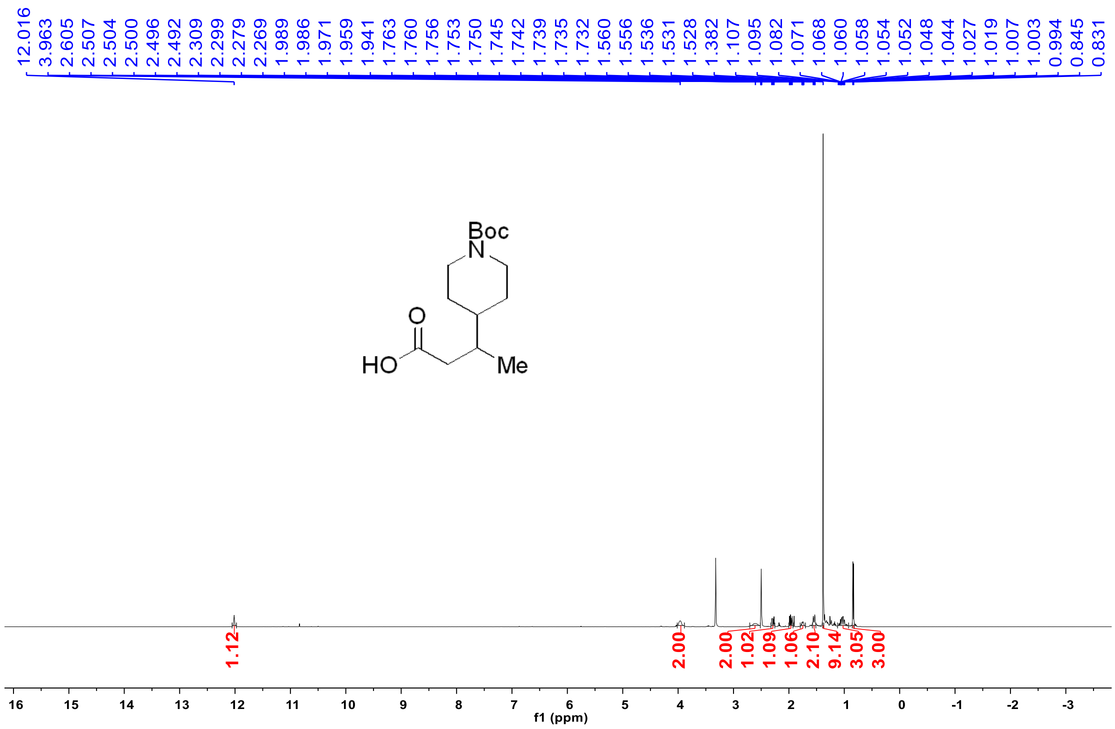

Supplementary Figure 118 ^{13}C NMR (125 MHz, $CDCl_3$) of **11:**

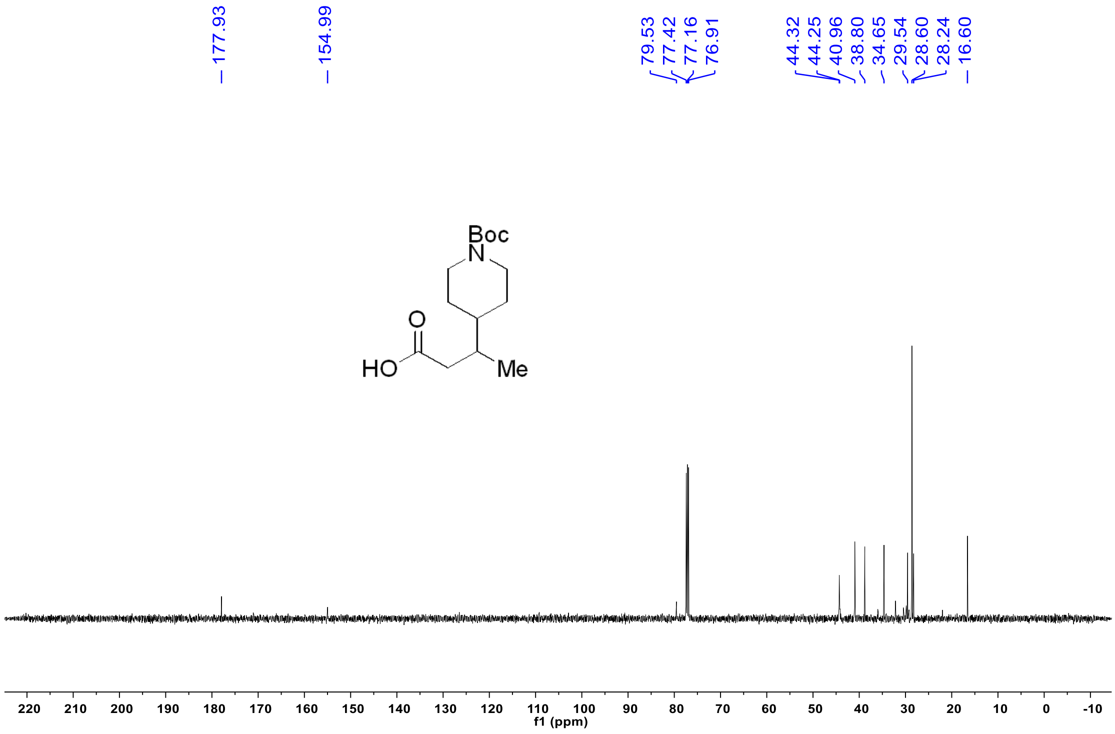

Supplementary Figure 119 ^{1}H NMR (500 MHz, $CDCl_3$) of ***d*-20:**

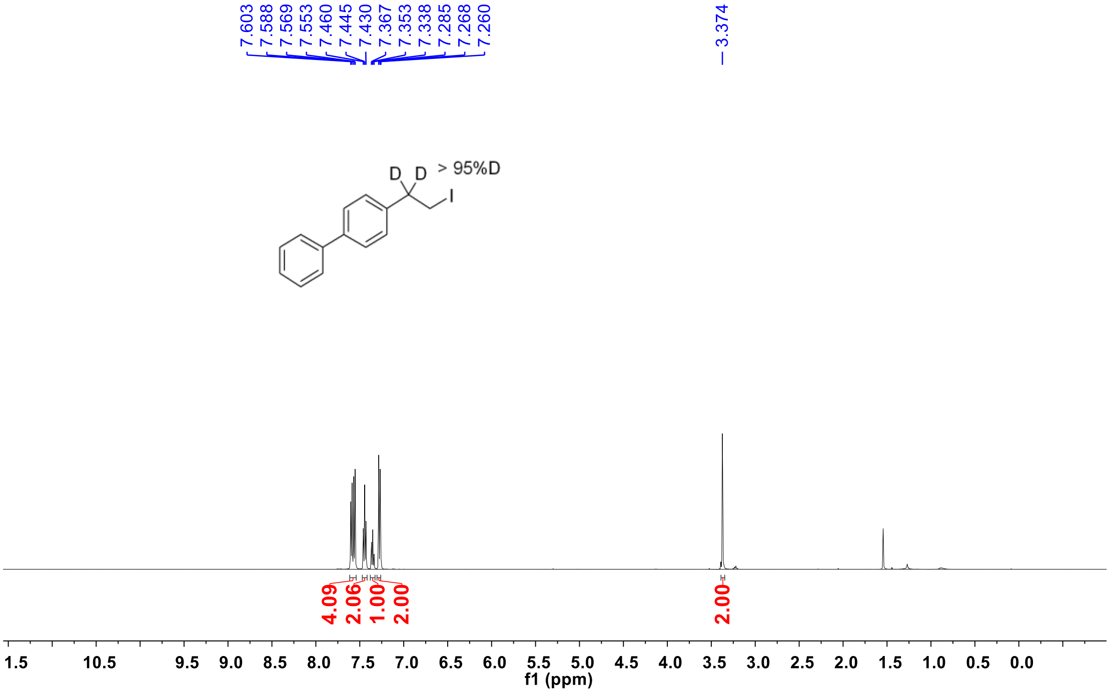

Supplementary Figure 120 ^{13}C NMR (125 MHz, $CDCl_3$) of ***d*-20:**

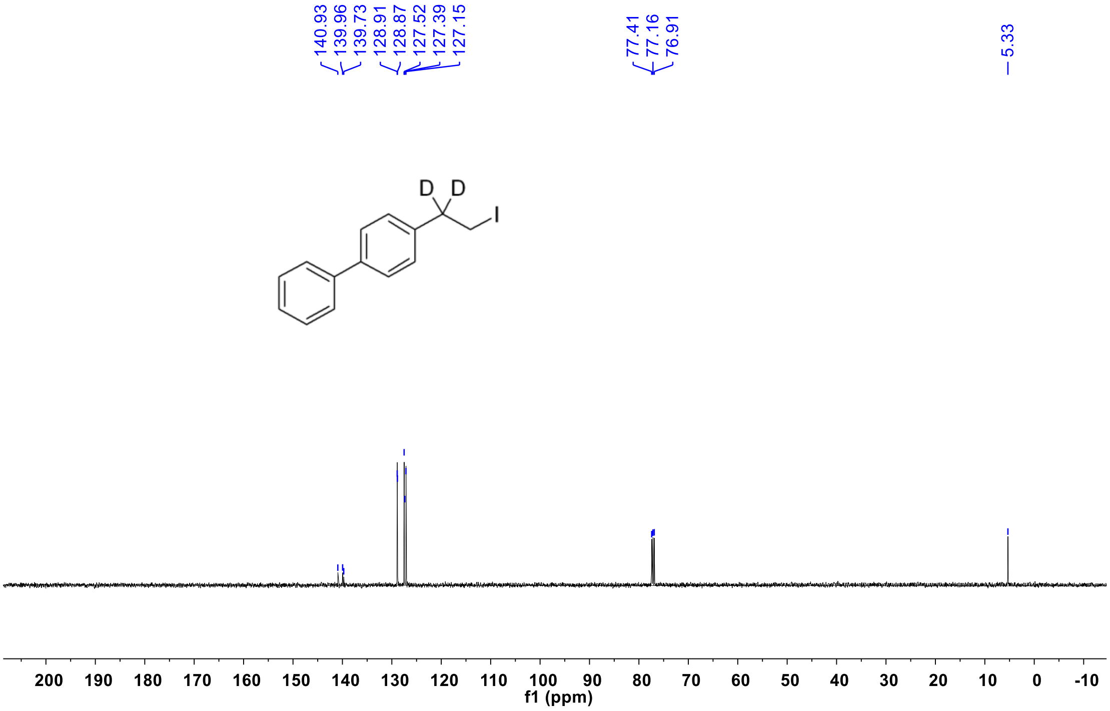

Supplementary Figure 121 ²D NMR (77 MHz, $CHCl_3$) of ***d*-20:**

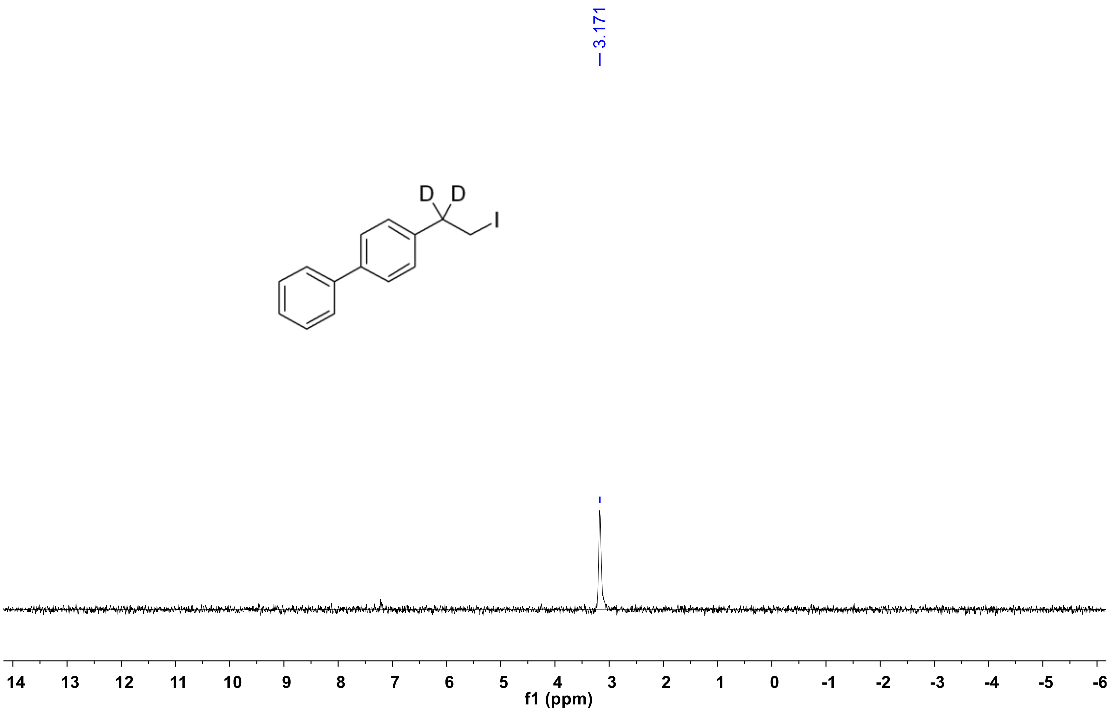

Supplementary Figure 122 ^{1}H NMR (500 MHz, $CDCl_3$) of ***d*-21:**

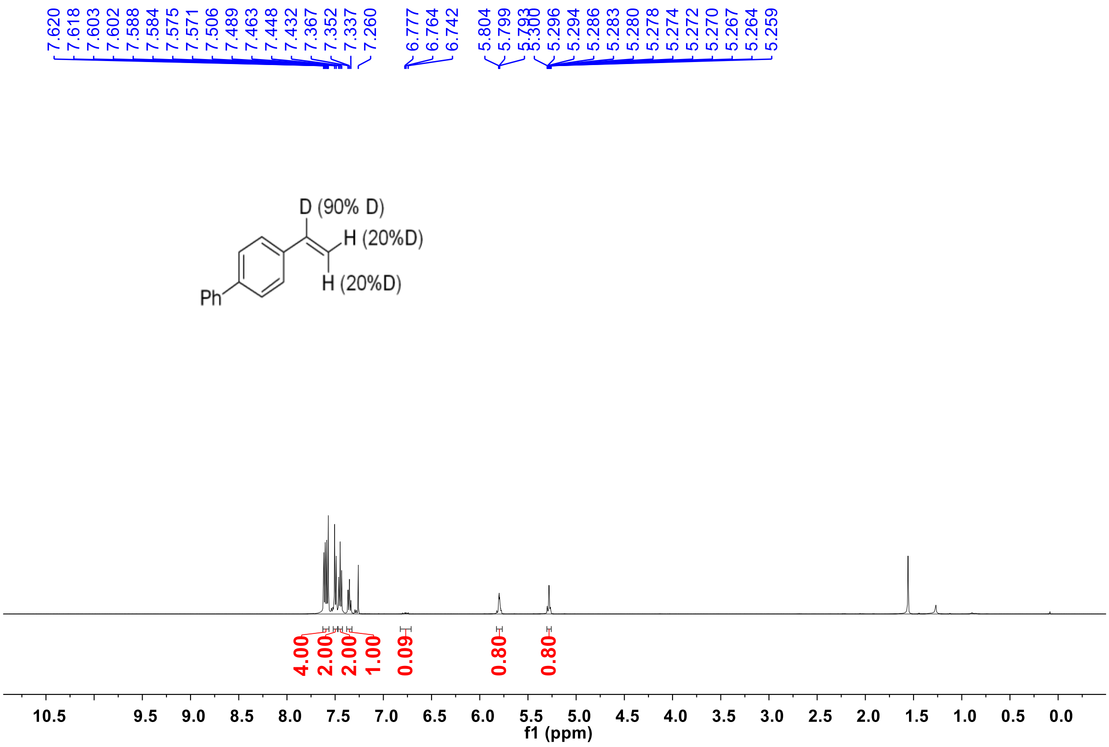

Supplementary Figure 123 ^{13}C NMR (100 MHz, $CDCl_3$) of ***d*-21:**

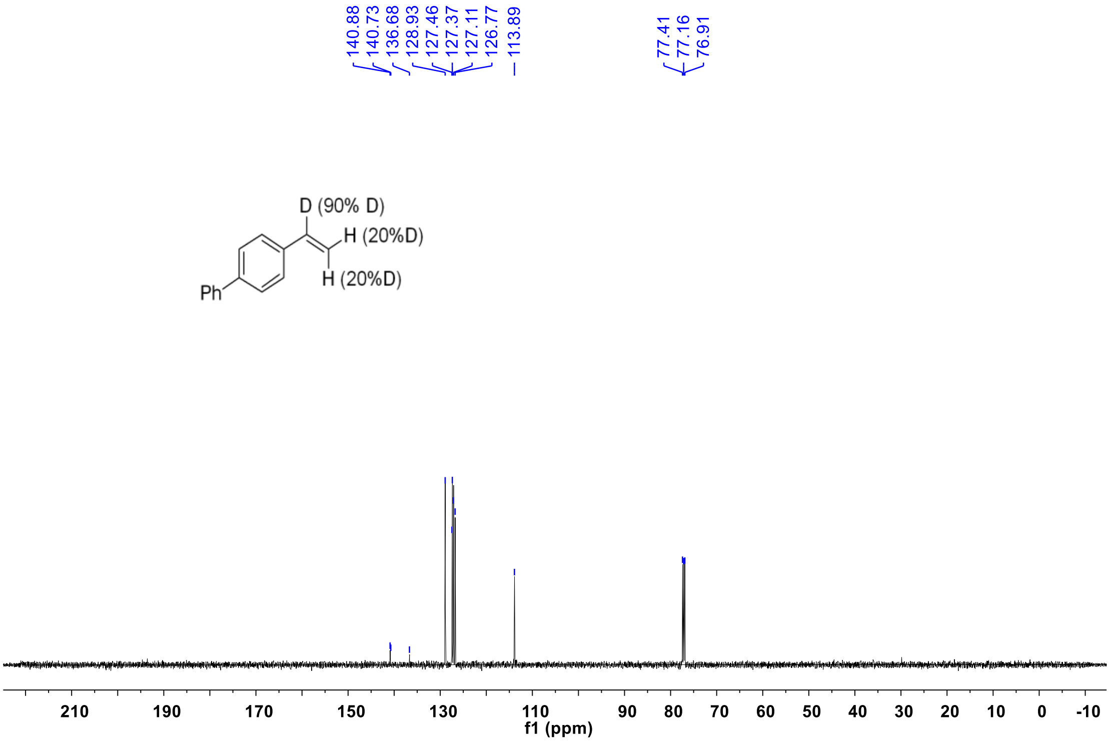

Supplementary Figure 124 ^{1}H NMR (500 MHz, $CDCl_3$) of ***d*-9aj:**

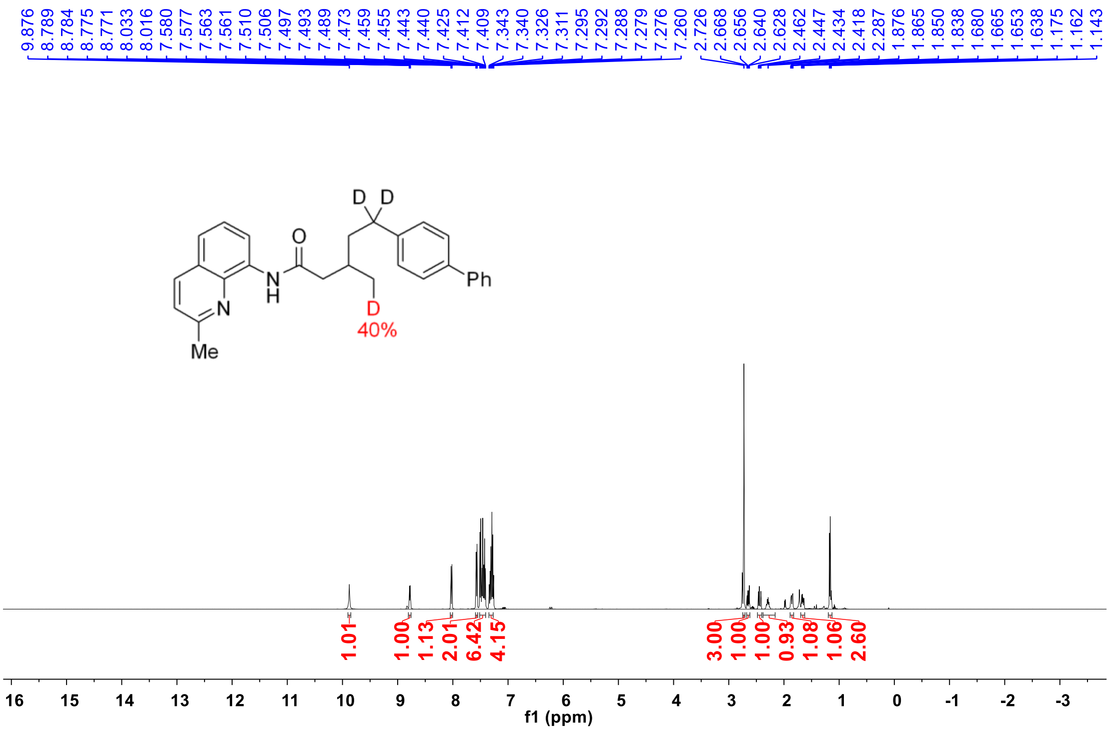

Supplementary Figure 125 ^{13}C NMR (100 MHz, $CDCl_3$) of ***d*-9aj:**

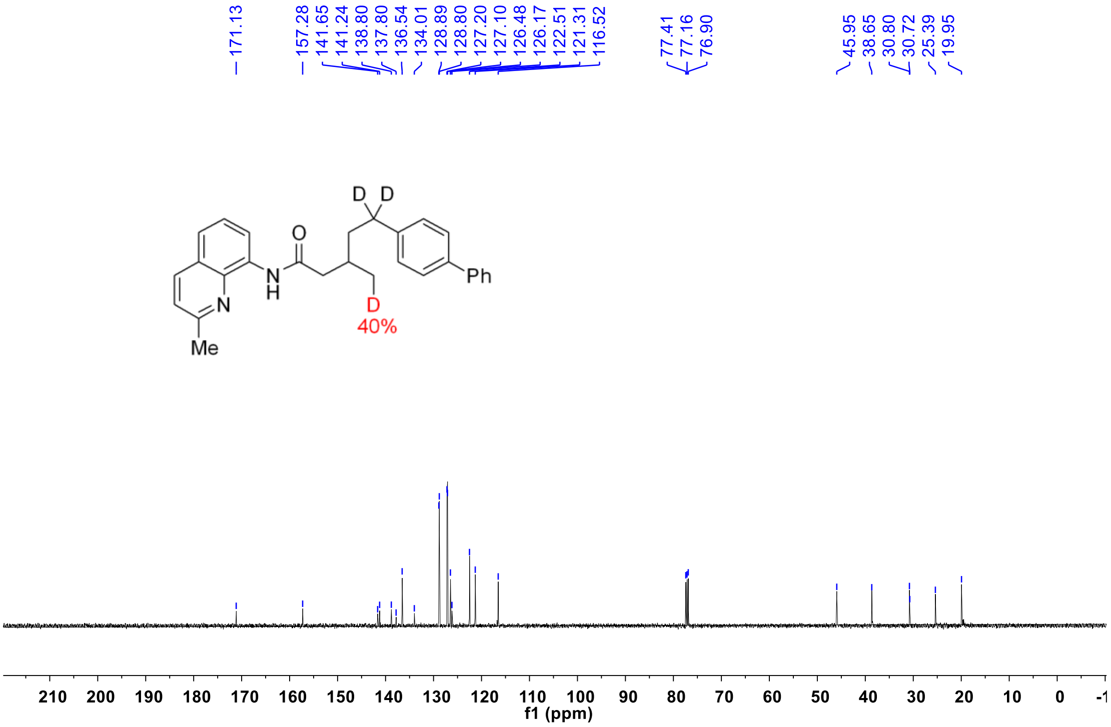

Supplementary Figure 126 ^{2}D NMR (77 MHz, $CHCl_3$) of ***d*-9aj:**

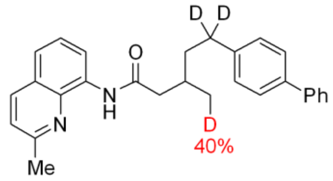

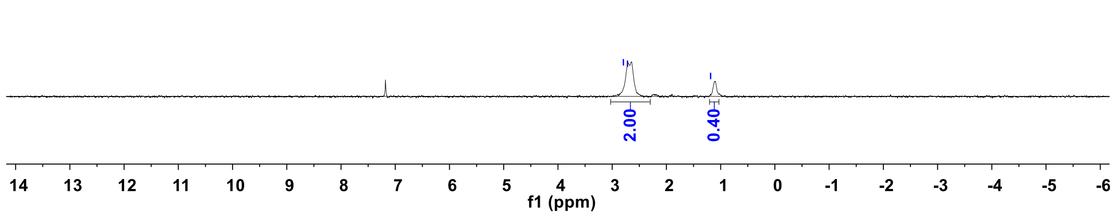

Supplementary Figure 127 ^{1}H NMR (500 MHz, $CDCl_3$) of **7a'**:

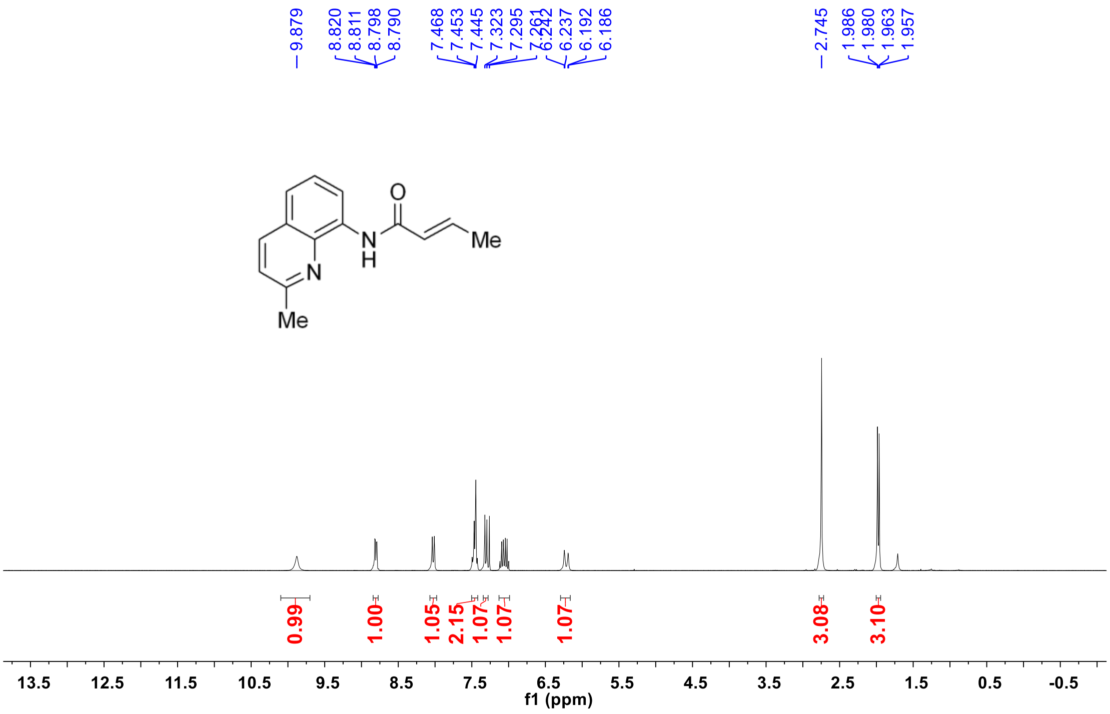

Supplementary Figure 128 ^{13}C NMR (100 MHz, $CDCl_3$) of **7a'**:

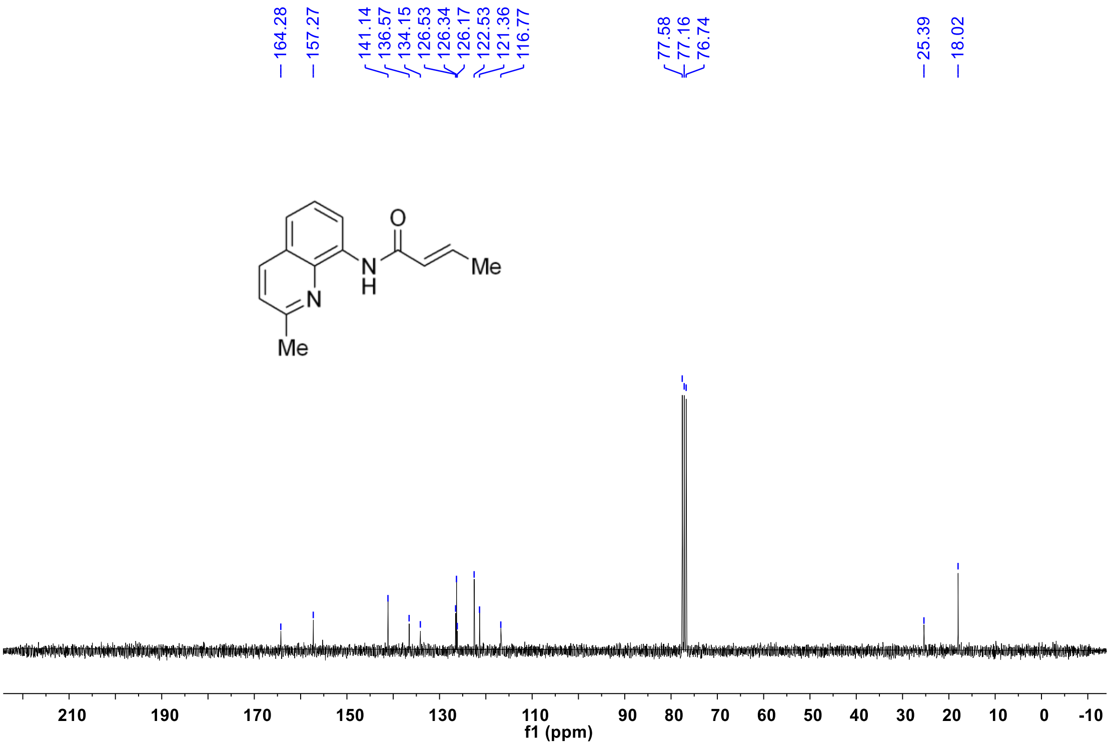

Supplementary Figure 129 ^{1}H NMR (500 MHz, $CDCl_3$) of **19**:

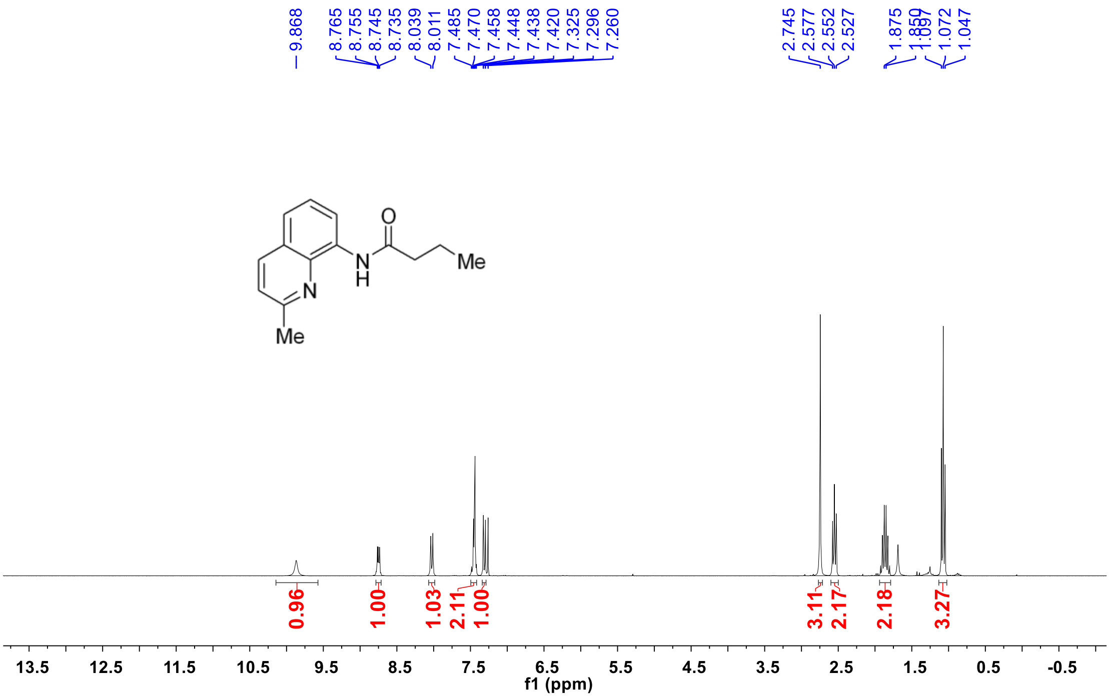

Supplementary Figure 130 ^{13}C NMR (100 MHz, $CDCl_3$) of **19**:

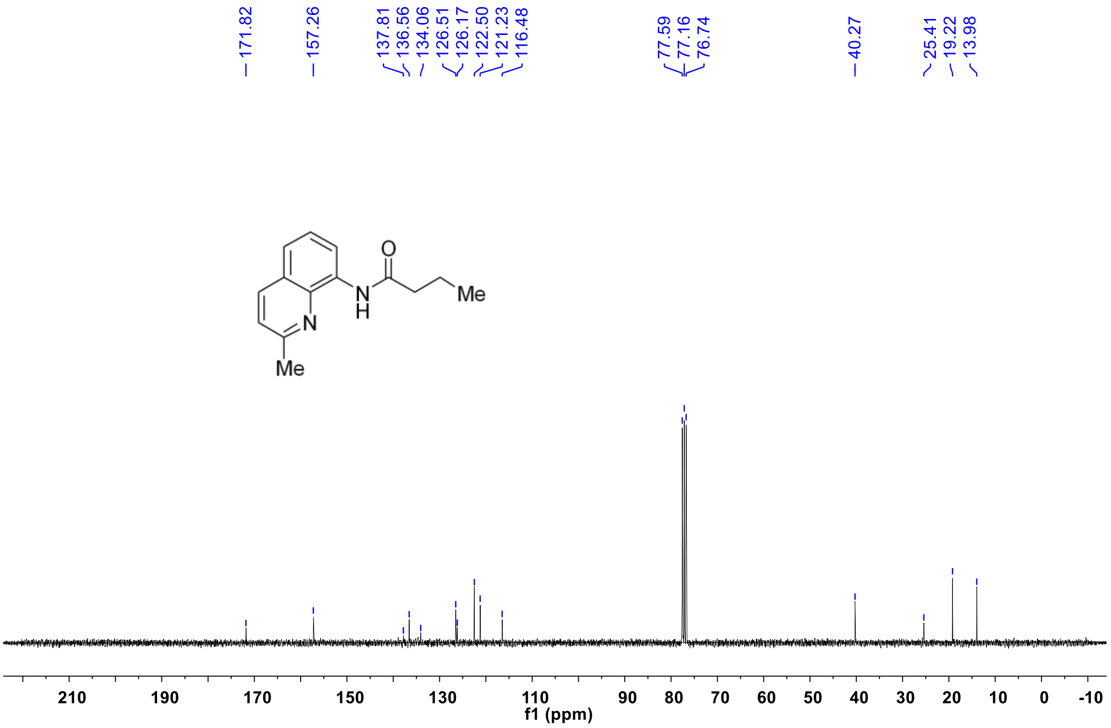

Supplementary Figure 131 ^{1}H NMR (500 MHz, $CDCl_3$) of **23**:

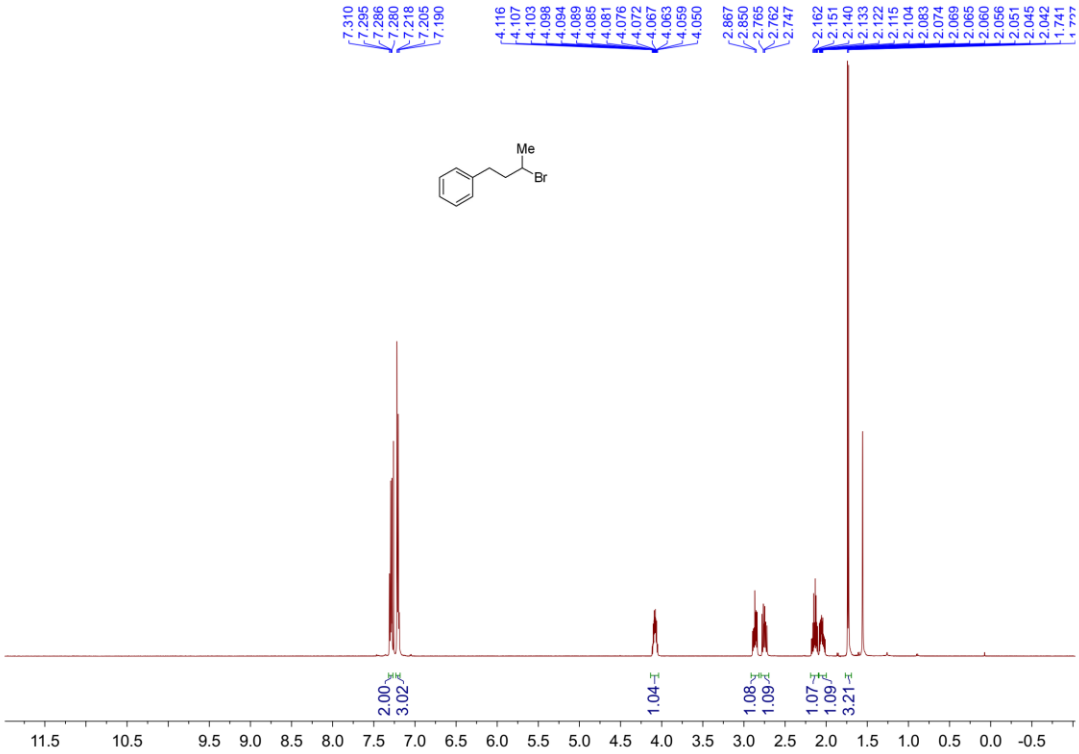

Supplementary Figure 132 ^{13}C NMR (126 MHz, $CDCl_3$) of **23**:

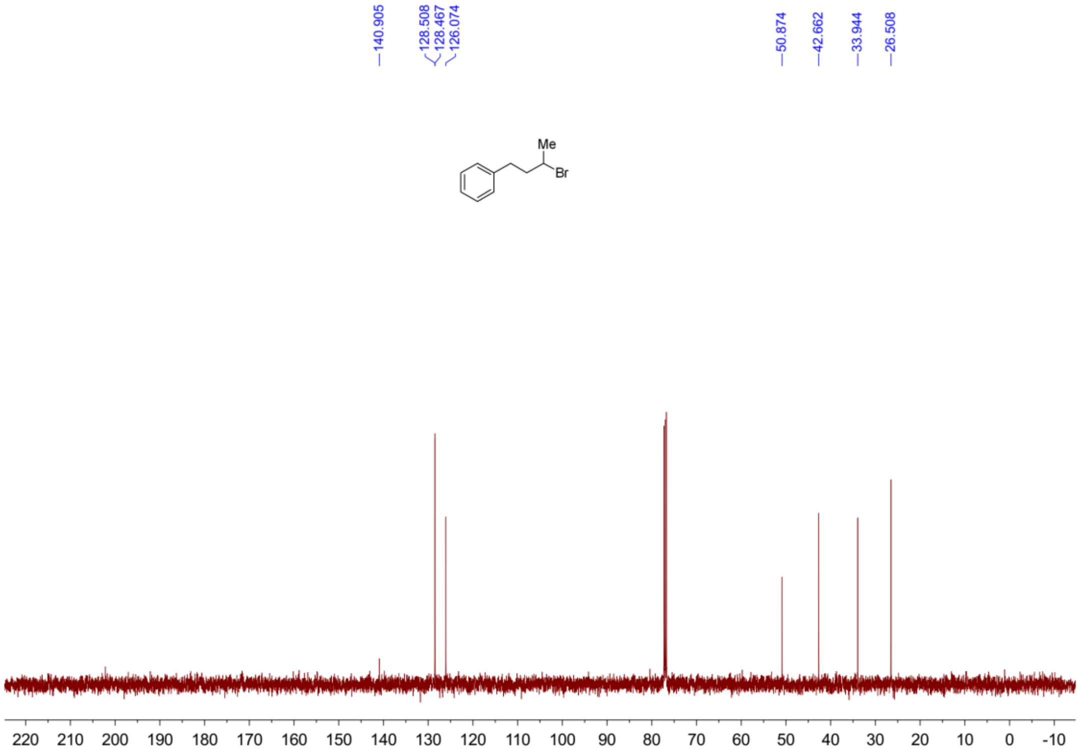

Supplementary Figure 133 ^{1}H NMR (500 MHz, $CDCl_3$) of **9al:**

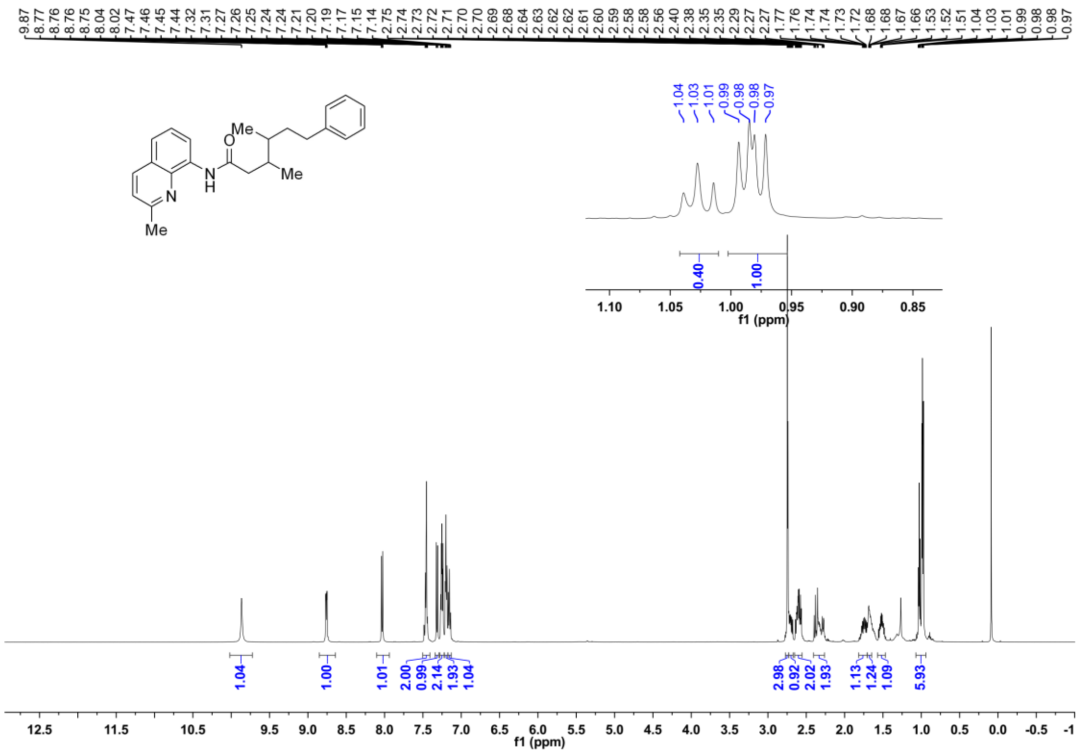

Supplementary Figure 134 ^{13}C NMR (126 MHz, $CDCl_3$) of **9al:**

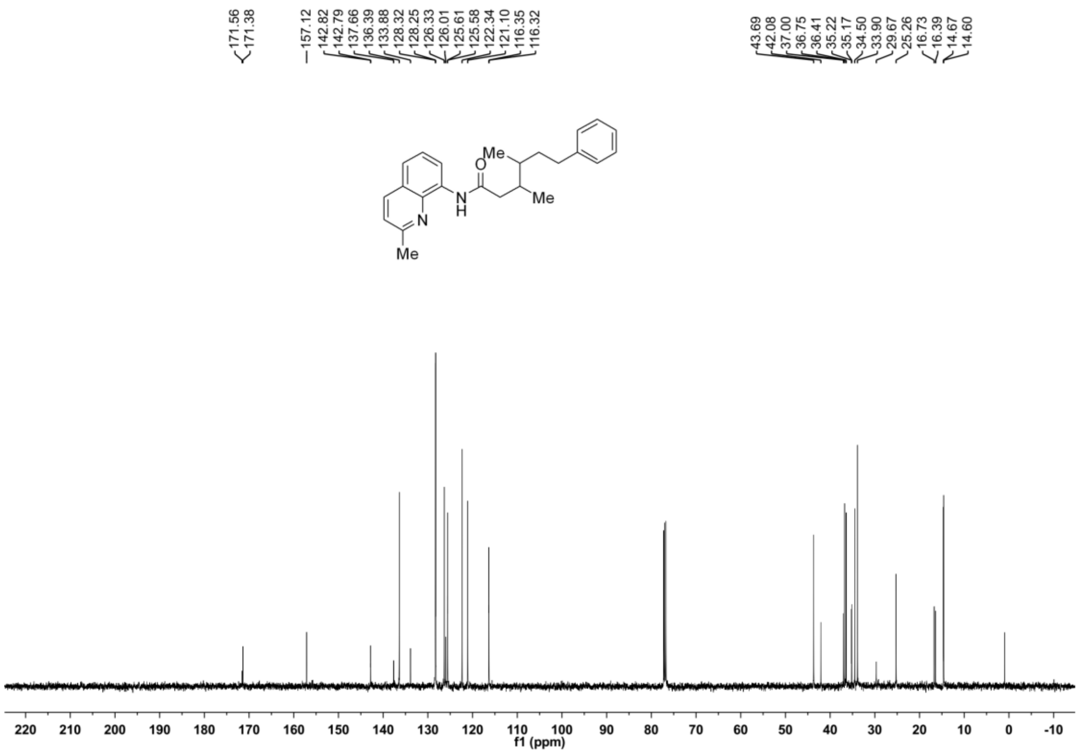

Supplementary Figure 135 ^{1}H NMR (400 MHz, $CDCl_3$) of **9w**：

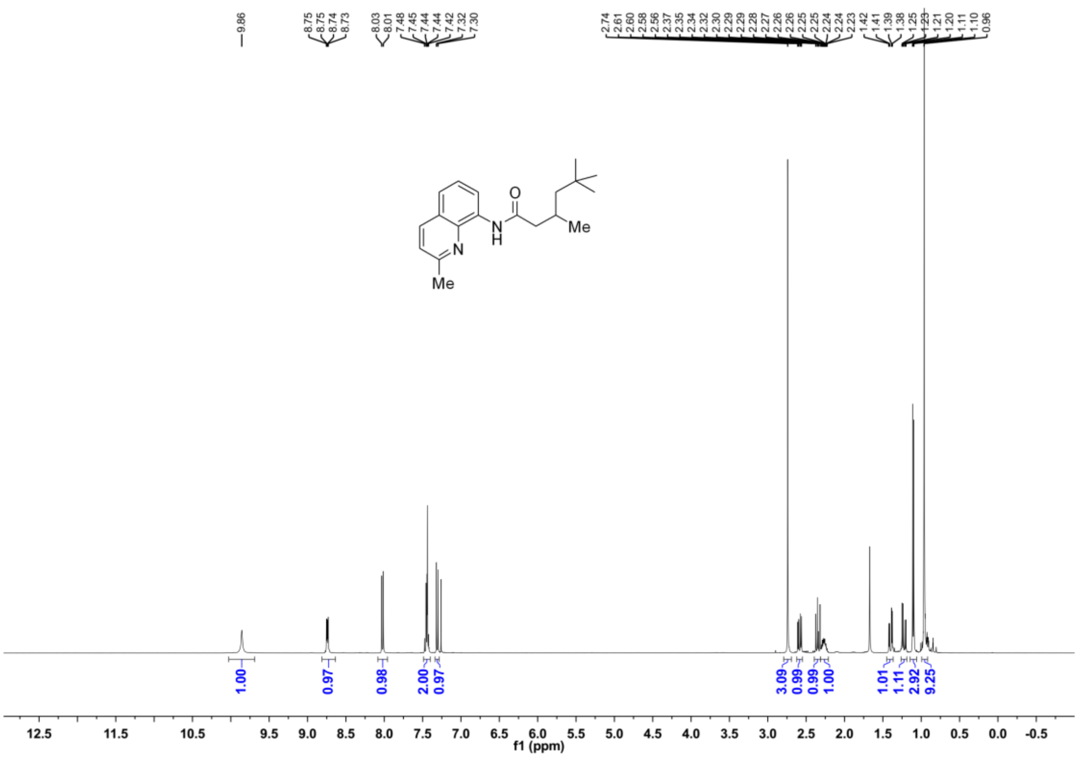

Supplementary Figure 136 ^{13}C NMR (101 MHz, $CDCl_3$) of **9w**：

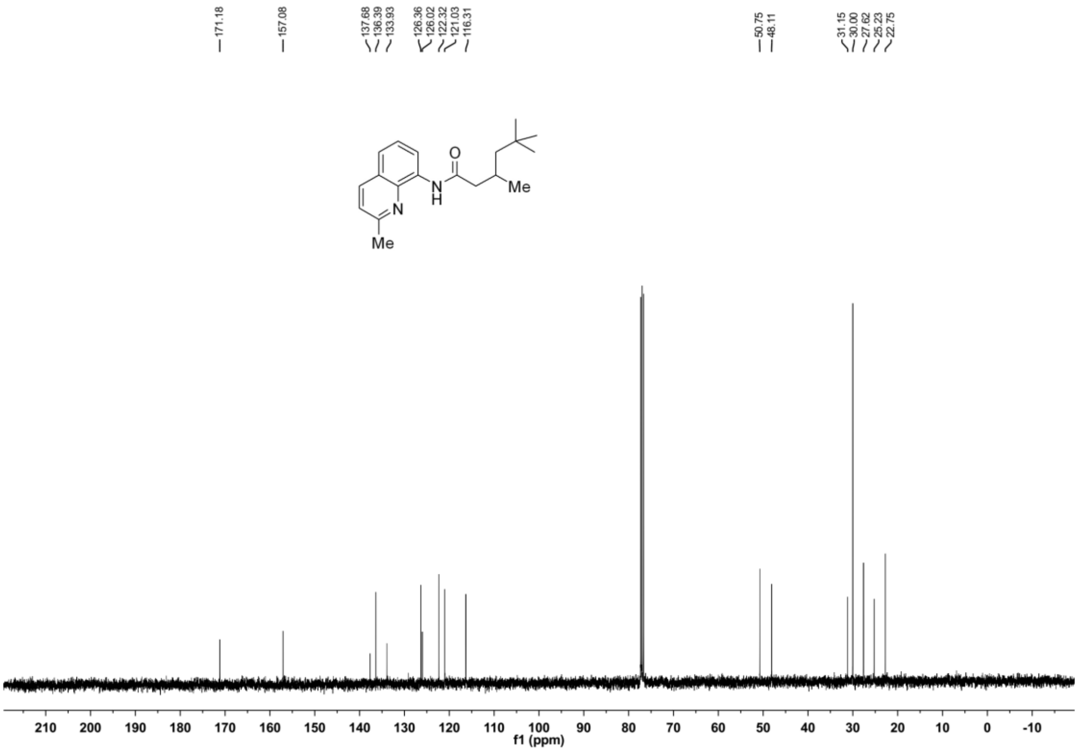

Supplementary Figure 137 ^{1}H NMR (500 MHz, $CDCl_3$) of **9w'**:

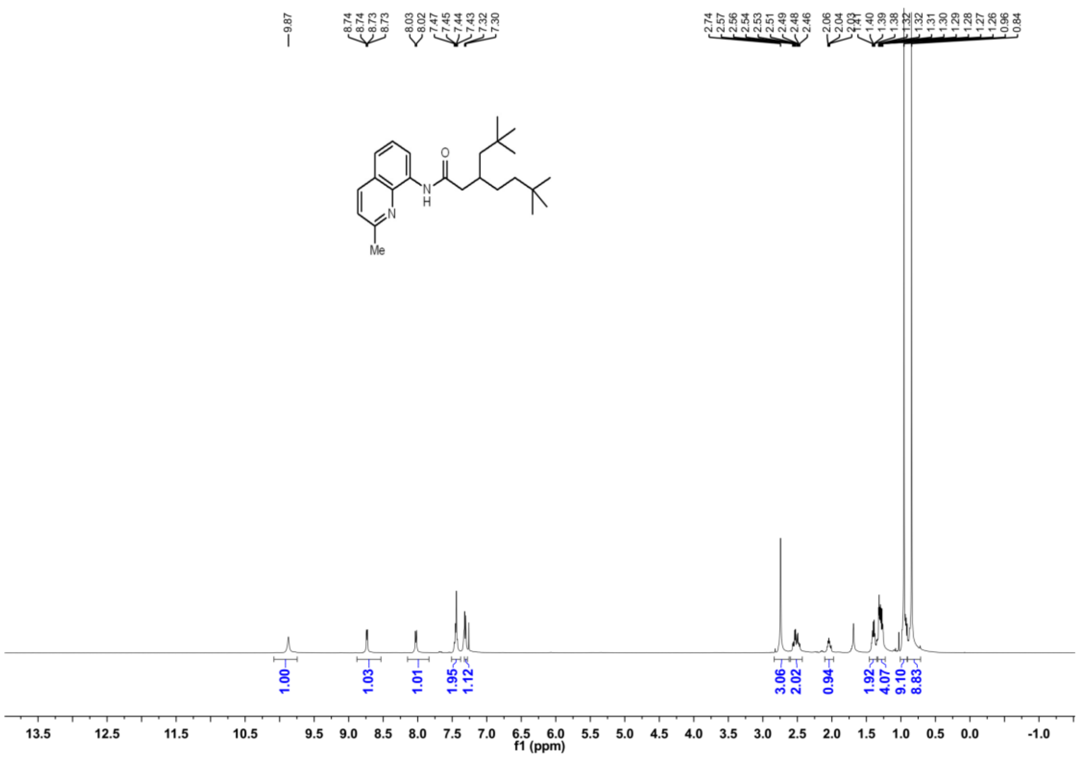

Supplementary Figure 138 ^{13}C NMR (126 MHz, $CDCl_3$) of **9w'**:

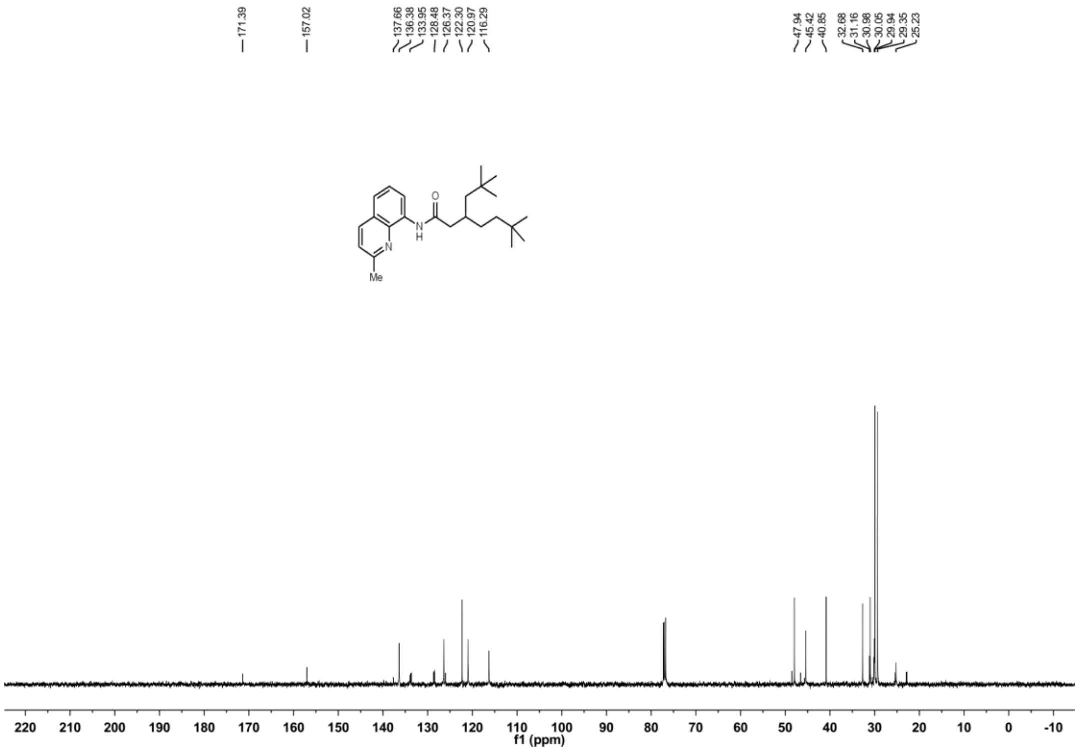

3. Supplementary References

1. Gurak, Jr. J. A., Jr.; Yang, K. S., Liu, Z. & Engle, K. M. Directed, regiocontrolled hydroamination of unactivated alkenes via protodepalladation. *J. Am. Chem. Soc.* **138**, 5805–5808 (2016).

2. Yang, K. S., Gurak, Jr. J. A., Liu, Z. & Engle, K. M. Catalytic, Regioselective hydrocarbofunctionalization of unactivated alkenes with diverse C–H nucleophiles. *J. Am. Chem. Soc.* **138**, 14705–14712 (2016).

3. Nimmagadda, S. K., Liu, M., Karunananda, M. K., Gao, D.-W., Apolinar, O., Chen, J. S., Liu, P. & Engle, K. M. Catalytic, enantioselective α-alkylation of azlactones with nonconjugated alkenes by directed nucleopalladation. *Angew. Chem. Int. Ed.* **58**, 3923–3927 (2019).

4. Verho, O., Maetani, M., Melillo, B., Zoller, J. & Schreiber, S. L. Stereospecific palladium-catalyzed C–H arylation of pyroglutamic acid derivatives at the C3 position enabled by 8-aminoquinoline as a directing Group. *Org. Lett.* **19**, 4424–4427 (2017).

5. Murai, M., Nishimuraa, K. & Takai, K. Palladium-catalyzed double-bond migration of unsaturated hydrocarbons accelerated by tantalum chloride. *Chem. Commun.* **55**, 2769–2772 (2019).

6. Kerr, W. J., Morrison, A. J., Pazicky, M. & Weber, T. Modified Shapiro reactions with bismesitylmagnesium as an efficient base reagent. *Org. Lett.* **14**, 2250–2253 (2012).

7. Cooper, P., Crisenza, G. E. M., Feron, L. J. & Bower, J. F. Iridium-catalyzed α-selective arylation of styrenes by dual C−H functionalization. *Angew. Chem. Int. Ed.* **57**, 14198–14202 (2018).

8. Sargent, B. T. & Alexanian, E. J. Palladium-catalyzed alkoxycarbonylation of unactivated secondary alkyl bromides at low pressure. *J. Am. Chem. Soc.* **138**, 7520−7523 (2016).

9. Lu, X., Xiao, B., Zhang, Z., Gong, T., Su, W., Yi, J., Fu, Y. & Liu, L. Practical carbon–carbon bond formation from olefins through nickel-catalyzed reductive olefin hydrocarbonation. *Nat. Commun.* **7**, 11129 (2016).

10. Zhou, F., Zhu, J., Zhang, Y. & Zhu, S. NiH-catalyzed reductive relay hydroalkylation: a strategy for the remote $C(sp^3)$–H alkylation of alkenes. *Angew. Chem. Int. Ed.* **57**, 4058–4062 (2018).
